# Isolating contiguous Pt atoms and forming Pt-Zn intermetallic nanoparticles to regulate selectivity in 4-nitrophenylacetylene hydrogenation

**DOI:** 10.1038/s41467-019-11794-6

**Published:** 2019-08-22

**Authors:** Aijuan Han, Jian Zhang, Wenming Sun, Wenxing Chen, Shaolong Zhang, Yunhu Han, Quanchen Feng, Lirong Zheng, Lin Gu, Chen Chen, Qing Peng, Dingsheng Wang, Yadong Li

**Affiliations:** 1 State Key Laboratory of Chemical Resource Engineering, Beijing University of Chemical Technology, Beijing, 100029 China; 2 Department of Chemistry, Tsinghua University, Beijing, 100084 China; 3 College of Science, China Agricultural University, Beijing, 100193 China; 4 Beijing Key Laboratory of Construction Tailorable Advanced Functional Materials and Green Applications, School of Materials Science and Engineering, Beijing Institute of Technology, Beijing, 100081 China; 5 Beijing Synchrotron Radiation Facility, Institute of High Energy Physics, Chinese Academy of Sciences, Beijing, 100049 China; 6 Institute of Physics, Chinese Academy of Sciences, Beijing, 100190 China

**Keywords:** Catalyst synthesis, Heterogeneous catalysis, Chemical engineering

## Abstract

Noble metals play a momentous role in heterogeneous catalysis but still face a huge challenge in selectivity control. Herein, we report isolating contiguous Pt atoms and forming Pt-Zn intermetallic nanoparticles as an effective strategy to optimize the selectivity of Pt catalysts. Contiguous Pt atoms are isolated into single atoms and Pt-Zn intermetallic nanoparticles are formed which are supported on hollow nitrogen-doped carbon nanotubes (PtZn/HNCNT), as confirmed by aberration-corrected high-resolution transmission electron microscopy and X-ray absorption spectrometry measurements. Interestingly, this PtZn/HNCNT catalyst promotes the hydrogenation of 4-nitrophenylacetylene to 4-aminophenylacetylene with a much higher conversion ( > 99%) and selectivity (99%) than the comparison samples with Pt isolated-single-atomic-sites (Pt/HNCNT) and Pt nanoparticles (Pt/CN). Further density functional theory (DFT) calculations disclose that the positive Zn atoms assist the adsorption of nitro group and Pt-Zn intermetallic nanoparticles facilitate the hydrogenation on nitro group kinetically.

## Abstract (web summary)

Noble metals play a momentous role in heterogeneous catalysis but still face a huge challenge in selectivity control. Herein, the authors demonstrate that isolating contiguous Pt atoms and forming Pt-Zn intermetallic nanoparticles is an effective strategy to optimize the selectivity of Pt catalysts.

## Introduction

Although noble metals are widely applied as heterogeneous catalysts in industrial processes, they are generally less selective to obtain the desired products with high yields^1–4^. Due to their specific electronic structure, traditional noble catalysts are usually highly active, but have great difficulty in discriminating competitive functional groups in many cases^5–15^. For instance, Pt catalyzed selective hydrogenation of the nitro group in nitrophenylacetylenes to obtain aminophenylacetylenes is a vital process in the production of antitumor agent Erlotinib and intermediates of fluorescent labels^16–18^. However, conventional Pt catalysts usually hydrogenate the highly reductive alkynyl group and the nitro group simultaneously, leading to a poor selectivity of desired products. Hence, exploiting effective strategies to modulate the selectivity of such Pt catalysts has drawn extensive research attention.

Tuning the electronic structure of noble metals has been emerging as an efficient strategy to optimize their catalytic selectivity^19–22^. Through this strategy, the adsorption/desorption properties of the relevant reaction species can be changed and the catalytic reaction pathway may be altered to achieve the ideal catalytic performance. So far, several approaches have been developed to adjust the electronic structures of noble metals, such as using surface organic ligand modifiers^23–25^, and creating metal–support interactions^26–31^. Besides, the construction of intermetallic compounds (IMCs) represents another powerful and flexible tool in this respect^32–44^. Upon precise design, noble metal atoms might be isolated into single atom on intermetallic structures orderly with the insertion of an inert metal (IM). Moreover, the formed noble metal–IM (NM–IM) bonds might alter the adsorption/desorption ability of reaction species and the reaction pathway, leaving possibility of catalyzing the reaction in the desired direction. Yet, researches devoted to improve the catalytic selectivity of NMs by this isolating contiguous NM atoms and forming NM–IM intermetallic nanoparticles strategy are still scarce.

Herein, we report isolating contiguous Pt atoms and forming Pt–Zn intermetallic nanoparticles effectively regulate the selectivity of Pt catalysts. PtZn IMCs supported on hollow nitrogen doped carbon nanotubes (PtZn/HNCNT) are synthesized by a sacrificial template method. Interestingly, this PtZn/HNCNT greatly increases the catalytic conversion (>99%) and selectivity (99%) in hydrogenation of 4-nitrophenylacetylene (4-NPA) to 4-aminophenylacetylene (4-APA) with ammonia borane as the hydrogenation source, outperforming the samples with Pt isolated-single-atomic-sites (Pt/HNCNT) and Pt nanoparticles (Pt/CN). Density functional theory (DFT) calculation reveals that the adsorption of nitro group is enhanced due to the Zn atoms, and the hydrogenation on nitro group is kinetically favored due to the Pt–Zn intermetallic nanoparticles.

## Results

### Synthesis and characterization of the catalyst

**Fig. 1:**
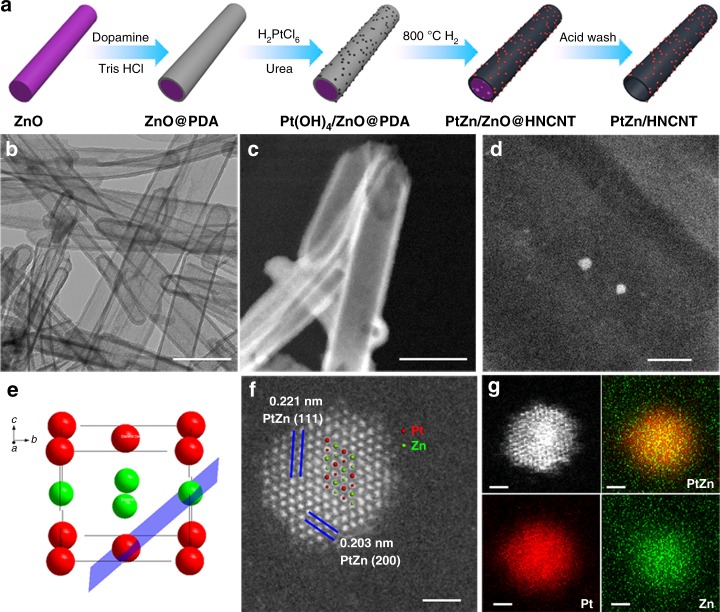
Synthetic scheme and characterization of the catalyst. **a** Synthetic strategy of PtZn intermetallic nanoparticles supported on hollow nitrogen-doped carbon nanotubes (PtZn/HNCNT). **b**–**d**, Transmission electron microscope (TEM) (**b**), high angel annular dark field scanning TEM (HAADF STEM) (**c**), and aberration-corrected (AC) HAADF STEM (**d**) images of PtZn/HNCNT. Scale bar, 500 nm (**b**); 200 nm (**c**); 10 nm (**d**). **e** Crystal structure of PtZn intermetallic compound (IMC) (Pt: red; Zn: green). **f**, **g** AC HAADF STEM (**f**) image and elemental mappings (**g**) of a PtZn IMC nanoparticle. Scale bar, 1 nm

PtZn/HNCNT was fabricated through a sacrificial template method (Fig. 1a). First, ZnO nanorods were coated with a thin layer of polydopamine. Second, Pt(OH)_4_ was loaded on the outlayer through a precipitation method. The as-prepared Pt(OH)_4_/ZnO@PDA was then reduced by hydrogen at 800 °C to form PtZn IMCs, which is the critical step in this strategy. In this step, the ZnO was reduced to zinc vapor, which assists the contiguous Pt atoms isolated by Zn atoms to form the PtZn IMCs at high temperature. Finally, an acid wash step was carried out to remove remaining ZnO.

Electron microscope was applied to observe the sample morphologies in the synthetic process. A ~20–30 nm thin layer of PDA was coated on the outside of ZnO nanorods after step 1 (Supplementary Fig. 1). No obvious nanoparticles existed in this layer after step 2, indicating the ultrasmall size of the Pt(OH)_4_ (Supplementary Fig. 2). After high-temperature treatment and acid wash, HNCNTs were formed with 2–6 nm nanoparticles supported on them (Fig. 1b–d and Supplementary Fig. 3). Aberration-corrected high-angle annular dark-field scanning transmission electron microscope (AC HAADF STEM) images were taken to measure the particle distribution (Supplementary Fig. 4), and the average particle size was 2.24 nm. XRD was then performed to examine the phase composition of the samples (Supplementary Fig. 5). Only graphitic carbon peaks were observed because of the ultrasmall size of the PtZn particles. A sample with higher Pt loading (1%) is prepared using the same method. The diffraction peaks in XRD (Supplementary Fig. 6) match well to that of PtZn (JCPDS No. 06-0604). To further testify the IMC nature of PtZn particles, AC HAADF STEM measurement was carried out. AC HAADF STEM of one PtZn nanoparticle exhibited clear bright dots, in agreement with the atomic arrangement of $(02\bar 2)$ plane in the PtZn crystal structure (Fig. 1e). The lattice spacing of 0.221 and 0.203 nm matched interplane distances of (111) and (200) planes of intermetallic PtZn (P4/mmm) respectively (Fig. 1f). The perfect agreement testified the nanoparticle is PtZn IMC. More AC HAADF STEM images are taken to prove that the nanoparticles are PtZn IMC. As shown in Supplementary Fig. 7, the atomic arrangement of another nanoparticle could match well with the (010) plane of PtZn. Due to the different atomic density, the heavier Pt atoms (bright) can be distinguished from Zn atoms (darker). Therefore, we think that the PtZn IMCs instead of core-shell alloys are formed in the PtZn/HNCNT. Further energy-dispersive X-ray (EDX) elemental mapping (Fig. 1g) analysis exhibited the simultaneous presence and homogeneous dispersion of Pt and Zn, indicating the uniform composition of the particle. For comparison, Pt/HNCNT was also prepared in a similar procedure except reduced at a low temperature (200 °C), where only Pt component is present on the HNCNT support due to the absence of Zn vapor (its characterizations are shown in Supplementary Figs. 8–12). AC HAADF STEM image (Supplementary Fig. 12) shows only bright dots on the support, indicating that the Pt component exists in the form of isolated-single-atomic-sites. Pt/CN, prepared by a similar method using polydopamine nanospheres as the support and treated in H_2_/Ar atmosphere at 800 °C, contains obvious Pt nanoparticles, as deduced from XRD, TEM, and AC HAADF STEM (Supplementary Fig. 13). These results prove that Pt atoms tend to aggregates upon high temperature, and the presence of ZnO helps to isolate contiguous Pt atoms by forming Pt–Zn bridge sites in the PtZn intermetallic nanoparticles. Using the similar method, PdZn/HNCNT with PdZn intermetallic nanoparticles supported on HNCNT was successfully obtained (its characterizations are shown in Supplementary Fig. 14), proving the generality of this strategy to synthesize IMCs.

**Fig. 2:**
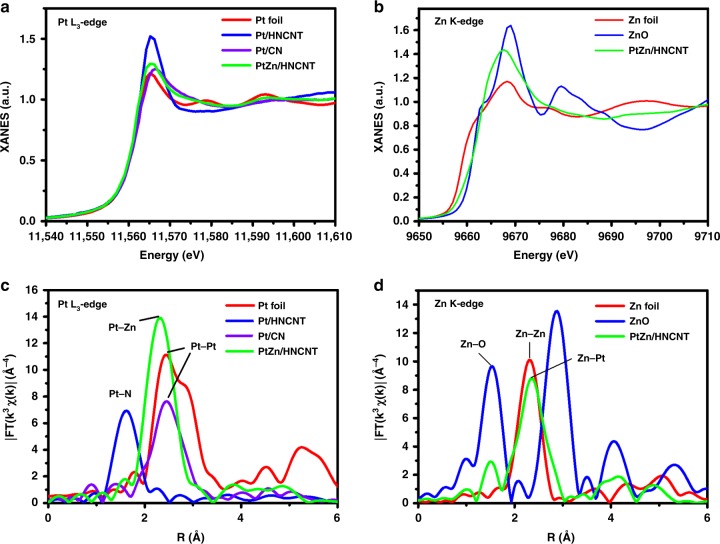
X-ray absorption spectroscopy characterization of the catalysts. **a**, **c** The normalized X-ray absorption near-edge structure (XANES) spectra (**a**) and Fourier transform extended X-ray absorption fine structure (FT-EXAFS) (**c**) at the Pt L_3_-edge of the PtZn/HNCNT, Pt isolated-single-atomic-site supported on hollow nitrogen-doped carbon nanotubes (Pt/HNCNT), Pt nanoparticles supported on nitrogen-doped carbon nanospheres (Pt/CN) and Pt foil. **b**, **d** The normalized XANES spectra (**b**) and FT-EXAFS (**d**) at the Zn K-edge of the PtZn/HNCNT, ZnO and Zn foil

To verify the different electronic structures between PtZn/HNCNT and Pt/HNCNT, X-ray photoelectron spectroscopy (XPS) and X-ray absorption spectrometric (XAS) measurements were performed. Supplementary Fig. 15 shows the XPS analysis at Pt 4*f*. Compared with Pt/HNCNT, a shift to lower binding energy is observed for PtZn/HNCNT. The Zn 2*p* peak of PtZn/HNCNT is shifted to higher binding energy than metallic zinc (Supplementary Fig. 16)^45^. These phenomena indicate the Pt electronic states are modified by Zn in PtZn/HNCNT via electron transfer from Zn to Pt atoms. As seen from the normalized X-ray absorption near-edge structure (XANES) curves at the Pt L_3_-edge (Fig. 2a), PtZn/HNCNT has lower energy of the adsorption edge (E_0_) than Pt foil and Pt/HNCNT, suggesting the electron richness of Pt atoms in PtZn/HNCNT. In comparison with Zn foil, this material exhibits higher height of white line (*H*_w_) and E_0_ at the Zn K-edge (Fig. 2b). It demonstrates the electron deficiency at the Zn atoms in PtZn/HNCNT, in accordance with the result of Pt L_3_-edge XANES spectra. Furthermore, the corresponding Fourier transform of extended X-ray absorption fine structure (EXAFS) oscillations (Supplementary Figs. 17 and 18) are performed at Pt L_3_-edge and Zn K-edge. The first nearest-coordination peak of PtZn/HNCNT displayed a slight shift in R space than that of Pt foil at Pt L_3_-edge (Fig. 2c), while a slight shift in reverse direction was also observed at Zn K-edge (Fig. 2d), indicating the mild change of atomic distance. Pt–Pt bonds at around 2.4 Å were obviously observed in Pt/CN, while only a peak at 1.6 Å in the first shell was observed in Pt/HNCNT (Fig. 2c). EXAFS fitting were further carried out to obtain quantitative structural configuration of Pt in the three samples (Supplementary Fig. 19 and Table S1). The average coordination number of Pt/HNCNT is 3.8, indicating a Pt–N_4_ structure. The experimental curves of Pt/CN and PtZn/HNCNT could fit well with that of Pt metals and PtZn IMC respectively, furthering confirming that the Pt dispersed as nanoparticles and isolated-single-atomic-sites in Pt/CN and Pt/HNCNT, respectively.

CO adsorption was carried out to study the dispersion and electronic structures of Pt phase (Supplementary Fig. 20). There is a red shift of the CO stretching frequency for PtZn/HNCNT compared to Pt/CN (from 2083 to 2053 cm^−1^), indicating that the electron density is increased for PtZn/HNCNT due to the presence of zinc^46–51^, in consistent with the observation of Pt XPS. Pt/HNCNT demonstrates a peak at 2143 cm^−1^, further confirming that the Pt component in this sample is dispersed as isolated-single-atomic-sites^52^.

### Catalytic performance for selective hydrogenation

**Fig. 3:**
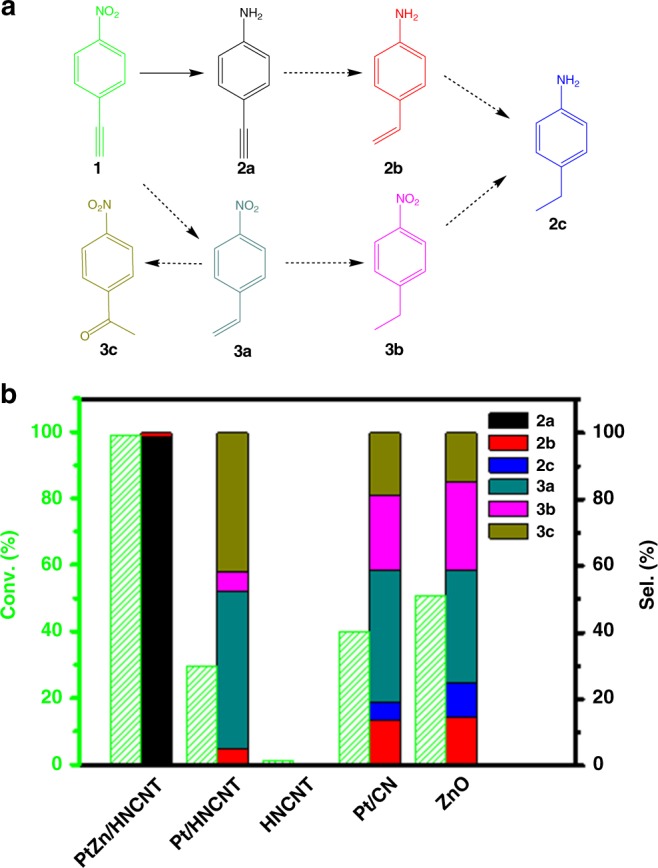
Catalytic performance of the catalysts. **a** Reaction pathway of the hydrogenation of 4-nitrophenylacetylene (4-NPA). **b** The catalytic performance of PtZn/HNCNT, Pt/HNCNT, hollow nitrogen-doped carbon nanotube (HNCNT), Pt/CN, and ZnO

Hydrogenation of 4-NPA (**1**) was then exploited as a model reaction to investigate the catalytic performance of PtZn/HNCNT. Since there are two easily reducible groups, nitro group and alkynyl group, the hydrogenation reaction has two possible pathways (Fig. 3a). To our delight, the PtZn/HNCNT exhibited a high hydrogenation selectivity to nitro functional group, producing 4-APA (**2a**). The conversion was 100% and the selectivity was 99% within 4 h using ammonia borane as the hydrogenation source (Fig. 3b). Trace amount of further hydrogenation product 4-aminostyrene (**2b**) was also detected. The 1H NMR and 13C NMR further confirmed the product is 4-aminophenylacetylene (Supplementary Figs. 21 and 22) and the mass recovery yield of **2b** is 93%. The kinetic profile was followed and shown in Supplementary Fig. 23. The conversion increased fast in the first 2 h while the selectivity kept 99 % to **2a**. Different reaction conditions were also studied and shown in Supplementary Table 2. The highest selectivity was obtained at 40 °C. Since the reaction was carried out in the pressure tube, a larger volume of tube was used to reduce the pressure during the reaction. However, a decreased selectivity was observed. In comparison, the Zn-free catalyst Pt/HNCNT was also applied into such reaction, but exhibited rather different performance as compared to PtZn/HNCNT. Nearly 30% conversion was observed after 4 h while a complex mixture containing 5% of **2b**, 47% of 4-nitrostyrene (**3a**), 6% of 4-nitroethylbenzene (**3b**), and 42% of 4-nitroacetophenone (**3c**) (generated from the oxidation of **3a** by the remaining air during the reaction process) was obtained. Nearly no reaction occurred in the absence of Pt (i.e., HNCNT, its characterizations are shown in Supplementary Figs. 24 and 25), proving that the active species are the Pt metals. Pt/CN, which is prepared without using ZnO and thus bearing Pt nanoparticles supported on CN support, was also compared. It exhibited similar selectivity to that of Pt/HNCNT, with 39.7% selectivity to **3a**. To compare the potential difference in selectivity of those catalysts, the selectivity was compared at similar conversion level (Supplementary Fig. 26). PtZn/HNCNT exhibited extremely high selectivity to **2a** compared to the Pt/HNCNT and Pt/CN samples. Obviously, PtZn/HNCNT catalyzed the hydrogenation of 4-NPA in different routes to Pt/HNCNT and Pt/CN. With isolating contiguous Pt and forming Pt–Zn bridge site, the PtZn/HNCNT can distinctively hydrogenate the nitro group in 4-NPA, achieving a high selectivity of **2a**. The catalytic performance of ZnO is also tested. It showed 51% conversion after 4 h, with 33.9% to **3a**. ZnO exhibits preferential hydrogenation to the alkynyl group. Since PtZn/HNCNT demonstrates preferential hydrogenation to the nitro group, it further excluded that zinc is the real active phase in the PtZn/HNCNT catalyst. In this study, PtZn/HNCNT can realize this process with a high selectivity in rather mild condition, which might promote the development of the selective hydrogenation of 4-NPA. Nitrobenzenes with electron donating and withdrawing groups were also used as the substrates (Supplementary Table 3). PtZn/HNCNT showed high selectivity for functionalized nitrobenzenes with electron donating or withdrawing groups in different positions. It indicated the outstanding performance of PtZn/HNCNT in selective hydrogenation of nitrobenzenes. Besides, the stability of the catalyst was tested. After reaction, there was nearly no change in the metal content (0.82% before and 0.80% after reaction), XRD, XPS, XAS, N_2_ sorption, and in situ Fourier-transform infrared spectroscopy of CO chemisorptions for PtZn/HNCNT (Supplementary Figs. 27–32). No obvious decrease in the catalytic performance was observed (Supplementary Fig. 33), suggesting the robust nature of the PtZn/HNCNT.

### DFT calculations of hydrogenation on the catalysts

To gain more insight into the high selectivity reason of PtZn/HNCNT, DFT calculations were utilized to distinguish the adsorption energies and hydrogenation energy barriers of 4-NPA on PtZn IMCs and Pt metals. The optimized geometry of 4-NPA@Pt(111) is shown in Supplementary Fig. 34. The distances between nitro group, aromatic ring, alkynyl group, and metal surface decrease gradually. The configuration which nitro group is most close to Pt surface was also considered (Supplementary Fig. 34), however, this configuration is 94 kJ mol^−1^ less stable. For PtZn $(02\bar 2)$ surface, the shortest distance between Pt atom in the same layer is 4.026 Å, which is significantly longer than it in Pt(111) surface (2.804 Å). Meanwhile the shortest distance between Pt and Zn in the same layer is 2.639 Å. In this sense, the Pt atom on the surface was isolated partly by two nearest-neighboring Zn atoms. The difference in electronegativity between Pt and Zn implies that the Pt atoms and Zn atoms in PtZn are negatively charged and positively charged, respectively, which is confirmed by the calculated charge population. As shown in Supplementary Fig. 35, maintaining more carbon atoms in both aromatic ring and alkynyl group close to Pt atoms and oxygen atoms in nitro close to Zn could make the configuration more stable. The adsorption configurations of 4-NPA at the Pt isolated-single-atomic-site (Pt–N–C) were also calculated and found that the one with the aromatic ring adsorbed on the Pt–N_4_ is more stable, but the difference between different modes was not big (Supplementary Fig. 36).

**Fig. 4:**
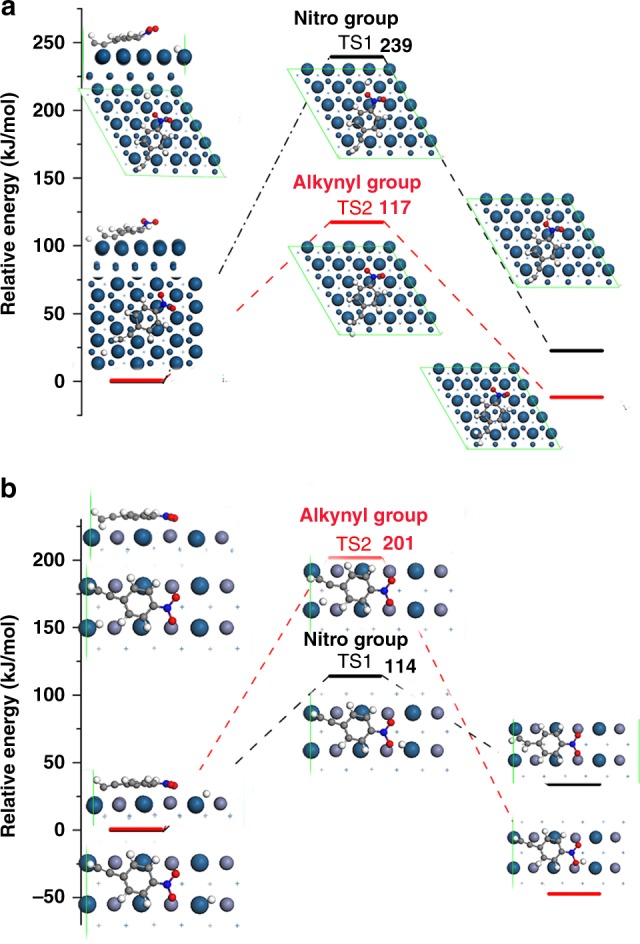
DFT calculations of 4-nitrophenylacetylene over the catalysts. **a**, **b** The first elementary hydrogenation reaction steps on both nitro and alkynyl groups over Pt(111) (**a**) and PtZn(022¯) (**b**) surface. Numbers labeled indicate the barriers of elementary steps (unit: kJ mol^−1^)

Hydrogenation processes could potentially occur on both nitro and alkynyl groups. To reveal the chemoselective hydrogenation process, the first elementary reaction steps on both groups were investigated. Hydrogen atom prefers to adsorb on the fcc-hollow site on Pt surface (Supplementary Fig. 37). Over Pt(111) surface, the energy barriers in the first step for hydrogenation on nitro group and alkynyl group are 239 and 117 kJ mol^−1^ (Fig. 4a), respectively, indicating that the hydrogenation process on nitro group is unfavorable kinetically. The thermochemistry-only potential energy pathways were constructed (Supplementary Figs. 38–47). In the first two hydrogenation steps, the alkynyl group is the favorable target, followed by the formation of vinyl group (intermediate **3a** in Fig. 3). The vinyl group does not achieve ethyl till the tenth hydrogenation step, meanwhile, the last hydrogenation step is endothermic. The long-lasting unsaturated C_2_H_4_-group should be the primitive intermediate for the formation of CH_3_-CO-C_6_H_4_-NO_2_ (**3c** in Fig. 3) and CH_3_-CH_2_-C_6_H_4_-NO_2_ (**3b** in Fig. 3). Over PtZn $(02\bar 2)$ surface, hydrogen atom prefers to adsorb on the Pt–Zn bridge site (Supplementary Fig. 48). The relative vertical distances between H* and its hydrogenation target atom (oxygen in nitro group or carbon atom in alkynyl group) are comparable (0.897 vs. 0.978 Å). For 4-NPA@PtZn $(02\bar 2)$, hydrogenation on nitro group is along the hydrogen atom diffusion path from Pt–Zn(bridge) to Zn–Pt(bridge) (along 〈011〉direction, Supplementary Fig. 49). The diffusion barrier is 29 kJ mol^−1^. Meanwhile, the hydrogenation on alkynyl group is along 〈100〉 direction. Hydrogen diffusion along this path is unfavorable (diffusion barrier is 143 kJ mol^−1^). Interestingly, the energy barriers in the first step for hydrogenation on nitro group and alkynyl group are 114 and 201 kJ mol^−1^ (Fig. 4b), respectively, indicating that the hydrogenation process on alkynyl group is unfavorable kinetically. Based on the direct hydrogenation path nitro group, the barriers of first hydrogenation step on alkynyl group for CH-C-C_6_H_4_-NOOH, CH-C-C_6_H_4_-NO, CH-C-C_6_H_4_-NHO, CH-C-C_6_H_4_-N, CH-C-C_6_H_4_-NH, and CH-C-C_6_H_4_-NH_2_ on IMC surface were also calculated (Supplementary Figs. 50–59). We can find that every reaction needs to climb over a barrier higher than 200 kJ mol^−1^, which is difficult to trigger the subsequent hydrogenation reactions on the alkynyl group. We considered that the such high reaction barrier on alkynyl part is the essential reason for the high selectivity on PtZn surface.

## Discussion

In conclusion, we have found that the selectivity of Pt catalysts can be effectively tuned by isolating Pt contiguous atoms and forming Pt–Zn intermetallic nanoparticles. Notably, the PtZn/HNCNT exhibits superior catalytic selectivity (99%) of the hydrogenation of 4-NPA to 4-APA in comparison to Pt/HNCNT. DFT calculations reveal that the existence of Zn atoms increases the adsorption of nitro groups, and Pt–Zn intermetallic nanoparticles promote the hydrogen atom diffusion path. This work provides a feasible strategy to tune the selectivity of NMs to achieve the desired products.

## Methods

### Synthesis of ZnO@PDA

ZnO nanorods were synthesized by a reported method^53^. Totally, 0.1 g ZnO nanorods were dispersed in 20 ml Tris buffer solution (10 mM, pH 8.5) by ultrasonication. Then 1 ml dopamine hydrochloride (100 mg) solution was added to the ZnO suspension while stirring. The suspension was allowed to stir for 12 h at room temperature. The resulting products were washed by deionized water and ethanol three times and collected by centrifugation. After dried at 80 °C in an oven, the desired ZnO@PDA was obtained.

### Synthesis of Pt(OH)_4_/ZnO@PDA

A total of 22.5 mg of urea was added in 100 ml of deionized water. Then, 200 mg of ZnO@PDA was added into the solution and ultrasonicated for 30 min. Totally, 10.5 μL of 0.1 g ml^−1^ H_2_PtCl_6_ aqueous solution was added and stirred for 3 h at room temperature. The mixture was then heated up to 90 °C for 12 h. The products were centrifuged and washed with deionized water three times. After drying in an oven at 80 °C overnight, Pt(OH)_4_/ZnO@PDA was obtained.

### Synthesis of PtZn/HNCNT

The obtained Pt(OH)_4_/ZnO@PDA was treated at 800 °C in a home-made porcelain boat with a cap under H_2_ (5% in Ar) atmosphere for 1 h in a tube furnace at a ramp rate of 5 °C min^−1^. After cooling down to room temperature naturally, the heat treated samples were washed in 5 M HCl for 12 h. Finally, the resulting product PtZn/HNCNT was obtained after the samples were centrifuged, washed with deionized water several times, and dried at 80 °C in an oven.

### Catalytic test for 4-nitrophenylacetylene hydrogenation

The selective hydrogenation of 4-nitrophenylacetylene (4-NPA) of the catalysts was tested in a 15 ml pressure bottle with 2 mg of the catalyst, 73.6 mg (0.5 mmol) of 4-NPA, 92.6 mg (3 mmol) of ammonia borane, 0.1 ml distilled water, and 4.9 ml ethanol. The reaction was performed at 40 °C for 4 h. Liquid samples was analyzed by gas chromatography with a Thermo Finnigan chromatograph equipped with a flame ionization detector and a DB-WAX capillary column (J&W, 30 m, 0.25 mm i.d.) with nitrogen as the carrier gas.

### Characterizations

Transmission electron microscopy (TEM) images were taken on a Hitachi HT7700 transmission electron microscope working at 100 kV. The high-resolution TEM, HAADF-STEM images, and the corresponding EDX mapping were recorded by a JEOL JEM-2100F high resolution TEM operating at 200 kV. Aberration-corrected HAADF-STEM images are taken on a JEOL JEM-ARM200F TEM/STEM with a spherical aberration corrector working at 300 kV. Powder X-ray diffraction patterns were measured with a Bruker D8 with Cu Kα radiation (*λ* = 1.5406 Å). The XPS was measured ex situ by a PHI Quantera SXM system under 3.1 × 10^−8^ Pa using Al^+^ radiation at room temperature. The binding energies were calibrated by referring C 1*s* peak to 284.8 eV. The metal content was determined by inductively coupled plasma optical emission spectroscopy (ICP-OES) on Thermo Fisher IRIS Intrepid II.

## Supplementary information

Supplementary Information

Peer Review

## Data Availability

The data that support the findings of this study are available from the corresponding author upon request.

## References

[1] Liu L, Corma A (2018). Metal catalysts for heterogeneous catalysis: from single atoms to nanoclusters and nanoparticles. Chem. Rev..

[2] Quan Z, Wang Y, Fang J (2013). High-index faceted noble metal nanocrystals. Acc. Chem. Res..

[3] Shoaib A (2016). Noble metal nanoclusters and their in situ calcination to nanocrystals: Precise control of their size and interface with TiO_2_ nanosheets and their versatile catalysis applications. Nano Res..

[4] Liu J, Krajangsri S, Yang J, Li J-Q, Andersson PG (2018). Iridium-catalysed asymmetric hydrogenation of allylic alcohols via dynamic kinetic resolution. Nat. Catal..

[5] Mao J (2017). Rational control of the selectivity of a ruthenium catalyst for hydrogenation of 4-nitrostyrene by strain regulation. Angew. Chem..

[6] Feng Q (2017). Isolated single-atom pd sites in intermetallic nanostructures: high catalytic selectivity for semihydrogenation of alkynes. J. Am. Chem. Soc..

[7] Wei H (2014). FeOx-supported platinum single-atom and pseudo-single-atom catalysts for chemoselective hydrogenation of functionalized nitroarenes. Nat. Commun..

[8] Liu L (2016). Generation of subnanometric platinum with high stability during transformation of a 2D zeolite into 3D. Nat. Mater..

[9] Imaoka T (2017). Platinum clusters with precise numbers of atoms for preparative-scale catalysis. Nat. Commun..

[10] Lucci FR (2015). Selective hydrogenation of 1,3-butadiene on platinum–copper alloys at the single-atom limit. Nat. Commun..

[11] Vilé G, Almora-Barrios N, López N, Pérez-Ramírez J (2015). Structure and reactivity of supported hybrid platinum nanoparticles for the flow hydrogenation of functionalized nitroaromatics. ACS Catal..

[12] Iwasa N, Mayanagi T, Ogawa N, Sakata K, Takezawa N (1998). New catalytic functions of Pd–Zn, Pd–Ga, Pd–In, Pt–Zn, Pt–Ga and Pt–In alloys in the conversions of methanol. Catal. Lett..

[13] Cuenya BR, Behafarid F (2015). Nanocatalysis: size- and shape-dependent chemisorption and catalytic reactivity. Surf. Sci. Rep..

[14] Moliner MGabay (2016). Reversible transformation of Pt nanoparticles into single atoms inside high-silica chabazite zeolite. J. Am. Chem. Soc..

[15] Vilé G, Richard-Bildstein S, Lhuillery A, Rueedi G (2018). Electrophile, substrate functionality, and catalyst effects in the synthesis of α-mono and di-substituted benzylamines via visible‐light photoredox catalysis in flow. ChemCatChem.

[16] Leonard KA (2000). A selenopyrylium photosensitizer for photodynamic therapy related in structure to the antitumor agent aa1 with potent in vivo activity and no long-term skin photosensitization. J. Med. Chem..

[17] Xu H, Li S, Huang W, Guo Q, Gao Y (2008). Synthesis and antitumor activities of erlotinib derivatives. J. Chin. Pharm. Univ..

[18] Corma A, Serna P, Concepción P, Calvino JJ (2008). Transforming nonselective into chemoselective metal catalysts for the hydrogenation of substituted nitroaromatics. J. Am. Chem. Soc..

[19] Nørskov JK, Bligaard T, Rossmeisl J, Christensen CH (2009). Towards the computational design of solid catalysts. Nat. Chem..

[20] Bell AT (2003). The impact of nanoscience on heterogeneous catalysis. Science.

[21] Chen G (2016). Interfacial electronic effects control the reaction selectivity of platinum catalysts. Nat. Mater..

[22] Mistry H, Varela AS, Kühl S, Strasser P, Cuenya BR (2016). Nanostructured electrocatalysts with tunable activity and selectivity. Nat. Rev. Mater..

[23] Yan H (2015). Single-atom Pd1/graphene catalyst achieved by atomic layer deposition: remarkable performance in selective hydrogenation of 1,3-butadiene. J. Am. Chem. Soc..

[24] Schoenbaum CA, Schwartz DK, Medlin JW (2014). Controlling the surface environment of heterogeneous catalysts using self-assembled monolayers. Acc. Chem. Res..

[25] Liu P, Qin R, Fu G, Zheng N (2017). Surface coordination chemistry of metal nanomaterials. J. Am. Chem. Soc..

[26] Zhang S (2018). Strong electronic metal-support interaction of Pt/CeO_2_ enables efficient and selective hydrogenation of quinolines at room temperature. J. Catal..

[27] Kattel S, Ramírez PJ, Chen JG, Rodriguez JA, Liu P (2017). Active sites for CO_2_ hydrogenation to methanol on Cu/ZnO catalysts. Science.

[28] Xu B, Yang H, Zhou G, Wang X (2014). Strong metal-support interaction in size-controlled monodisperse palladium-hematite nano-heterostructures during a liquid-solid heterogeneous catalysis. Sci. China Mater..

[29] Matsubu JC (2016). Adsorbate-mediated strong metal–support interactions in oxide-supported Rh catalysts. Nat. Chem..

[30] Li Z (2018). Reactive metal–support interactions at moderate temperature in two-dimensional niobium-carbide-supported platinum catalysts. Nat. Catal..

[31] Sibin D, Rongming W, Jingyue L (2018). Stability investigation of a high number density Pt_1_/Fe_2_O_3_ single-atom catalyst under different gas environments by HAADF-STEM. Nanotechnology.

[32] Zhang P (2015). One-pot synthesis of ternary Pt–Ni–Cu nanocrystals with high catalytic performance. Chem. Mater..

[33] Armbrüster M (2012). Al13Fe4 as a low-cost alternative for palladium in heterogeneous hydrogenation. Nat. Mater..

[34] Wu Y (2013). Defect-dominated shape recovery of nanocrystals: a new strategy for trimetallic catalysts. J. Am. Chem. Soc..

[35] Fu Q-Q, Li H-H, Ma S-Y, Hu B-C, Yu S-H (2016). A mixed-solvent route to unique PtAuCu ternary nanotubes templated from Cu nanowires as efficient dual electrocatalysts. Sci. China Mater..

[36] Fan H, Cheng M, Wang Z, Wang R (2017). Layer-controlled Pt-Ni porous nanobowls with enhanced electrocatalytic performance. Nano Res..

[37] Zhao EW (2017). Silica-encapsulated Pt-Sn intermetallic nanoparticles: a robust catalytic platform for parahydrogen-induced polarization of gases and liquids. Angew. Chem..

[38] Qi Z (2017). Sub-4 nm PtZn intermetallic nanoparticles for enhanced mass and specific activities in catalytic electrooxidation reaction. J. Am. Chem. Soc..

[39] Armbrüster M (2010). Pd-Ga intermetallic compounds as highly selective semihydrogenation catalysts. J. Am. Chem. Soc..

[40] Shao L (2011). Nanosizing intermetallic compounds onto carbon nanotubes: active and selective hydrogenation catalysts. Angew. Chem. Int. Ed..

[41] Friedrich M, Teschner D, Knop-Gericke A, Armbrüster M (2012). Surface and subsurface dynamics of the intermetallic compound ZnNi in methanol steam reforming. J. Phys. Chem. C.

[42] Kang Y, Pyo JB, Ye X, Gordon TR, Murray CB (2012). Synthesis, shape control, and methanol electro-oxidation properties of Pt-Zn alloy and Pt_3_Zn intermetallic nanocrystals. ACS Nano.

[43] Jana S, Chang JW, Rioux RM (2013). Synthesis and modeling of hollow intermetallic Ni–Zn nanoparticles formed by the Kirkendall effect. Nano Lett..

[44] Furukawa S, Yoshida Y, Komatsu T (2014). Chemoselective hydrogenation of nitrostyrene to aminostyrene over Pd- and Rh-based intermetallic compounds. ACS Catal..

[45] Fouad OA, Ismail AA, Zaki ZI, Mohamed RM (2006). Zinc oxide thin films prepared by thermal evaporation deposition and its photocatalytic activity. Appl. Catal. B.

[46] Camacho-Bunquin J (2018). Atomically precise strategy to a PtZn alloy nanocluster catalyst for the deep dehydrogenation of n‑butane to 1,3-butadiene. ACS Catal..

[47] Boccuzzi F, Chiorino A, Guglielminotti E (1998). Effects of structural defects and alloying on the FTIR spectra of CO adsorbed on Pt/ZnO. Surf. Sci..

[48] Brandt RK, Hughes MR, Bourget LP, Truszkowska K, Greenler RG (1993). The interpretation of CO adsorbed on Pt/SiO_2_ of two different particle-size distributions. Surf. Sci..

[49] Brandt RK, Sorbello RS, Greenler RG (1992). Site-specific, coupled-harmonic-oscillator model of carbon monoxide adsorbed on extended, single-crystal surfaces and on small crystals of platinum. Surf. Sci..

[50] Yoshida H, Narisawa S, Fujita SI, Ruixia L, Arai M (2012). In situ FTIR study on the formation and adsorption of CO on alumina-supported noble metal catalysts from H_2_ and CO_2_ in the presence of water vapor at high pressuresw. Phys. Chem. Chem. Phys..

[51] Yoshida H, Igarashi N, Fujita SI, Panpranot J, Arai M (2015). Influence of crystallite size of TiO_2_ supports on the activity of dispersed Pt catalysts in liquid-phase selective hydrogenation of 3-nitrostyrene, nitrobenzene, and styrene. Catal. Lett..

[52] Ding K (2015). Identification of active sites in CO oxidation and water-gas shift over supported Pt catalysts. Science.

[53] Cheng B, Samulski ET (2004). Hydrothermal synthesis of one-dimensional ZnO nanostructures with different aspect ratios. Chem. Commun..

