# Supplementary material for: Isolating contiguous Pt atoms and forming Pt-Zn intermetallic nanoparticles to regulate selectivity in 4-nitrophenylacetylene hydrogenation
Source: Nat Commun. 2019 Aug 22;10:3787. doi: 10.1038/s41467-019-11794-6 (PMC6706404; doi:10.1038/s41467-019-11794-6)
Supplement: Supplementary file 1 — Supplementary Information [file 41467_2019_11794_MOESM1_ESM.pdf]

Isolating contiguous Pt atoms and forming Pt-Zn intermetallic nanoparticles to regulate selectivity in 4-nitrophenylacetylene hydrogenation

Supplementary Information

Han et al.

Contents

Supplementary Notes…..**2**

Supplementary Figures and Tables..**7**

Supplementary References..**39**

Supplementary Notes

Chemicals.

All chemicals were of analytical grade and used as received without further purification. Dopamine hydrochloride was purchased from Beijing HWRK Chem Co. LTD. Tris(hydroxymethyl) aminomethane was purchased from Alfa Aesar (China) Chemicals Co., Ltd. Urea was purchased from Beijing Tongguang Fine Chemicals Company. H_2PtCl_6 and ammonia borane was purchased from Sigma-aldrich company. 4-nitrophenylacetylene was purchased from damas-beta company.

Synthesis of Pt/HNCNT.

The Pt/HNCNT was obtained by a similar procedure to PtZn/HNCNT except that the $Pt(OH)_4$/ZnO@PDA was treated at 200 ^oC in an H_2 (5% in Ar) atmosphere for 1 h in a tube furnace at a ramp rate of 2 ^oC min $^{-1}$.

Synthesis of Pt/CN.

The Pt/CN was obtained by a similar procedure to PtZn/HNCNT except that the $Pt(OH)_4$/PDA used polydopamine nanospheres instead of ZnO@PDA and Pt/CN was

without acid washing process. The polydopamine nanospheres were prepared according to literature[1]. The average diameter is around 200 nm.

Synthesis of PdZn/HNCNT.

The PdZn/HNCNT was obtained by a similar procedure to PtZn/HNCNT except that the Na_2PdCl_4 instead of H_2PtCl_6.

Characterization of products

Product **2a** was isolated by flash column chromatography on silica gel with petroleum ether/ethyl acetate as eluent. As a known compound, the product **2a** is characterized by comparison of their ^{1}H NMR and ^{13}C NMR spectroscopic data with those reported in the literature. All chemical shifts (δ) are reported in ppm and coupling constants (*J*) in Hz. All chemical shifts were reported relative to tetramethylsilane (0 ppm for ^{1}H), and $CDCl_3$ (77.16 ppm for ^{13}C), respectively. CO FTIR characterizations were carried out on a Brucker Tenser II *in situ* infrared spectrometer with MCT detector using a home-made cell. The samples were pretreated with H_2/Ar at 200 ^{o}C for 1 h. After cooling down to room temperature (20 ^{o}C) and flushing with Ar for 20 min, the background spectrum was collected. Then, CO gas was introduced into the sample holder for 30 min. The sample was flushing with Ar and spectra were collected every 15 s.

Recycle of the catalyst

The catalyst was centrifuged out and washed with ethanol for several times. After drying in oven at 80 °C, it was reused directly.

XAFS measurements: The X-ray absorption find structure spectra (Pt L_3-edge and Zn K-edge) were collected at BL1W1B station in Beijing Synchrotron Radiation Facility (BSRF). The data were collected in fluorescence excitation mode using a Lytle detector. All samples were pelletized as disks of 13 mm diameter using graphite powder as a binder.

XAFS Analysis and Results: The acquired EXAFS data were processed according to the standard procedures using the ATHENA module implemented in the IFEFFIT software packages. The EXAFS spectra were gained by subtracting the post-edge background from the overall absorption and then normalizing with respect to the edge-jump step. Subsequently, the $\chi(k)$ data were Fourier transformed to real (R) space using a hanning windows (dk=1.0 $Å^{-1}$) to separate the EXAFS contributions from different coordination shells. Least-squares curve parameter fitting was carried out using the ARTEMIS module of IFEFFIT software packages to obtain the quantitative structural parameters around central atoms.

Computational details.

The calculations were performed ultilizing the plane-wave pseudopotential method in the framework of density functional theory (DFT). The ion core and valence electron interaction was described by Vanderbilt-type ultrasoft

pseudopotential. The electron exchange-correlation effects were described by the generalized gradient approximation (GGA) in the form of Perdew-Burke-Ernzerh (PBE) functional[2]. All spin-polarized density functional theory (DFT) calculations were performed on the Cambridge serial total energy package (CASTEP) program[3]. The role of the van der Waals (vdw) force should not be ignored in the considered systems. Hence, the dispersion correction included DFT-D method was used[4]. The plane-wave cutoff enrgy was set to 350 eV. The convergence thresholds between optimization cycles for energy change and maximum force were set as 10^{-5} eV/atom and 0.03 eV/Å, respectively.

Surfaces were modeled using *p*(5×5) and *p*(3×4) unit cells for Pt(111) and PtZn($02\bar{2}$), respectively.

The Pt(111) surface was constructed by four Pt layers, which contains 75 Pt atoms. The PtZn($02\bar{2}$) surface was constructed by five layers, which contains 60 Pt atoms and 60 Zn atoms. A vacuum region of 12 Å was created along the surface to avoid interactions between the slabs. For both slab models, the coordinates of atoms in top two layers were relaxed fully during the geometry optimization. Brillouin zone integration was sampled with 1×1×1.

The transition states were searched by the generalized synchronous transit (LST/QST) method[5]. The algorithm performs a linear synchronous transit (LST) optimization, followed by repeated conjugate gradient minimizations and quadratic synchronous transit (QST) maximizations until a transition state has been located.

Using the above calculation method, the bulk PtZn was firstly optimized, obtaining the following lattice constants: a=b=4.026 Å, c=3.450 Å. The calculation results are well consistent with the XRD measurements: a=b=4.025 Å, c=3.491 Å. These calculation results indicate that the calculation models and method in the present work are reasonable.

Supplementary figures and tables

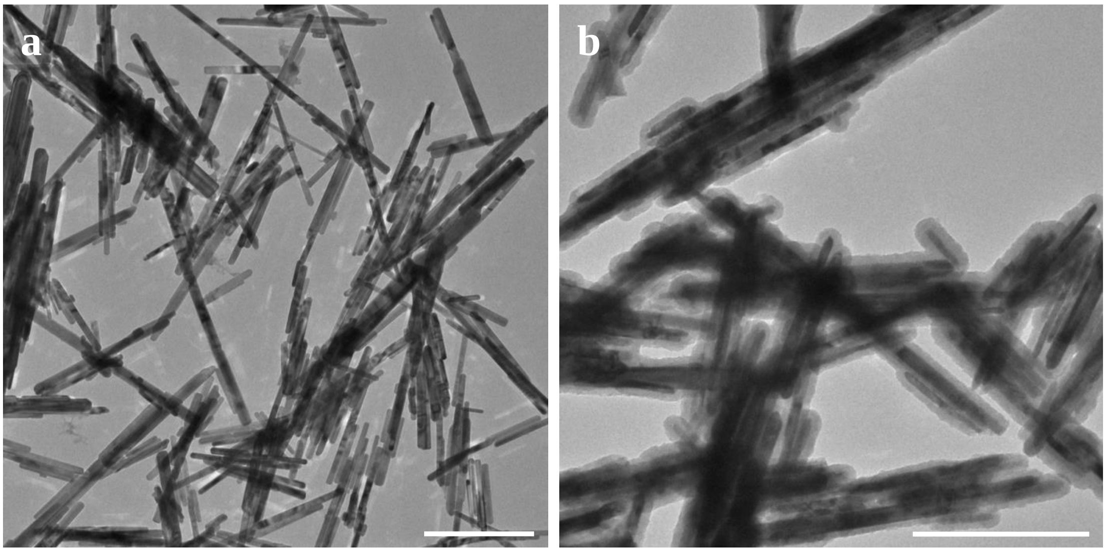

Supplementary Figure 1│TEM images of ZnO and ZnO@PDA. a ZnO; **b** ZnO@PDA. PDA is coated on the outsurface of ZnO nanorods uniformly. Scale bar, 500 nm.

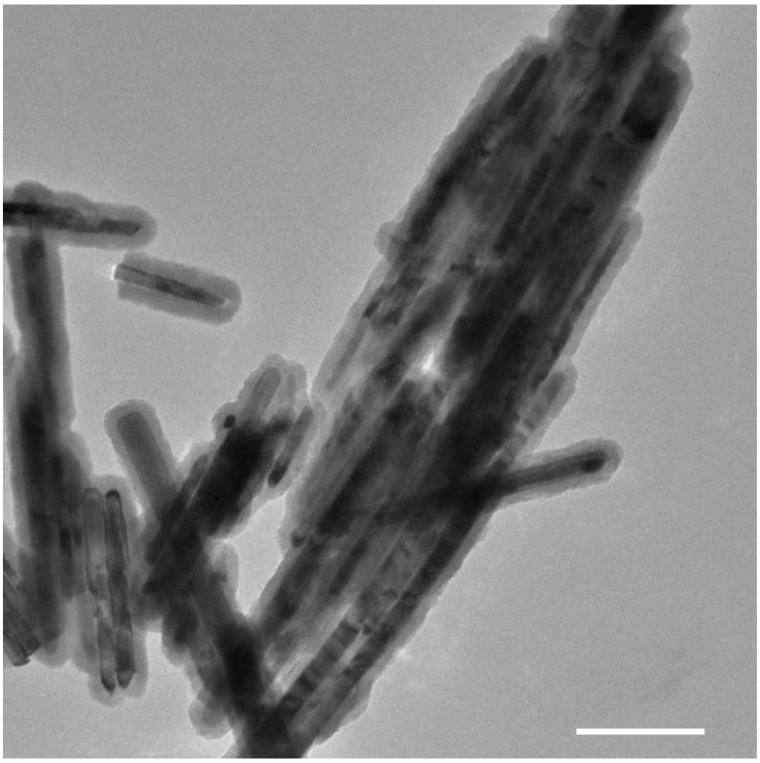

Supplementary Figure 2│ TEM image of $Pt(OH)_4$/ZnO@PDA. No obvious nanoparticles could be observed on the PDA layer. Scale bar, 500 nm.

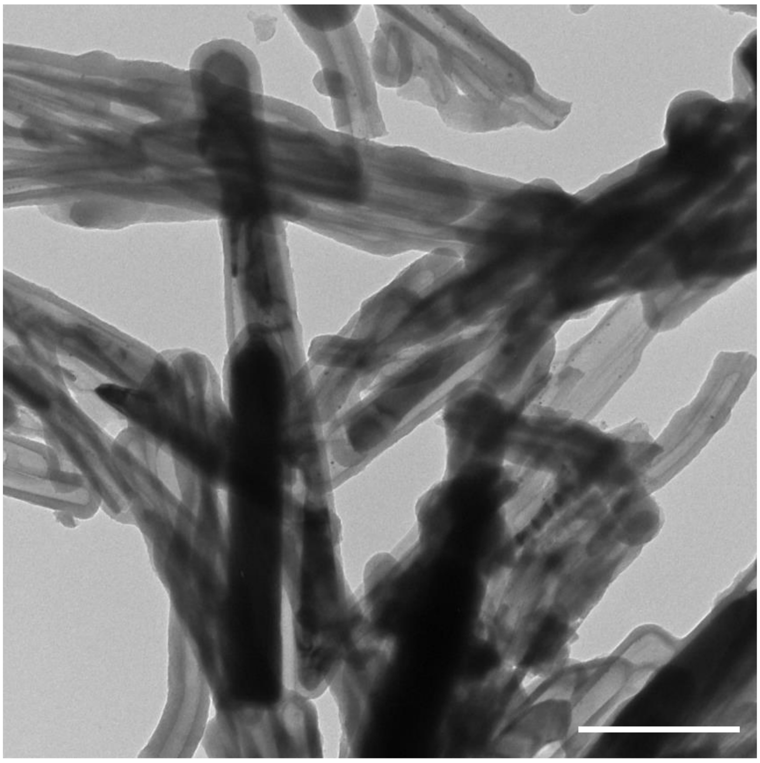

Supplementary Figure 3 | TEM image of PtZn/ZnO@HNCNT. ZnO nanorods are partically consumed. Scale bar, 500 nm.

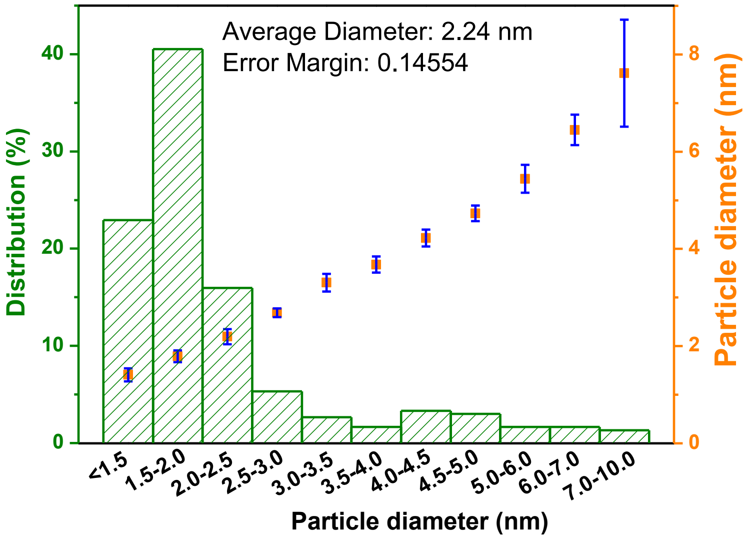

Supplementary Figure 4 | PtZn particle size distribution in PtZn/HNCNT. The distribution is determined by AC HAADF STEM images.

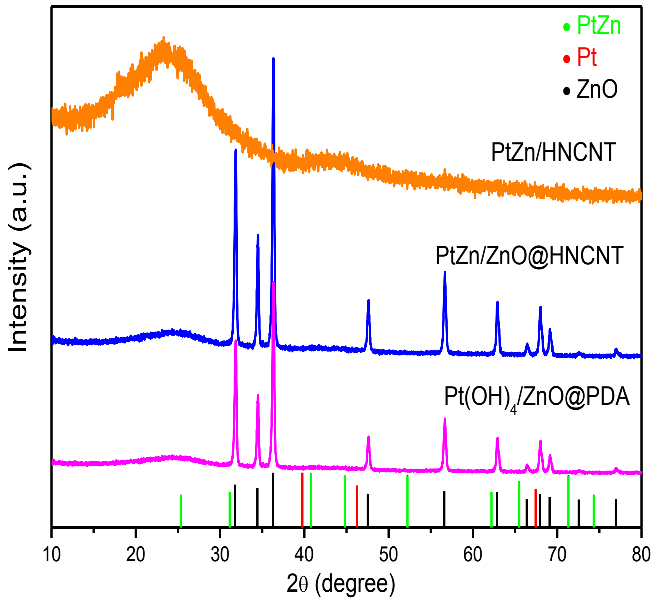

Supplementary Figure 5│X-ray diffraction patterns of $Pt(OH)_4$/ZnO@PDA, PtZn/ZnO@HNCNT, and PtZn/HNCNT. The diffraction peaks for $Pt(OH)_4$/ZnO@PDA and PtZn/ZnO@HNCNT could match well with ZnO. No obvious peaks of Pt or PtZn could be observed in the PtZn/HNCNT diffraction patterns due to the small size the the PtZn intermetallic nanoparticles.

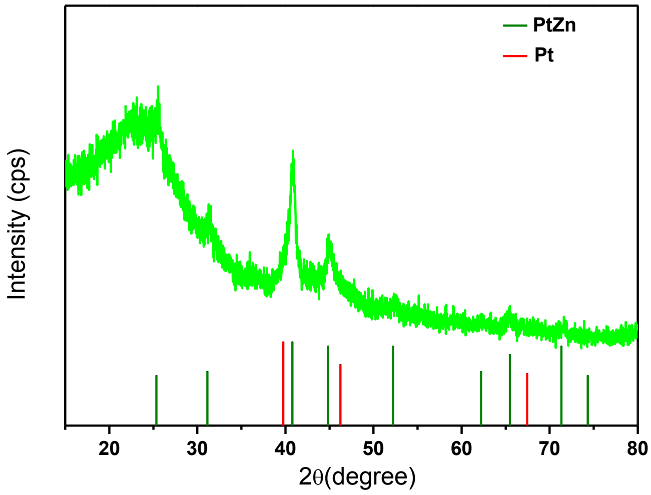

Supplementary Figure 6│XRD of PtZn/HNCNT prepared from 1% $Pt(OH)_4$/ZnO@PDA. The XRD patterns of this PtZn/HNCNT can be well identified to the diffraction peaks of PtZn intermetallic compound.

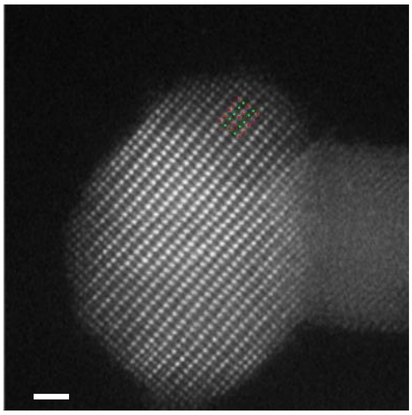

Supplementary Figure 7 | AC HAADF STEM image of a PtZn nanoparticle. The AC HAADF STEM image could match well with the (010) plane of PtZn (Pt, red; Zn, green). Scale bar, 1 nm.

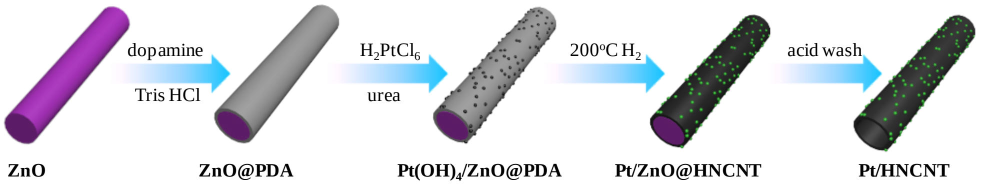

Supplementary Figure 8 | Scheme of the synthesis of Pt/HNCNT. $Pt(OH)_4$/ZnO@PDA is reduced at 200 ºC to avoid the evaporation of Zn.

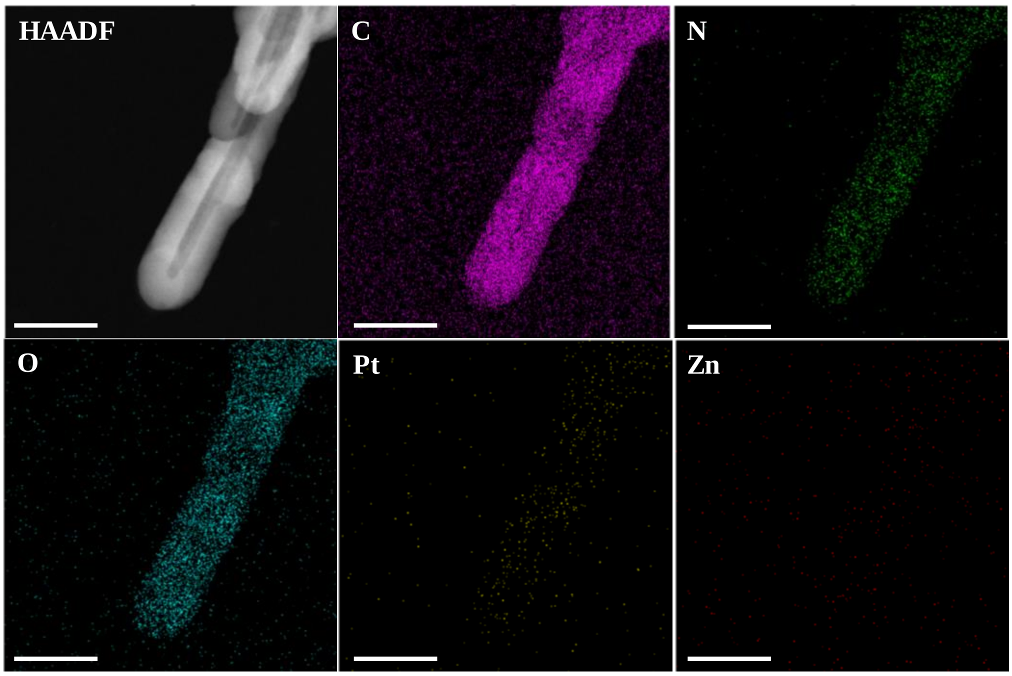

Supplementary Figure 9 | Dark field STEM and corresponding elemental mapping of Pt/HNCNT. The EDX elemental mapping images display that the distribution of C (purple), N (green), O (blue) and Pt (yellow) are dispersed uniformly in the shell of Pt/HNCNT. No signal of Zn could be detected. Scale bar, 250 nm.

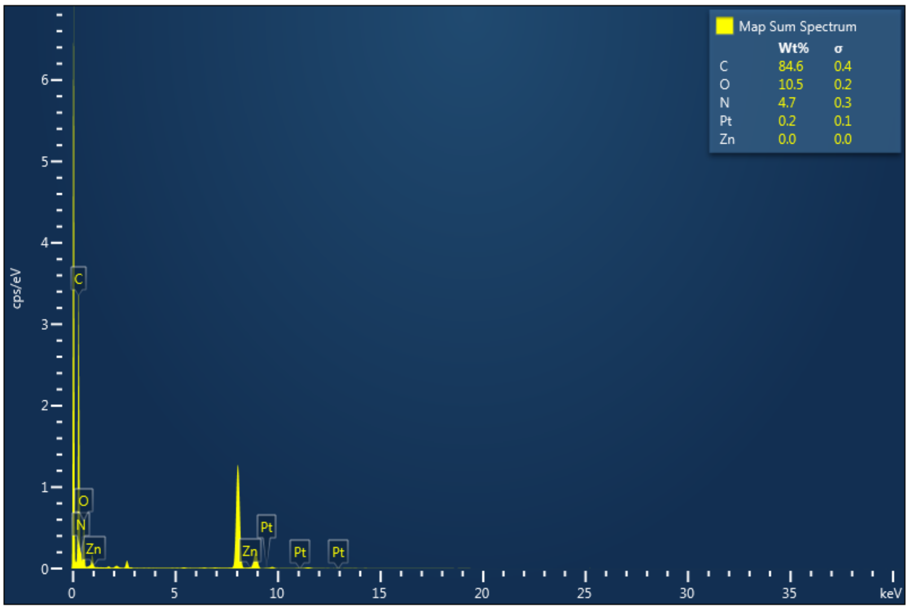

Supplementary Figure 10 | Energy disperse spectra of Pt/HNCNT. The EDS shows that the loading of Zn was too low to be detected.

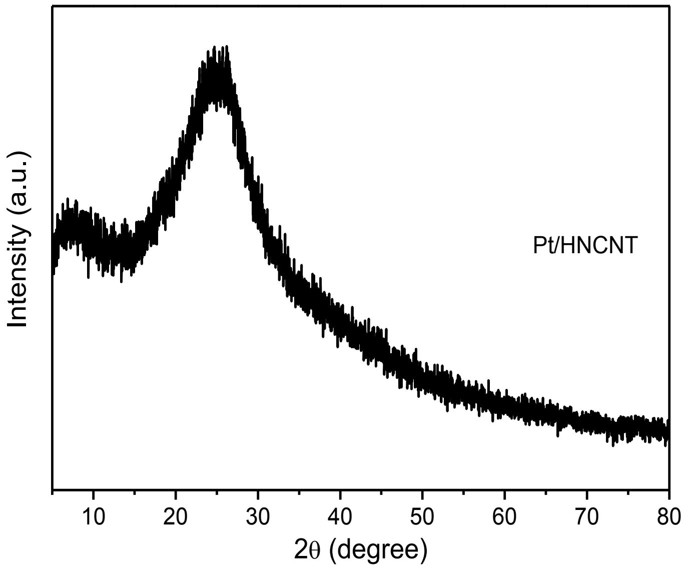

Supplementary Figure 11│X-ray diffraction patterns of Pt/HNCNT. The XRD of Pt/HNCNT demonstrates that no signal for metallic Pt could be detected.

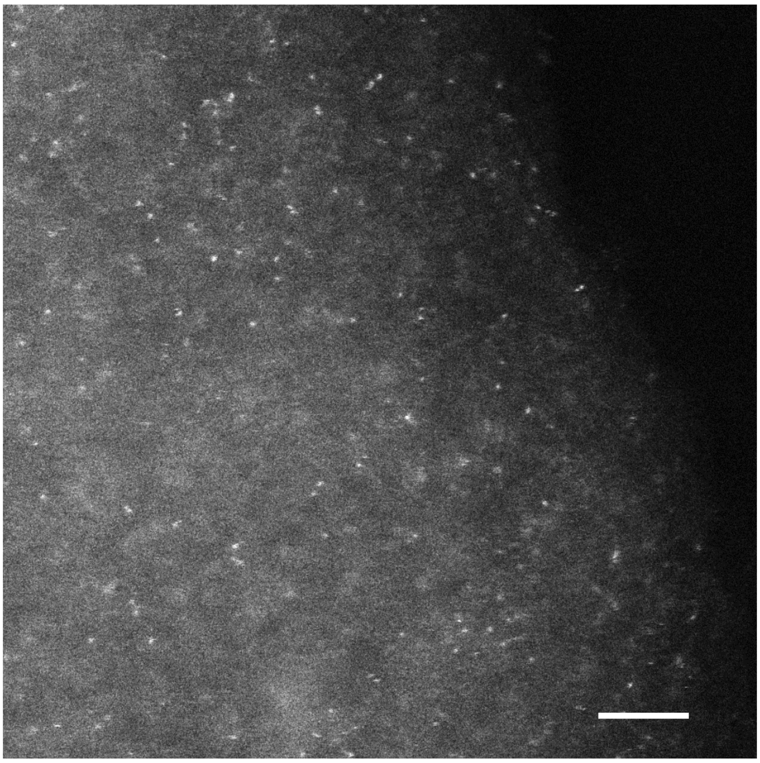

Supplementary Figure 12│AC HAADF STEM image Pt/HNCNT. Only discrete bright dots are observed. Scale bar, 2 nm.

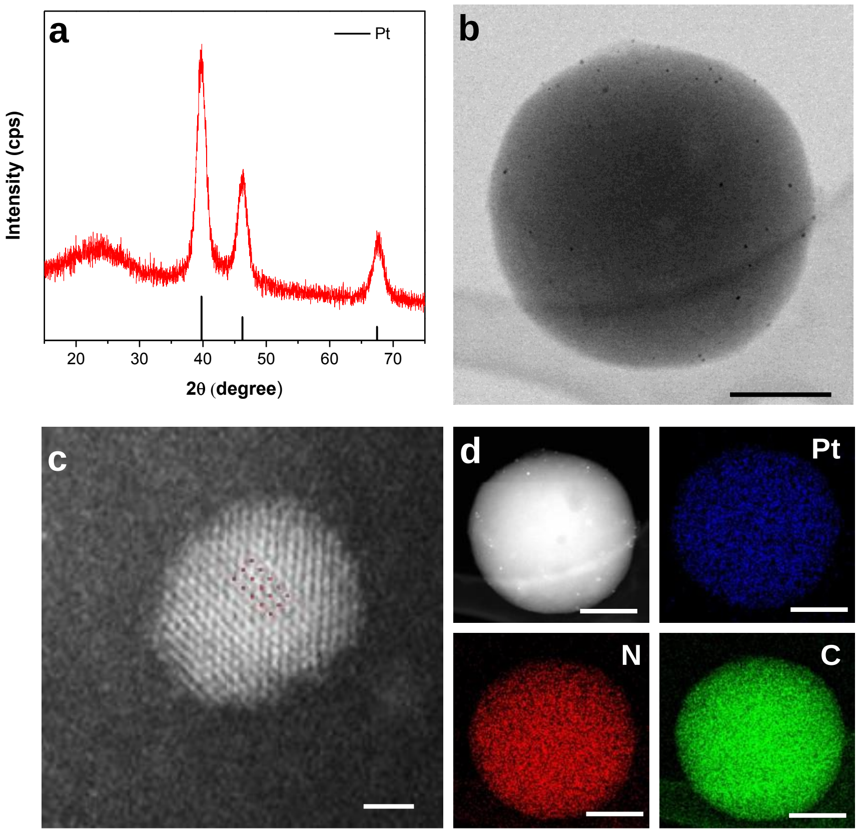

Supplementary Figure 13 | Characterizations of the Pt/CN catalyst. a XRD of Pt/CN. The peaks match well with metallic Pt. **b** TEM of Pt/CN. Obvious nanoparticles could be observed on the CN nanospheres. Scale bar, 100 nm. **c** AC HAADF STEM image of a Pt nanoparticle. The atomic arrangement could match well with the lattice of Pt. Scale bar, 1 nm. **d** Elemental mappings of Pt/CN. The Pt (blue), N (red) and C (green) distribute uniformly on the nanosphere. Scale bar, 100 nm.

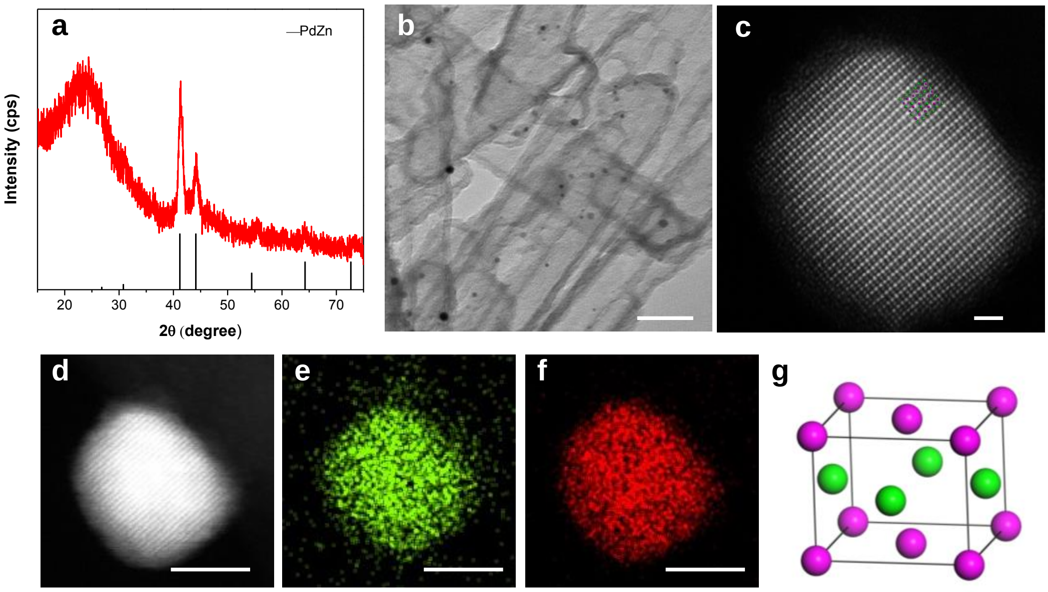

Supplementary Figure 14│Characterizations of the PdZn/HNCNT. a XRD of PdZn/HNCNT. The XRD patterns can match well with PdZn intermetallic compound. **b** TEM image of PdZn/HNCNT. Obvious nanoparticles with size of 2-10 nm are presented on the HNCNT shell. Scale bar, 50 nm. **c** AC HAADF STEM image of a PdZn nanoparticle. The atomic arrangement could match well with the PdZn intermetllic compound. Pd atoms (pink) and Zn atoms (green). Scale bar, 1 nm. **d-f** elemental mappings of PdZn/HNCNT. Pd (red) and Zn (green) distribute uniformly on the nanoparticle. Scale bar, 5 nm. **g** Crystal structure of PdZn intermetallic compound.

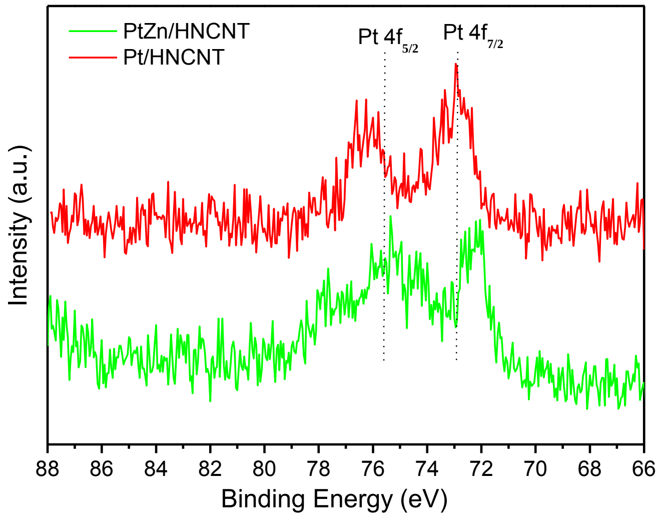

Supplementary Figure 15│Pt 4*f* XPS of PtZn/HNCNT and Pt/HNCNT. There is a shift to lower binding energy for PtZn/HNCNT compared to Pt/HNCNT.

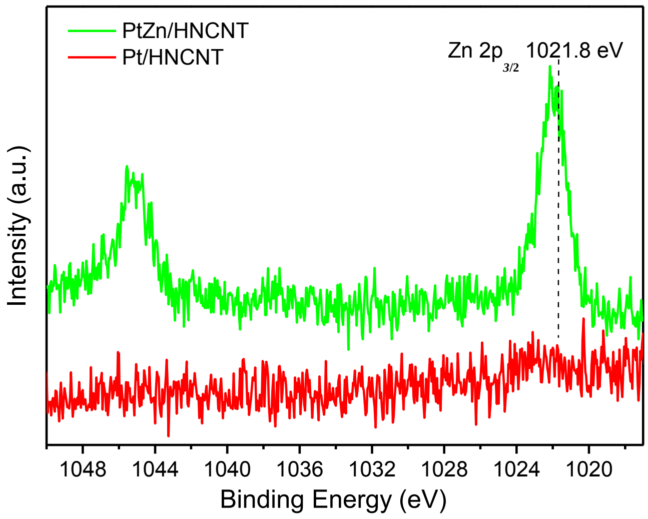

Supplementary Figure 16│Zn 2*p* XPS of PtZn/HNCNT and Pt/HNCNT. No Zn peak shows in the Pt/HNCNT.

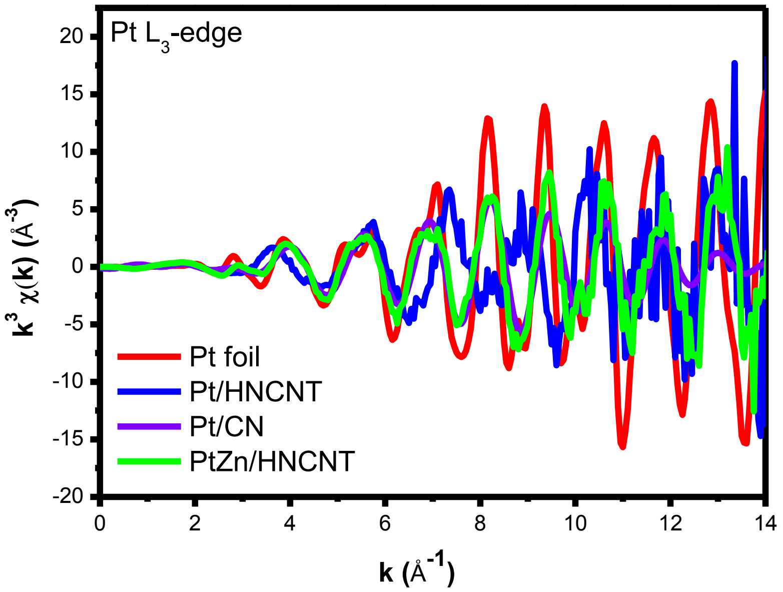

Supplementary Figure 17│The EXAFS oscillation functions at the Pt L$_3$-edge. Pt foil, red; Pt/HNCNT, blue; Pt/CN, purple; PtZn/HNCNT, green.

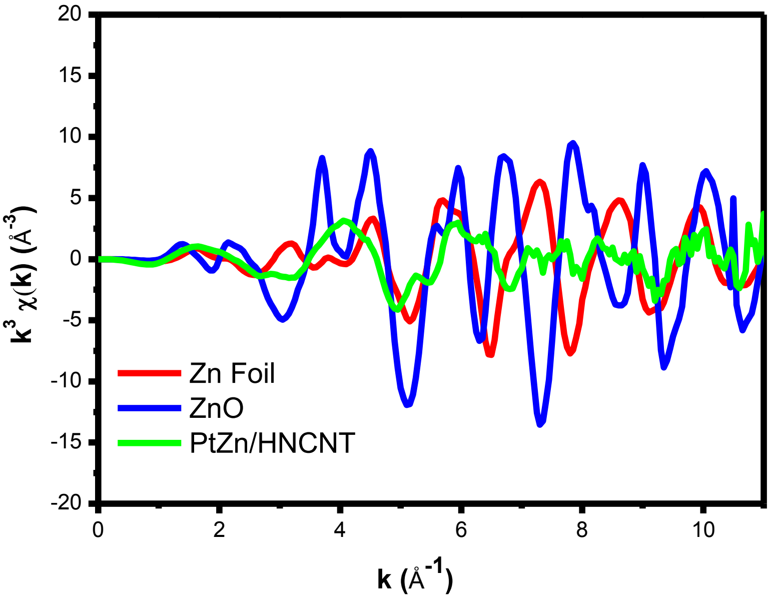

Supplementary Figure 18│The EXAFS oscillation functions at the Zn K-edge. Zn foil, red; ZnO, blue; PtZn/HNCNT, green.

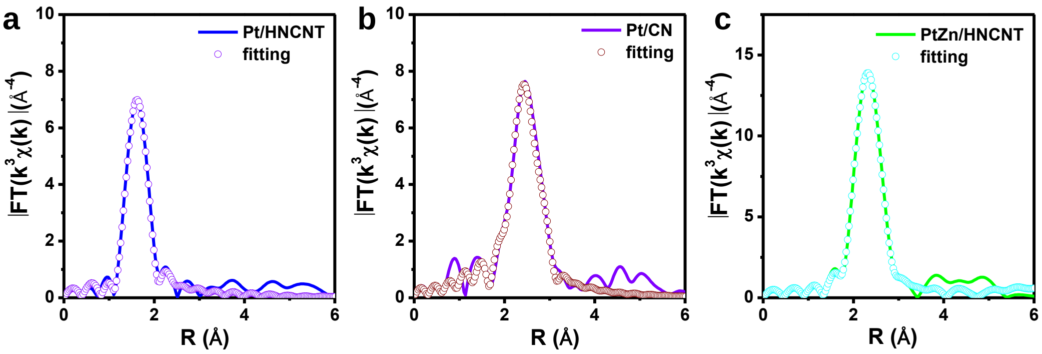

Supplementary Figure 19 │ The fitting results of the EXAFS spectra. **a** Pt/HNZNT, **b** Pt/CN, and **c** PtZn/HNCNT.

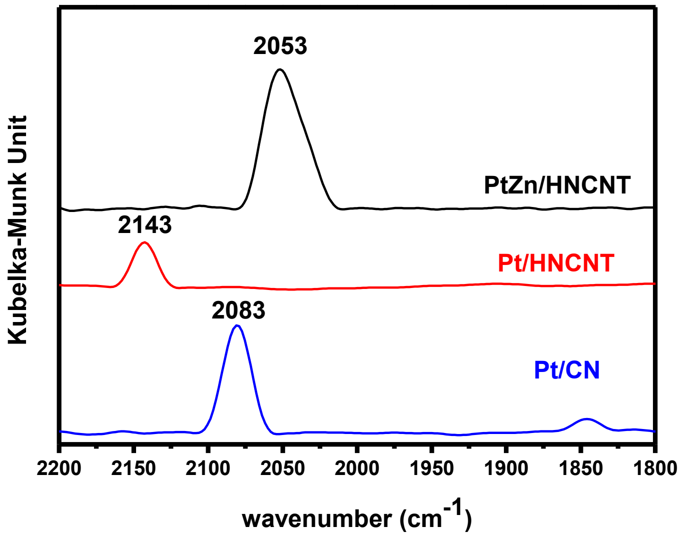

Supplementary Figure 20 │ *In situ* FTIR spectra of CO chemisorption for PtZn/HNCNT, Pt/HNCNT, and Pt/CN. There is a red shift of the CO stretching frequency for PtZn/HNCNT compared to Pt/CN. The CO stretching peak is located at 2143 cm^{-1} for Pt/HNCNT.

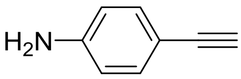

4-Ethynylaniline (2a)[1]. ^{1}H NMR (400 MHz, $CDCl_3$): δ 7.28 (d, *J* = 8.4 Hz, 2H), 6.56 (d, *J* = 8.4 Hz, 2H), 3.74 (s, 2H), 2.96 (s, 1H); ^{13}C NMR (101 MHz, $CDCl_3$): δ 147.1, 133.5, 114.7, 111.3, 84.5, 75.0.

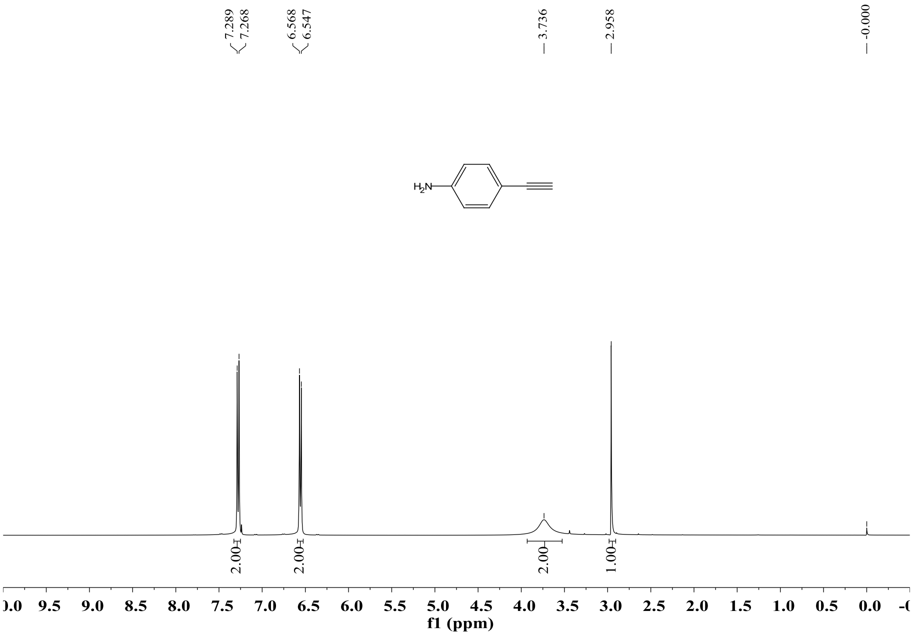

Supplementary Figure 21│^{1}H NMR (400 MHz, $CDCl_3$) spectrum of 4-ethynylaniline (2a). There are four types of H. The ^{1}H NMR confirms that the chemical structure of the product is 4-ethynylaniline.

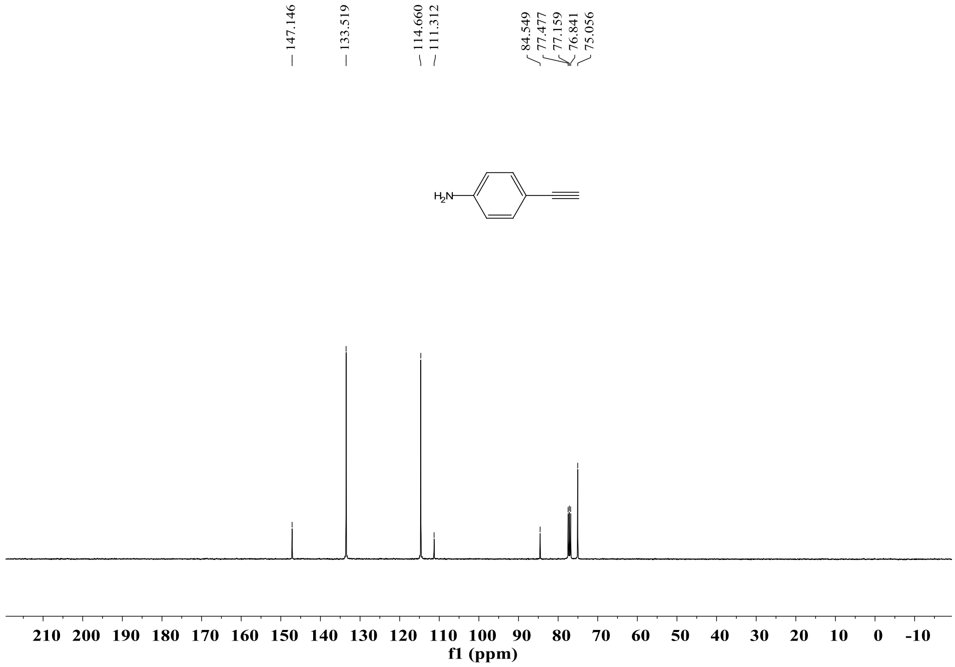

Supplementary Figure 22 | ^{13}C NMR (101 MHz, $CDCl_3$) spectrum of 4-ethynylaniline (2a). The ^{13}C NMR mathces well with that of 4-ethynylaniline.

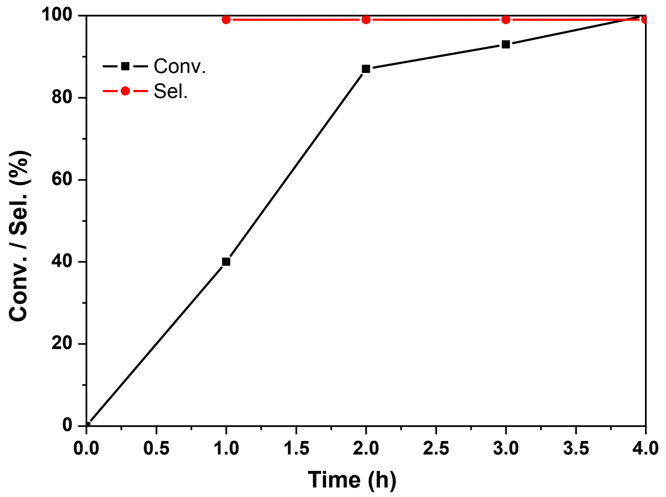

Supplementary Figure 23 | Kinetic profile of 4-nitrophenylacetylene hydrogenation over PtZn/HNCNT. The conversion increases with the reaction time while the selectivity is nearly the same.

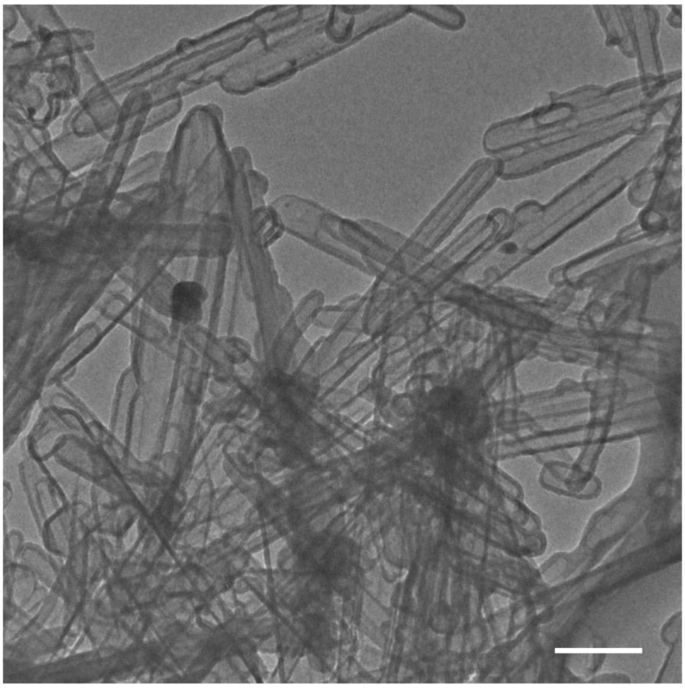

Supplementary Figure 24│TEM image of HNCNT. HNCNT is consisted of nitrogen-doped carbon nanotube with a thickness of 10-30 nm. Scale bar, 200 nm.

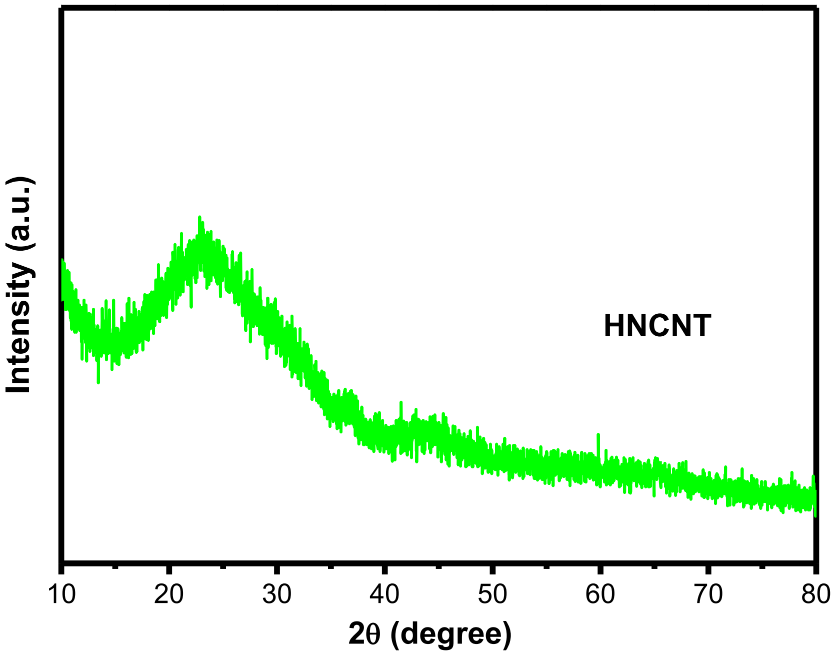

Supplementary Figure 25│XRD of HNCNT. The XRD patterns only show peak of graphitic carbon.

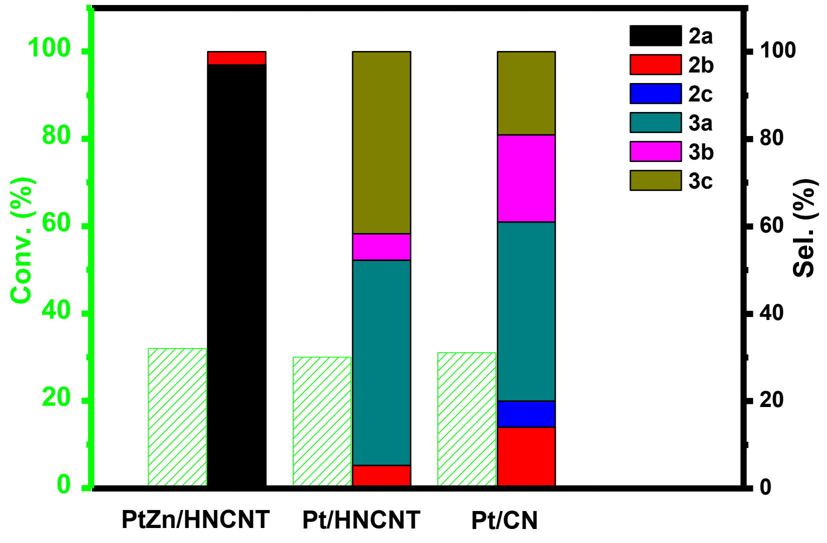

Supplementary Figure 26│The catalytic performance of PtZn/HNCNT, Pt/HNCNT and Pt/CN at similar conversion level. The selectivity of PtZn/HNCNT for **2a** is high (98%), while no such product could be observed for Pt/HNCNT and Pt/CN.

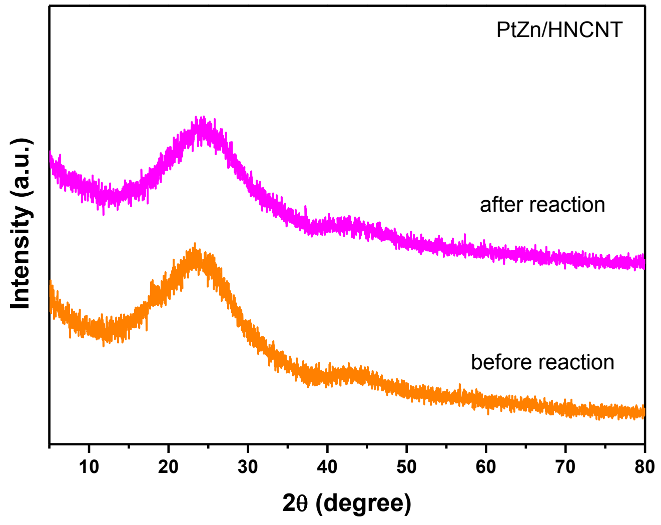

Supplementary Figure 27│XRD patterns of PtZn/HNCNT before and after the reaction. No obvious difference can be observed for the XRD.

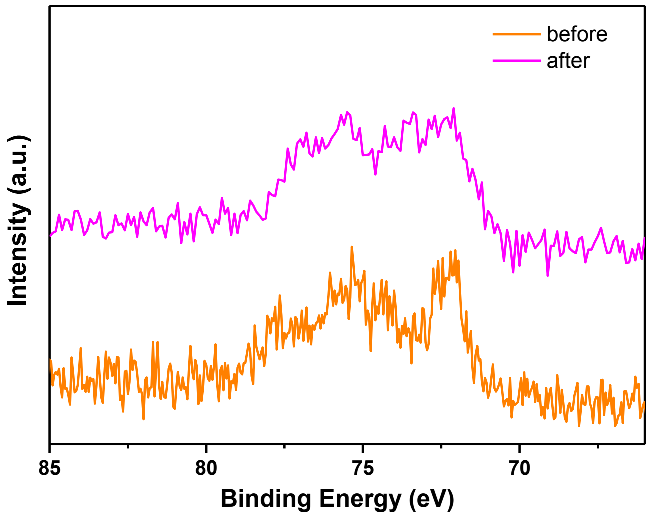

Supplementary Figure 28│Pt 4*f* XPS of PtZn/HNCNT before and after reaction. No obvious peak shift for the Pt 4*f* XPS can be observed.

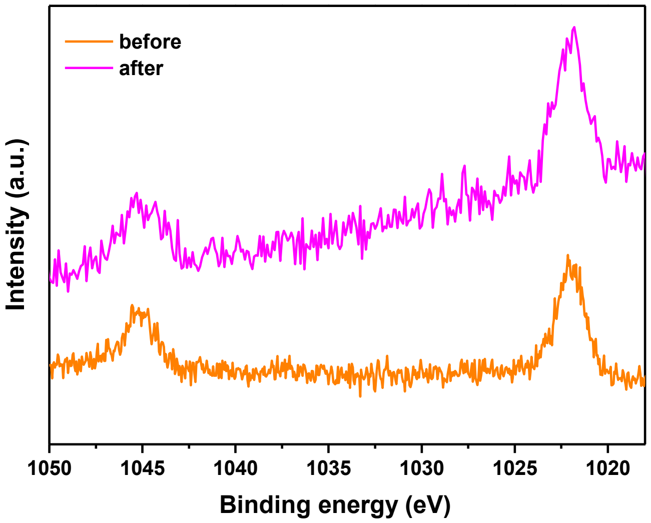

Supplementary Figure 29│Zn 2*p* XPS of PtZn/HNCNT before and after reaction. No obvious peak shift for the Zn 2*p* XPS can be observed.

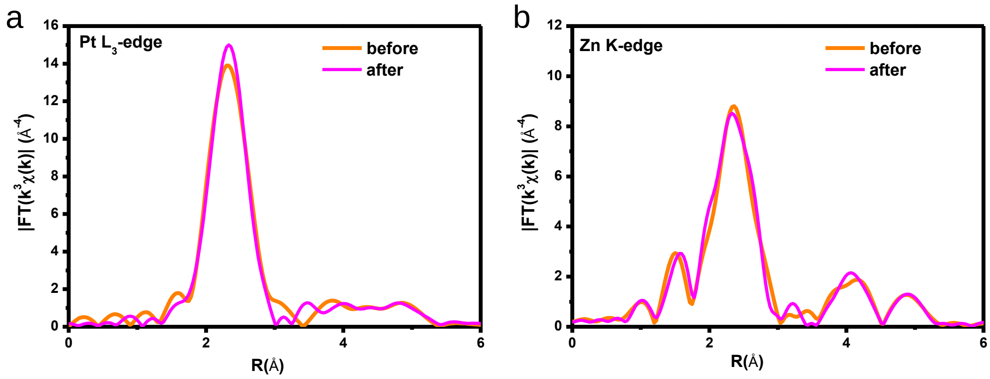

Supplementary Figure 30│The FT-EXAFS of PtZn/HNCNT before and after the reaction. **a** Pt L_3-edge and **b** Zn K-edge. There is no obvious change in the FT-EXAFS at Pt L_3-edge and Zn K-edge.

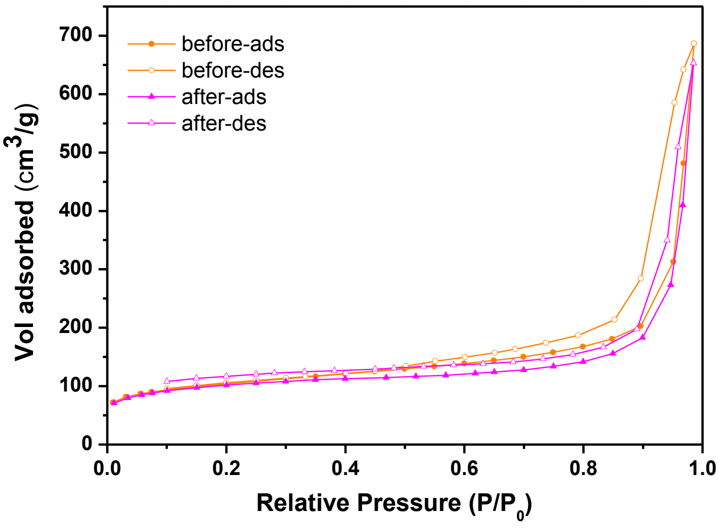

Supplementary Figure 31│N_2 adsorption and desorption isotherm of PtZn/HNCNT before and after the reaction. There is no obvious change in the nitrogen sorption isotherms of PtZn/HNCNT before and after the reaction.

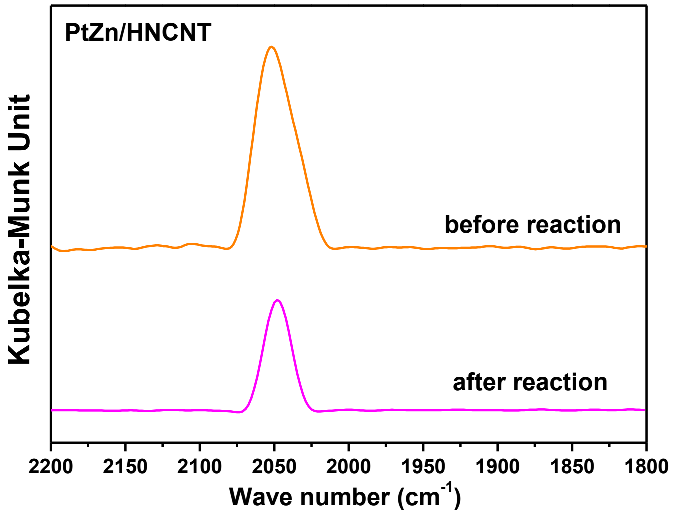

Supplementary Figure 32│*In situ* FTIR spectra of CO chemisorption of PtZn/HNCNT before and after the reaction. The CO chemisorption peak has no obvious shift.

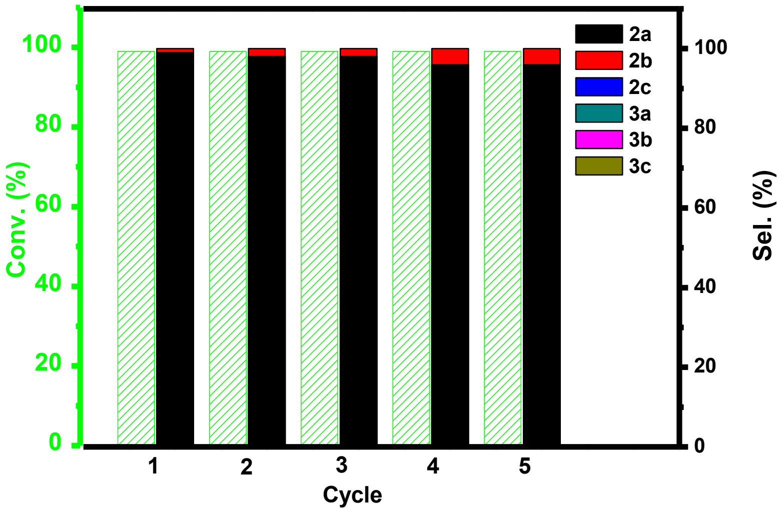

Supplementary Figure 33│Recyclability of PtZn/HNCNT in the selective hydrogenation of 4-nitrophenylacetylene. The conversion still keeps 100% after 5 runs while the selectivity has a bit decrease.

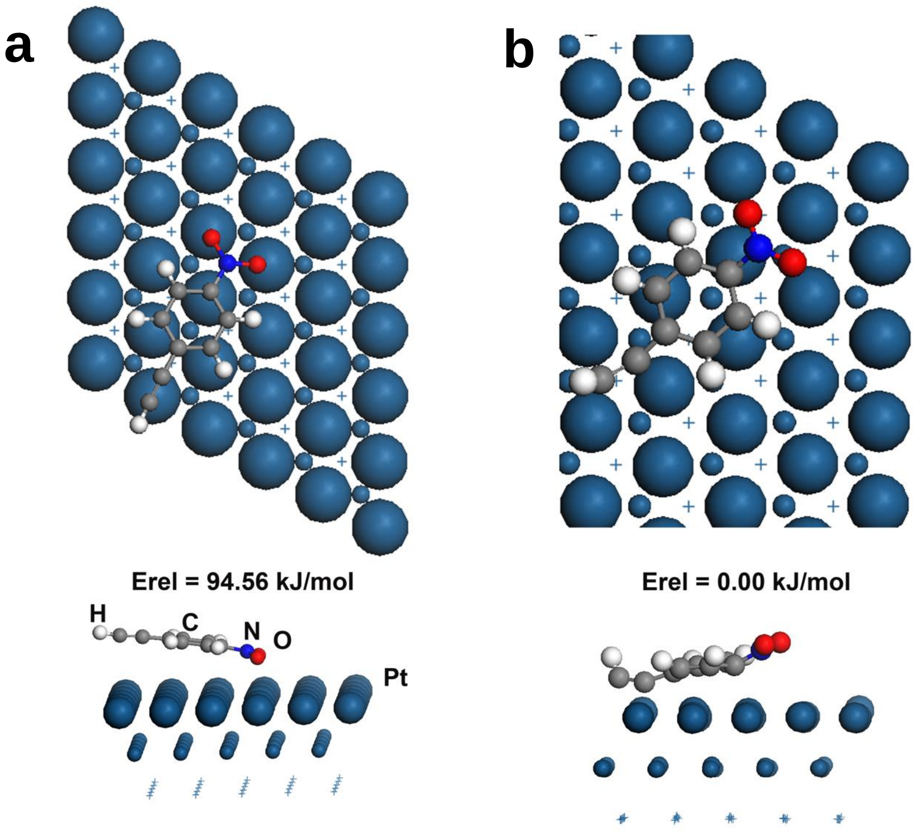

Supplementary Figure 34│Top and side view of adsorption configurations for 4-NPA@Pt(111) surface. The right configuration (b) is more stable.

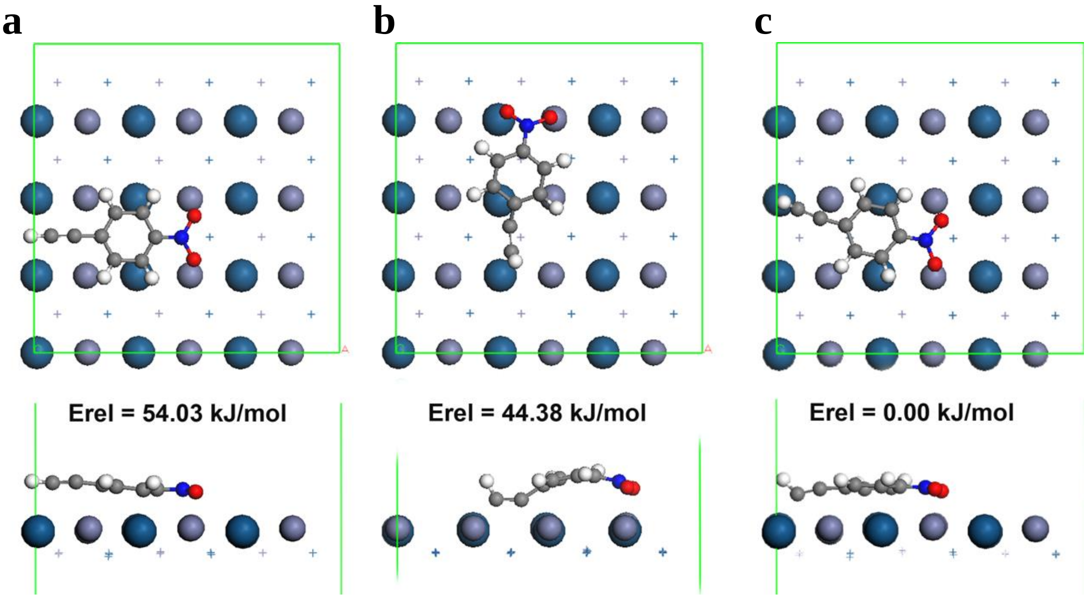

Supplementary Figure 35│Top and side view of adsorption configurations for 4-NPA@PtZn($02\bar{2}$) surface. Zn(grey). The right configuration (c) is more stable.

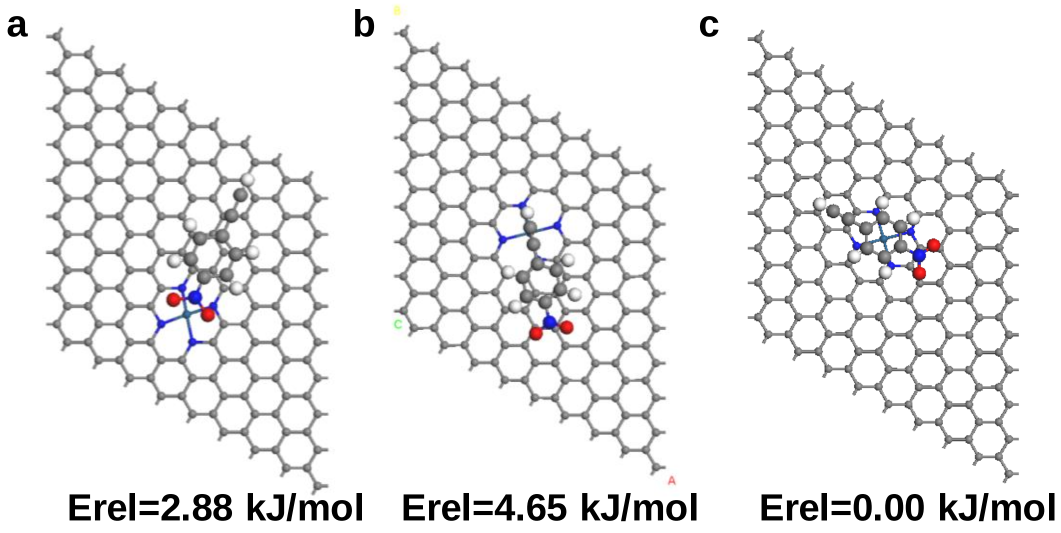

Supplementary Figure 36 | Different adsorption configurations for 4-NPA@Pt-N-C surface. The right configuration (c) is more stable.

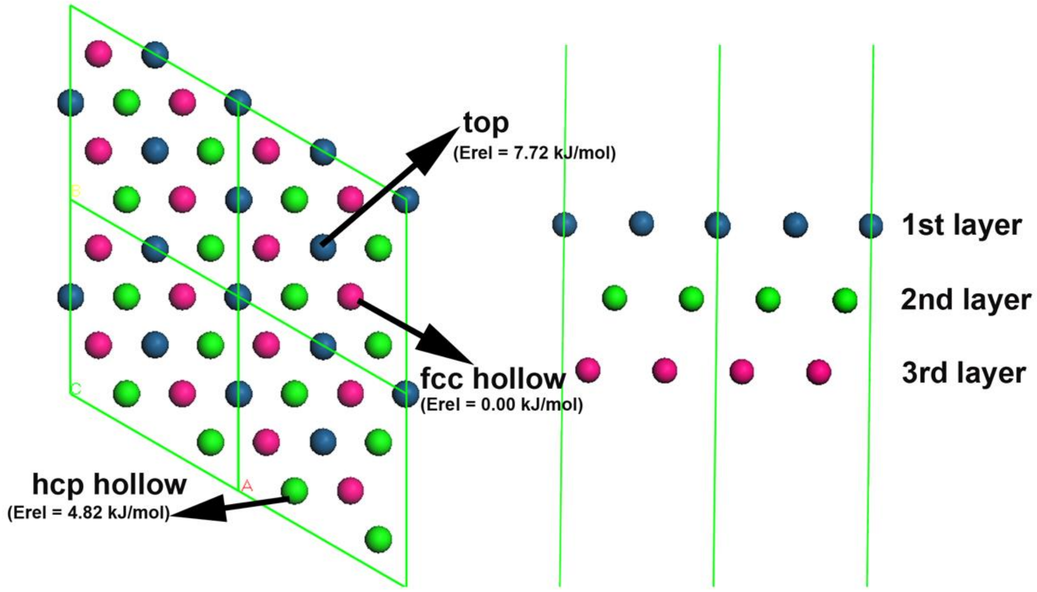

Supplementary Figure 37 | Schematic illustration of the high-symmetry sites on Pt(111) surface. The values in parentheses represent the relative total energy for hydrogen atom on the site.

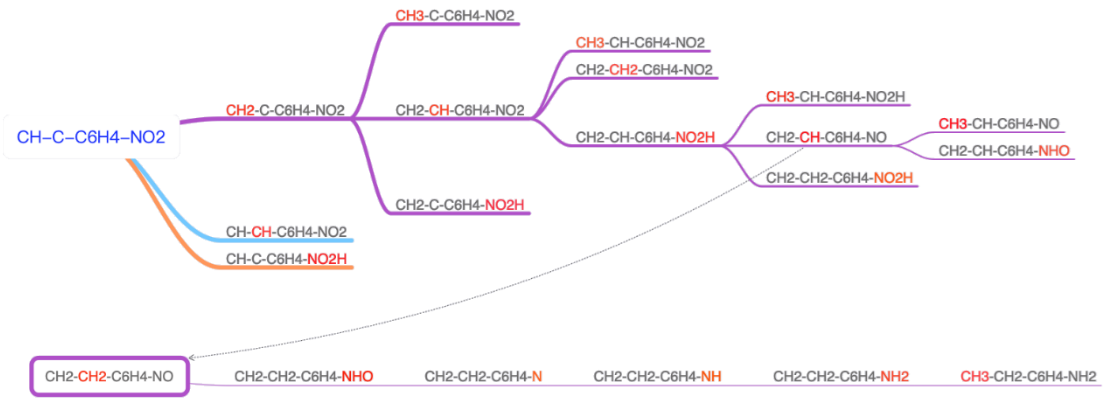

Supplementary Figure 38│The simplified hydrogenation reaction pathway of CH-C-C_6H_4-NO_2 on the Pt(111) surface. The hydrogenation sites are marked in red.

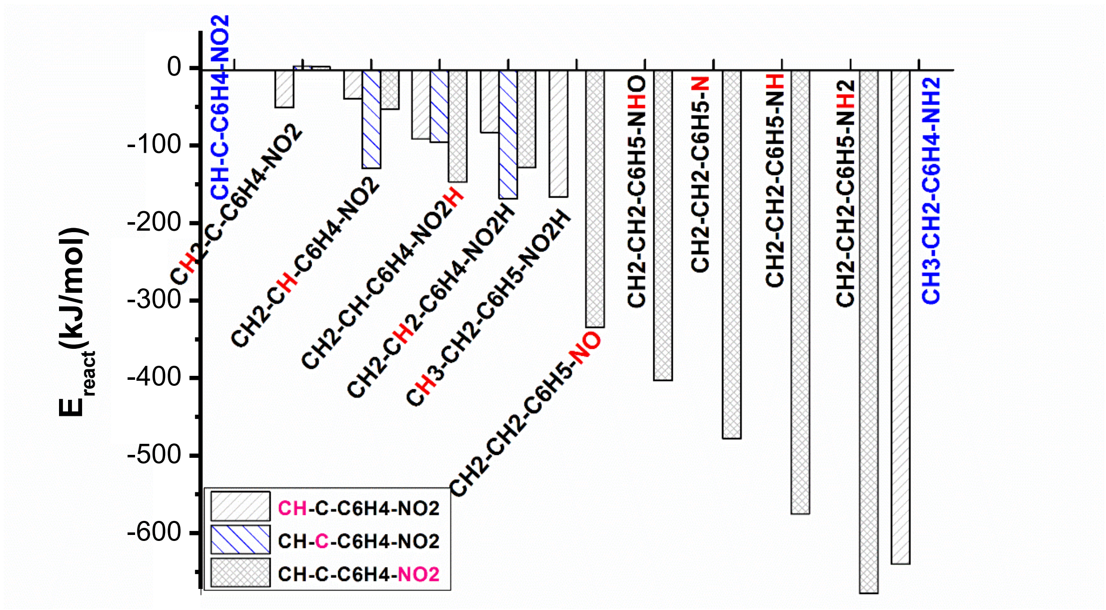

Supplementary Figure 39│The reaction energies of hydrogenation reaction pathway of CH-C-C_6H_4-NO_2 on the Pt(111) surface. The hydrogenation sites are marked in red.

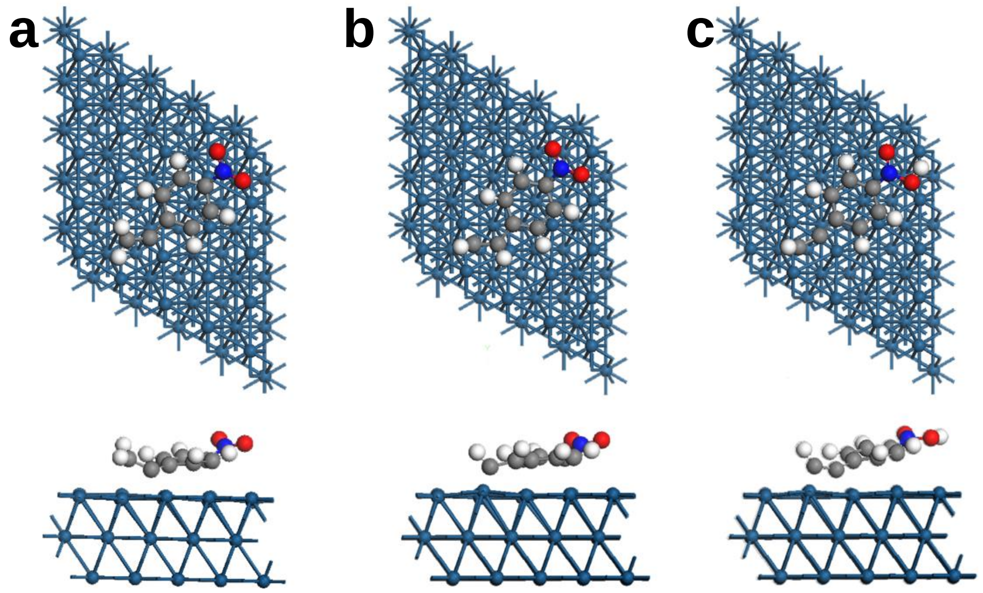

Supplementary Figure 40 | Optimized structures of intermediates for 4-NPA 1st hydrogenation reaction step over Pt(111) surface.

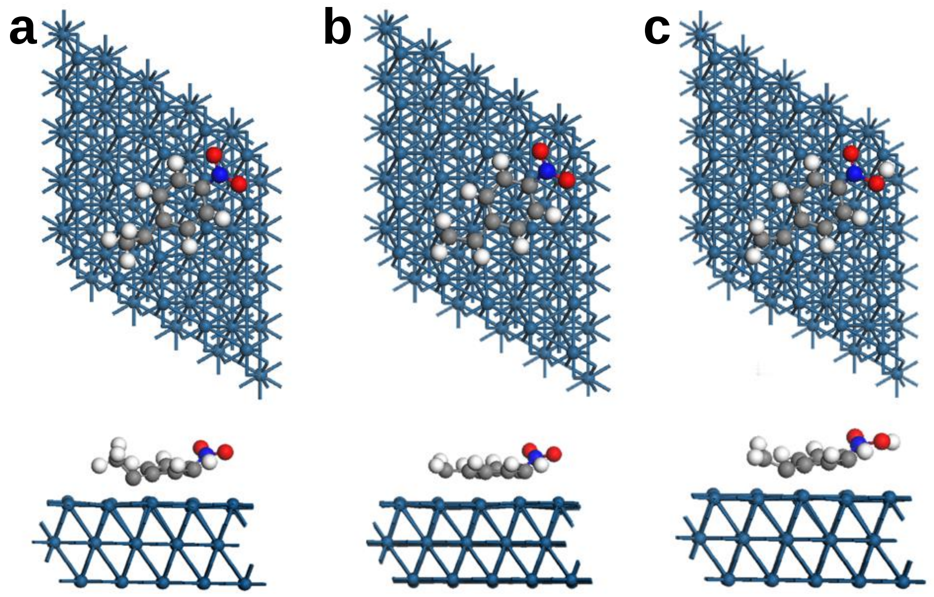

Supplementary Figure 41 | Optimized structures of intermediates for 4-NPA 2nd hydrogenation reaction step over Pt(111) surface.

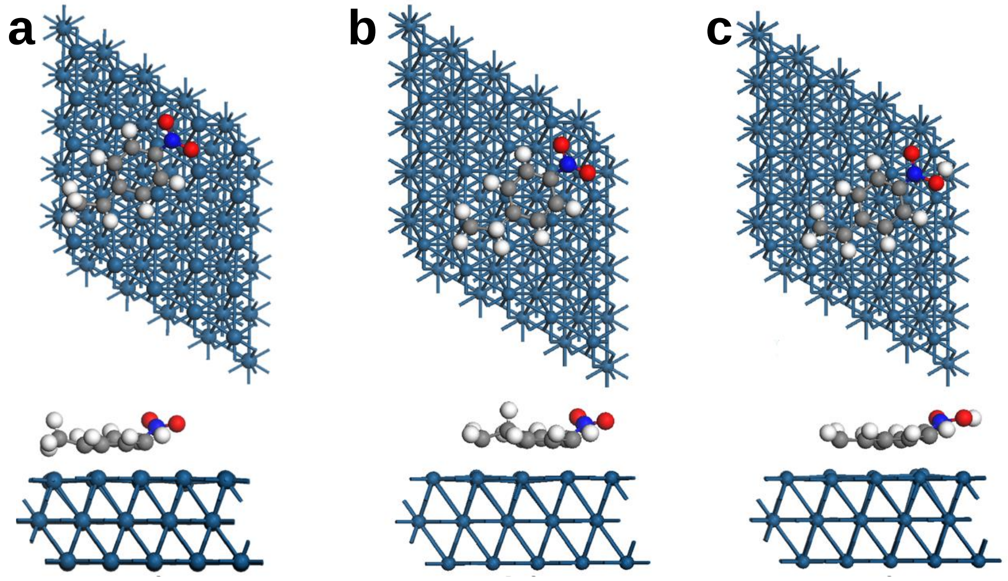

Supplementary Figure 42 | Optimized structures of intermediates for 4-NPA 3rd hydrogenation reaction step over Pt(111) surface.

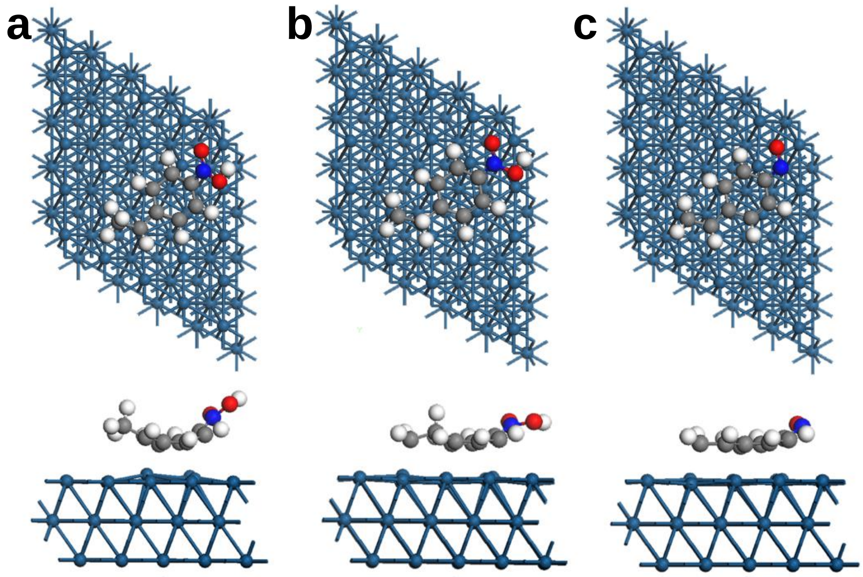

Supplementary Figure 43 | Optimized structures of intermediates for 4-NPA 4th hydrogenation reaction step over Pt(111) surface.

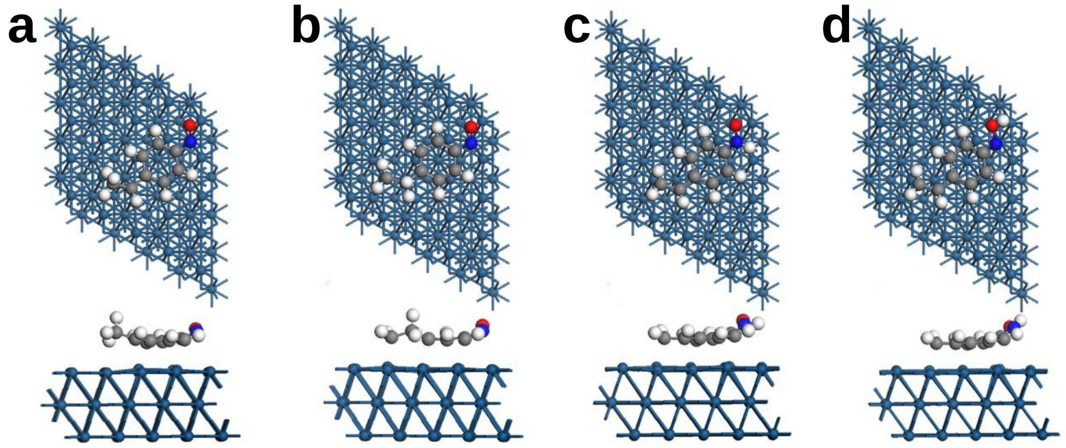

Supplementary Figure 44│Optimized structures of intermediates for 4-NPA 5th hydrogenation reaction step over Pt(111) surface.

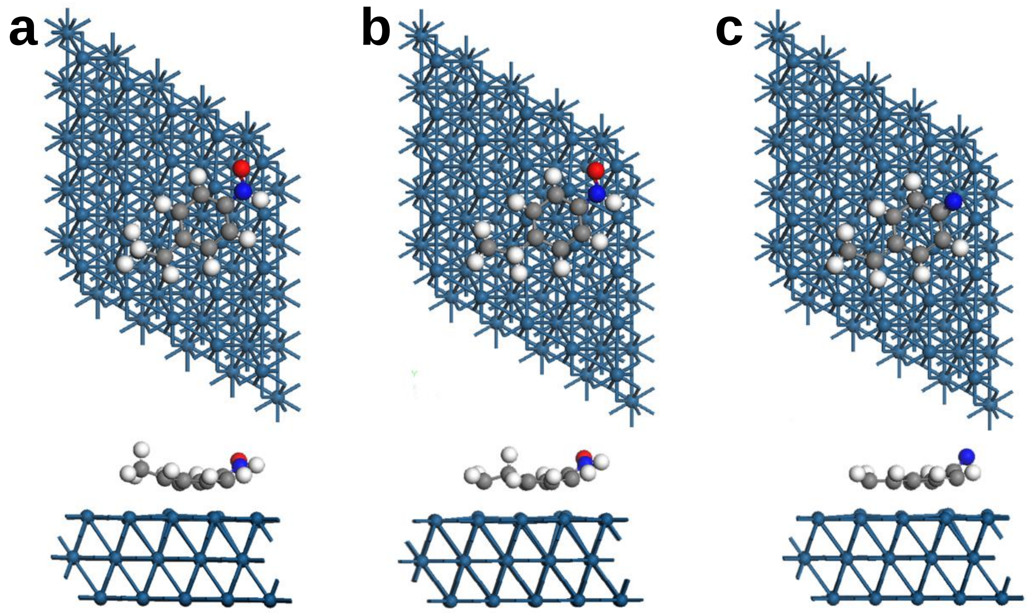

Supplementary Figure 45│Optimized structures of intermediates for 4-NPA 6th hydrogenation reaction step over Pt(111) surface.

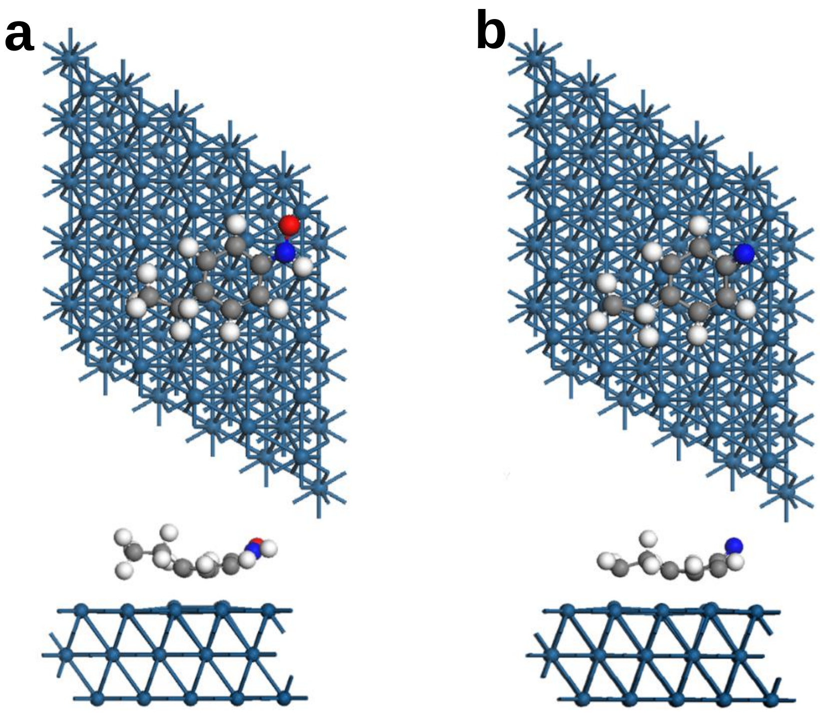

Supplementary Figure 46 | Optimized structures of intermediates for 4-NPA 7th hydrogenation reaction step over Pt(111) surface.

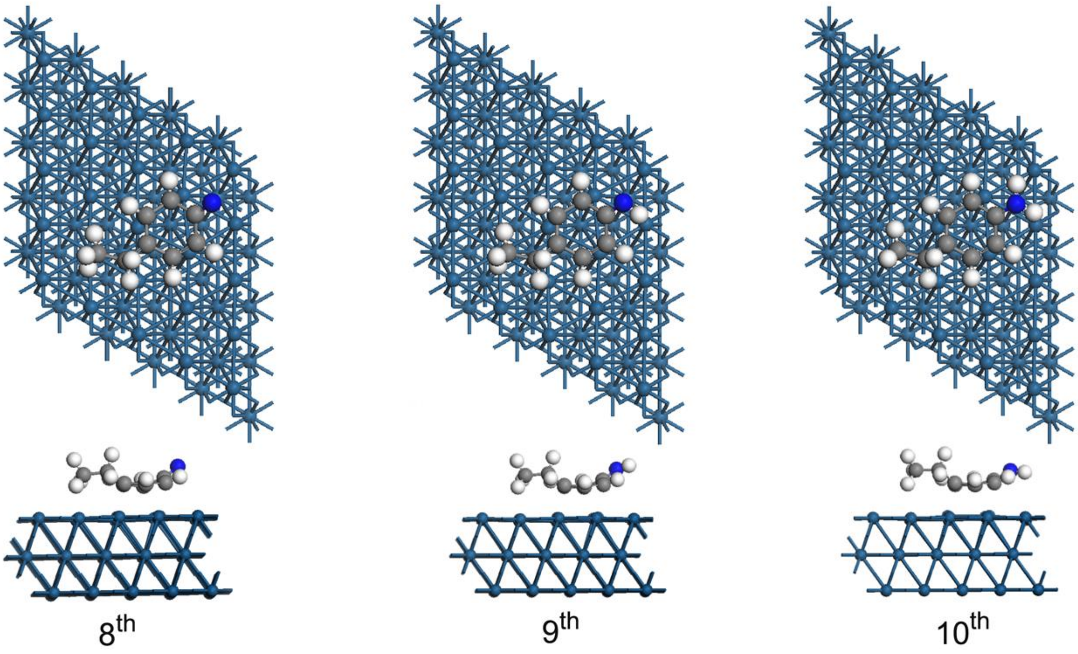

Supplementary Figure 47 | Optimized structures of intermediates for 4-NPA 8th, 9th and 10th hydrogenation reaction step over Pt(111) surface.

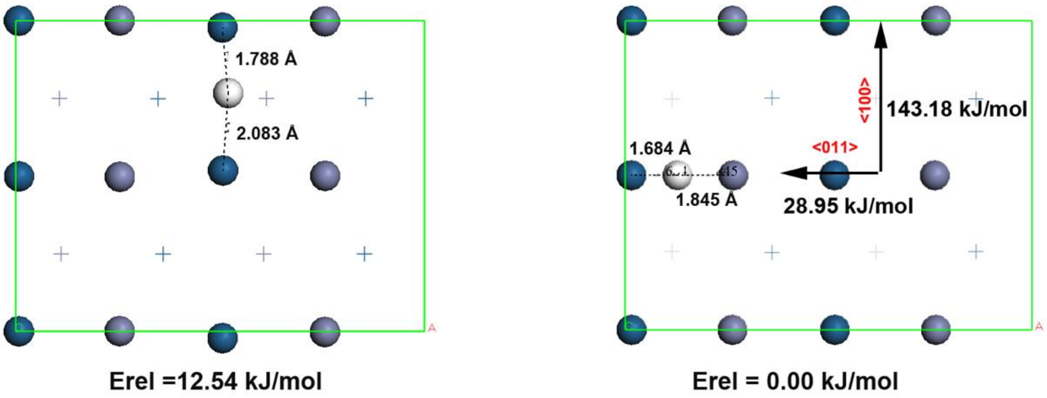

Supplementary Figure 48 | Top view of adsorption configurations for H@PtZn($02\bar{2}$) surface. The right configuration is more stable. Two hydrogen atom diffusion paths together with their diffusion barriers are labeled.

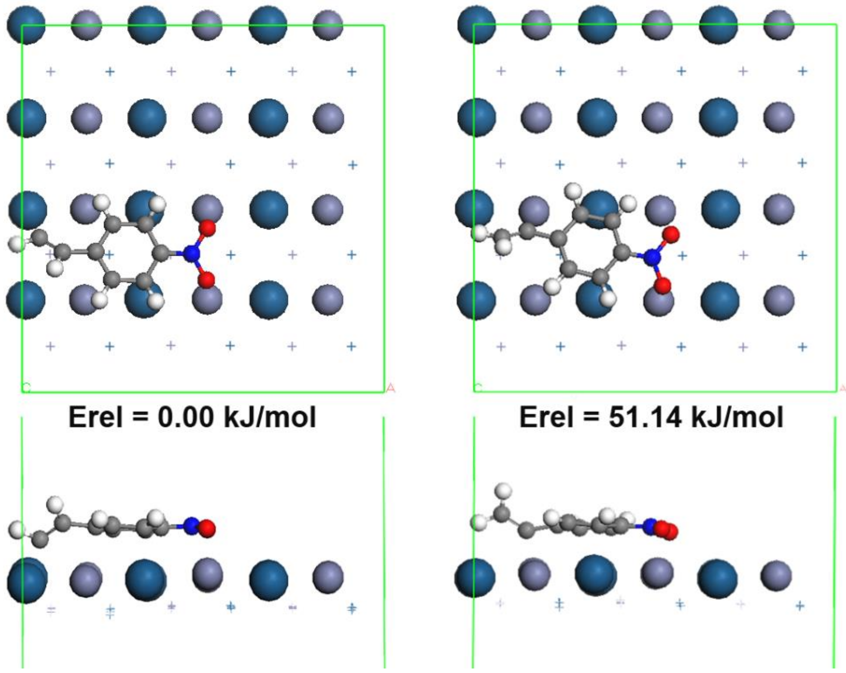

Supplementary Figure 49 | Top and side view of different product configurations of the first hydrogenation step of alkynyl group for 4-NPA@PtZn($02\bar{2}$) surface. The left configuration is more stable.

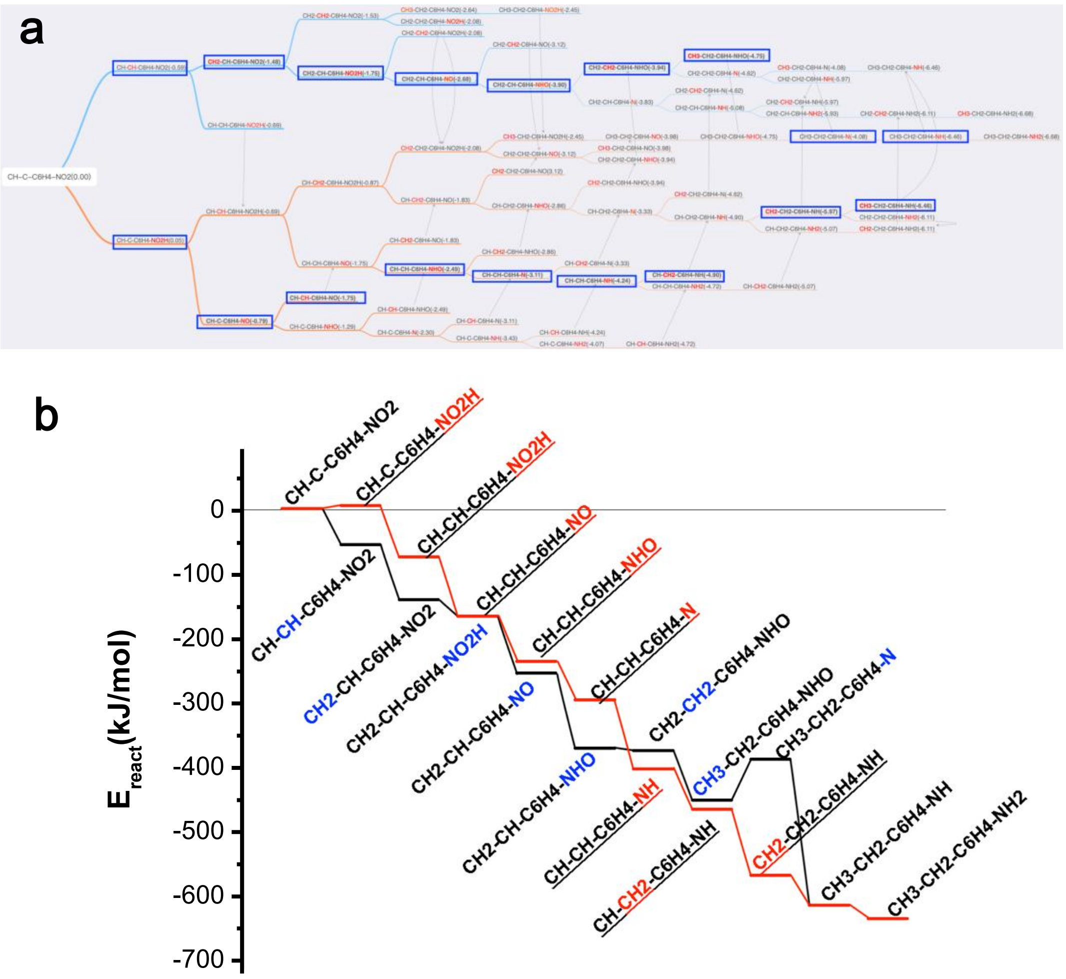

Supplementary Figure 50│Hydrogenation reaction pathway of CH-C-C_6H_4-NO_2 on the PtZn(02$\bar{2}$) surface. a The simplified hydrogenation reaction pathway. **b** The corresponding reaction energies of hydrogenation reaction pathway. The hydrogenation sites are marked in red. Blue boxes mark the intermediates with lower relative energies (unit : kJ/mol).

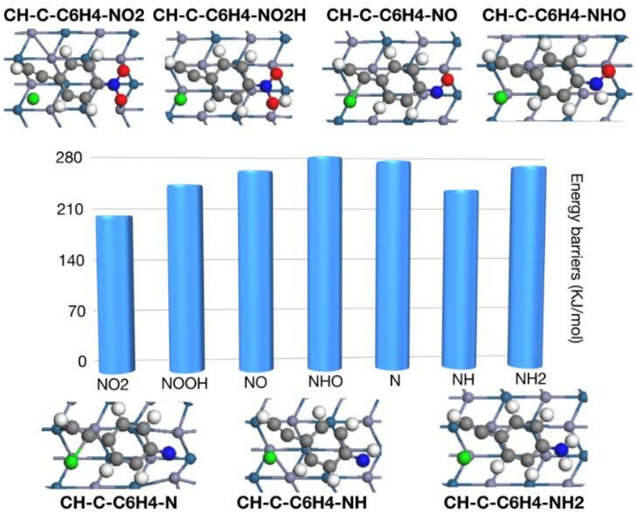

Supplementary Figure 51│The calculated energy barriers of hydrogenation reactions on alkynyl group for CH-C-C_6H_4-NO_2/NOOH/NO/NHO/N/NH/NH_2 on alloy surface. The insets show the corresponding geometries of transition states.

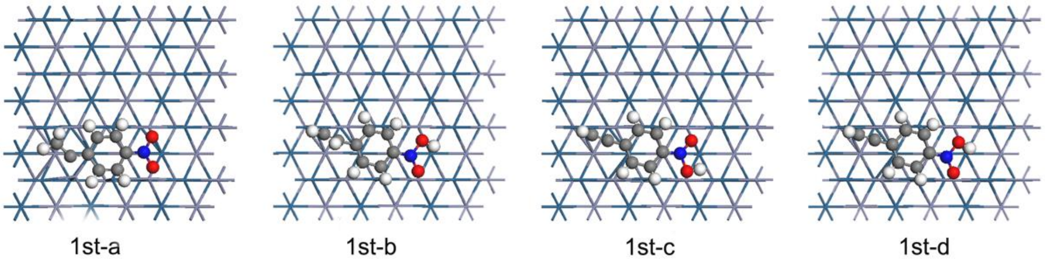

Supplementary Figure 52│Optimized structures of intermediates for 4-NPA 1st hydrogenation reaction step over PtZn($02\bar{2}$) surface.

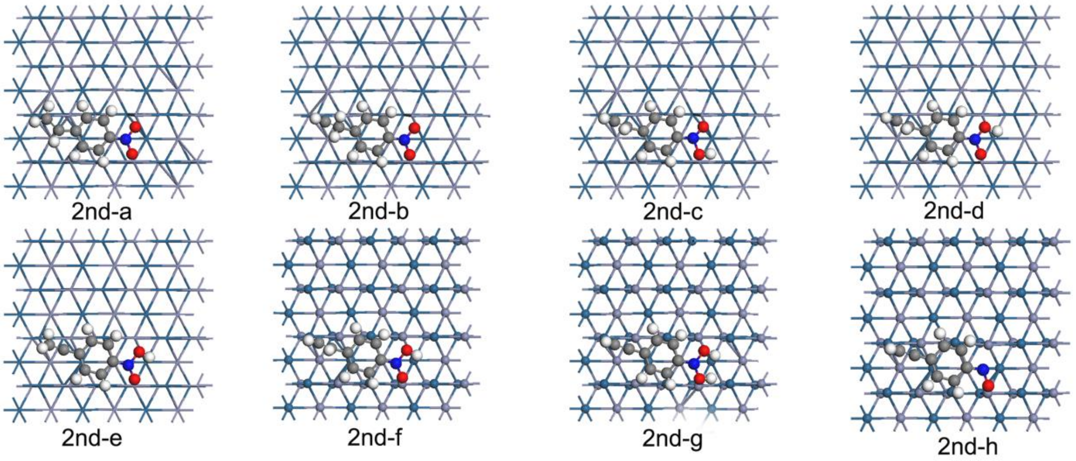

Supplementary Figure 53│Optimized structures of intermediates for 4-NPA 2nd hydrogenation reaction step over PtZn($02\bar{2}$) surface.

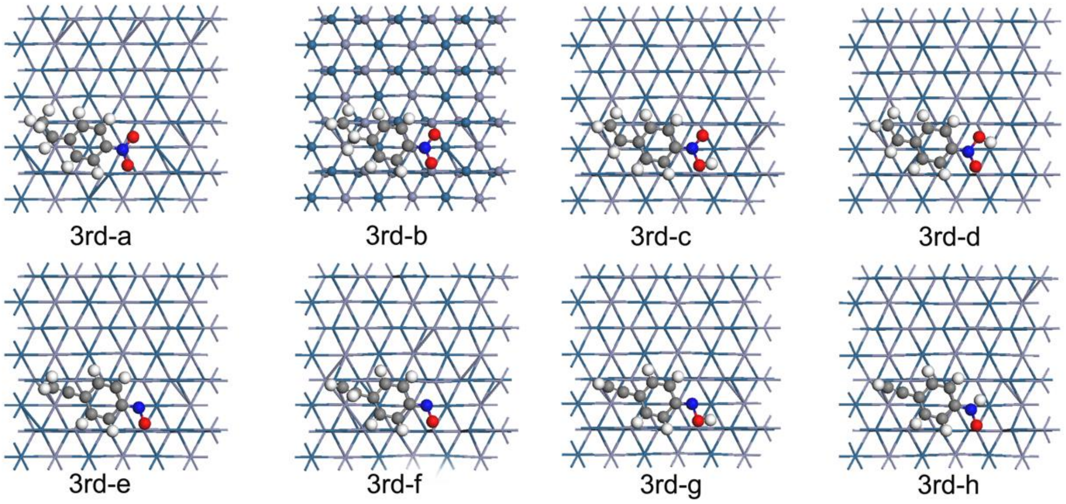

Supplementary Figure 54│Optimized structures of intermediates for 4-NPA 3rd hydrogenation reaction step over PtZn($02\bar{2}$) surface.

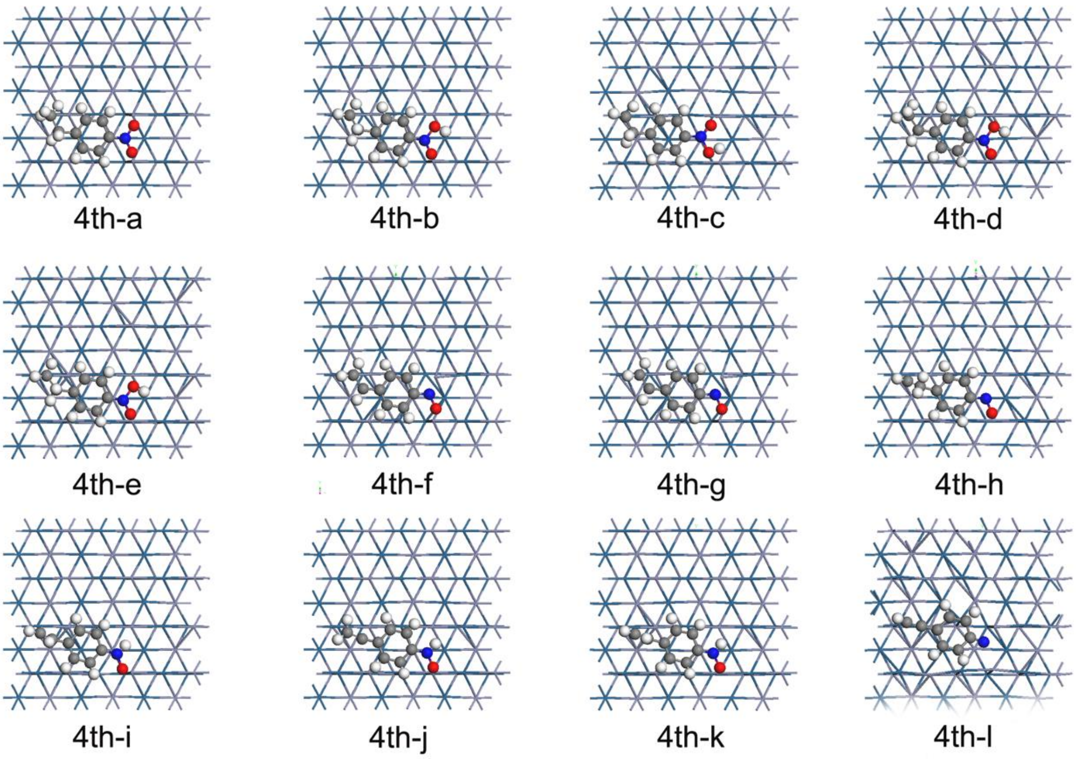

Supplementary Figure 55 | Optimized structures of intermediates for 4-NPA 4th hydrogenation reaction step over PtZn($02\bar{2}$) surface.

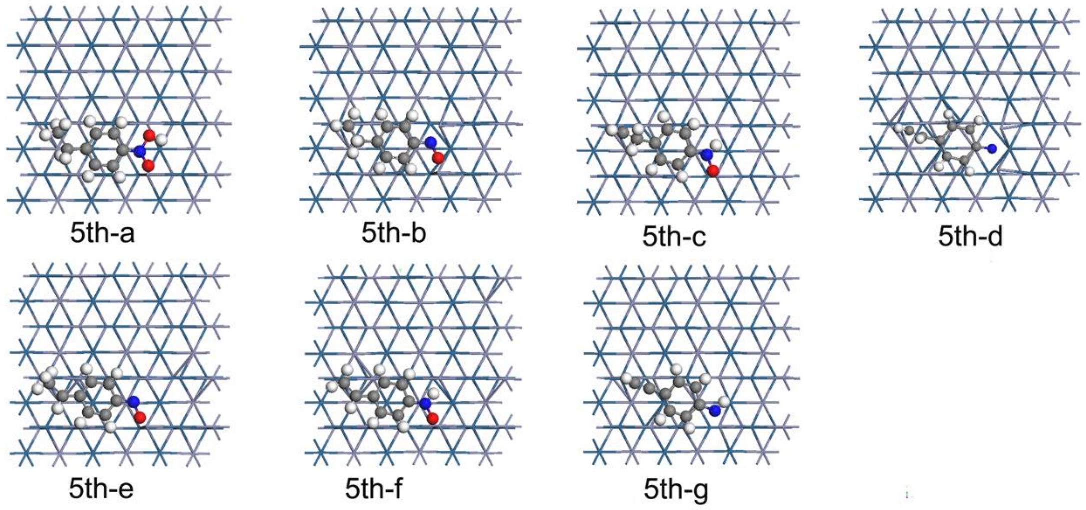

Supplementary Figure 56 | Optimized structures of intermediates for 4-NPA 5th hydrogenation reaction step over PtZn($02\bar{2}$) surface.

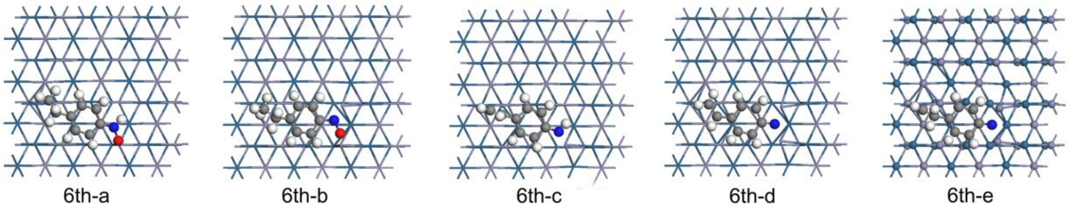

Supplementary Figure 57│Optimized structures of intermediates for 4-NPA 6th hydrogenation reaction step over PtZn($02\bar{2}$) surface.

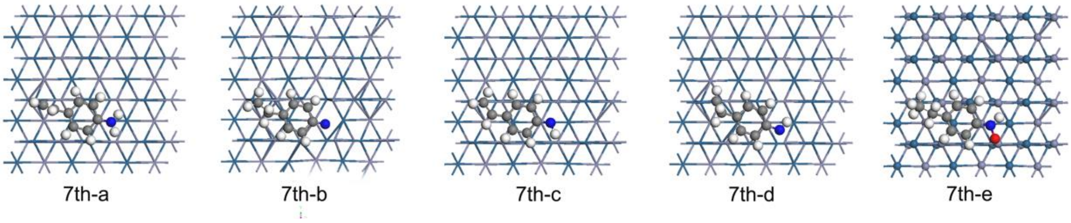

Supplementary Figure 58│Optimized structures of intermediates for 4-NPA 7th hydrogenation reaction step over PtZn($02\bar{2}$) surface.

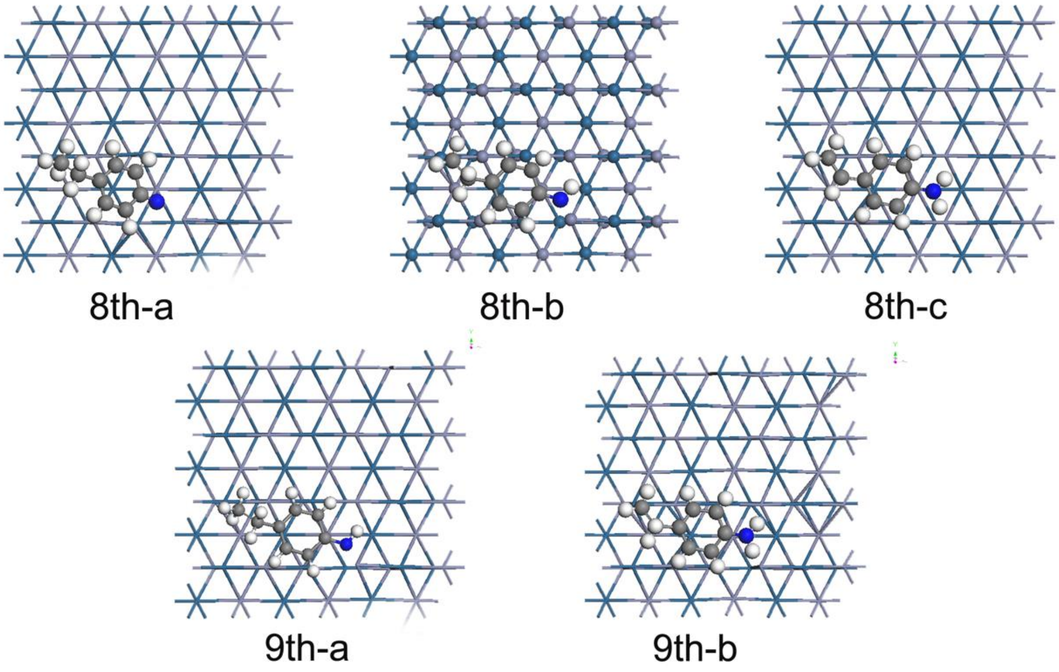

Supplementary Figure 59│ Optimized structures of intermediates for 4-NPA 8th abd 9th hydrogenation reaction steps over PtZn($02\bar{2}$) surface.

Supplementary Table 1. Summary of the EXAFS fitting results of PtZn/HNCNT, Pt/CN, and Pt/HNCNT.

sample	Scattering pair	C.N.	R(Å)	$\sigma^2(10^{-3}Å^2)$	R factor
PtZn/HNCNT	Pt-Zn	5.8 ± 1.1	2.66 ± 0.03	1.0 ± 0.3	0.005
Pt/CN	Pt-Pt	10.6 ± 1.6	2.78 ± 0.01	2.8 ± 0.2	0.002
Pt/HNCNT	Pt-N	3.8 ± 0.4	1.72 ± 0.04	6.0 ± 0.3	0.003

C.N. is the coordination number; R is interatomic distance (the bond length between central atoms and surrounding coordination atoms); σ^2 is Debye-Waller factor (a measure of thermal and static disorder in absorber-scatterer distances); R factor is used to value the goodness of the fitting.

Supplementary Table 2. Effect of temperature and pressure in the hydrogenation of 4-nitro phenylacetylene over the PtZn/HNCNT catalyst.

Entry	Temp (^oC)	Time (h)	Tube Vol (ml)	Conv.(%)	Sel.(%)
1	40	4	15	100	98.0
2	60	2	15	99.0	76.5
3	70	1	15	99.5	70.0
4	40	4	35	100	90.0

Condition: 4-nitrophenylacetylene 0.5 mmol, ammonia borane 3 mmol, catalyst 2.0 mg, ethanol 4.9 ml, deionized water 0.1 ml, reaction was carried out in a pressure tube.

Supplementary Table 3. Substrate scope of hydrogenation of functionalized nitrobenzenes over the PtZn/HNCNT catalyst.

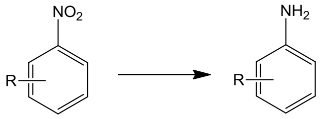

Entry	R	Conv.(%)	Sel.(%)
1	H	100	100
2	4-Cl	100	100
3	4-$CONH_2$	100	100
4	4-CH_2CH_3	94.9	100
5	4-NH_2	100	100
6	2-Cl	100	100
7	3-C≡CH	100	86.5

Condition: substrate 0.5 mmol, ammonia borane 3 mmol, catalyst 2.0 mg, ethanol 4.9 ml, deionized water 0.1 ml, reaction was carried out in a 15 ml pressure tube, reaction time 4 h.

References

1 Han, A. *et al.* A Polymer Encapsulation Strategy to Synthesize Porous Nitrogen-Doped Carbon-Nanosphere-Supported Metal Isolated-Single-Atomic-Site Catalysts. *Adv. Mater.* **30**, 201706508 (2018).

2 Perdew, J., Burke, K. & Ernzerhof, M. *Phys. Rev. Lett.* **77**, 3865-3868 (1996).

3 Clark, S. J. *et al.* First principles methods using CASTEP. *Z. Kristallogr.* **220**, 567-570 (2005).

4 McNellis, E. R., Meyer, J. & Reuter, K. Azobenzene at coinage metal surfaces: role of dispersive van der Waals interactions. *Phys. Rev. B* **80**, 205414 (2009).

5 Govind, N., Petersen, M., Fitzgerald, G., King-Smith, D. & Andzelm, J. A generalized synchronous transit method for transition state location. *Comput. Mater. Sci.* **28**, 250-258 (2003).
