# Supplementary material for: Isolating contiguous Pt atoms and forming Pt-Zn intermetallic nanoparticles to regulate selectivity in 4-nitrophenylacetylene hydrogenation
Source: Nat Commun. 2019 Aug 22;10:3787. doi: 10.1038/s41467-019-11794-6 (PMC6706404; doi:10.1038/s41467-019-11794-6)
Supplement: Supplementary file 2 — Peer Review [file 41467_2019_11794_MOESM2_ESM.pdf]

Reviewers' comments:

Reviewer #1 (Remarks to the Author):

This manuscript reports on the synthesis of isolated Pt atoms and Pt-Zn bridge sites and its applications for the hydrogenation of 4-nitrophenylacetylene. The manuscript is well written and interesting enough to be considered to publish in Nature Communications after authors address the points below properly. The followings are critiques and comments:

1) Authors reported that the selectivity over PtZn/HNCNT and Pt/HNCNT is very different, as shown in Figure 3. However, selectivity over different catalysts should be compared at similar conversion level because selectivity can be dependent on conversion. Also, please include the activity and selectivity results over Pt/CN catalyst for comparison.

2) Authors claimed that high temperature reduction induces the contiguous Pt atoms isolating by Zn atoms to form Pt-Zn bridge sites. XAS and electron microscope measurement were used to characterize the catalyst structure. However, I don't think those two techniques are enough to prove what authors claimed.
As shown in Figure 2, no Pt-Pt contribution was observed over PtZn/HNCNT, but, at the same time, there is no Pt-Pt contribution over Pt/HNCNT, potentially questioning the role of ZnO. Or, it is possible that XAS is not sensitive enough to see Pt-Pt contribution due to the low Pt loading. In this sense, CO FTIR characterization, a more surface sensitive technique, needs to be performed to fully characterize the structure of Pt species. I suggest performing CO FTIR characterizations on Pt/HNCNT, PT/CN, and PtZn/HNCNT before and after calcination.

3) It is well-known that Pt single atoms are susceptible to sintering under reductive reaction conditions.(Liu et al. 2018 Nanotechnology 29 204002) In this work, reaction temperature is low and ammonia borane was used as the hydrogen source for hydrogenation reaction, so Pt single atoms can be stable throughout the reaction. However, CO FTIR (and XAS if possible) of Pt/HNCNT, PT/CN, and PtZn/HNCNT should to be performed on the catalysts before and after reactions.
4) Authors need to provide more information about pretreatment after synthesis and before activity measurements and characterizations. Pretreatment information is important because the state of catalysts becomes different depending on pretreatment. For example, there was no description about the pretreatment done before catalytic activity measurement for 4-nitrophenylacetylene hydrogenation, XPS, and XAS measurements. If no pretreatment was done on catalysts before measurements, then characterizations on catalysts before and after reactions become more important.

5) In the experimental section, very limited information on characterizations was also provided. For XPS characterization, the binding energies need to be calibrated based on a reference point (typically the C1s peak) to compare the shift in binding energies but there is no description on calibration. In addition, I am not quite sure whether XPS was performed in situ or ex situ. Besides XPS, more detailed description on characterizations needs to be included.

6) Please add the following reference studying the selective 4-nitrophenylacetylene hydrogenation reaction over Pt/TiO2 catalyst by modifying the exposed Pt facets: Corma et al. J. AM. CHEM. SOC. 2008, 130, 8748–8753. This work tells us why the formation of Pt single atoms is important for the selective hydrogenation of 4-nitrophenylacetylene.

Reviewer #2 (Remarks to the Author):

The present study reported the high catalytic activity and selectivity of PtZn alloy supported on hollow nitrogen-doped carbon nanotubes toward the hydrogenation of 4-nitrophenylacetylene.The single Pt

atom embedded on the PtZn surface was identified as the active site in cooperation with the Zn site. I cannot recommend the publication of the manuscript in the present form. Major revisions considered the following comments should be made.

1. The enhanced catalytic performance of monometallic by forming alloys has been reported extensively before, e.g. Pt alloys in electrochemistry, Cu alloys for CO2 activation, and Pd alloys for hydrogenation. What is new for the present PtZn as compared to the previous?

2. Is there any surface segregation of Pt from the bulk to the PtZn surface and formation of Pt overlayer?

3. Is the carbon support play any role? The DFT modeling completely ignored the effect of support and the low-coordinated sites in the PtZn nanoparticles. Is it reasonable for comparison with experiment?

4.DFT calculations only considered the first hydrogenation step to correlate with the experimental observation. This is too simplified. A full reaction path way toward the experimental product should be considered, which improves the mechanistic understanding and makes the connection with the measurement.

5. The mechanistic understanding of the different behavior in selectivity between Pt and PtZn is missing. Or why can the formation of alloy change the selectivity? In-depth analysis from DFT calculations and/or well-defined characterization may help.

Reviewer #3 (Remarks to the Author):

The manuscript investigates the use of an intermetallic Pt-Zn catalyst for the selective hydrogenation of a nitro-compound (nitrophenylacetylene). The authors prepare the catalyst via a sacrificial template method where the ZnO nanorods are first coated with a polymer (PDA) and then deposited with a Pt precursor (Pt(OH)4). Reduction of the obtained material at high temperature leads to the final catalyst featuring Pt-Zn nanoparticles on a ZnO support. The material was characterized by standard ex situ techniques such as TEM, XPS, XRD, and XAS; catalytic tests were conducted in batch at fixed conditions. Finally, the authors initiated preliminary DFT calculations to rationalize the catalytic performance.

I have several points of concern with this work, rendering it not yet suitable for publication in a top-tier journal.

- From a synthetic point of view, the authors propose that high temperature reduction of Pt(OH)4/ZnO@PDA leads to zinc vapor, ‘which assists the contiguous Pt atom isolation’. From my understanding of the thermal evaporation deposition process, while zinc vapor is formed, the Pt is reduced, forming Pt0, which aggregates/clusters forming nanoparticles, incorporating part of the Zn from the surface. At the same time, the zinc vapor from the atmosphere cannot participate into the process. It does only after cooling down the system, where the vapor redeposits forming a new zinc layer (see Appl Catal B 2006, 62, 144-149). If this interpretation is correct, I would expect a core-shell nanoparticle, with a core of Pt, and a shell of Zn. This brings to additional questions on what is the real active phase and whether catalysis is happing on the zinc oxide surface. Thus, it is fundamental that the authors perform an in depth microscopic and spectroscopic investigation of the catalyst, profiling the full nanoparticle in depth.

- From Figure 1d, it appears that the catalyst has an average particle size of 10 nm. This is however contradicted by Figure 1f, where the average particle size is around 2 nm. A full particle size plot where more than 300 particles are measured is necessary. In this plot, please report error margins.

- ZnO is a reducible oxide and several other metal oxides (In_2O_3, CeO_2, $PtO_2 \cdot H_2O$) have been recently reported as selective hydrogenation catalysts. Catalytic tests with the pure oxide as benchmark need to be performed. This is to complement Figure 3, where the absence of Zn on the surface leads to incomplete conversion, suggesting zinc as the real active phase.

- Only one substrate has been hydrogenated (nitrophenylacetylene), and the final product was even not characterized by liquid 1H NMR, 13C NMR, TGA, DSC, LC-MS, and HR-MS, to prove that it is the correct product. How was the product isolated? Rotatory evaporation? What is the recovery mass yield?

- With one single substrate, the authors cannot claim that the work could find applications in the 'fine chemical sector'. Please include a broad substrate scope where the nitro-substrate is functionalized with electron donating and withdrawing groups as shown elsewhere (see ACS Catal 2015, 5, 3767-3778).

- In Figure 2, XAS is a bulk technique and cannot be used to prove surface Pt dispersion. Please conduct an in depth XPS analysis with Ar sputtering and chemisorption analyses to calculate the Pt dispersion.

- The DFT profile in Figure 4 is too preliminary since only H_2 splitting has been calculated. Please show the full hydrogenation profile with the remaining steps (till product desorption).

- The authors have preferred to reference to their own papers and several outstanding contributions and advances on hydrogenation catalysis from 2010 to today have been omitted.

- The catalytic tests are also incomplete. The authors have not shown any catalyst stability, demonstrating absence of metal restructuring upon 20-40 h on stream. The stability has to be integrated, of course, with the complete characterization of the used catalyst (metal content, XPS, XAS, XRD, physisorption, and chemisorption). The catalytic performance has to be complemented with a full kinetic analysis, where the authors take a material and investigate the effect of temperature and pressure, in order to identify the optimal reaction conditions. In the Supporting Information, it is also evident that the authors have taken only 2 mg of catalyst and performed the tests with such a small quantity of material. This is however (at least in my experience) unpractical, since small variation in the mass (2.5 mg instead of 2) will bring to huge differences (25% more). Please conduct new tests using at least 100 mg of catalyst.

- What is the real active phase? I propose as well to complement the work with some operando XAS studies to address this important question.

- Intermetallic catalysts (Pd-Zn and Pt-Zn) have been already widely reported (see Catal Lett 1998, 54, 119-123). Can the author compare both an intermetallic Pt-Zn with core-shell catalysts (Pt in the core with Zn on the surface; Pt on the surface and Zn in the core).

- Please remove lines 141-143, since this is not correct.

- The paper is a clear example on catalysis on nanoparticles. The authors, however, have preferred to choose the catchy 'Pt-Zn bridge sites' terminology (title and abstract) to recall the exciting and fast-moving field of single-site catalysis, likely to attract citations. This title is however misleading, considering that the work provides little insights on single-site catalysis, and thus must be modified, substituting 'bridge sites' with 'intermetallic nanoparticles'.

Dear all reviewers,

Thank you very much for reviewing our manuscript entitled "Isolating contiguous Pt atoms and forming Pt-Zn bridge sites to regulate selectivity in catalytic hydrogenation of 4-nitrophenylacetylene" (reference number: NCOMMS-18-27012). We have carefully read your comments and appreciate for your comments which help us improve the quality of the whole manuscript. Based on the reviewers' comments and the revision request, we have tried our best to revise the manuscript and supplementary information accordingly. The reviewers' comments are laid out below in *Italic font* and specific concerns have been numbered. Our responses are given in normal font and changes/additions to the manuscript and supplementary information are highlighted by using blue colored text.

Following is our point to point answer to all the comments:

Reviewer #1:

This manuscript reports on the synthesis of isolated Pt atoms and Pt-Zn bridge sites and its applications for the hydrogenation of 4-nitrophenylacetylene. The manuscript is well written and interesting enough to be considered to publish in Nature Communications after authors address the points below properly. The followings are critiques and comments:

Comment 1. *Authors reported that the selectivity over PtZn/HNCNT and Pt/HNCNT is very different, as shown in Figure 3. However, selectivity over different catalysts should be compared at similar conversion level because selectivity can be dependent on conversion. Also, please include the activity and selectivity results over Pt/CN catalyst for comparison.*

Response: We sincerely appreciate your valuable suggestion. As shown in Figure 3a, Pt/HNCNT exhibited 47% selectivity to **3a** at the conversion of 29.3%. A reaction kinetic curve of PtZn/HNCNT is added in Supplementary Fig. 18. As can be seen from Supplementary Fig. 18, at a similar conversion level (40%), PtZn/HNCNT exhibited 99% selectivity to **2a**. Based on these results, it can be seen that PtZn/HNCNT prefers hydrogenation of the nitro group while Pt/HNCNT prefers the alkynyl group.

Pt/CN is prepared by a similar method but with polydopamine nanospheres as the support (the synthesis method is given in the Supplementary Information Page 2). The activity and selectivity of Pt/CN is added in Figure 3 in the revised manuscript. It showed 39.8% conversion, while the selectivity is 4.8% for **2c**, 11.6% for **2b**, 19.3% for **3b**, 34.2% for **3a**, and 16.2% for **3c**. Pt/CN exhibited preference to the hydrogenation of alkynyl group, which agrees well with that of Pt/HNCNT. It further indicates that the hydrogenation of alkynyl group is preferred without zinc.

A discussion is therefore added in page 9 of the revised manuscript.

"In comparison, the Zn-free catalyst Pt/HNCNT was also applied into such reaction, but exhibited rather different performance as compared to PtZn/HNCNT. Nearly 30% conversion was observed after 4 h while a complex mixture containing 5% of **2b**, 47% of 4-nitrostyrene (**3a**), 6% of 4-nitroethylbenzene (**3b**) and 42% of 4-nitroacetophenone (**3c**) (generated from the oxidation of **3a** by the remaining air during the reaction process) was obtained. Nearly no reaction occurred in the absence of Pt (Supplementary Fig. 19-20), proving that the active species are the Pt metals.

Pt/CN, which is prepared without using ZnO and thus bearing Pt nanoparticles supported on CN support, was also compared. It exhibited similar selectivity to that of Pt/HNCNT, with 34.2% selectivity to **3a**. Obviously, PtZn/HNCNT catalyzed the hydrogenation of 4-NPA in different routes to Pt/HNCNT and Pt/CN."

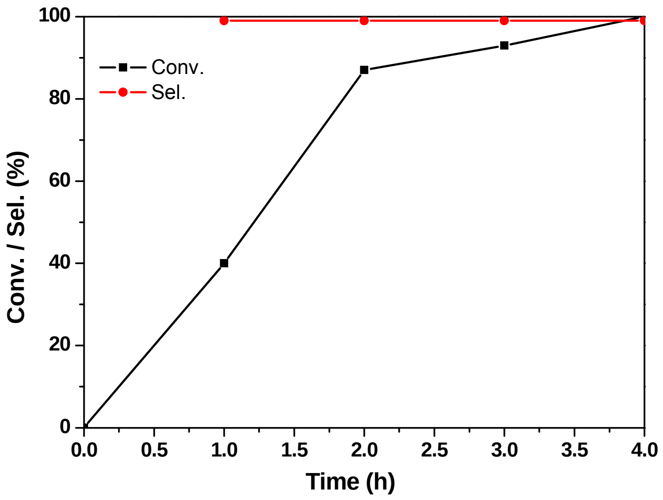

Supplementary Figure 18. Kinetic profile of 4-nitrophenylacetylene hydrogenation over PtZn/HNCNT.

Comment 2. *Authors claimed that high temperature reduction induces the contiguous Pt atoms isolating by Zn atoms to form Pt-Zn bridge sites. XAS and electron microscope measurement were used to characterize the catalyst structure. However, I don't think those two techniques are enough to prove what authors claimed. As shown in Figure 2, no Pt-Pt contribution was observed over PtZn/HNCNT, but, at the same time, there is no Pt-Pt contribution over Pt/HNCNT, potentially questioning the role of ZnO. Or, it is possible that XAS is not sensitive enough to see Pt-Pt contribution due to the low Pt loading. In this sense, CO FTIR characterization, a more surface sensitive technique, needs to be performed to fully characterize the structure of Pt species. I suggest performing CO FTIR characterizations on Pt/HNCNT, PT/CN, and PtZn/HNCNT before and after calcination.*

Response: We sincerely appreciate your valuable suggestion.

CO FTIR characterizations on Pt/HNCNT, Pt/CN, PtZn/HNCNT before and after calcinations are measured and shown below in Figure R1-2. However, the peaks are rather low, which may be due to the low loading of Pt. No much difference could be observed between the sample Pt/HNCNT, Pt/CN, and PtZn/HNCNT.

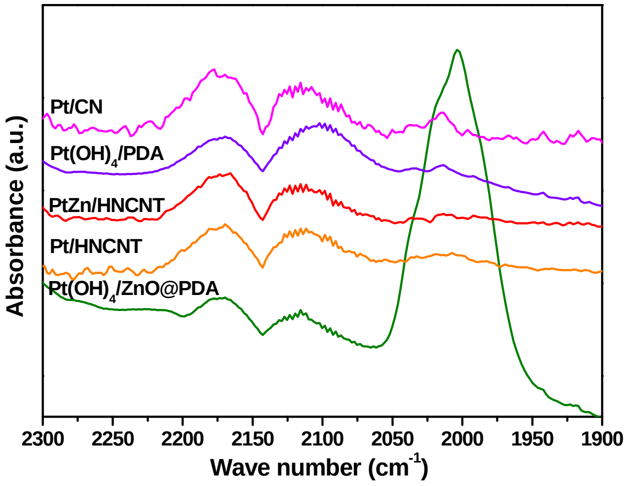

Figure R1-2. IR-CO chemisorptions stack-plot spectra of PtZn/HNCNT, Pt/HNCNT, $Pt(OH)_4$/ZnO@PDA, Pt/CN and $Pt(OH)_4$/PDA. The in situ Fourier transform infrared (FTIR) spectra of CO adsorption were carried out on a Nicolet 380/ZDF-X-LED in situ infrared spectrometer. The sample (if the transmittance is not satisfied, the sample was mixed with a little amount of KBr) was palletized into a disk. Prior to the CO adsorption, the sample were pretreated with Ar at 200 ^{o}C for 1 h. After cooling down to room temperature (20 ^{o}C), the background spectrum was collected. Then, CO gas was introduced into the sample holder for 30 min. The sample was flushing with Ar and a spectra was then collected every 5 min over the course of 30 min. The spectra shown in this figure are taken after 30 min of flushing with Ar.

However, we tried our best to testify that Pt atoms are isolated by Zn atoms and forming Pt-Zn bridge sites from other characterizations. Firstly, we prepared a sample with higher loading of Pt (1%) using the same method (it is added as Supplementary Fig. 6). Its XRD patterns agreed well with that of PtZn (JCPDS No. 06-0604). This observation further indicated the Pt dispersion can be influenced by zinc due to the high temperature. Secondly, we did more microscopic characterization. A clear image showing the atomic arrangement of Pt and Zn from AC HAADF STEM is observed and added as Supplementary Fig. 7 in the revised Supplementary Information. Discussions about these two new evidences are added in page 6 of the revised manuscript and we hope it could convince your respected reviewers and readers.

"XRD was then performed to examine the phase composition of the samples (Supplementary Fig. 5). Only graphitic carbon peaks were observed because of the ultrasmall size of the PtZn particles. A sample with higher Pt loading (1%) is prepared using the same method. The diffraction peaks in XRD (Supplementary Fig. 6) match well to that of PtZn (JCPDS No 06-0604). To further testify the IMC nature of PtZn particles, AC HAADF STEM measurement was carried out. AC HAADF STEM of one PtZn nanoparticle exhibited clear bright dots, in agreement with the atomic arrangement of ($02\bar{2}$) plane in the PtZn crystal structure (Fig. 1e). The lattice spacing of 0.221 and 0.203 nm matched interplane distances of (111) and (200) planes of intermetallic PtZn (P4/mmm) respectively (Fig. 1f). The perfect agreement testified the nanoparticle is PtZn intermetallic compound. More AC HAADF STEM images are taken to prove that the nanoparticles are PtZn

intermetallic compound. As shown in Supplementary Fig. 7, the atomic arrangement of another nanoparticle could match well with the (010) plane of PtZn. Due to the different atomic density, the heavier Pt atoms (bright) can be distinguished from Zn atoms (darker). Therefore, we think that the PtZn intermetallic compounds instead of core-shell alloys are formed in the PtZn/HNCNT."

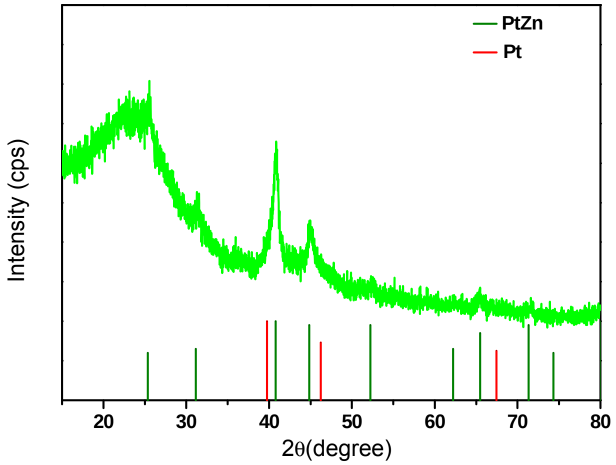

Supplementary Figure 6. XRD of PtZn/HNCNT prepared from 1% Pt/ZnO@PDA.

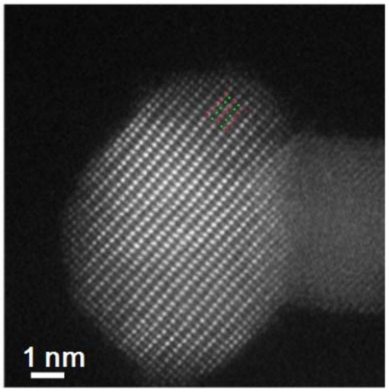

Supplementary Figure 7. AC HAADF STEM image of a PtZn nanoparticle. The red and green spheres represent Pt and Zn atoms, respectively.

Comment 3. *It is well-known that Pt single atoms are susceptible to sintering under reductive reaction conditions.(Liu et al. 2018 Nanotechnology 29 204002) In this work, reaction temperature is low and ammonia borane was used as the hydrogen source for hydrogenation reaction, so Pt single atoms can be stable throughout the reaction. However, CO FTIR (and XAS if possible) of Pt/HNCNT, PT/CN, and PtZn/HNCNT should to be performed on the catalysts before and after reactions.*

Response: We appreciate your valuable comment. The CO FTIR of Pt/HNCNT, Pt/CN and PtZn/HNCNT before and after reactions were carried out, and the spectra are shown below in Figure R1-3. Even though no obvious changes could be observed, the peaks are low due to the low

loading of Pt and cannot be used to state that the samples are stable, so we did some other experiments to prove the stability of the sample. The XAS of PtZn/HNCNT before and after reactions are added in Supplementary Fig. 24. There is no obvious difference of the FT-EXAFS spectra after the reaction, suggesting that the materials are stable under this reaction condition. Besides, metal content, XRD, XPS, and N_2 sorption of the catalyst after reaction are also carried out and added in Supplementary Fig. 21-25. No obvious changes were observed, further indicating the stability of the sample. A discussion is added in page 9.

"Besides, the stability of the catalyst was tested. After reaction, there was nearly no change in the metal content (0.82% before and 0.80% after reaction), XRD, XPS, XAS, and N_2 sorption for PtZn/HNCNT (Supplementary Fig. 21-25). No obvious decrease in the catalytic performance was observed (Supplementary Fig. 26), suggesting the robust nature of the PtZn/HNCNT."

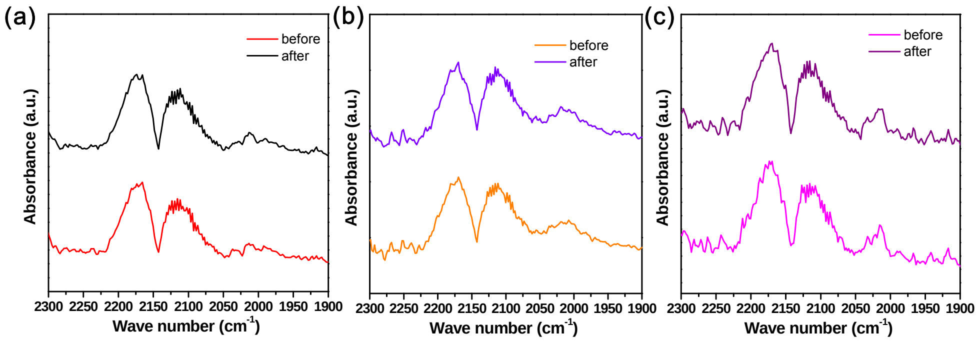

Figure R1-3. CO FTIR spectra of (a) PtZn/HNCNT, (b) Pt/HNCNT and (c) Pt/CN before and after the reaction.

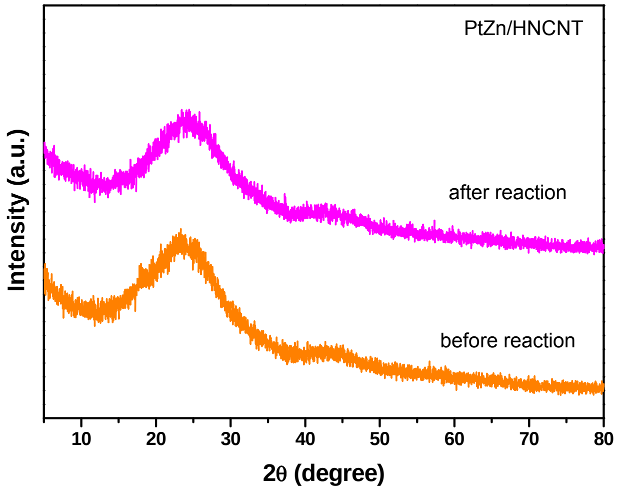

Supplementary Figure 21. XRD patterns of PtZn/HNCNT before and after the reaction.

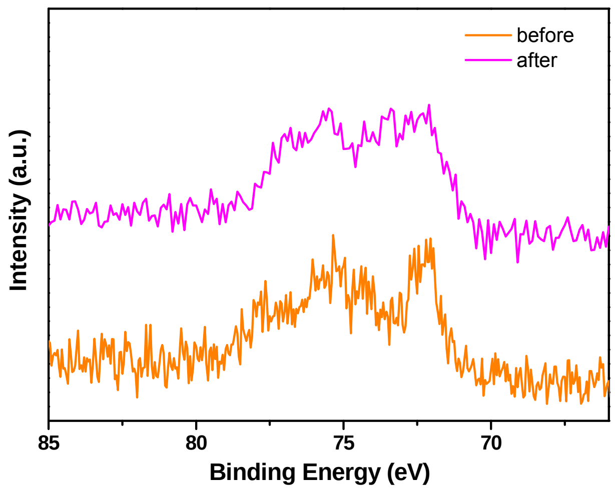

Supplementary Figure 22. Pt 4f XPS of PtZn/HNCNT before and after reaction.

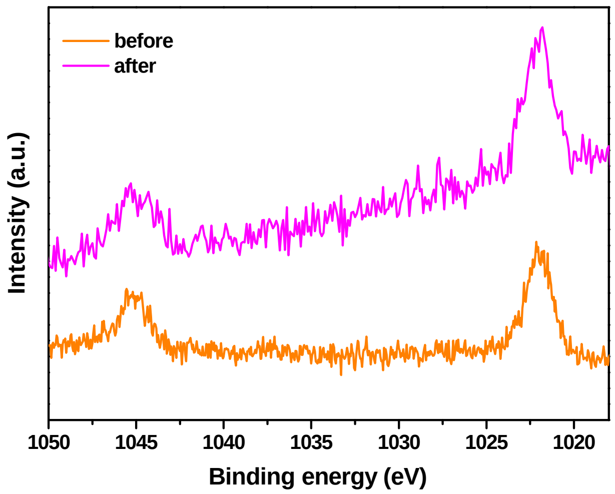

Supplementary Figure 23. Zn 2p XPS of PtZn/HNCNT before and after reaction.

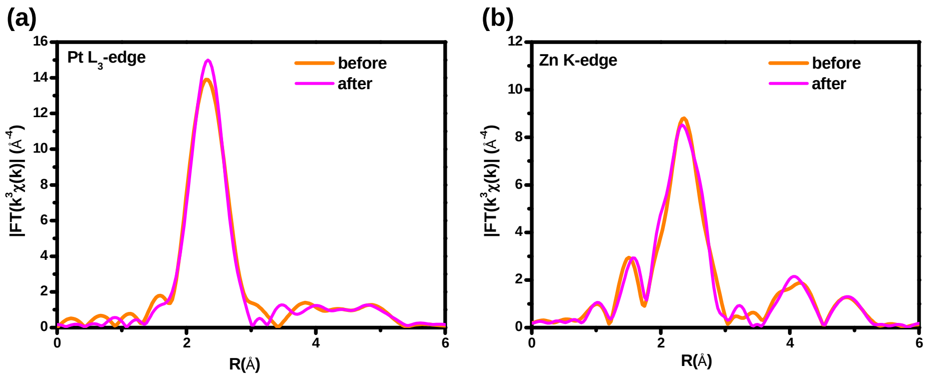

Supplementary Figure 24. The FT-EXAFS at the (a) Pt L_3-edge and (b) Zn K-edge of PtZn/HNCNT before and after the reaction.

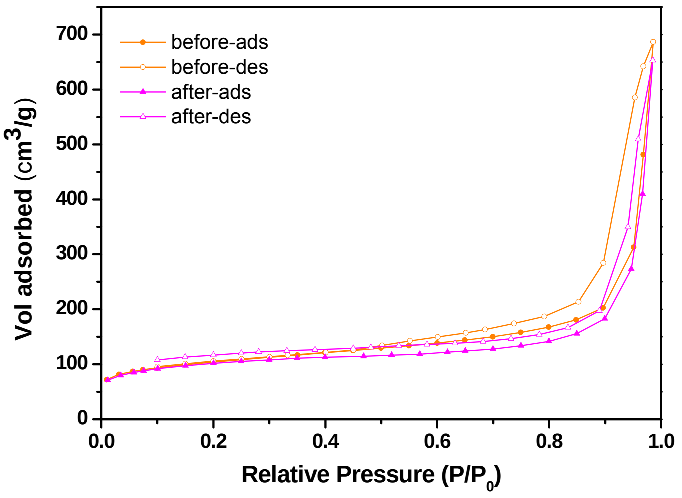

Supplementary Figure 25. N_2 adsorption and desorption isotherm of PtZn/HNCNT before and after the reaction.

Comment 4. *Authors need to provide more information about pretreatment after synthesis and before activity measurements and characterizations. Pretreatment information is important because the state of catalysts becomes different depending on pretreatment. For example, there was no description about the pretreatment done before catalytic activity measurement for 4-nitrophenylacetylene hydrogenation, XPS, and XAS measurements. If no pretreatment was done on catalysts before measurements, then characterizations on catalysts before and after reactions become more important.*

Response: We sincerely appreciate your valuable comment. In our experiment, there is no pretreatment after synthesis and before activity measurements and characterization. The samples are stored in glass sample bottle with caps at room temperature. As you suggested, more characterizations (such as XRD, XAS, XPS, N_2 sorption, etc.) of the catalysts before and after reactions (Supplementary Figure 21-25) are added in the revised manuscript. A discussion about the recycled catalysts is added in page 9.

"After reaction, there was nearly no change in the metal content (0.82% before and 0.80% after reaction), XRD, XPS, XAS, and N_2 sorption for PtZn/HNCNT (Supplementary Fig. 21-25)."

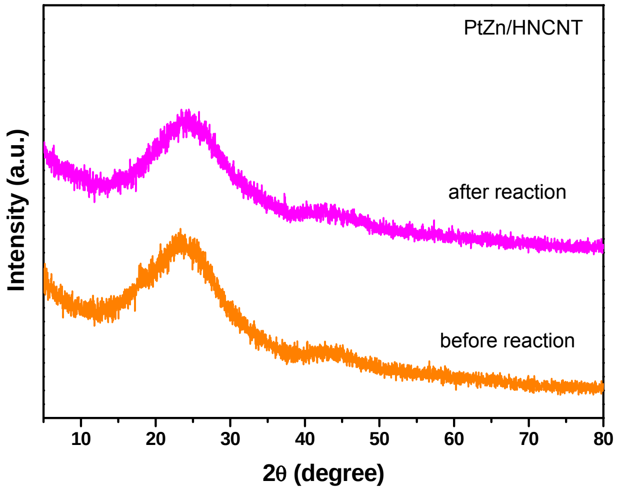

Supplementary Figure 21. XRD patterns of PtZn/HNCNT before and after the reaction.

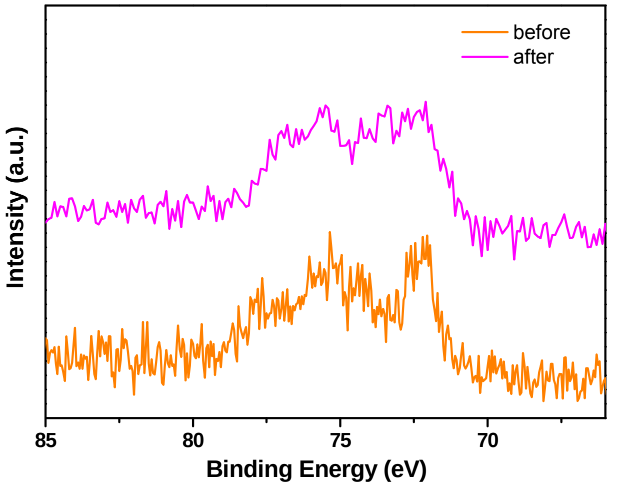

Supplementary Figure 22. Pt 4f XPS of PtZn/HNCNT before and after reaction.

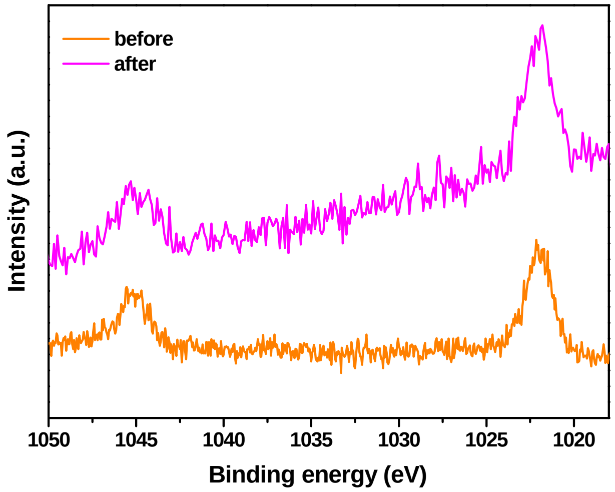

Supplementary Figure 23. Zn 2p XPS of PtZn/HNCNT before and after reaction.

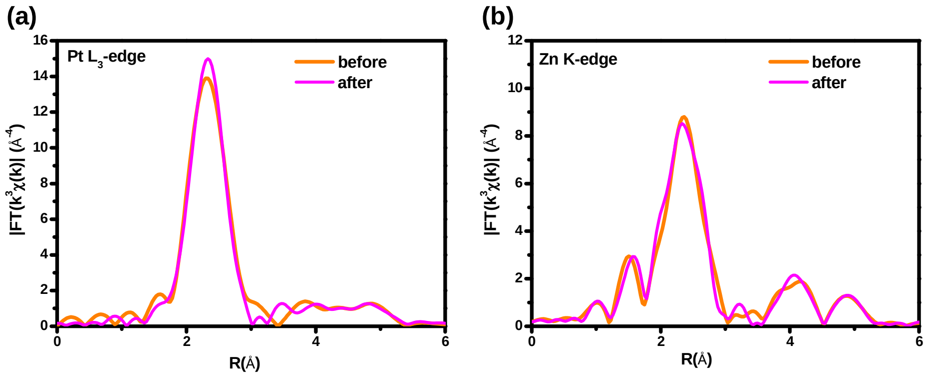

Supplementary Figure 24. The FT-EXAFS at the (a) Pt L_3-edge and (b) Zn K-edge of PtZn/HNCNT before and after the reaction.

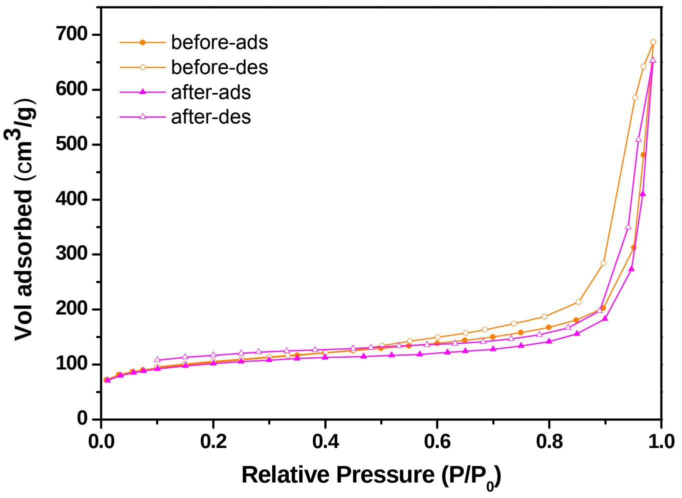

Supplementary Figure 25. N_2 adsorption and desorption isotherm of PtZn/HNCNT before and after the reaction.

Comment 5. *In the experimental section, very limited information on characterizations was also provided. For XPS characterization, the binding energies need to be calibrated based on a reference point (typically the C1s peak) to compare the shift in binding energies but there is no description on calibration. In addition, I am not quite sure whether XPS was performed in situ or ex situ. Besides XPS, more detailed description on characterizations needs to be included.*

Response: Thanks very much for your valuable suggestion. The XPS binding energies was calibrated based on C 1s peak and XPS was performed ex situ. More detailed description (labeled in blue color) on characterizations was provided in the Experimental part in Page 16 of the revised manuscript.

"Characterizations. Transmission electron microscopy (TEM) images were taken on a Hitachi HT7700 transmission electron microscope working at 100 KV. The high-resolution TEM, HAADF-STEM images, and the corresponding Energy-dispersive X-ray (EDX) mapping were recorded by a JEOL JEM-2100F high resolution transmission electron microscope operating at 200 kV. Aberration-corrected HAADF-STEM images are taken on a JEOL JEM-ARM200F TEM/STEM with a spherical aberration corrector working at 300 kV. Powder X-ray diffraction patterns were measured with a Bruker D8 with Cu Kα radiation (λ =1.5406 Å). The X-ray photoelectron spectroscopy (XPS) was measured ex situ by a PHI Quantera SXM system under 3.1 x 10^{-8} Pa using Al^{+} radiation at room temperature. The binding energies were calibrated by referring C 1s peak to 284.8 eV. The metal content was determined by inductively coupled plasma optical emission spectroscopy (ICP-OES) on Thermo Fisher IRIS Intrepid II."

Comment 6. *Please add the following reference studying the selective 4-nitrophenylacetylene hydrogenation reaction over Pt/TiO_2 catalyst by modifying the exposed Pt facets: Corma et al. J. AM. CHEM. SOC. 2008, 130, 8748–8753. This work tells us why the formation of Pt single atoms*

is important for the selective hydrogenation of 4-nitrophenylacetylene.

Response: We are very grateful for your valuable comment. This paper is added as reference 15 in the revised manuscript.

"15 Corma, A., Serna, P., Concepción, P. & Calvino, J. J. Transforming Nonselective into Chemoselective Metal Catalysts for the Hydrogenation of Substituted Nitroaromatics. *J. Am. Chem. Soc.* **130**, 8748-8753 (2008)."

Reviewer #2:

The present study reported the high catalytic activity and selectivity of PtZn alloy supported on hollow nitrogen-doped carbon nanotubes toward the hydrogenation of 4-nitrophenylacetylene.The single Pt atom embedded on the PtZn surface was identified as the active site in cooperation with the Zn site. I cannot recommend the publication of the manuscript in the present form. Major revisions considered the following comments should be made.

Comment 1. *The enhanced catalytic performance of monometallic by forming alloys has been reported extensively before, e.g. Pt alloys in electrochemistry, Cu alloys for CO_2 activation, and Pd alloys for hydrogenation. What is new for the present PtZn as compared to the previous?*

Response: Thank you very much for this comment. There are three novel points for the present PtZn work as compared to the previous.

Firstly, we provided a new sacrificial template strategy to prepare PtZn intermetallic nanoparticles with small sizes in this work. In this strategy, ZnO works as the zinc source as well as the template for hollow nitrogen doped carbon nanotubes. The average particle size in this method is 2.4 nm. This work provides a novel method to synthesize small sized PtZn intermetallic nanoparticles.

Secondly, we found that PtZn intermetllic nanoparticles is highly selective in the hydrogenation of 4-nitrophenylacetylene, showing hydrogenative preference to the nitro group in the presence of highly reducible alkynyl group. This work gives a new example of PtZn intermetallic compounds as effective catalysts in selective catalytic reactions.

Thirdly, we proposed a new insight to understand intermetallic compounds from the view of single atomic sites. In the PtZn intermetallic nanoparticles, contiguous Pt atoms are isolated into single atoms and Pt-Zn bridge sites are formed, and the neighbouring Zn atoms have two roles. One is to isolate the contiguous Pt atoms, the other is to form Pt-Zn bridge sites to assist the adsorption of nitro group and facilitate the hydrogenation on nitro group kinetically.

Comment 2. *Is there any surface segregation of Pt from the bulk to the PtZn surface and formation of Pt overlayer?*

Response: We sincerely appreciate your valuable suggestion. It is generally agreed that Pt surface segregation in Pt alloy catalysis plays a critical role in improving the ORR performance. Pt-Fe, Pt-Co and Pt-Ni alloys have been determined to show better ORR catalytic performance than pure

Pt if the Pt atoms segregated to the outermost surface and formed an overlayer on these alloys (*Science*, **315**, 493-497 (2007); *J. Am. Chem. Soc.*, **126**, 4717-4725 (2004); Surf. Sci. **605**, 1577–1582 (2011); *J. Phys. Chem. B*, **106**, 4181–4191 (2002); *J. Phys. Chem. B*, **106**, 11970-11979 (2002)). In this context, we modeled the PtZn(02$\bar{2}$) surface using a periodic slab model, in which there are five layers of atoms in the [02$\bar{2}$] direction. The segregation structures were achieved by exchanging Zn atom at the first layer and Pt atoms at the second layer. Therefore, the topmost layer is fully covered by Pt atoms. In the same way, the structure with exchanging Zn atom at the second layer and Pt atoms at the third layer was also considered. The geometries and relative energies were shown in Figure R2-2. From Figure R2-2, we can determined that the considered segregation structures are less stable than the intrinsic PtZn(02$\bar{2}$) surface.

Meanwhile, we did not observe the segregation structures through our experimental results. If the segregation occurs, there should be a clear interface between Pt overlayer and PtZn surface from AC HAADF STEM. However, we only observe uniform atomic arrangements. Therefore, we think that no surface segregation of Pt from the bulk to the PtZn surface takes place.

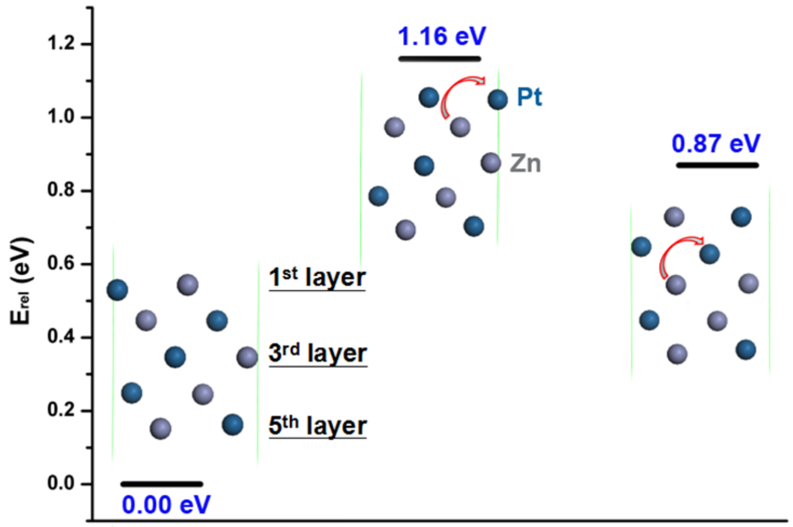

Figure R2-2. Structures and relative energies of PtZn(02$\bar{2}$) surface and the considered segregation configurations with Pt monolayer.

Comment 3. *Is the carbon support play any role? The DFT modeling completely ignored the effect of support and the low-coordinated sites in the PtZn nanoparticles. Is it reasonable for comparison with experiment?*

Response: Thanks very much for your valuable suggestion. As shown in Figure 3b, the carbon support, HNCNT, was also tested as a catalyst for this hydrogenation of 4-nitrophenylacetylene. There was nearly no reaction. Therefore, we think that the carbon support didn't play any role in the catalytic process.

In theoretical calculations, the supports were included in the modeling only if the sites at the interface between the metal particles and the support were relevant for the reaction. For catalysis

reactions contain small-size reactants and small-size metal nanoparticles (for example, the extreme case-single atom catalysis), the discussion of support effect might be necessary (*J. Am. Chem. Soc.*, **139**, 6190-6199 (2017); *J. Am. Chem. Soc.*, **138**, 10467-10476 (2016); *J. Am. Chem. Soc.*, **140**, 7407–7410 (2018)). Generally, the carbon support is not considered in theoretical modeling. For instance, the commercial carbon supported 20 wt % platinum Pt/C ORR catalysis is simplified as Pt(111) surface, which is a widely used theoretical model. In one previous work, the carbon support in sub-4 nm PtZn intermetallic nanoparticles catalyst was also neglected in modeling (*J. Am. Chem. Soc.*, **139**, 4762-4768 (2017)).

To confirm the reasonableness of ignoring the effect of support, a graphene supported 4-layers-Pt(111)/5-layers-PtZn($02\bar{2}$) interfaces were constructed. The interface structures are shown in Figure R2-3a. Due to the better ductility of Pt/PtZn, strain is applied on metal/alloy surfaces to reduce the interface mismatch. The mean absolute strains for 4-layers-Pt(111)/graphene and 5-layers-PtZn($02\bar{2}$)/graphene structures are only 0.97 % and 1.09 %, respectively. These tiny strains ensure the electronic properties are not perturbed by the applied strains notably (*Science*, **354**, 1410-1414 (2016); *Angew. Chem. Int. Ed.*, **56**, 11971-11975 (2017)). The 4-layers-Pt(111)/graphene and 5-layers-PtZn($02\bar{2}$)/graphene structures contain 20 and 90 atoms, respectively. The charge populations of the metal or alloy layers close to the carbon support change significantly, however the charge populations of the outmost layer does not change significantly, as shown in Figure R2-3b. Meanwhile, calculating the projected density of states (PDOS) for *d*-band of outmost metal atoms indicated that the carbon support does not change the electronic properties of outmost layer significantly (Figure R2-3c).

It is no doubt that the low-coordinated sites such as vertexes, edges and steps would play an important role in the processes such as hydrogen adsorption and diffusion, adsorption of 4-nitrophenylacetylene and its corresponding intermediates and the entire hydrogenation reaction of 4-nitrophenylacetylene. Several slab models which contain individual type of low-coordinated sites (vertexes, edges, and steps) exclusively are needed to systematically discuss the effect of each low-coordinated site (*J. Am. Chem. Soc.*, **130**, 370-381 (2008)). For example, Metal(321) surface is usually chosen to mimic step edges of nanoparticels with (111) terraces. Au(321) surface was chosen as a model to describe the defect-rich surface of gold nanoparticels (*J. Phys. Chem. C*, **119**, 9215-9226 (2015); *Chem. Phys. Lett.*, **525**, 87-91 (2012); *J. Phys. Chem. C*, 10.1021/acs.jpcc.8b06676 (2018)). Pt(321) and PtZn(321) surfaces were constructed in the similar way to determine the hydrogen and 4-nitrophenylacetylene adsorption configurations. The most stable adsorption site of hydrogen atom on Pt(321) and PtZn(321) is the bridge site of Pt atoms on 1st and 2nd layer (Figure R2-3d, Figure R2-3e), which is different form the hollow site on Pt(111) and Pt-Zn bridge site on PtZn($02\bar{2}$) surface. Meanwhile, the step edges reinforces the distortion of 4-nitrophenylacetylene, since the size of 4-nitrophenylacetylene molecule is long and would span several steps on the surface (Figure R2-3f). The intensive distortion on alkynyl or nitro group would definitely change the relative reaction activity, but that is beyond the scope of the present work.

In this context, only the defect-free surface is considered due to the complexity and diversity of the intractable computational demands to low-coordinated sites.

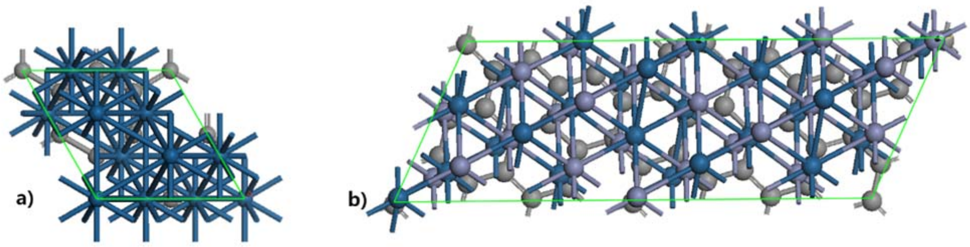

Figure R2-3a. The topview of (a) Pt(111)/graphene interface and (b) PtZn$(02\bar{2})$/graphene interface

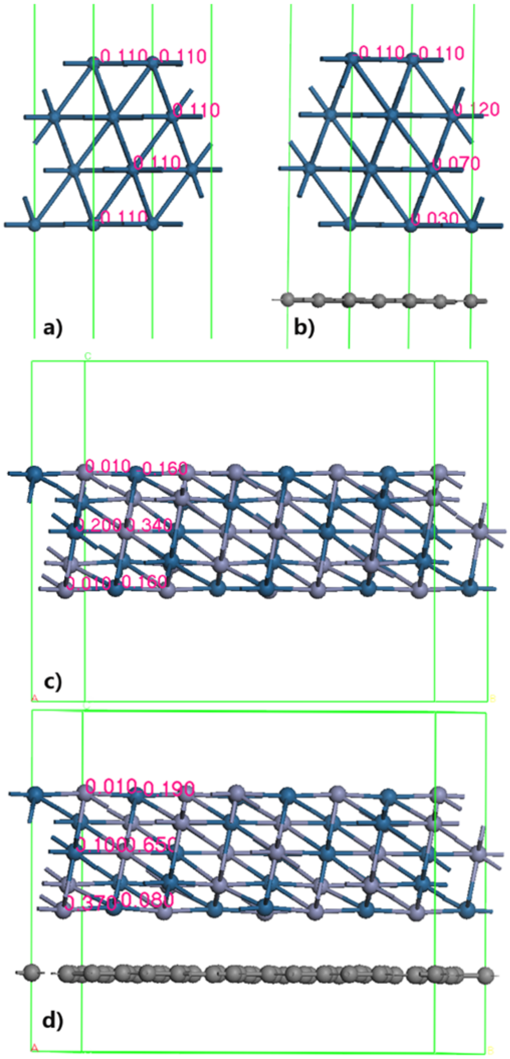

Figure R2-3b. The geometries of (a) Pt(111) surface, (b) Pt(111)/graphene interface, (c) PtZn$(02\bar{2})$ surface and (d) PtZn$(02\bar{2})$/graphene interface. The Mulliken charge populations were labeled.

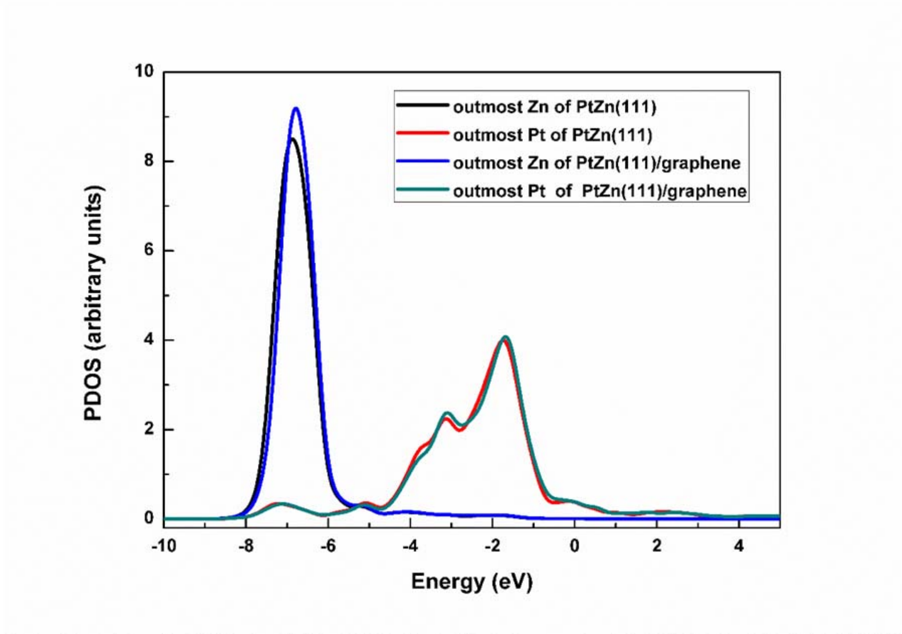

Figure R2-3c. The projected density of states (PDOS) for *d*-band of outmost metal atoms of PtZn(111) surface and PtZn(02$\overline{2}$)/graphene interface.

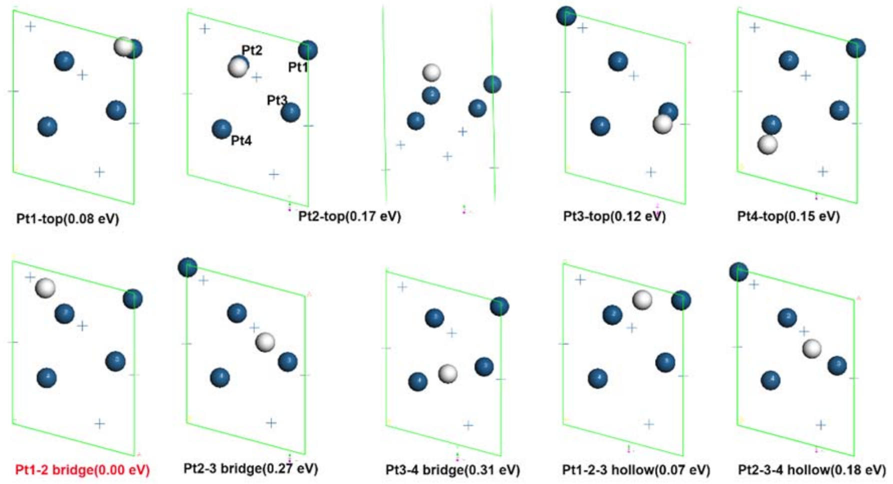

Figure R2-3d. The configurations of hydrogen atoms on Pt(321) surface.

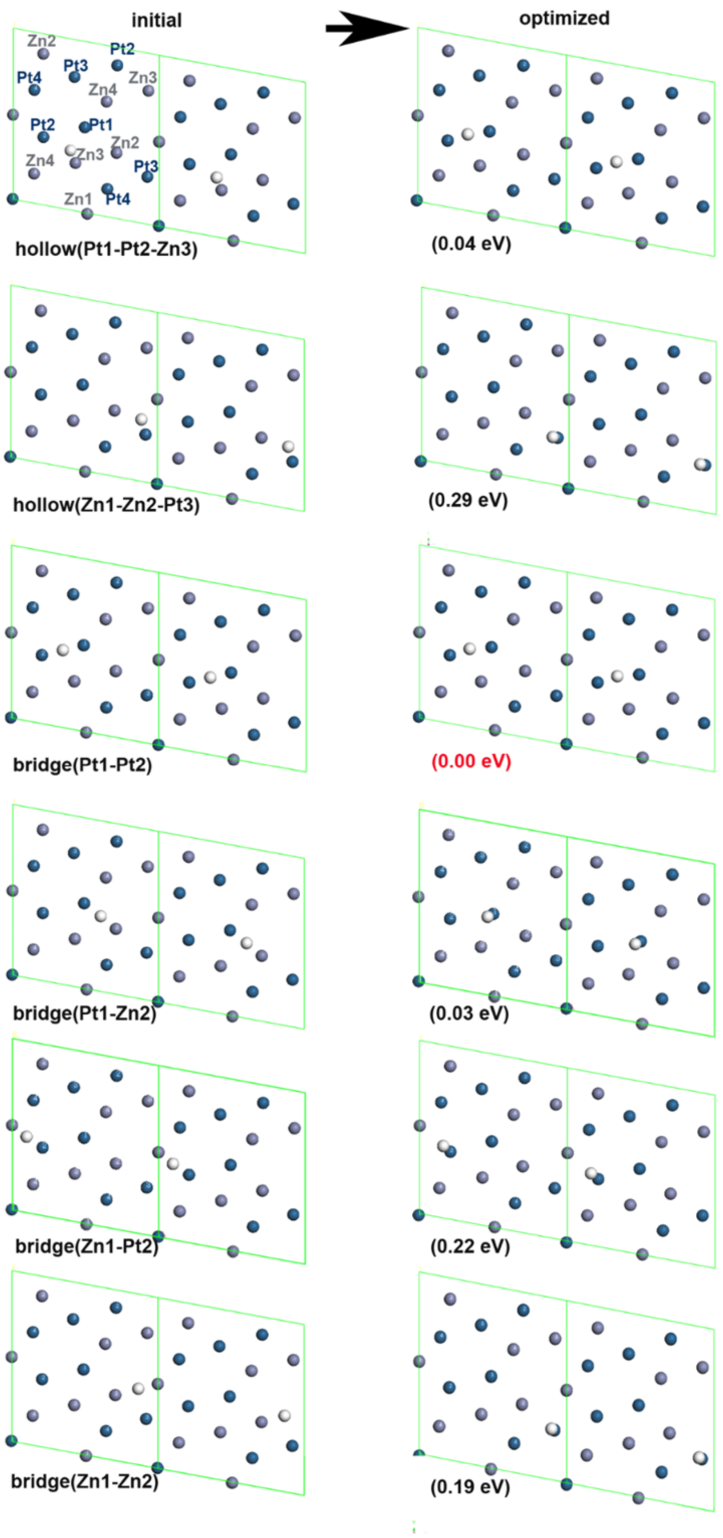

Figure R2-3e. The configurations of hydrogen atoms on PtZn(321) surface.

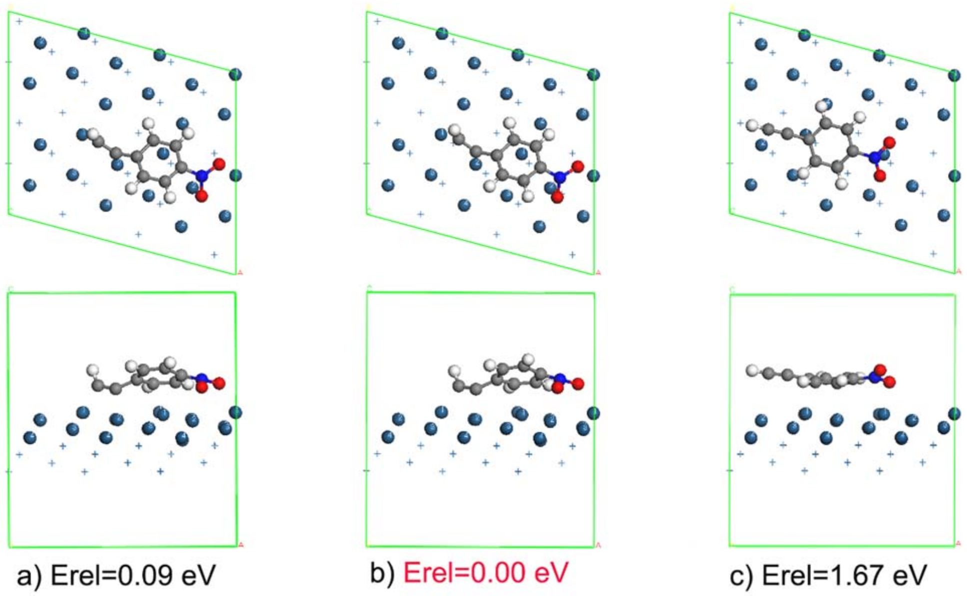

Figure R2-3f. The top and side view of considered 4-nitrophenylacetylene adsorption configurations on Pt(321) surface.

Comment 4. *DFT calculations only considered the first hydrogenation step to correlate with the experimental observation. This is too simplified. A full reaction path way toward the experimental product should be considered, which improves the mechanistic understanding and makes the connection with the measurement.*

Response: Thanks for your valuable suggestion. Actually, we considered the full reaction path at first. The reactant molecule contains two functional groups: alkynyl and nitro. A full hydrogenation reaction path from CH-C-C_6H_4-NO_2 to CH_3-CH_2-C_6H_4-NH_2 involves 10 hydrogenation steps. In each steps (at least the in the initial several steps), hydrogenation reaction could occur on alpha-C, beta-C and the oxygen sites (In PtZn system, the two oxygen atoms in nitro group at the alloy surface are asymmetric. Hence the hydrogenation reaction on each oxygen atoms should be considered one by one.). In this sense the branches in the entire reaction path contains grow explosive (Figure R2-4a**)**. It is burdensome to investigate the entire reaction path.

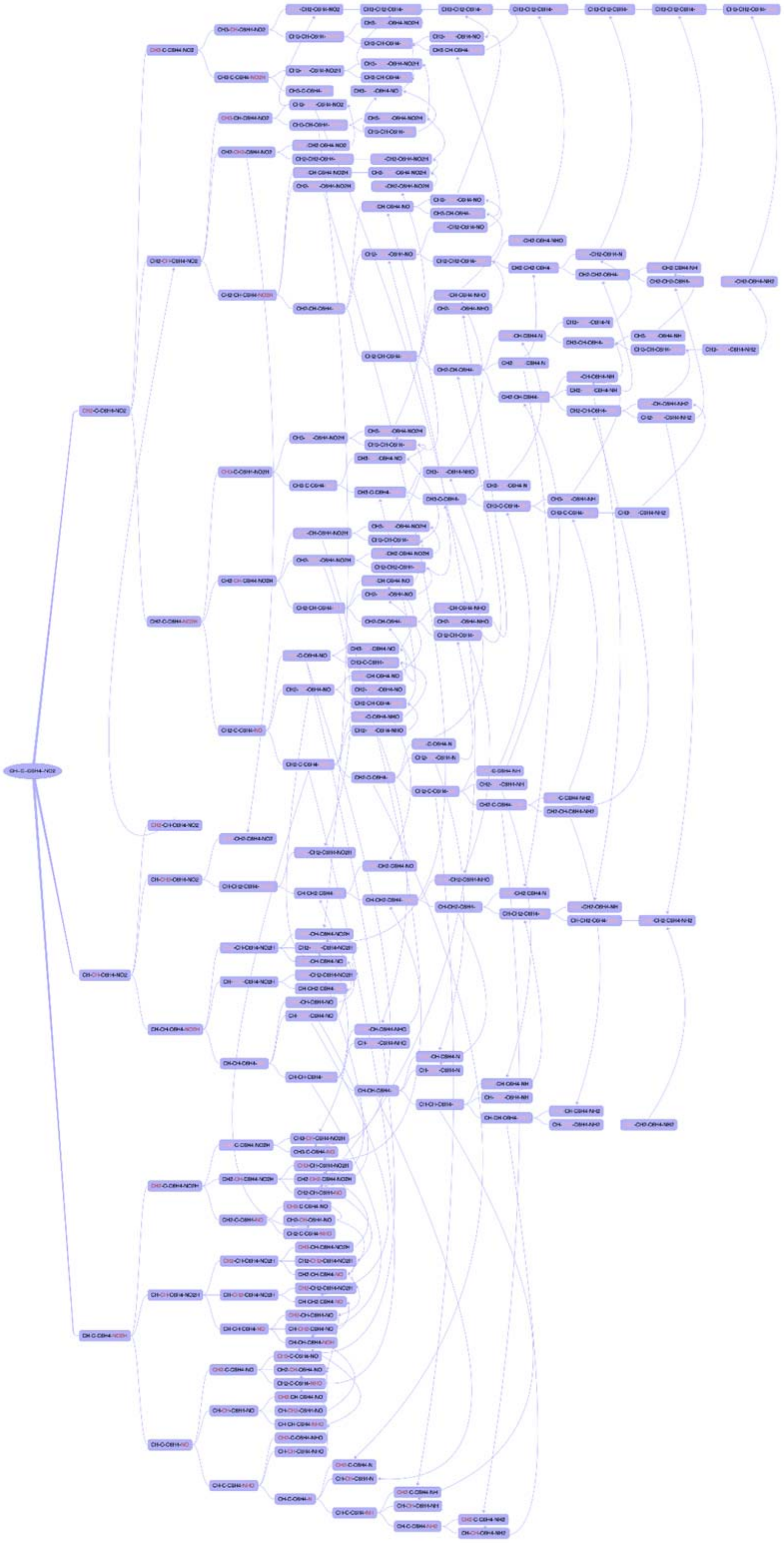

Figure R2-4a. The proposed reaction pathway for hydrogenation of CH-C-C_6H_4-NO_2 on the considered surface. Based on our calculation, the C_mH_n-C_6H_4-NHO is more stable than C_mH_n-C_6H_4-NOH for each considered (m, n). Hence in this pathway, only C_mH_n-C_6H_4-NHO is considered. The hydrogenation sites are marked in red.

In this sense, we determined that the comparing the reaction barriers of the initial hydrogenation step on both alkynyl and nitro on Pt and PtZn alloy surface is a good point to emphasize the selectivity in the context. For example, the reaction barrier of hydrogenation on alkynyl is almost twice of it on nitro for PtZn surface, indicating that the higher barrier (201 KJ/mol) is difficult to trigger the subsequent reactions.

In the revised version, we have tried our best to consider the full reaction path for both Pt and PtZn systems. The thermochemistry-only potential energy pathways were constructed (*J. Phys. Chem. C*, 10.1021/acs.jpcc.7b11629 (2018); *J. Phys. Chem. B*, 122, 432–443 (2018)). For reations on Pt(111) surface, the 1st hydrogen atom was attached on CH, C and NO_2 sites, respectively. Based on the reaction energy calculated, the most thermochemistical favorable intermediate is filtered and selected as the reactant for the next hydrogenation step. The simplified hydrogenation reaction pathway and the corresponding reaction energies of CH-C-C_6H_4-NO_2 on the Pt(111) surface is shown in Supplementary Figure 30 and Supplementary Figure 31. In the first two hydrogenation steps, the alkynyl group is the favorable target, followed by the formation of vinyl group (intermediate **3a** in Fig. 3). The vinyl group does not achieve ethyl till the 10th hydrogenation step, meanwhile, the last hydrogenation step is endothermic. The long-lasting unsaturated C_2H_4- group should be the primitive intermediate for the formation of CH_3-CO-C_6H_4-NO_2 (**3c** in Fig 3.) and CH_3-CH_2-C_6H_4-NO_2 (**3b** in Fig 3.)

For PtZn surface, the alkynyl and nitro group stay on the different surface patterns, where alkynyl and nitro are close to Pt and Zn, respectively. To consider the hydrogenation reaction pathway, firstly, one hydrogen atom was attached on CH, C and NO_2 sites, respectively. Based on the reaction energy calculated, the most thermochemistical favorable alkynyl part and nitro part intermediates are filtered and selected as the reactant for the next hydrogenation step. Even after such filtering, the reaction pathway is still too complex as shown in Supplementary Figure 36.

In this part, an alternative route was considered to correlate with the experimental observation. The overall direct hydrogenation paths has been identified as $PhNO_2$*→PhNOOH*→PhNO*→PhNHO*→PhN*→PhNH*→$PhNH_2$* (*Chem Eur J.*, **21**, 19016-19027 (2015); *Angew Chem Int Ed.*, **56**, 11971-11975 (2017)). The barriers of hydrogenation reactions on alkynyl group for CH-C-C_6H_4-NO_2/NOOH/NO/NHO/N/NH/NH_2 on alloy surface were calculated. The calculation results are shown in Supplementary Figure 35. We can find that every reaction needs to climb over a barrier higher than 200 KJ/mol, which is difficult to trigger the subsequent hydrogenation reactions on the alkynyl group. We considered that the such high reaction barrier on alkynyl part is the essential reason for the high selectivity on PtZn surface.

The DFT part is revised in page 12-13.

"Hydrogenation processes could potentially occur on both nitro and alkynyl groups. To reveal the chemoselective hydrogenation process, the first elementary reaction steps on both groups were investigated. Hydrogen atom prefers to adsorb on the fcc-hollow site on Pt surface (Supplementary Fig. 29). Over Pt(111) surface, the energy barriers in the first step for hydrogenation on nitro group and alkynyl group are 239 and 117 kJ/mol (Fig. 4a), respectively, indicating that the hydrogenation process on nitro group is unfavorable kinetically. The thermochemistry-only potential energy pathways were constructed (Supplementary Fig. 30 and Supplementary Fig. 31)[37,38]. In the first two hydrogenation steps, the alkynyl group is the favorable target, followed by the formation of vinyl group (intermediate **3a** in Fig. 3). The vinyl

group does not achieve ethyl till the 10th hydrogenation step, meanwhile, the last hydrogenation step is endothermic. The long-lasting unsaturated C_2H_4- group should be the primitive intermediate for the formation of CH_3-CO-C_6H_4-NO_2 (**3c** in Fig. 3) and CH_3-CH_2-C_6H_4-NO_2 (**3b** in Fig. 3). Over PtZn(02$\bar{2}$) surface, hydrogen atom prefers to adsorb on the Pt-Zn bridge site (Supplementary Fig. 32). The relative vertical distances between H* and its hydrogenation target atom (oxygen in nitro group or carbon atom in alkynyl group) are comparable (0.897 vs 0.978 Å). For 4-NPA@PtZn(02$\bar{2}$), hydrogenation on nitro group is along the hydrogen atom diffusion path from Pt-Zn(bridge) to Zn-Pt(bridge) (Supplementary Fig. 33). The diffusion barrier is 0.30 eV (28.8 kJ/mol). Meanwhile, the hydrogenation on alkynyl group is along the perpendicular direction. Hydrogen diffusion along this path is unfavorable (diffusion barrier is 1.48 eV). Interestingly, the energy barriers in the first step for hydrogenation on nitro group and alkynyl group are 114 and 201 kJ/mol (Fig. 4b), respectively, indicating that the hydrogenation process on alkynyl group is unfavorable kinetically. Based on the direct hydrogenation path nitro group, the barriers of first hydrogenation step on alkynyl group for CH-C-C_6H_4-NOOH/NO/NHO/N/NH/NH_2 on alloy surface were also calculated (Supplementary Fig. 34 and Fig. 35). We can find that every reaction needs to climb over a barrier higher than 200 kJ/mol, which is difficult to trigger the subsequent hydrogenation reactions on the alkynyl group. We considered that the such high reaction barrier on alkynyl part is the essential reason for the high selectivity on PtZn surface."

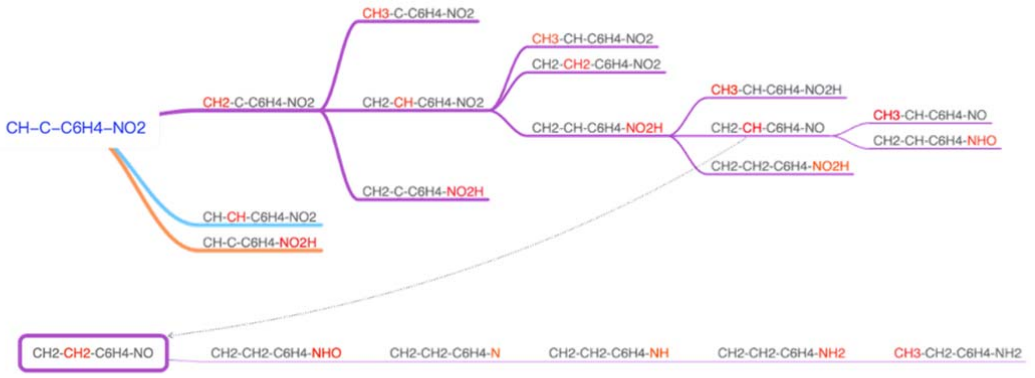

Supplementary Figure 30. The simplified hydrogenation reaction pathway of CH-C-C_6H_4-NO_2 on the Pt(111) surface. The hydrogenation sites are marked in red.

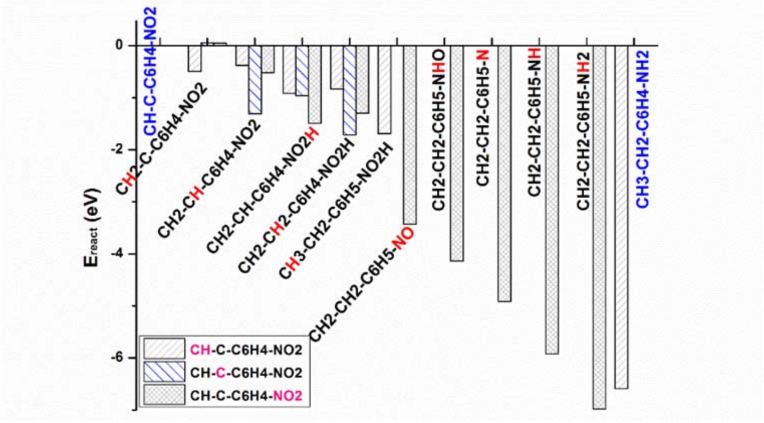

Supplementary Figure 31. The reaction energies of hydrogenation reaction pathway of CH-C-C_6H_4-NO_2 on the Pt(111) surface. The hydrogenation sites are marked in red.

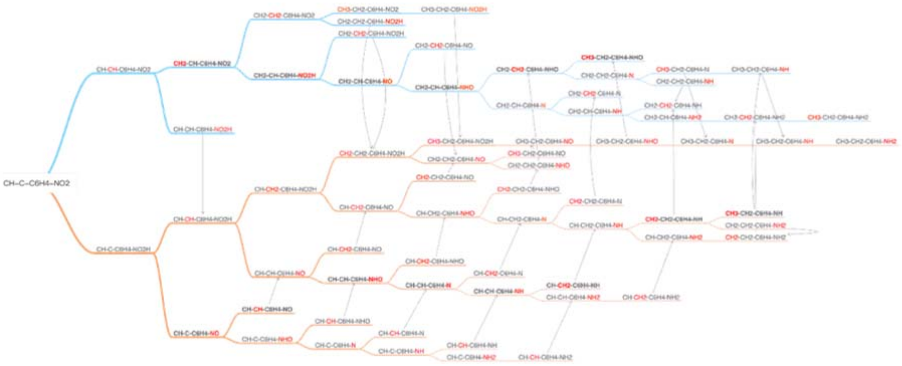

Supplementary Figure 34. The simplified hydrogenation reaction pathway of CH-C-C_6H_4-NO_2 on the PtZn(02$\bar{2}$) surface. The hydrogenation sites are marked in red.

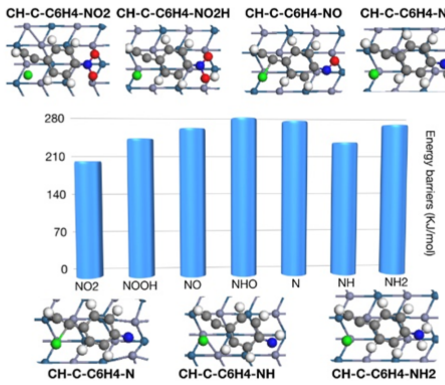

Supplementary Figure 35. The calculated energy barriers of hydrogenation reactions on alkynyl group for CH-C-C_6H_4-NO_2/NOOH/NO/NHO/N/NH/NH_2 on alloy surface. The insets show the corresponding geometries of transition states.

Comment 5. *The mechanistic understanding of the different behavior in selectivity between Pt and PtZn is missing. Or why can the formation of alloy change the selectivity? In-depth analysis from DFT calculations and/or well-defined characterization may help.*

Response: Thanks very much for your valuable suggestion. In our reply to reviewer 2 comment 4, we tried to understand the selectivity of PtZn from the high reaction barrier on alkynyl part. Meanwhile, we suggest that the different behavior in selectivity between Pt and PtZn mainly originate from the adsorption configuration of CH-C-C_6H_4-NO_2 on the two surfaces. As shown in Supplementary Figure 30-31, alkynyl group adsorbed on Pt surface with a 3-fold hollow mode, which is suggested as the energetically favored adsorption configuration for acetylene on Pt(111) surface (*J. Phys. Chem. B*, **107**, 12287-12295 (2003)), meanwhile the nitro group is far away from the Pt(111) surface due to the electrostatic repulsion between the negative charged Pt and negative charged nitro group. For this configuration, the hydrogenation reaction on alkynyl part is favorable. However, PtZn alloy leads an alternate Pt-Zn surface, where Pt is negative charged and Zn is positive charged. The nitro group perfers to approach the Zn atoms to make the configuration stable, meanwhile, the surface pattern could not accommodate alkynyl group a Pt-Pt-Pt 3-fold hollow site any more. Hence, only one carbon atom in alkynyl group anchored on

the alloy surface. For this configuration, the hydrogenation reaction on nitro part is favorable. In summary, it is the charge population induced seesaw-like adsorption orientation lead to the relative selectivity of the two surfaces. An in-depth analysis of DFT calculations are added in page 12-13.

“Hydrogenation processes could potentially occur on both nitro and alkynyl groups. To reveal the chemoselective hydrogenation process, the first elementary reaction steps on both groups were investigated. Hydrogen atom prefers to adsorb on the fcc-hollow site on Pt surface (Supplementary Fig. 29). Over Pt(111) surface, the energy barriers in the first step for hydrogenation on nitro group and alkynyl group are 239 and 117 kJ/mol (Fig. 4a), respectively, indicating that the hydrogenation process on nitro group is unfavorable kinetically. The thermochemistry-only potential energy pathways were constructed (Supplementary Fig. 30 and Supplementary Fig. 31)[37,38]. In the first two hydrogenation steps, the alkynyl group is the favorable target, followed by the formation of vinyl group (intermediate **3a** in Fig. 3). The vinyl group does not achieve ethyl till the 10th hydrogenation step, meanwhile, the last hydrogenation step is endothermic. The long-lasting unsaturated C_2H_4- group should be the primitive intermediate for the formation of CH_3-CO-C_6H_4-NO_2 (**3c** in Fig. 3) and CH_3-CH_2-C_6H_4-NO_2 (**3b** in Fig. 3). Over PtZn(02$\bar{2}$) surface, hydrogen atom prefers to adsorb on the Pt-Zn bridge site (Supplementary Fig. 32). The relative vertical distances between H* and its hydrogenation target atom (oxygen in nitro group or carbon atom in alkynyl group) are comparable (0.897 vs 0.978 Å). For 4-NPA@PtZn(02$\bar{2}$), hydrogenation on nitro group is along the hydrogen atom diffusion path from Pt-Zn(bridge) to Zn-Pt(bridge) (Supplementary Fig. 33). The diffusion barrier is 0.30 eV (28.8 kJ/mol). Meanwhile, the hydrogenation on alkynyl group is along the perpendicular direction. Hydrogen diffusion along this path is unfavorable (diffusion barrier is 1.48 eV). Interestingly, the energy barriers in the first step for hydrogenation on nitro group and alkynyl group are 114 and 201 kJ/mol (Fig. 4b), respectively, indicating that the hydrogenation process on alkynyl group is unfavorable kinetically. Based on the direct hydrogenation path nitro group, the barriers of first hydrogenation step on alkynyl group for CH-C-C_6H_4-NOOH/NO/NHO/N/NH/NH_2 on alloy surface were also calculated (Supplementary Fig. 34 and Fig. 35). We can find that every reaction needs to climb over a barrier higher than 200 kJ/mol, which is difficult to trigger the subsequent hydrogenation reactions on the alkynyl group. We considered that the such high reaction barrier on alkynyl part is the essential reason for the high selectivity on PtZn surface.”

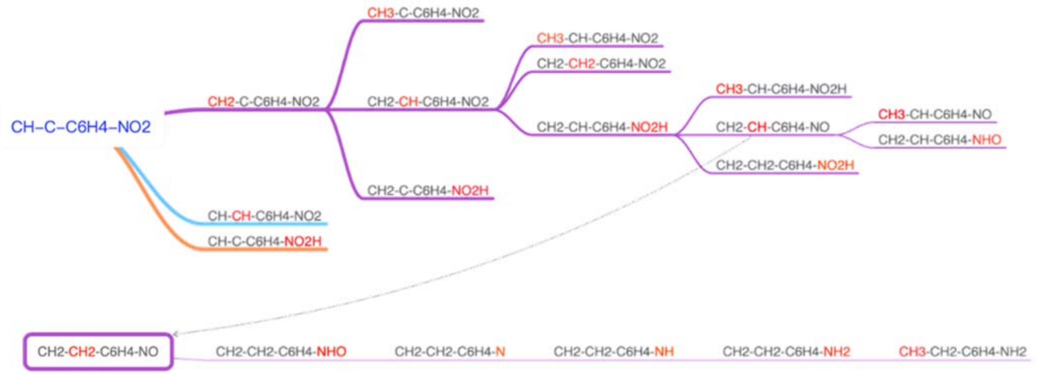

Supplementary Figure 30. The simplified hydrogenation reaction pathway of CH-C-C_6H_4-NO_2 on the Pt(111) surface. The hydrogenation sites are marked in red.

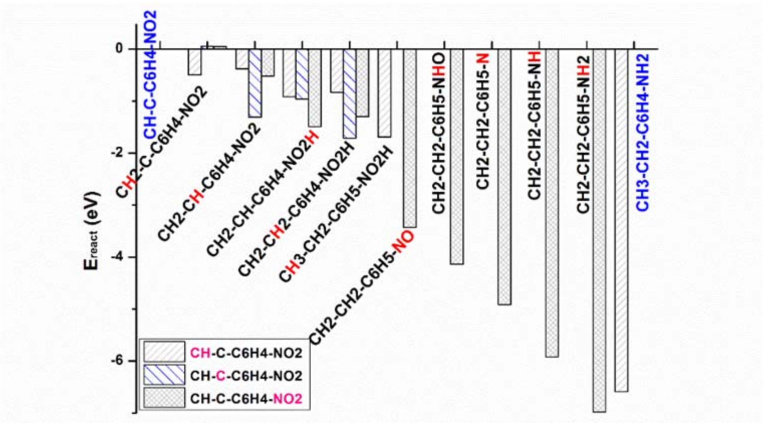

Supplementary Figure 31. The reaction energies of hydrogenation reaction pathway of CH-C-C_6H_4-NO_2 on the Pt(111) surface. The hydrogenation sites are marked in red.

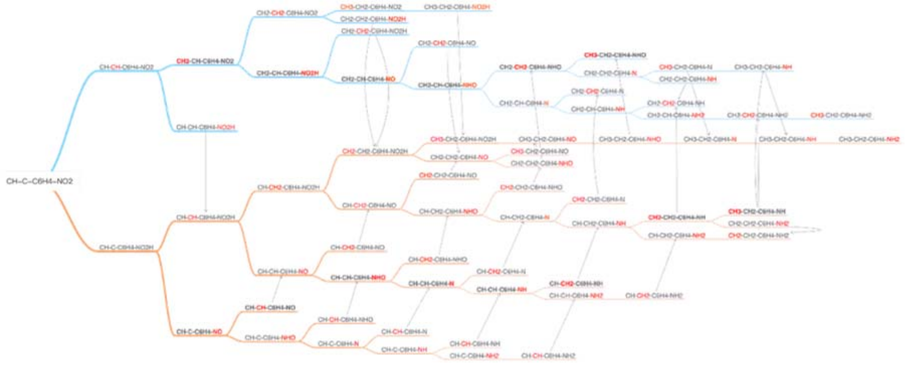

Supplementary Figure 34. The simplified hydrogenation reaction pathway of CH-C-C_6H_4-NO_2 on the PtZn(02$\bar{2}$) surface. The hydrogenation sites are marked in red.

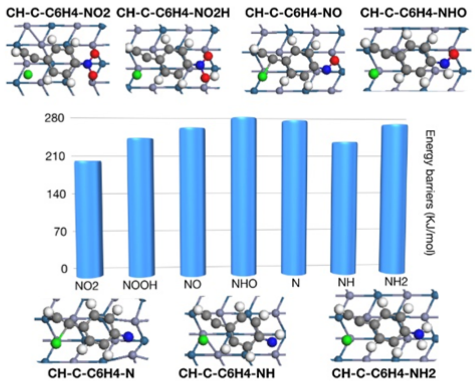

Supplementary Figure 35. The calculated energy barriers of hydrogenation reactions on alkynyl group for CH-C-C_6H_4-NO_2/NOOH/NO/NHO/N/NH/NH_2 on alloy surface. The insets show the corresponding geometries of transition states.

Reviewer #3:

The manuscript investigates the use of an intermetallic Pt-Zn catalyst for the selective hydrogenation of a nitro-compound (nitrophenylacetylene). The authors prepare the catalyst via a sacrificial template method where the ZnO nanorods are first coated with a polymer (PDA) and then deposited with a Pt precursor ($Pt(OH)_4$). Reduction of the obtained material at high temperature leads to the final catalyst featuring Pt-Zn nanoparticles on a ZnO support. The material was characterized by standard ex situ techniques such as TEM, XPS, XRD, and XAS; catalytic tests were conducted in batch at fixed conditions. Finally, the authors initiated preliminary DFT calculations to rationalize the catalytic performance.

I have several points of concern with this work, rendering it not yet suitable for publication in a top-tier journal.

Comment 1. *From a synthetic point of view, the authors propose that high temperature reduction of $Pt(OH)_4$/ZnO@PDA leads to zinc vapor, 'which assists the contiguous Pt atom isolation'. From my understanding of the thermal evaporation deposition process, while zinc vapor is formed, the Pt is reduced, forming Pt^0, which aggregates/clusters forming nanoparticles, incorporating part of the Zn from the surface. At the same time, the zinc vapor from the atmosphere cannot participate into the process. It does only after cooling down the system, where the vapor redeposits forming a new zinc layer (see Appl Catal B 2006, 62, 144-149). If this interpretation is correct, I would expect a core-shell nanoparticle, with a core of Pt, and a shell of Zn. This brings to additional questions on what is the real active phase and whether catalysis is happing on the zinc oxide surface. Thus, it is fundamental that the authors perform an in depth microscopic and spectroscopic investigation of the catalyst, profiling the full nanoparticle in depth.*

Response: We appreciate your valuable comment.

Firstly, intermetallic PtZn nanoparticles can be formed through reaction of Pt nanoparticles with Zn vapor. One previous work obtained intermetallic PtZn nanoparticles with 3−15 nm diameter by reaction of carbon-supported Pt nanoparticles with Zn vapor at 500 °C for 8 h under flowing nitrogen at atmospheric pressure (*Chem. Mater.*, **21**, 2661–2667 (2009)). In our present work, ZnO are reduced to Zn by H_2, which evaporates at high temperature and interacts with Pt to form PtZn intermetallic nanoparticles. To testify this point, AC HAADF STEM characterization is further taken carefully to see the atomic arrangement of PtZn intermetallic nanoparticles. One AC HAADF STEM image of PtZn nanoparticle viewed along the [010] zone axis is found and added in Supplementary Figure 7. Due to the different atomic density, the heavier Pt atoms (bright) can be distinguished from Zn atoms (darker). The two atomic plane spaces are 0.348 nm and 0.200 nm, corresponding to the (001) and (200) lattice spacing of PtZn intermetallic compound respectively. The atomic arrangement in AC HAADF STEM could match well with the (010) plane generated from the crystal structure of PtZn, further proving that the nanoparticle is PtZn intermetallic compound. What's more, to further prove this point, a sample with higher Pt loading (1%) is prepared using the same method. The diffraction peaks in XRD match well to that of PtZn (JCPDS No 06-0604). Therefore, we think that the PtZn intermetallic compounds instead of core-shell alloys are formed in the PtZn/HNCNT.

PtZn intermetallic nanoparticles are the real active phase. ZnO has been removed after the acid wash step. As seen from the XRD patterns of PtZn/HNCNT in Supplementary Figure 5, no peaks of ZnO appeared, indicating that there is no ZnO remained. Therefore, ZnO cannot be the

catalytic sites.

The characterization part of PtZn/HNCNT is revised in page 6 to further testify that PtZn intermetallic compounds are formed.

"A sample with higher Pt loading (1%) is prepared using the same method. The diffraction peaks in XRD (Supplementary Fig. 6) match well to that of PtZn (JCPDS No 06-0604). To further testify the IMC nature of PtZn particles, AC HAADF STEM measurement was carried out. AC HAADF STEM of one PtZn nanoparticle exhibited clear bright dots, in agreement with the atomic arrangement of ($02\bar{2}$) plane in the PtZn crystal structure (Fig. 1e). The lattice spacing of 0.221 and 0.203 nm matched interplane distances of (111) and (200) planes of intermetallic PtZn (P4/mmm) respectively (Fig. 1f). The perfect agreement testified the nanoparticle is PtZn intermetallic compound. More AC HAADF STEM images are taken to prove that the nanoparticles are PtZn intermetallic compound. As shown in Supplementary Fig. 7, the atomic arrangement of another nanoparticle could match well with the (010) plane of PtZn. Due to the different atomic density, the heavier Pt atoms (bright) can be distinguished from Zn atoms (darker). Therefore, we think that the PtZn intermetallic compounds instead of core-shell alloys are formed in the PtZn/HNCNT."

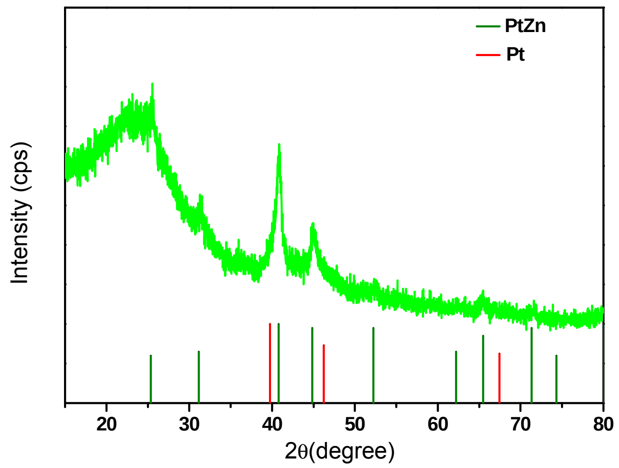

Supplementary Figure 6. XRD of PtZn/HNCNT prepared from 1% Pt/ZnO@PDA.

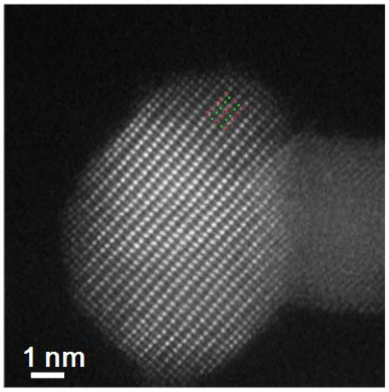

Supplementary Figure 7. AC HAADF STEM image of a PtZn nanoparticle. The red and green spheres represent Pt and Zn atoms, respectively.

Comment 2. *From Figure 1d, it appears that the catalyst has an average particle size of 10 nm. This is however contradicted by Figure 1f, where the average particle size is around 2 nm. A full particle size plot where more than 300 particles are measured is necessary. In this plot, please report error margins.*

Response: Thanks very much for your valuable comment. More AC HAADF STEM images are taken to obtain the particle size plot, which are shown in Supplementary Figure 4. The average diameter was 2.24 nm based on 301 particles, while the error margins is 0.14554. A discussion about this is added in page 6 in the manuscript.

"Aberration-corrected high-angle annular dark-field scanning transmission electron microscope (AC HAADF STEM) images were taken to measure the particle distribution (Supplementary Fig. 4), and the average particle size was 2.24 nm."

To avoid the indistinct, Figure 1d is replaced by a more representative AC HAADF STEM image.

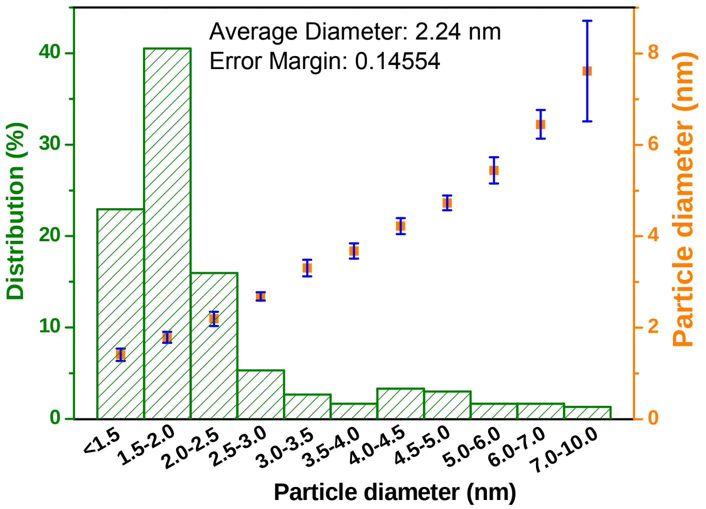

Supplementary Figure 4. PtZn particle size distribution in PtZn/HNCNT determined by AC HAADF STEM images.

Comment 3. *ZnO is a reducible oxide and several other metal oxides (In_2O_3, CeO_2, PtO_2•H_2O) have been recently reported as selective hydrogenation catalysts. Catalytic tests with the pure oxide as benchmark need to be performed. This is to complement Figure 3, where the absence of Zn on the surface leads to incomplete conversion, suggesting zinc as the real active phase.*

Response: We appreciate your valuable suggestion. ZnO is a reducible oxide and can be a hydrogenation catalyst. The catalytic performance of ZnO is tested and added in Figure 3. It showed 51 % conversion after 4 h, with 33.9 % to **3a**, 26.5 % to **3b**, 14.9 % to **3c**, 10.3 % to **2c**, and 14.4 % to **2b**. ZnO exhibits preference hydrogenation to the alkynyl group. Since PtZn/HNCNT exhibits preferential hydrogenation to the nitro group, it indicated that zinc is not the real active phase in the PtZn/HNCNT catalyst. A discussion is added in page 9 about this.

"The catalytic performance of ZnO is also tested. It showed 51% conversion after 4 h, with 33.9% to **3a**. ZnO exhibits preference hydrogenation to the alkynyl group. Since PtZn/HNCNT demonstratess preferential hydrogenation to the nitro group, it further excluded that zinc is the real

active phase in the PtZn/HNCNT catalyst.”

Comment 4. *Only one substrate has been hydrogenated (nitrophenylacetylene), and the final product was even not characterized by liquid 1H NMR, 13C NMR, TGA, DSC, LC-MS, and HR-MS, to prove that it is the correct product. How was the product isolated? Rotatory evaporation? What is the recovery mass yield?*

Response: Thanks very much for your valuable suggestion. The product was isolated by rotatory evaporation first and the product mass obtained is 90 mg (there are some triethoxy borate caused by the ammonia borane). Then the product was isolated by flash column chromatography on silica gel with petroleum ether/ethyl acetate as eluent. After column separation, the product mass obtained is 54.5 mg. The recovery mass yield is 93%. The 1H NMR and 13C NMR spectra of the product are added in the Supplementray information Figure 16 and 17. And a discussion is added in page 9.

“The 1H NMR and 13C NMR further confirmed the product is 4-aminophenylacetylene (Supplementary Fig. 16-17) and the mass recovery yield of **2b** is 93%.”

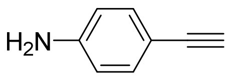

4-Alkynylaniline (2a)[1]. ^{1}H NMR (400 MHz, $CDCl_3$): δ 7.28 (d, J = 8.4 Hz, 2H), 6.56 (d, J = 8.4 Hz, 2H), 3.74 (s, 2H), 2.96 (s, 1H); ^{13}C NMR (101 MHz, $CDCl_3$): δ 147.1, 133.5, 114.7, 111.3, 84.5, 75.0.

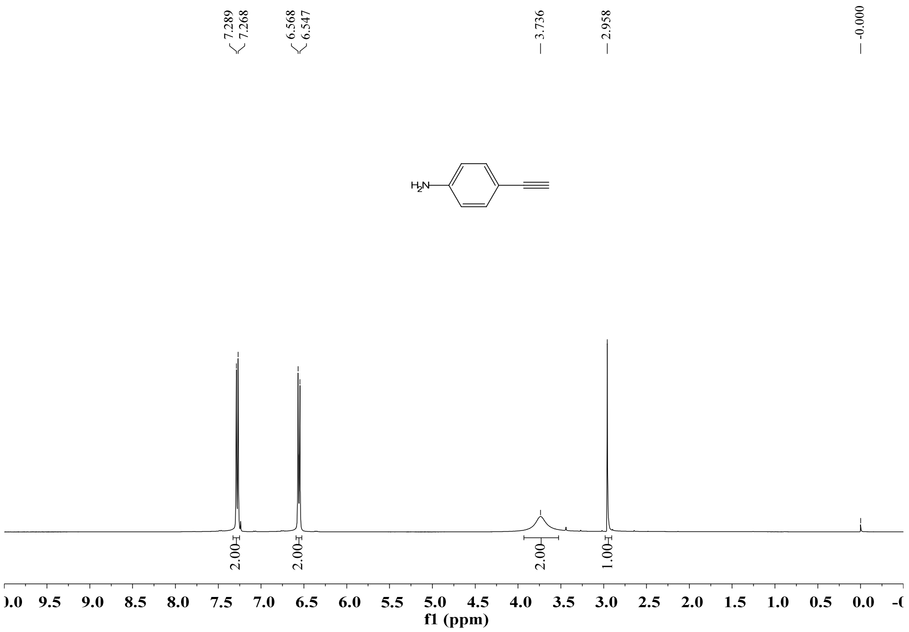

Supplementary Figure 16. ^{1}H NMR (400 MHz, $CDCl_3$) spectrum of 4-alkynylaniline (**2a**).

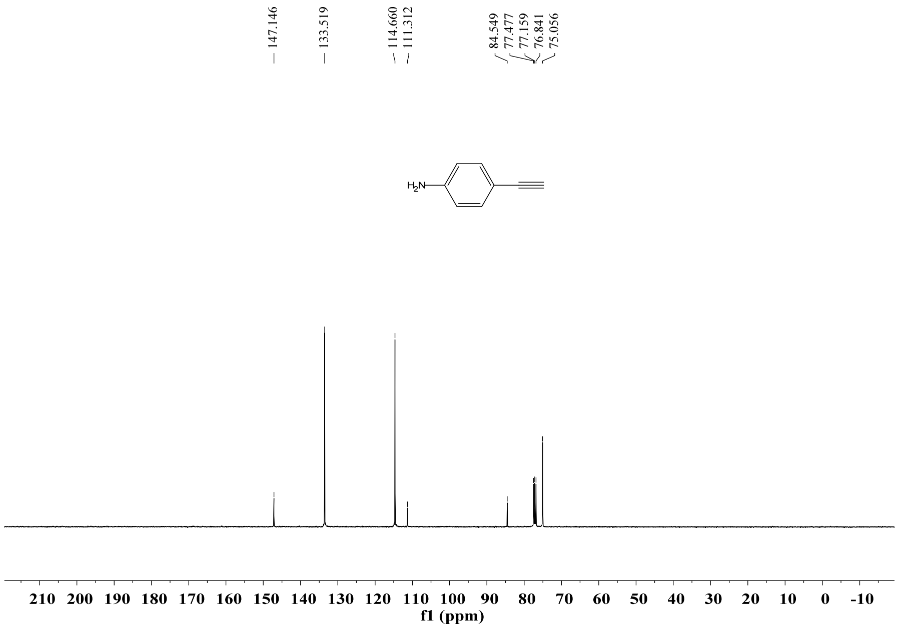

Supplementary Figure 17. ^{13}C NMR (101 MHz, $CDCl_3$) spectrum of 4-alkynylaniline (**2a**).

Comment 5. *With one single substrate, the authors cannot claim that the work could find applications in the 'fine chemical sector'. Please include a broad substrate scope where the nitro-substrate is functionalized with electron donating and withdrawing groups as shown elsewhere (see ACS Catal 2015, 5, 3767-3778).*

Response: We appreciate your valuable suggestion. A broad substrate scope where the nitro-substrate is functionlized with electron donating and withdrawing groups has been added in Supplementary Table 2. It indicated that the PtZn/HNCNT is quite active and selective for hydrogenation of nitrobenzene with different functional groups. It showed high selectivity for nitrobenzene with electron donating and withdrawing groups, no matter which positions the functional group sit. A discussion about this is added in page 10.

"Nitrobenzenes with electron donating and withdrawing groups were also used as the substrates (Supplementary Table 2). PtZn/HNCNT showed high selectivity for functionalized nitrobenzenes with electron donating or withdrawing groups in different positions. It indicated the outstanding performance of PtZn/HNCNT in selective hydrogenation of nitrobenzenes."

Supplementary Table 2. Substrate scope of hydrogenation of functionalized nitrobenzenes over the PtZn/HNCNT catalyst.

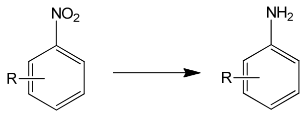

Entry	R	Conv.(%)	Sel.(%)
1	H	100	100
2	4-Cl	100	100
3	4-$CONH_2$	100	100
4	4-CH_2CH_3	94.9	100
5	4-NH_2	100	100
6	2-Cl	100	100
7	3-C≡CH	100	86.5

Condition: substrate 0.5 mmol, ammonia borane 3 mmol, catalyst 2.0 mg, ethanol 4.9 ml, deionized water 0.1 ml, reaction was carried out in a 15 ml pressure tube, reaction time 4 h.

Comment 6. *In Figure 2, XAS is a bulk technique and cannot be used to prove surface Pt dispersion. Please conduct an in depth XPS analysis with Ar sputtering and chemisorption analyses to calculate the Pt dispersion.*

Response: We appreciate your valuable suggestion. The synchrotron radiation photoemission spectroscopy (SRPES) was carried out in the National Synchrotron Radiation Laboratory (NSRL) in Heifei, China. XPS spectra were measured using different photon energies, i.e. 400, 600 and 1486.6 eV respectively. Due to the high binding energy of Zn (> 1000 eV), it cannot be tested using the low photon energy. Therefore, we measured the XPS spectra of Pt and C and calculated the Pt fraction to C. The results are shown bellow in Figure R3-6a. However, since the PtZn intermetallic nanoparticles are not so uniform (ranging from <2 nm to 7 nm), this in-depth XPS results cannot be used to prove the nanoparticles are uniform (Pt:Zn ratio are not changed) from the core to shell through the fraction Pt to C.

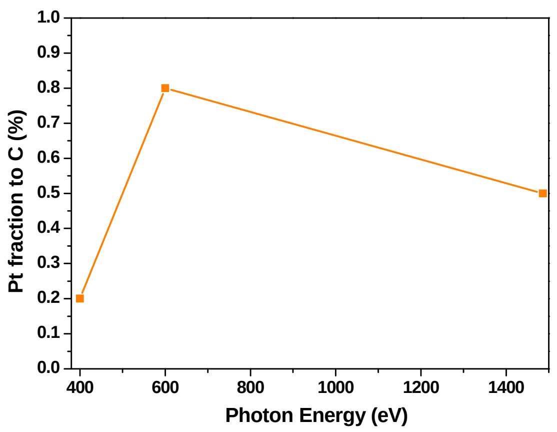

Figure R3-6a. Dependence of Pt fractions to C as a function of photon energy.

CO FTIR was also carried out the study the Pt dispersion shown in Figure R3-6b. However, the peaks are rather low and no much difference could be observed between the sample Pt/HNCNT, Pt/CN, and PtZn/HNCNT, which may be due to the low loading of Pt.

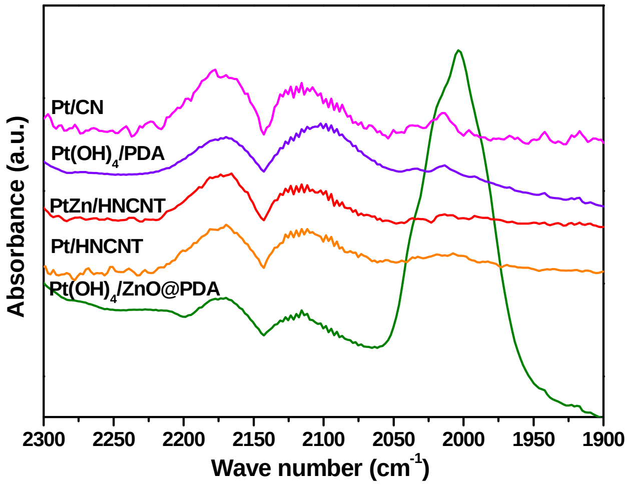

Figure R3-6b. IR-CO chemisorptions stack-plot spectra of PtZn/HNCNT, Pt/HNCNT, $Pt(OH)_4/ZnO$@PDA, Pt/CN and $Pt(OH)_4$/PDA. The in situ Fourier transform infrared (FTIR) spectra of CO adsorption were carried out on a Nicolet 380/ZDF-X-LED in situ infrared spectrometer. The sample (if the transmittance is not satisfied, the sample was mixed with a little amount of KBr) was palletized into a disk. Prior to the CO adsorption, the sample were pretreated with Ar at 200 ^oC for 1 h. After cooling down to room temperature (20 ^oC), the background spectrum was collected. Then, CO gas was introduced into the sample holder for 30 min. The sample was flushing with Ar and a spectra was then collected every 5 min over the course of 30 min. The spectra shown in this figure are taken after 30 min of flushing with Ar.

Comment 7. *The DFT profile in Figure 4 is too preliminary since only H_2 splitting has been calculated. Please show the full hydrogenation profile with the remaining steps (till product desorption).*

Response: Thanks for your suggestion. Actually, we considered the full reaction path at first. The reactant molecule contains two functional groups: alkynyl and nitro. A full hydrogenation reaction path from $CH\text{-}C\text{-}C_6H_4\text{-}NO_2$ to $CH_3\text{-}CH_2\text{-}C_6H_4\text{-}NH_2$ involves 10 hydrogenation steps. In each steps (at least the in the initial several steps), hydrogenation reaction could occur on alpha-C, beta-C and the oxygen sites (In PtZn system, the two oxygen atoms in nitro group at the alloy surface are asymmetric. Hence the hydrogenation reaction on each oxygen atoms should be considered one by one.) In this sense the branches in the entire reaction path contains grow explosive (Figure R3-7a**)**. It is burdensome to investigate the entire reaction path.

In this sense, we determined that the comparing the reaction barriers of the initial hydrogenation step on both alkynyl and nitro on Pt and PtZn alloy surface is good point to emphasize the selectivity in the context. For example, the reaction barrier of hydrogenation on alkynyl is almost twice of it on nitro for PtZn surface, indicating that the higher barrier (201 KJ/mol) is difficult to trigger the subsequent reactions.

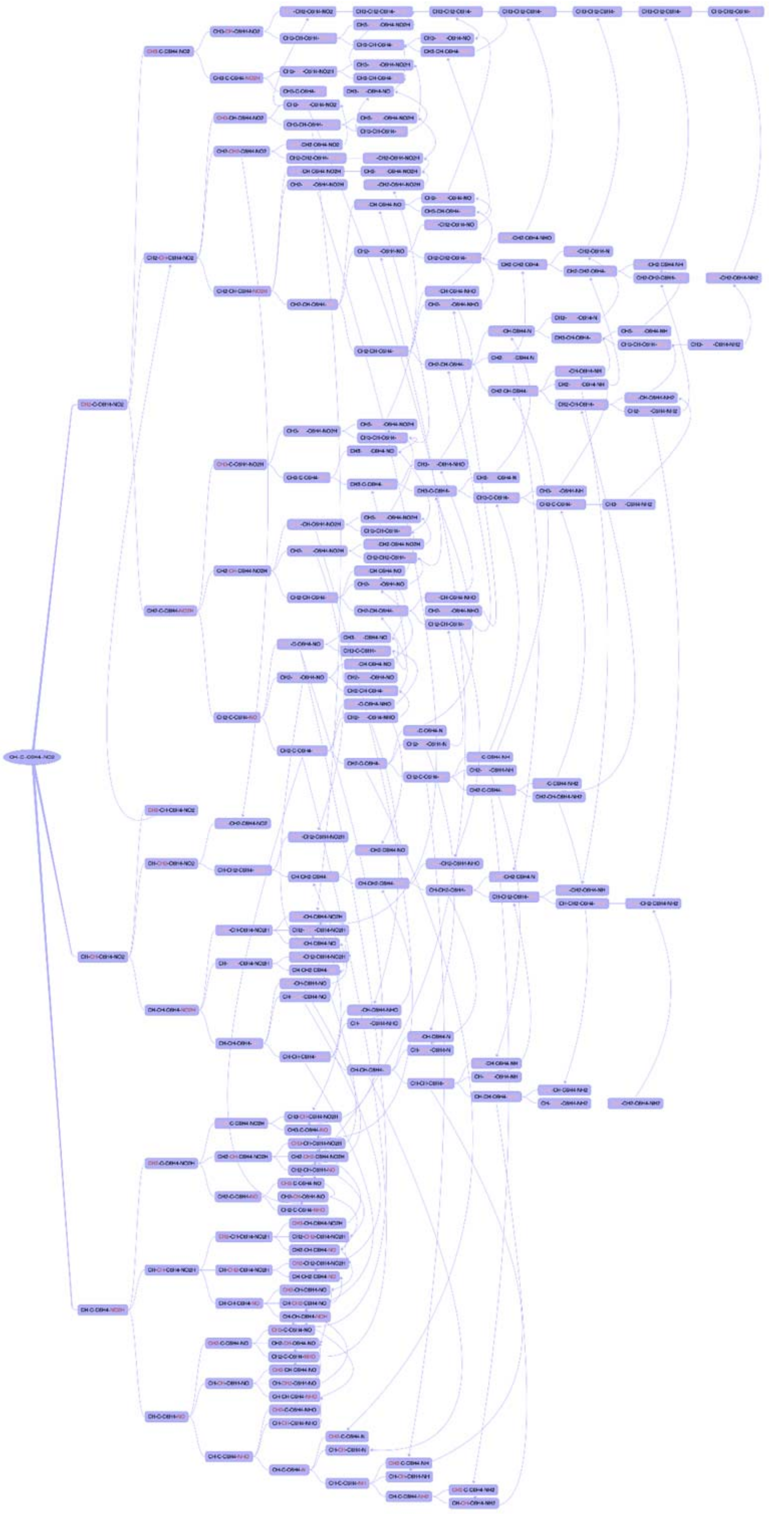

Figure R3-7a. The proposed reaction pathway for hydrogenation of CH-C-C_6H_4-NO_2 on the considered surface. Based on our calculation, the C_mH_n-C_6H_4-NHO is more stable than C_mH_n-C_6H_4-NOH for each considered (m, n). Hence in this pathway, only C_mH_n-C_6H_4-NHO is considered. The hydrogenation sites are marked in red.

In the revised version, we have tried our best to consider the full reaction path for both Pt and PtZn systems. The thermochemistry-only potential energy pathways were constructed (*J. Phys. Chem. C*, 10.1021/acs.jpcc.7b11629 (2018); *J. Phys. Chem. B*, **122**, 432–443 (2018)). For reations on Pt(111) surface, the 1st hydrogen atom was attached on CH, C and NO_2 sites, respectively. Based on the reaction energy calculated, the most thermochemistical favorable intermediate is filtered and selected as the reactant for the next hydrogenation step. The simplified hydrogenation reaction pathway and the corresponding reaction energies of CH-C-C_6H_4-NO_2 on the Pt(111) surface is shown in Supplementary Figure 30 and Supplementary Figure 31. In the first two hydrogenation steps, the alkynyl group is the favorable target, followed by the formation of vinyl group (intermediate **3a** in Fig. 3). The vinyl group does not achieve ethyl till the 10th hydrogenation step; meanwhile, the last hydrogenation step is endothermic. The long-lasting unsaturated C_2H_4- group should be the primitive intermediate for the formation of CH_3-CO-C_6H_4-NO_2 (**3c** in Fig. 3) and CH_3-CH_2-C_6H_4-NO_2 (**3b** in Fig. 3).

For PtZn surface, the alkynyl and nitro group stay on the different surface patterns, where alkynyl and nitro are close to Pt and Zn, respectively. To consider the hydrogenation reaction pathway, firstly, one hydrogen atom was attached on CH, C and NO_2 sites, respectively. Based on the reaction energy calculated, the most thermochemistical favorable alkynyl part and nitro part intermediates are filtered and selected as the reactant for the next hydrogenation step. Even after such filtering, the reaction pathway is still too complex as shown in Supplementary Figure 34.

In this part, an alternative route was considered to correlate with the experimental observation. The overall direct hydrogenation paths has been identified as $PhNO_2^* \rightarrow PhNOOH^* \rightarrow PhNO^* \rightarrow PhNHO^* \rightarrow PhN^* \rightarrow PhNH^* \rightarrow PhNH_2^*$ (*J. Phys. Chem. C*, 10.1021/acs.jpcc.7b11629 (2018); *J. Phys. Chem. B*, 122, 432–443 (2018)). The barriers of hydrogenation reactions on alkynyl group for CH-C-C_6H_4-NO_2/NOOH/NO/NHO/N/NH/NH_2 on alloy surface were calculated. The calculation results are shown in Supplementary Figure 35. We can find that every reaction needs to climb over a barrier higher than 200 KJ/mol, which is difficult to trigger the subsequent hydrogenation reactions on the alkynyl group. We considered that the such high reaction barrier on alkynyl part is the essential reason for the high selectivity on PtZn surface.

In page 12-13 of the manuscript, the DFT part is revised.

"Hydrogenation processes could potentially occur on both nitro and alkynyl groups. To reveal the chemoselective hydrogenation process, the first elementary reaction steps on both groups were investigated. Hydrogen atom prefers to adsorb on the fcc-hollow site on Pt surface (Supplementary Fig. 29). Over Pt(111) surface, the energy barriers in the first step for hydrogenation on nitro group and alkynyl group are 239 and 117 kJ/mol (Fig. 4a), respectively, indicating that the hydrogenation process on nitro group is unfavorable kinetically. The thermochemistry-only potential energy pathways were constructed (Supplementary Fig. 30 and Supplementary Fig. 31)[37,38]. In the first two hydrogenation steps, the alkynyl group is the favorable target, followed by the formation of vinyl group (intermediate **3a** in Fig. 3). The vinyl group does not achieve ethyl till the 10th hydrogenation step, meanwhile, the last hydrogenation step is endothermic. The long-lasting unsaturated C_2H_4- group should be the primitive intermediate for the formation of CH_3-CO-C_6H_4-NO_2 (**3c** in Fig. 3) and CH_3-CH_2-C_6H_4-NO_2 (**3b** in Fig. 3). Over PtZn(02$\bar{2}$) surface, hydrogen atom prefers to adsorb on the Pt-Zn bridge site (Supplementary Fig. 32). The relative vertical distances between H* and its hydrogenation target

atom (oxygen in nitro group or carbon atom in alkynyl group) are comparable (0.897 vs 0.978 Å). For 4-NPA@PtZn(02$\bar{2}$), hydrogenation on nitro group is along the hydrogen atom diffusion path from Pt-Zn(bridge) to Zn-Pt(bridge) (Supplementary Fig. 33). The diffusion barrier is 0.30 eV (28.8 kJ/mol). Meanwhile, the hydrogenation on alkynyl group is along the perpendicular direction. Hydrogen diffusion along this path is unfavorable (diffusion barrier is 1.48 eV). Interestingly, the energy barriers in the first step for hydrogenation on nitro group and alkynyl group are 114 and 201 kJ/mol (Fig. 4b), respectively, indicating that the hydrogenation process on alkynyl group is unfavorable kinetically. Based on the direct hydrogenation path nitro group, the barriers of first hydrogenation step on alkynyl group for CH-C-C_6H_4-NOOH/NO/NHO/N/NH/NH_2 on alloy surface were also calculated (Supplementary Fig. 34 and Fig. 35). We can find that every reaction needs to climb over a barrier higher than 200 kJ/mol, which is difficult to trigger the subsequent hydrogenation reactions on the alkynyl group. We considered that the such high reaction barrier on alkynyl part is the essential reason for the high selectivity on PtZn surface."

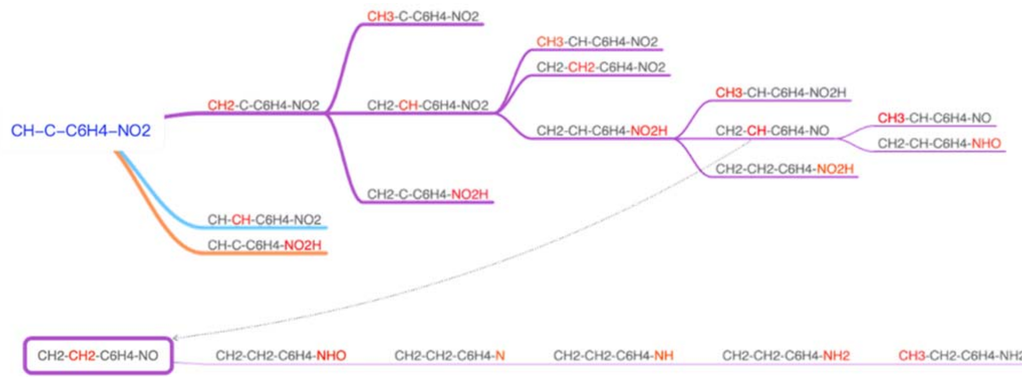

Supplementary Figure 30. The simplified hydrogenation reaction pathway of CH-C-C_6H_4-NO_2 on the Pt(111) surface. The hydrogenation sites are marked in red.

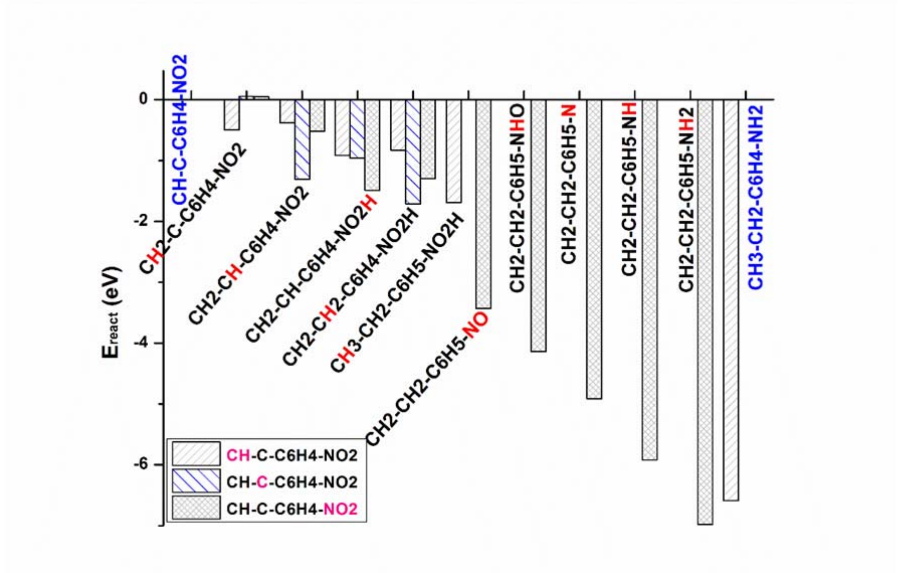

Supplementary Figure 31. The reaction energies of hydrogenation reaction pathway of CH-C-C_6H_4-NO_2 on the Pt(111) surface. The hydrogenation sites are marked in red.

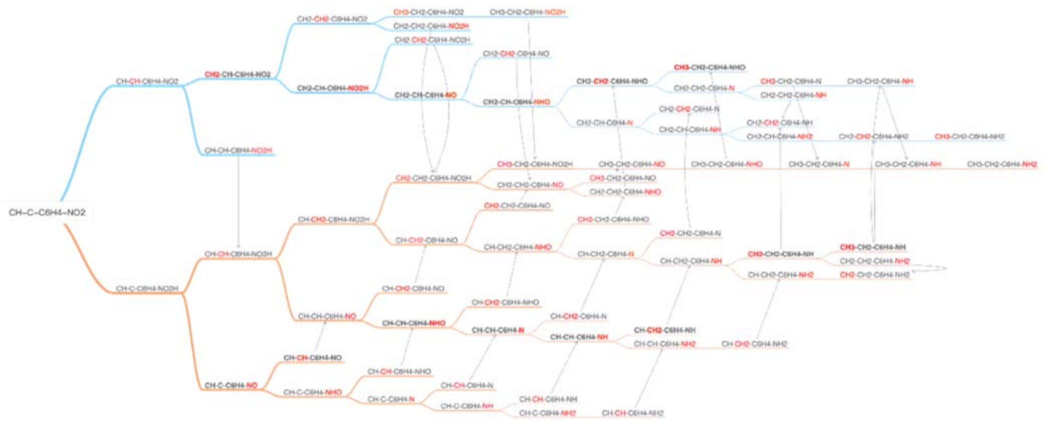

Supplementary Figure 34. The simplified hydrogenation reaction pathway of CH-C-C_6H_4-NO_2 on the PtZn(02$\bar{2}$) surface. The hydrogenation sites are marked in red.

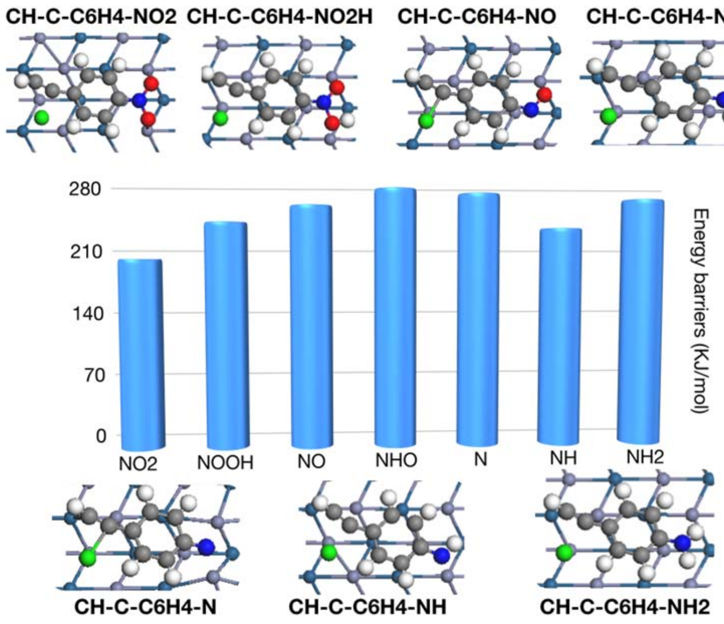

Supplementary Figure 35. The calculated energy barriers of hydrogenation reactions on alkynyl group for CH-C-C_6H_4-NO_2/NOOH/NO/NHO/N/NH/NH_2 on alloy surface. The insets show the corresponding geometries of transition states.

Comment 8. *The authors have preferred to reference to their own papers and several outstanding contributions and advances on hydrogenation catalysis from 2010 to today have been omitted.*

Response: We appreciate your valuable suggestion. We have revised the references with outstanding works hydrogenation catalysis published recently as reference 7-12.

“7 Wei, H. *et al.* FeOx-supported platinum single-atom and pseudo-single-atom catalysts for chemoselective hydrogenation of functionalized nitroarenes. *Nat. Commun.* **5**, 5634 (2014).

8 Liu, L. *et al.* Generation of subnanometric platinum with high stability during transformation of a 2D zeolite into 3D. *Nat. Mater.* **16**, 132-138 (2016).

9 Imaoka, T. *et al.* Platinum clusters with precise numbers of atoms for preparative-scale catalysis. *Nat. Commun.* **8**, 688 (2017).

10 Lucci, F. R. *et al.* Selective hydrogenation of 1,3-butadiene on platinum–copper alloys at the single-atom limit. *Nat. Commun.* **6**, 8550 (2015).

11 Vilé, G., Almora-Barrios, N., López, N. & Pérez-Ramírez, J. Structure and Reactivity of Supported Hybrid Platinum Nanoparticles for the Flow Hydrogenation of Functionalized Nitroaromatics. *ACS Catal.* **5**, 3767-3778 (2015).

12 Iwasa, N., Mayanagi, T., Ogawa, N., Sakata, K. & Takezawa, N. New catalytic functions of Pd–Zn, Pd–Ga, Pd–In, Pt–Zn, Pt–Ga and Pt–In alloys in the conversions of methanol. *Catal. Lett.* **54**, 119-123 (1998)."

Comment 9. *The catalytic tests are also incomplete. The authors have not shown any catalyst stability, demonstrating absence of metal restructuring upon 20-40 h on stream. The stability has to be integrated, of course, with the complete characterization of the used catalyst (metal content, XPS, XAS, XRD, physisorption, and chemisorption). The catalytic performance has to be complemented with a full kinetic analysis, where the authors take a material and investigate the effect of temperature and pressure, in order to identify the optimal reaction conditions. In the Supporting Information, it is also evident that the authors have taken only 2 mg of catalyst and performed the tests with such a small quantity of material. This is however (at least in my experience) unpractical, since small variation in the mass (2.5 mg instead of 2) will bring to huge differences (25% more). Please conduct new tests using at least 100 mg of catalyst.*

Response: We appreciate your valuable suggestion.

(1) The stability and characterizations after reactions are carried out and shown in Supplementary Figure 21-26. A discussion is added in page 10.

"Besides, the stability of the catalyst was tested. After reaction, there was nearly no change in the metal content (0.82% before and 0.80% after reaction), XRD, XPS, XAS, and N_2 sorption for PtZn/HNCNT (Supplementary Fig. 21-25). No obvious decrease in the catalytic performance was observed (Supplementary Fig. 26), suggesting the robust nature of the PtZn/HNCNT."

The CO FTIR of Pt/HNCNT, Pt/CN, and PtZn/HNCNT before and after reactions were carried out, and the spectra are shown below in Figure R3-9. Even though no obvious changes could be observed, the peaks are low due to the low loading of Pt and cannot be used to state that the samples are stable, so we did some other experiments to prove the stability of the sample.

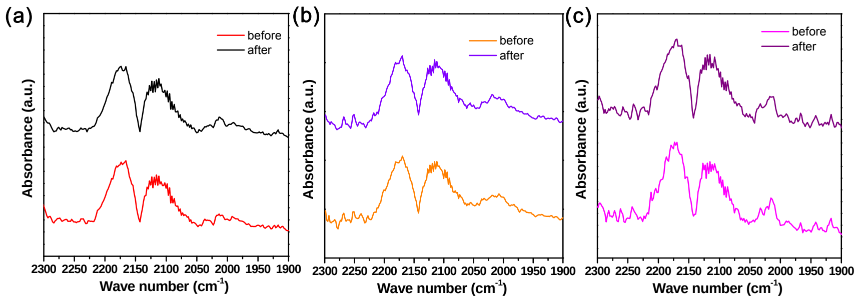

Figure R3-9. CO FTIR spectra of (a) PtZn/HNCNT, (b) Pt/HNCNT and (c) Pt/CN before and after the reaction.

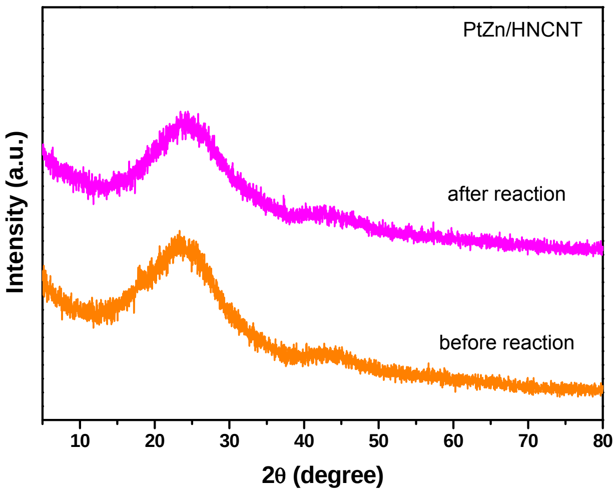

Supplementary Figure 21. XRD patterns of PtZn/HNCNT before and after the reaction.

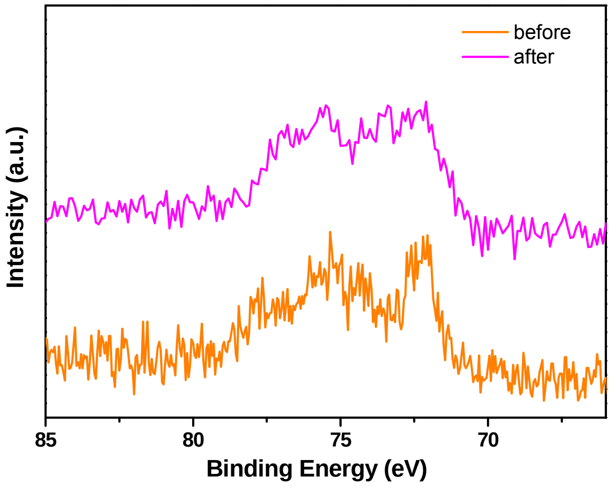

Supplementary Figure 22. Pt 4f XPS of PtZn/HNCNT before and after reaction.

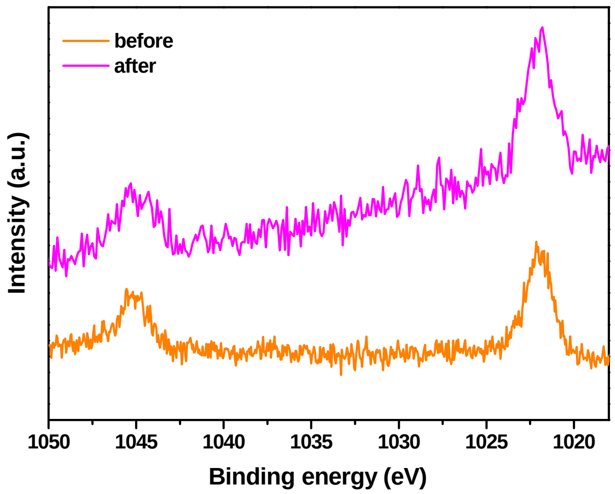

Supplementary Figure 23. Zn 2p XPS of PtZn/HNCNT before and after reaction.

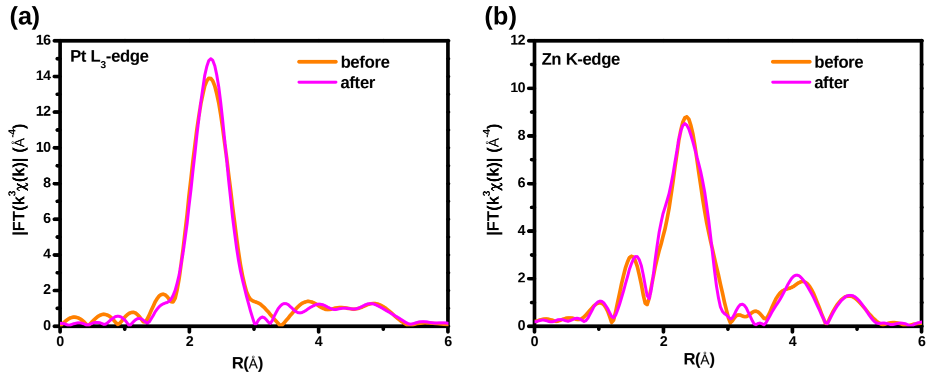

Supplementary Figure 24. The FT-EXAFS at the (a) Pt L_3-edge and (b) Zn K-edge of PtZn/HNCNT before and after the reaction.

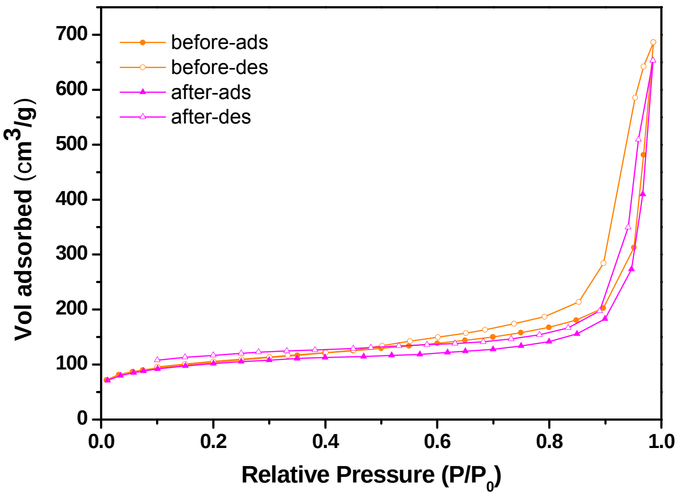

Supplementary Figure 25. N_2 adsorption and desorption isotherm of PtZn/HNCNT before and after the reaction.

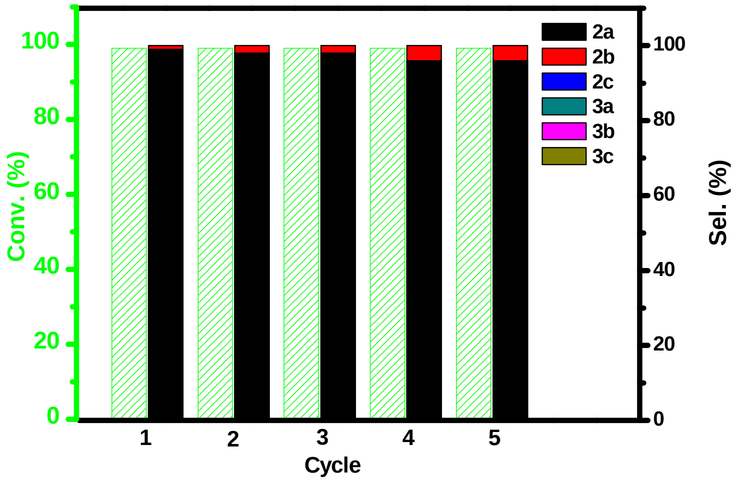

Supplementary Figure 26. Recyclability of PtZn/HNCNT in the selective hydrogenation of 4-nitrophenylacetylene.

(2) The kinetic curve is added in Figure S18. A discussion is added in page 9.

"The kinetic profile as followed and shown in Supplementary Figure 18. The conversion increased fast in the first 2 h while the selectivity kept 99 % to **2a**."

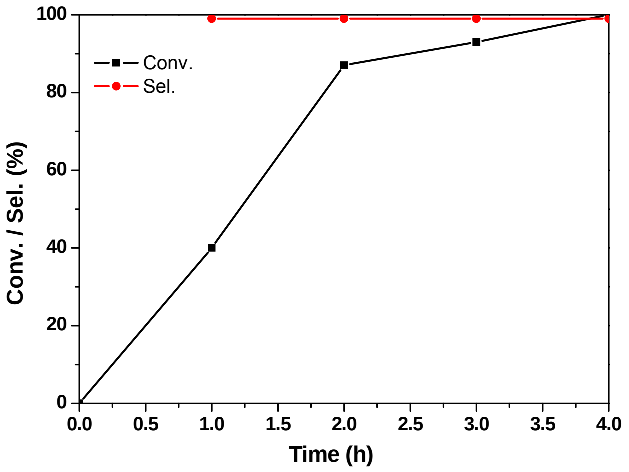

Supplementary Figure 18. Kinetic profile of 4-nitrophenylacetylene hydrogenation over PtZn/HNCNT.

(3) The effect of temperature and pressure is added in Table S1. A discussion is added in page 9.

"Different reaction conditions was also studied and shown in Supplementary Table 1. The highest selectivity was obtained at 40 ^{o}C. Since the reaction was carried out in the pressure tube, a larger volume of tube was used to reduce the pressure during the reaction. However, a decreased selectivity was observed."

Supplementary Table 1. Effect of temperature and pressure in the hydrogenation of 4-nitro phenylacetylene over the PtZn/HNCNT catalyst.

Entry	Temp (^{o}C)	Time (h)	Tube Vol (ml)	Conv.(%)	Sel.(%)
1	40	4	15	100	98.0
2	60	2	15	99.0	76.5
3	70	1	15	99.5	70.0
4	40	4	35	100	90.0

Condition: 4-nitrophenylacetylene 0.5 mmol, ammonia borane 3 mmol, catalyst 2.0 mg, ethanol 4.9 ml, deionized water 0.1 ml, reaction was carried out in a pressure tube.

(4) The support of our material is hollow carbon nanotubes, which are light-weighted. A 100 mg of catalyst in the 5 ml of solvent caused slurry that is not easy to stir during the reaction. We scale up the reaction to 10 mg, and the conversion (100%) and selectivity (98%) is nearly the same as that when using 2 mg catalyst. Therefore, we carried out the reactions using 2 mg catalyst.

Comment 10. *What is the real active phase? I propose as well to complement the work with some operando XAS studies to address this important question.*

Response: We appreciate your suggestion. Base on the experimental results (AC HAADF STEM,

ex situ XAS, and controlled activity tests), we think that PtZn intermetallic nanoparticles are the active phase. This reaction is carried out in a pressure tube. We're sorry that we didn't find a proper operando setup to do the operando XAS studies. Even so, we tried our best to prove that PtZn is the active phase. More AC HAADF STEM images are taken and one of them showed a clear Pt and Zn atomic arrangement (Supplementary Figure 7). XRD of a PtZn/HNCNT sample prepared by the same method but higher Pt loading clearly showed the diffraction patterns of PtZn (JCPDS No. 06-0604). Those observations indicated that PtZn are formed under our condition. Besides, ZnO is used as the catalyst but showed a different hydrogenation direction towards the alkynyl group, excluding the possibility that zinc sinter at the surface of Pt nanoparticles (easily form ZnO as no pretreatment was carried out before the reaction) as the active phase. Besides, Pt/CN was prepared without using ZnO and only Pt nanoparticles are formed. It also exhibited preference to the alkynyl group, indicating that Pt cannot be the active phase. Therefore, we think that PtZn intermetallic compounds are the real active phase.

Comment 11. *Intermetallic catalysts (Pd-Zn and Pt-Zn) have been already widely reported (see Catal Lett 1998, 54, 119-123). Can the author compare both a n intermetallic Pt-Zn with core-shell catalysts (Pt in the core with Zn on the surface; Pt on the surface and Zn in the core).*

Response: We appreciate your valuable suggestion. Since Zn can easily evaporate at high temperature, it is hard to synthesis Pt-Zn core-shell catalyst. We have searched previous report carefully, but failed to find an effective method to prepare intermetallic PtZn with core-shell structures. However, we prepared Pt_3Zn/HNCNT, which are enriched with Pt (the surface of the particle is also enriched with Pt) compared to PtZn/HNCNT. It shows high selectivity for the alkynyl group (conversion after 4 h: 50.7%, selectivity: 39.6% for **3a**, 23.4% for **3b**, 19.4% for **3c**, 11.4% for **2b**, 6.2% for **2c**). Besides, ZnO (in a Pt-Zn core-shell structure, the surface is enriched with zinc, which can easily form ZnO if no pretreatment was carried out before the reaction) was also tested as a catalyst. It also selectively hydrogenates the alkynyl group. Therefore, we think that PtZn with the 1:1 ratio, where Pt atoms are isolated into single atomic sites, is important for this selective hydrogenation process.

Comment 12. *Please remove lines 141-143, since this is not correct.*

Response: Thank you for this comment. We have deleted the sentence "It is noteworthy that this hydrogenation reaction is usually carried out under hydrogen with high pressure and high temperature (>80 ^{o}C), limiting its practical applications.[28-32]" in the line 141-143.

Comment 13. *The paper is a clear example on catalysis on nanoparticles. The authors, however, have preferred to choose the catchy 'Pt-Zn bridge sites' terminology (title and abstract) to recall the exciting and fast-moving field of single-site catalysis, likely to attract citations. This title is however misleading, considering that the work provides little insights on single-site catalysis, and thus must be modified, substituting 'bridge sites' with 'intermetallic nanoparticles'.*

Response: We are very grateful for your valuable suggestion. According to your suggestion, we use "intermetallic nanoparticles" to replace "bridge site" in the title, and revise the title of the paper as "Isolating contiguous Pt atoms and forming Pt-Zn intermetallic nanoparticles to regulate

selectivity in catalytic hydrogenation of 4-nitrophenylacetylene”.

Reviewers' comments:

Reviewer #1 (Remarks to the Author):

The points I have raised previously have not been satisfactorily addressed. I still have many points of concern with this work, rendering it not yet suitable for publication in Nature Communications.

1) The authors included Supplementary Figure 18 to address my concerns on the potential difference in selectivity at different conversion range. However, the selectivity (S. Figure 18) was collected at transient state. The selectivity should be measured at steady state and similar conversion by decreasing the amount of catalysts or changing a space velocity.

2) CO FTIR spectra of PtZn/HNCNT, Pt/HNCNT, $Pt(OH)_4/ZnO$@PDA, Pt/CN and $Pt(OH)_4$/PDA are not still convincing that PtZn/HNCNT catalyst has atomically dispersed Pt species. The authors ascribed its low (almost no) intensity to the low Pt loading but the Pt loading does not seem low considering the number of atoms seen in Figure 1 g. Also, if the Pt loading is ~0.8wt% as said in the draft, we should be able to see very clear spectra. Previous single atom work (Derita et al. J. Am. Chem. Soc. 2017, 139, 14150−14165) reported very clear a Pt single atom peak with 0.05wt% Pt/TiO_2 catalyst. It is possible that the clear spectra was not observed because the catalysts were not properly pre-treated. Oxygen on Pt species should be properly removed by in situ reduction to induce the interaction between CO and Pt species for CO FTIR.

3) The authors said that catalysts were not pretreated before characterizations and reaction. Pretreatment of catalysts is important to characterize the intrinsic properties of catalysts because catalysts generally undergo changes such as oxidation state and surface reconstruction during pretreatment and reaction. However, all characterization results included were collected neither in situ nor operando, so I am not sure whether these results properly represent the intrinsic structure of catalysts.

4) The authors claimed that the ZnO reduction to zinc vapor helps the contiguous Pt atoms to isolate by forming Pt-Zn intermetallic structure. Therefore, I assume that Pt/HNCNT, which was synthesized without Zn, should include contiguous Pt species. However, Pt-Pt contribution was not observed in Pt/HNCNT like PtZn/HNCNT, and this is questioning the role of Zn. As the authors claimed, it might be true that the presence of ZnO leads the formation of Pt-Zn intermetallic structure but I am not sure about Zn species helping to isolate Pt atoms.

5) In the response, the authors claimed "Nearly no reaction occurred in the absence of Pt (Supplementary Fig. 19-20), proving that the active species are the Pt metals." Supplementary Fig. 19-20 are nothing related to reactivity. This should be corrected.
In addition, the authors need to be cautious about the active species because, in the response, the authors said Pt-Zn intermetallic structure is important by stating, "PtZn with the 1:1 ratio, where Pt atoms are isolated into single atomic sites, is important for this selective hydrogenation process."

Reviewer #2 (Remarks to the Author):

The responses to the comments are mostly satisfactory and the manuscript has been improved a lot after the revision. There are two issues, which still need the author to consider carefully before publication.

1. The significance in term of synthesis and catalytic performance of PdZn is ok; however, the bifunctional effect at the Pt-Zn bridge is rather old topic, which is not new at all.

2. It is not straightforward to understand why the Pt segregation on PtZn alloy is not preferred. Should

Pt have lower surface energy than Zn? The fundamental understanding based on theory should be included. In addition, even if Zn stay on the surface, will it be stable under reaction conditions? Will Zn to ZnO transition occur? Is there any experimental evidence on that?

Reviewer #3 (Remarks to the Author):

The authors have made minor modifications to the paper, including only a few ex situ characterizations (which do not provide further insights) and a substrate scope analysis. All major comments raised by Reviewers #2 and #3 during the first round of Peer Review on the surface segregation of metal species, core-shell synthesis, and microkinetic analysis have remained unaddressed.

In the replies, the authors write: "we have tried our best to consider the full reaction path for both Pt and PtZn systems. The thermochemistry-only potential energy pathways were constructed (J. Phys. Chem. C, 10.1021/acs.jpcc.7b11629 (2018); J. Phys. Chem. B, 122, 432–443 (2018))". These two papers have nothing to do with the reduction of nitroaromatics to anilines, as they deal with the mechanism of nitric oxide reduction to nitrogen. The full reaction scheme (which is as well unreadable) is hence questionable.

In the Supporting Info, the authors have included a scheme of the possible reaction path, but have not conducted a full energy analysis of the different steps by DFT (as requested by Reviewers #2 and #3), indicating for instance which elementary steps are critical to the reaction. Thus, there is still no fundamental understanding of the process in the work.

Many sentences in the DFT part seems to be taken from already published papers (see: Journal of Advanced Physics, 2016, 5, pp. 354-358). The authors mention: "the hydrogenation on alkynyl group is along the perpendicular direction", which is questionable considering that catalysis happens at the surface (and the surface here is planar). Most of the theoretical figures are in kJ/mol but, in the discussion, the authors jump from eV to kJ/mol, rendering the DFT analysis unclear and suspicious.

There have been many reports on nitro hydrogenation on PtZn systems (ACS Catal, 2016, 6, 742–746; J. Mol. Catal. 1992, 71, 317-333; ACS Catal., 2014, 4, pp 1441–1450). I do not see what the added value of this publication truly is, or what the novelty of this paper is. It will not attract enough citations.

For all these comments, the paper remains unsuitable for publication and I again recommend rejection.

Dear all reviewers,

Thank you very much for reviewing our manuscript entitled "Isolating contiguous Pt atoms and forming Pt-Zn intermetallic nanoparticles to regulate selectivity in catalytic hydrogenation of 4-nitrophenylacetylene" (reference number: NCOMMS-18-27012A). We have carefully read your valuable comments and appreciate for your efforts which help us to improve the quality of the whole manuscript. Based on the reviewers' comments and the revision request, we have tried our best to revise the manuscript and supplementary information accordingly. The reviewers' comments are laid out below in *Italic font* and specific concerns have been numbered. Our responses are given in normal font and changes/additions to the manuscript and supplementary information are highlighted by using red colored text.

Following is our point to point answer to all the comments:

Reviewer #1:
The points I have raised previously have not been satisfactorily addressed. I still have many points of concern with this work, rendering it not yet suitable for publication in Nature Communications.
Comment 1. *The authors included Supplementary Figure 18 to address my concerns on the potential difference in selectivity at different conversion range. However, the selectivity (S. Figure 18) was collected at transient state. The selectivity should be measured at steady state and similar conversion by decreasing the amount of catalysts or changing a space velocity.*
Response:
We thank the reviewer and appreciate the insightful comment and suggestion, which has helped us much in improving the quality of our manuscript. As suggested by your respected reviewer, we have measured the catalytic performance by decreasing the amount of catalysts, and collected selectivity at a steady state. The PtZn/HNCNT still exhibits high selectivity to 4-aminophenylacetylene, indicating its outstanding hydrogenation selectivity. These results are added in 1st paragraph line 17-20 page 10 of the main text.
"To compare the potential difference in selectivity of those catalysts, the selectivity was compared at similar conversion level (Supplementary Fig. 25). PtZn/HNCNT exhibited extremely high selectivity to **2a** compared to the Pt/HNCNT and Pt/CN samples."

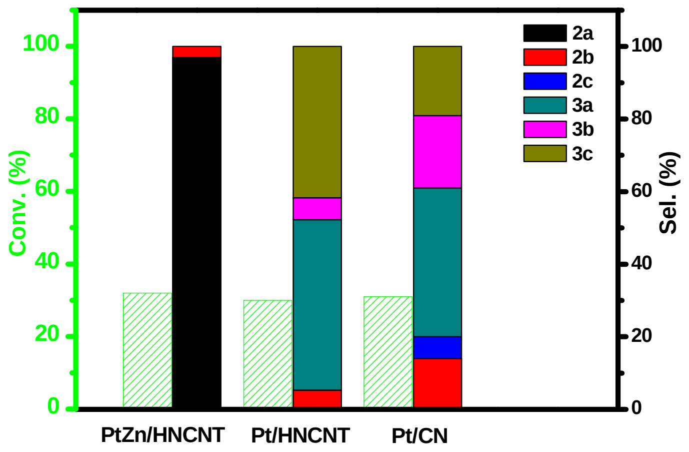

Supplementary Figure 25. The catalytic performance of PtZn/HNCNT, Pt/HNCNT and Pt/CN at similar conversion level.

Comment 2. *CO FTIR spectra of PtZn/HNCNT, Pt/HNCNT, Pt(OH)$_4$/ZnO@PDA, Pt/CN and Pt(OH)$_4$/PDA are not still convincing that PtZn/HNCNT catalyst has atomically dispersed Pt species. The authors ascribed its low (almost no) intensity to the low Pt loading but the Pt loading does not seem low considering the number of atoms seen in Figure 1 g. Also, if the Pt loading is ~0.8wt% as said in the draft, we should be able to see very clear spectra. Previous single atom work (Derita et al. J. Am. Chem. Soc. 2017, 139, 14150−14165) reported very clear a Pt single atom peak with 0.05wt% Pt/TiO$_2$ catalyst. It is possible that the clear spectra was not observed because the catalysts were not properly pre-treated. Oxygen on Pt species should be properly removed by in situ reduction to induce the interaction between CO and Pt species for CO FTIR.*

Response:

We really appreciate the reviewer's insightful comments and valuable suggestions. It is amazing that clear CO FTIR spectra are obtained after the catalysts were pre-treated properly according to the respected reviewer's suggestions. Here, we want to express our sincere thanks to the reviewer for the skillful suggestions, which help us much to get useful and clear CO FTIR spectra. The CO FTIR spectra of PtZn/HNCNT, Pt/HNCNT and Pt/CN are added as Supplementary Figure 19 in page 16 of Supplementary Information. A discussion about the CO FTIR spectra is added in the main text, page 8, 2nd paragraph line 1-7.

"CO adsorption was carried out to study the dispersion and electronic structures of Pt phase (Supplementary Fig. 19). There is a red shift of the CO stretching frequency for PtZn/HNCNT compared to Pt/CN (from 2083 cm^{-1} to 2053 cm^{-1}), indicating that the electron density is increased for PtZn/HNCNT due to the presence of zinc[37], in consistent with the observation of Pt XPS. Pt/HNCNT demonstrates a peak at 2143 cm^{-1}, further confirming that the Pt component in this sample is dispersed as isolated-single-atomic-sites[38]."

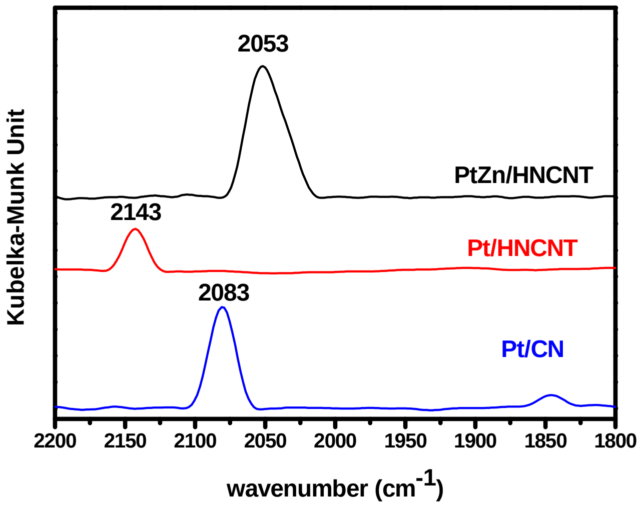

Supplementary Figure 17. *In situ* FTIR spectra of CO chemisorption for PtZn/HNCNT, Pt/HNCNT, and Pt/CN.

Comment 3. *The authors said that catalysts were not pretreated before characterizations and reaction. Pretreatment of catalysts is important to characterize the intrinsic properties of catalysts because catalysts generally undergo changes such as oxidation state and surface reconstruction during pretreatment and reaction. However, all characterization results included were collected neither in situ nor operando, so I am not sure whether these results properly represent the intrinsic structure of catalysts.*

Response:

We appreciate the reviewer's insightful comments very much. Pretreatment of catalysts is really important to characterize the intrinsic properties of catalysts. We totally agree with this point. The samples in our work were reduced in H_2 (5% in Ar) atmosphere during the synthetic process, and used in the characterizations and catalytic reactions immediately after the preparation. Hence, it equates that there are pretreatment process for our catalysts. We conducted another reduction process in H_2 (5% in Ar) atmosphere the same as that in the synthetic process before the characterization and catalytic reaction, if the catalysts were kept for a long time. Therefore, our characterization results are identical to be taken with pretreatment, and these results could represent the intrinsic structure of catalysts.

Comment 4. *The authors claimed that the ZnO reduction to zinc vapor helps the contiguous Pt atoms to isolate by forming Pt-Zn intermetallic structure. Therefore, I assume that Pt/HNCNT, which was synthesized without Zn, should include contiguous Pt species. However, Pt-Pt contribution was not observed in Pt/HNCNT like PtZn/HNCNT, and this is questioning the role of Zn. As the authors claimed, it might be true that the presence of ZnO leads the formation of Pt-Zn intermetallic structure but I am not sure about Zn species helping to isolate Pt atoms.*

Response:

We sincerely appreciate your kind comment. We are sorry that we didn't write clearly enough in our former manuscript. In order to more clearly demonstrate the synthetic strategy of our work, we have carefully revised our manuscript (*please see: 1st paragraph line 5-13, page 7*).

Here, we want to give your respected reviewer detailed explanation of the synthetic mechanism of PtZn/HNCNT. In this synthetic strategy, the ZnO reduction to zinc vapor indeed helps the contiguous Pt atoms to isolate by forming Pt-Zn intermetallic structure. Pt/HNCNT, which was synthesized at low temperature (200 ^{o}C) to avoid the involvement of Zn in the final product, is composed of Pt isolated-single-atomic-sites, and therefore Pt-Pt contribution was not observed from XAFS characterization for Pt/HNCNT. AC HAADF STEM image of Pt/HNCNT is added as Supplementary Figure 12 (page 11 of the Supplementary Information) to further testify that the Pt are dispersed atomically in the Pt/HNCNT. The Pt/CN, which was synthesized using PDA nanospheres instead of ZnO@PDA as the support to avoid the involvement of Zn in the final product, is composed of Pt nanoparticles, as Pt atoms tend to aggregate at the high-temperature reduction process (800 ^{o}C). The XAFS spectra of Pt/CN (Figure 2 in page 9 of the main text) are added in this revised manuscript, and obvious Pt-Pt bonds could be observed for this Pt/CN (Figure 2c). More evidences including XRD, TEM, AC HAADF and elemental mapping are provided as Supplementary Figure 13 in page 12 of the Supplementary Information. These results prove well that our synthetic mechanism is correct, i.e. Pt atoms tend to aggregate into contiguous Pt (forming Pt nanoparticles) at high temperature, and the presence of ZnO would generate zinc vapor, helping to isolated Pt atoms and thus leading to the formation of Pt-Zn intermetallic structure.

These results have been added in the main text, 1st paragraph line 5-13 of page 7 and 1st paragraph line 11-14 as well as 2nd paragraph line 1-7 of page 8 as follows.

"AC HAADF STEM image (Supplementary Fig. 12) shows only bright dots on the support, indicating that the Pt component exists in the form of isolated-single-atomic-sites. Pt/CN, prepared by a similar method using polydopamine nanospheres as the support and treated in H_2/He atmosphere at 800 ^{o}C, contains obvious Pt nanoparticles, as deduced from XRD, TEM and AC HAADF STEM (Supplementary Fig. 13). These results prove that Pt atoms tend to aggregates upon high temperature, and the presence of ZnO helps to isolate contiguous Pt atoms by forming Pt-Zn bridge sites in the PtZn intermetallic nanoparticles."

"Pt-Pt bonds at around 2.4 Å were obviously observed in Pt/CN, while only a peak at 1.6 Å in the first shell was observed in Pt/HNCNT (Fig. 2c), furthering confirming that the Pt dispersed as nanoparticles and isolated-single-atomic-sites in Pt/CN and Pt/HNCNT respectively."

"CO adsorption was carried out to study the dispersion and electronic structures of Pt phase (Supplementary Fig. 19). There is a red shift of the CO stretching frequency for PtZn/HNCNT compared to Pt/CN (from 2083 cm^{-1} to 2053 cm^{-1}), indicating that the electron density is increased for PtZn/HNCNT due to the presence of zinc[37], in consistent with the observation of Pt XPS. Pt/HNCNT demonstrates a peak at 2143

cm^{-1}, further confirming that the Pt component in this sample is dispersed as isolated-single-atomic-sites[38]."

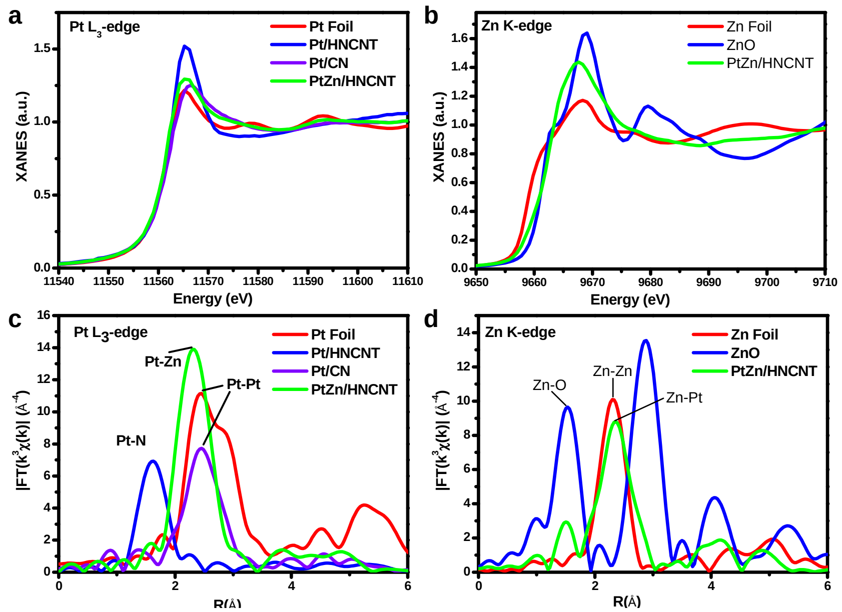

Fig. 2 | X-ray absorption spectroscopy characterization of the catalysts. a,c, The normalized XANES spectra (a) and FT-EXAFS (c) at the Pt L_3-edge of the PtZn/HNCNT, Pt/HNCNT, Pt/CN and Pt foil. b,d, The normalized XANES spectra (b) and FT-EXAFS (d) at the Zn K-edge of the PtZn/HNCNT and Zn foil.

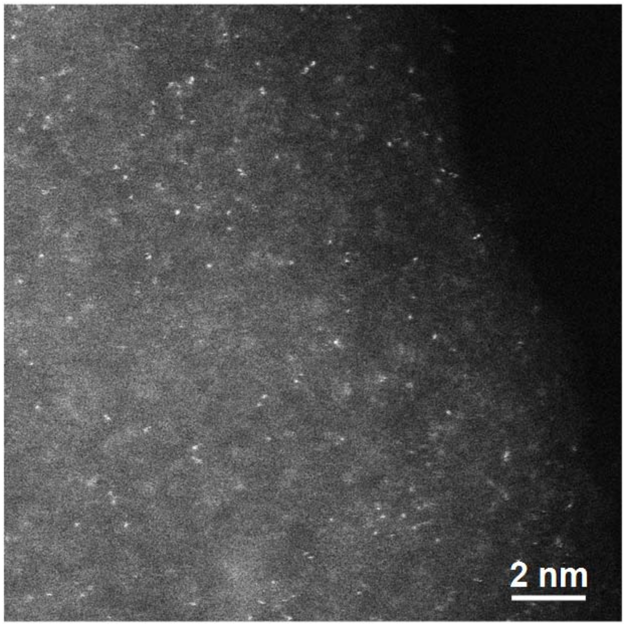

Supplementary Figure 12. AC HAADF STEM image Pt/HNCNT.

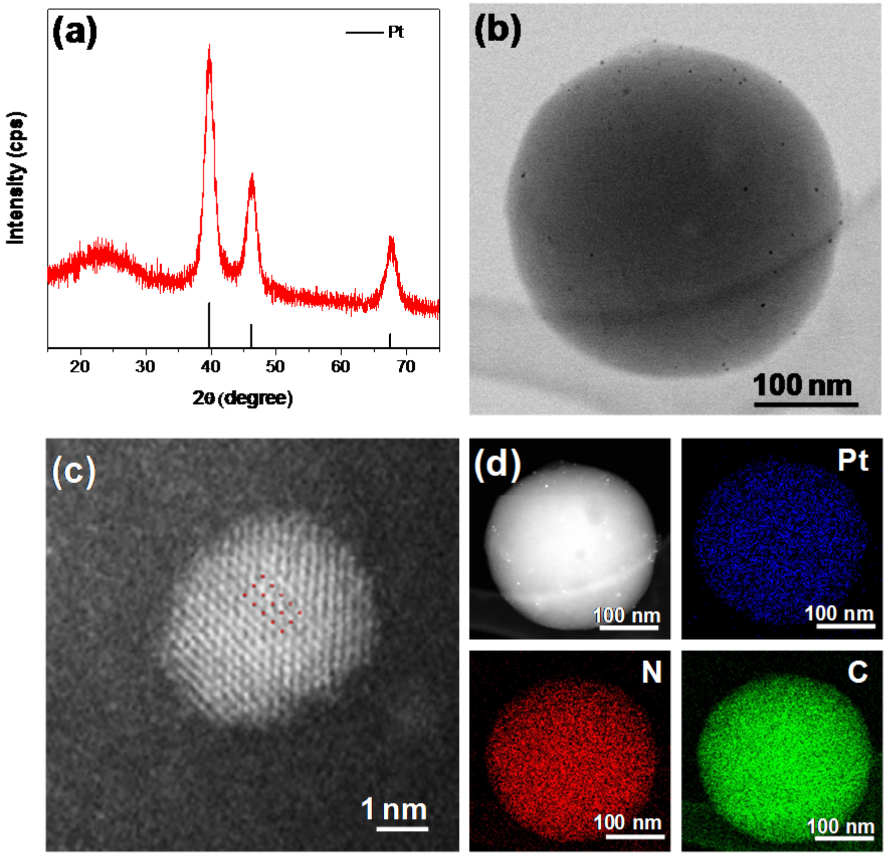

Supplementary Figure 13. Characterizations of the Pt/CN catalyst. XRD (a), TEM (b), AC HAADF STEM image (c), and elemental mappings (d) of Pt/CN.

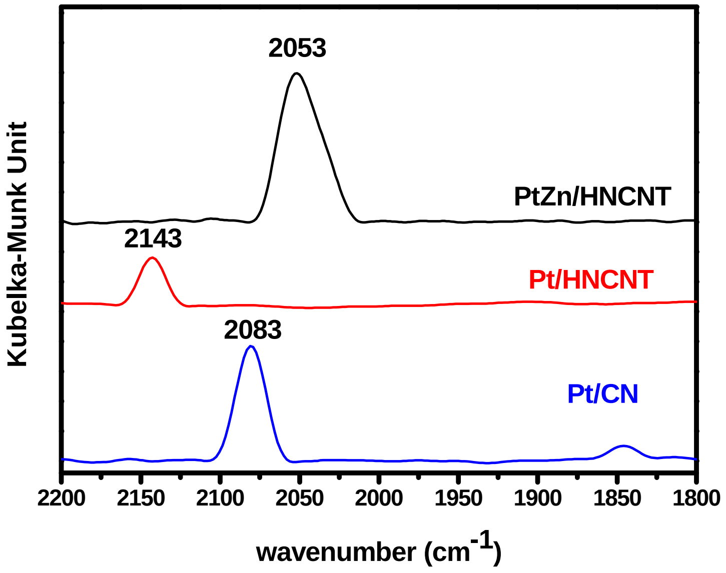

Supplementary Figure 19. *In situ* FTIR spectra of CO chemisorption for PtZn/HNCNT, Pt/HNCNT, and Pt/CN.

Comment 5. *In the response, the authors claimed "Nearly no reaction occurred in the absence of Pt (Supplementary Fig. 19-20), proving that the active species are the Pt metals." Supplementary Fig. 19-20 are nothing related to reactivity. This should be corrected.*

Response:

We appreciate your kind comment. The supplementary Figs. 19-20 are characterization of sample in the absence of Pt, i.e. the HNCNT. We are sorry that we didn't write clearly enough in the previous version. We revise this description in 1st paragraph line 13 page 10 of the main text to avoid misunderstanding.

"Nearly no reaction occurred in the absence of Pt (i.e. HNCNT, its characterizations are shown in Supplementary Fig. 23-24), proving that the active species are the Pt metals."

Comment 6. *In addition, the authors need to be cautious about the active species because, in the response, the authors said Pt-Zn intermetallic structure is important by stating, "PtZn with the 1:1 ratio, where Pt atoms are isolated into single atomic sites, is important for this selective hydrogenation process."*

Response:

Thanks very much for your valuable comment. We are sorry that we didn't write clearly enough about the active species in our former manuscript. In order to more clearly demonstrate the active species, we have carefully revised our manuscript about the comparison sample to highlight the importance of isolating contiguous Pt atoms and forming Pt-Zn bridge site in the selective hydrogenation of the 4-nitrophenylacetylene (*please see: 1st paragraph, page 7; 1st and 2nd paragraph of page 8; 1st paragraph, page 10.*).

"AC HAADF STEM image (Supplementary Fig. 12) shows only bright dots on the support, indicating that the Pt component exists in the form of isolated-single-atomic-sites. Pt/CN, prepared by a similar method using polydopamine nanospheres as the support and treated in H_2/He atmosphere at 800 ^{o}C, contains obvious Pt nanoparticles, as deduced from XRD, TEM and AC HAADF STEM (Supplementary Fig. 13). These results prove that Pt atoms tend to aggregates upon high temperature, and the presence of ZnO helps to isolate contiguous Pt atoms by forming Pt-Zn bridge sites in the PtZn intermetallic nanoparticles."

"Pt-Pt bonds at around 2.4 Å were obviously observed in Pt/CN, while only a peak at 1.6 Å in the first shell was observed in Pt/HNCNT (Fig. 2c), furthering confirming that the Pt dispersed as nanoparticles and isolated-single-atomic-sites in Pt/CN and Pt/HNCNT respectively."

"CO adsorption was carried out to study the dispersion and electronic structures of Pt phase (Supplementary Fig. 19). There is a red shift of the CO stretching frequency for PtZn/HNCNT compared to Pt/CN (from 2083 cm^{-1} to 2053 cm^{-1}), indicating that the electron density is increased for PtZn/HNCNT due to the presence of zinc[37], in consistent with the observation of Pt XPS. Pt/HNCNT demonstrates a peak at 2143 cm^{-1}, further confirming that the Pt component in this sample is dispersed as isolated-single-atomic-sites[38]."

"To compare the potential difference in selectivity of those catalysts, the selectivity was compared at similar conversion level (Supplementary Fig. 25). PtZn/HNCNT exhibited extremely high selectivity to **2a** compared to the Pt/HNCNT and Pt/CN samples. Obviously, PtZn/HNCNT catalyzed the hydrogenation of 4-NPA in different routes to Pt/HNCNT and Pt/CN. With isolating contiguous Pt and forming Pt-Zn bridge site, the PtZn/HNCNT can distinctively hydrogenate the nitro group in 4-NPA, achieving a high selectivity of **2a**."

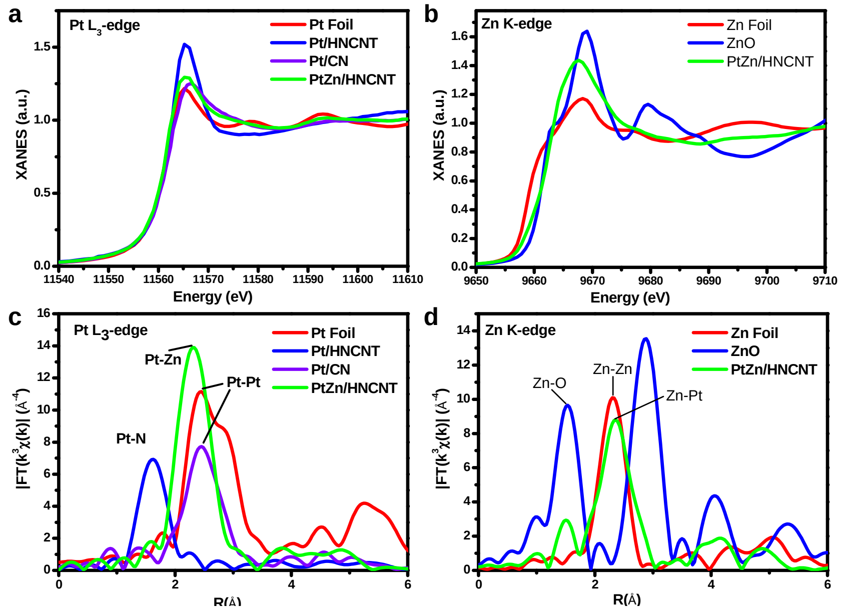

Fig. 2 | X-ray absorption spectroscopy characterization of the catalysts. a,c, The normalized XANES spectra (a) and FT-EXAFS (c) at the Pt L_3-edge of the PtZn/HNCNT, Pt/HNCNT, Pt/CN and Pt foil. b,d, The normalized XANES spectra (b) and FT-EXAFS (d) at the Zn K-edge of the PtZn/HNCNT and Zn foil.

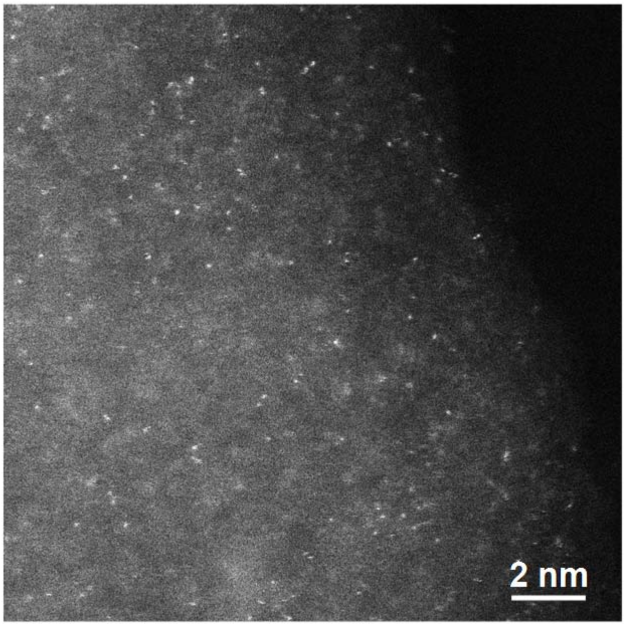

Supplementary Figure 12. AC HAADF STEM image Pt/HNCNT.

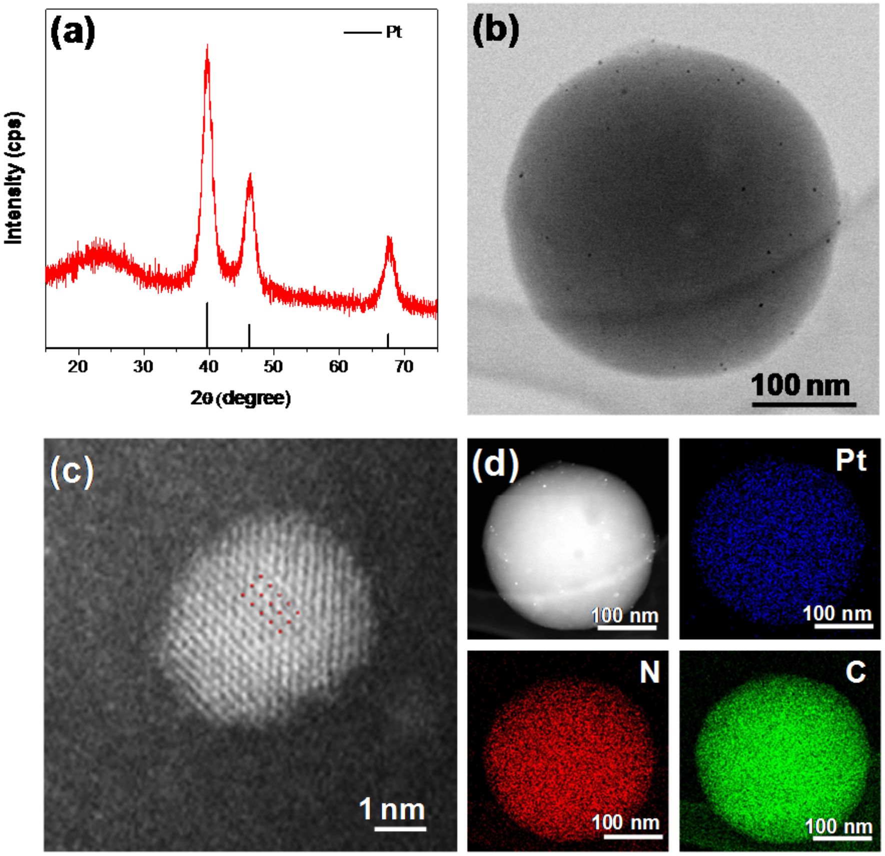

Supplementary Figure 13. Characterizations of the Pt/CN catalyst. XRD (a), TEM (b), AC HAADF STEM image (c), and elemental mappings (d) of Pt/CN.

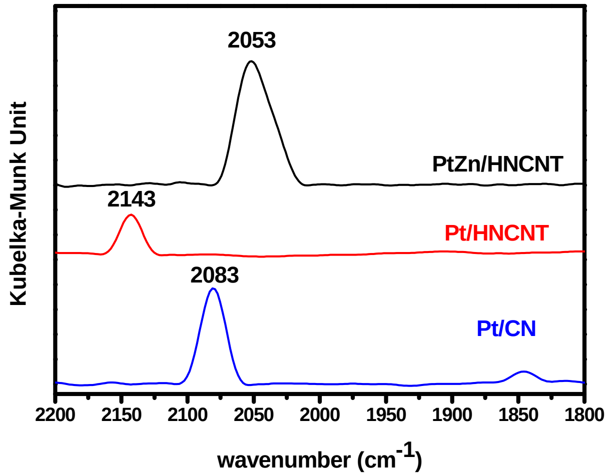

Supplementary Figure 19. *In situ* FTIR spectra of CO chemisorption for PtZn/HNCNT, Pt/HNCNT, and Pt/CN.

Reviewer #2:

The responses to the comments are mostly satisfactory and the manuscript has been improved a lot after the revision. There are two issues, which still need the author to consider carefully before publication.

Comment 1. *The significance in term of synthesis and catalytic performance of PdZn is ok; however, the bifunctional effect at the Pt-Zn bridge is rather old topic, which is not new at all.*

Response:

We appreciate the reviewer's valuable comment, which help us to improve the quality and theme of our work. We are sorry that we didn't write clearly enough in the former manuscript. In order to demonstrate the significance of our work more clearly, we have carefully revised our manuscript (*please see: paragraph beginning in Page 7, 1st paragraphs, highlighted in* ***RED***).

Here, we want to express the significance of our work from the following three aspects.

(1) The synthetic strategy to fabricate PtZn intermetallic nanoparticles in this paper is a novel and interesting strategy. Actually, this strategy is a general strategy to construct ultra-small Zn based intermetallic nanoparticles. To prove this generality, we further extend this strategy to PdZn intermetallic nanoparticles using Na_2PdCl_4 as the Pd source. PdZn/HNCNT is successfully obtained (The characterizations are given as Supplementary Figure 14 in page 13 of the Supplementary Information). A description is added in paragraph 1 page 7 of the revised manuscript to emphasize the generality of this synthetic strategy.

"Using the similar method, PdZn/HNCNT with PdZn intermetallic nanoparticles supported on HNCNT was successfully obtained (its characterizations are shown in Supplementary Fig. 14), proving the generality of this strategy to synthesize intermetallic compounds."

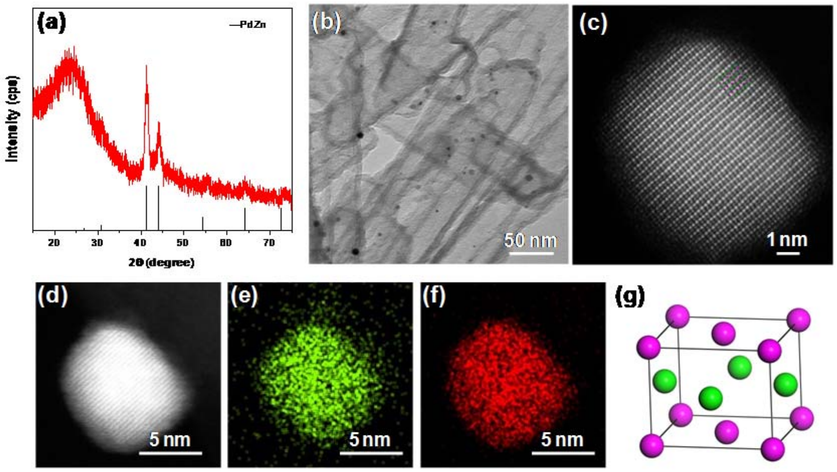

Supplementary Figure 14. Characterizations of the PdZn/HNCNT. XRD (a), TEM (b), AC HAADF STEM image (c), and elemental mappings (d-f) of PdZn/HNCNT. Crystal structure of PdZn intermetallic compound (g).

(2) The hydrogenation of 4-nitrophenylacetylene to 4-aminophenylacetylene is a rather challenging reaction as the alkynyl group are easily hydrogenated by Pt catalysts. In this work, we found that isolating contiguous Pt and forming Pt-Zn bridge sites can selectively hydrogenate the amino group in the presence of alkynyl group. It offers new application of PtZn intermetallic compounds in selective catalytic reactions.

(3) The bifunctional effect at the Pt-Zn bridge is indeed an old topic. Recently, single atom catalysis has attracted extensive research attention and rapidly rises up as a research hotspot in catalysis. In this work, we find that the Pt isolated-single-atomic-site catalyst (Pt/HNCNT) has poor selectivity. Instead, PtZn/HNCNT, where contiguous Pt atoms are isolated by Zn atoms and forming Pt-Zn bridge sites gave a high selectivity to 4-aminophenylacetylene. This work finds that single atom catalysis doesn't work in every condition. In the selective hydrogenation of 4-nitrophenylacetylene, isolating Pt into single-atomic-sites is not enough to bear high selectivity. The neighboring Zn atoms, which form Pt-Zn bridge sites with Pt atoms, also play important roles in the catalytic process. This work sheds light on the way to obtain efficient noble-metal catalysts through isolating the contiguous noble metal atoms and forming noble metal-non noble metal bridge sites in distinct intermetallic compounds.

Comment 2. *It is not straightforward to understand why the Pt segregation on PtZn alloy is not preferred. Should Pt have lower surface energy than Zn? The fundamental understanding based on theory should be included. In addition, even if Zn stay on the surface, will it be stable under reaction conditions? Will Zn to ZnO transition occur? Is there any experimental evidence on that?*

Response:

We sincerely appreciate your insightful comment. We will answer your questions from the following aspects.

(1) Segregation is indeed a common phenomenon for alloys (Phys. Rev. B, 209, 79, 094112; Nat. Mater., 2013, 12, 765; J. Phys. Chem. C, 2008, 112, 10242). Pt segregation does occur on Pt-based alloys easily (Nat. Mater., 2013, 12, 765; Nat. Mater., 2016, 15, 1188). However, intermetallic compounds, possessing distinct crystal structures with highly ordered atomic arrangements, are more stable in nature and show different properties to the traditional alloys. Therefore they have received more research attention in the past decades (ACS Catal., 2017, 7, 75). Segregation is not easy to take place for intermetallic compounds. The PtZn nanoparticles synthesized in our work are PtZn intermetallic compounds with fixed molar ratio (Pt:Zn is 1:1) and distinct crystal structure, which are stable in nature. Firstly, we want to express why PtZn intermetallic compound is stable and segregation is not preferred based on theory.

The surface energies of Pt and Zn were previous reported (Surf. Sci., 1998, 411, 186), and listed below. We can see that Pt has higher surface energy than Zn.

Table R2-1. Surface energies of Pt and Zn.

	surface	Calculated Surface energy (J/m^2)	Experimental surface energy (J/m^2)
Pt	(111)	2.299	2.489,2.475
	(100)	2.734	
	(110)	2.819	
Zn	(0001)	0.989	0.993,0.990

From a theoretical view, neither Pt nor Zn segregation on the PtZn intermetallic compound is preferred, which was discussed in a previous report comprehensively (Phys. Rev. B, 2003, 68, 075417). Through calculation, they found that "c*alculated surface energies of PdZn and PtZn alloys show that the surfaces exposing stoichiometric layers are more stable than surfaces comprising alternating Pd (Pt) and Zn layers. The surface energy values of alloys lie between the surface energies of the individual components, but they differ from their composition weighted averages. Compared with the pure metals, the valence d-band widths and the Pd or Pt partial densities of states at the Fermi level are dramatically reduced in PdZn and PtZn alloys.*"

What's more, we also conducted theoretical calculations to prove that segregation is not easy to occur on the PtZn intermetallic compound. In the 1st-round of reply, we calculated the relative energy for Pt-segregation on PtZn alloy based on the comment 2 of your respected reviewer #2, and found that the Pt segregation is not preferred (please see Figure R2-1). In this 2nd-round response, oxygen induced surface segregation is additionally calculated based on your concern about stability under reaction condition. Since previous studies have determined that surface segregation can be substantially modified by the presence of adsorbates (Phys Chem Chem Phys, 2012, 14, 7448-7455; Surf Sci 1991, 247, L215-L220; Surf Sci 1992, 261, 342-348;

Phys Chem Chem Phys, 2009, 11, 3263-3270; Chem Phys Lett 2012, 521, 98-103; Chem Phys Lett 2011, 503, 97; Phys Rev B 2005, 72, 205409), the pristine PtZn($02\bar{2}$) together with the Zn-segregated PtZn($02\bar{2}$) and Pt-segregated PtZn($02\bar{2}$) slab models are considered here in the presence of oxygen. The most stable oxygen adsorption configuration is the pristine PtZn ($02\bar{2}$), as shown in Figure R2-2.

Based on the above theory, we deduce that PtZn intermetallic compounds are stable, neither Pt segregation nor Zn segregation occurs, even in the presence of oxygen.

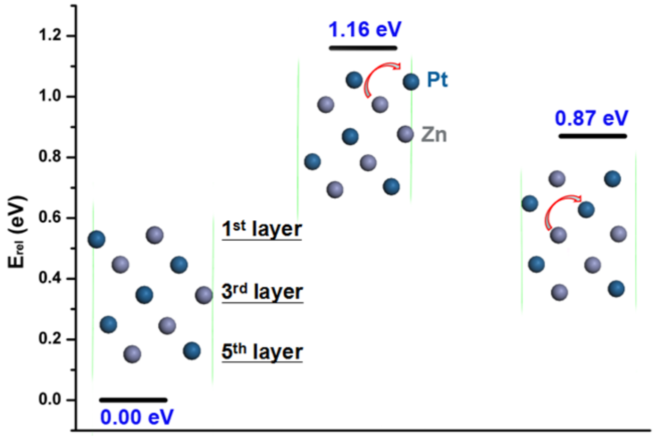

Figure R2-1. Structures and relative energies of PtZn($02\bar{2}$) surface and the considered segregation configurations with Pt monolayer.

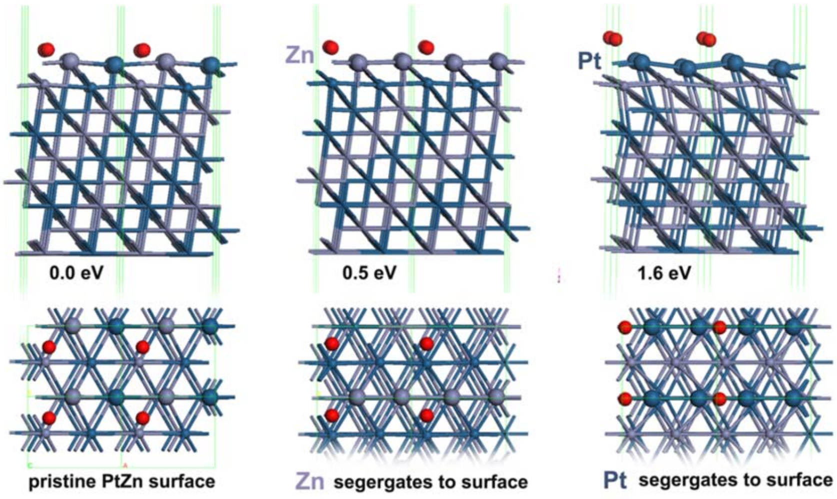

Figure R2-2. Surface segregation of Zn and Pt in the presence of oxygen.

(2) Secondly, we find experimental evidences that segregation is not preferred for the PtZn/HNCNT sample. Zn atoms stay on the surface within the crystal structure of PtZn intermetallic compounds instead of forming metallic zinc layer. It is stable under catalytic reactions and its transformation to ZnO is not easy to occur as the Pt-Zn bond energy is high. We did the in situ CO FTIR for PtZn/HNCNT. It showed

typical linearly CO bonds on Pt atoms at 2053 cm^{-1}, indicating that zinc segregation doesn't occur (please see Supplementary Figure 19 shown below). And the red shift of the CO linear bonds for PtZn/HNCNT compared to Pt/CN (with Pt nanoparticles) also indicates that Pt segregation doesn't happen. Besides, the CO linear bonds positions of PtZn/HNCNT before and after reactions are nearly the same, indicating that no ZnO or Pt segregation occurs during the catalytic process (please see Supplementary Figure 31 shown below). These experimental evidences testify that PtZn intermetallic compound is formed without Pt or Zn segregation, and it is stable under reaction condition.

Taken the theory and experimental evidences together, we prove that PtZn intermetallic nanoparticles synthesized in this paper is stable and no segregation occur.

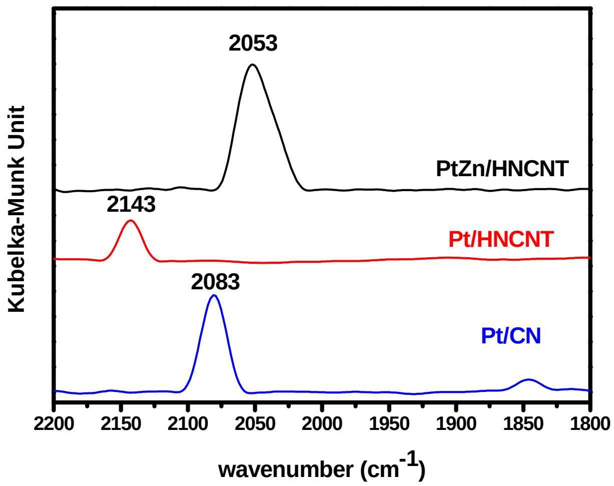

Supplementary Figure 19. *In situ* FTIR spectra of CO chemisorption for PtZn/HNCNT, Pt/HNCNT, and Pt/CN.

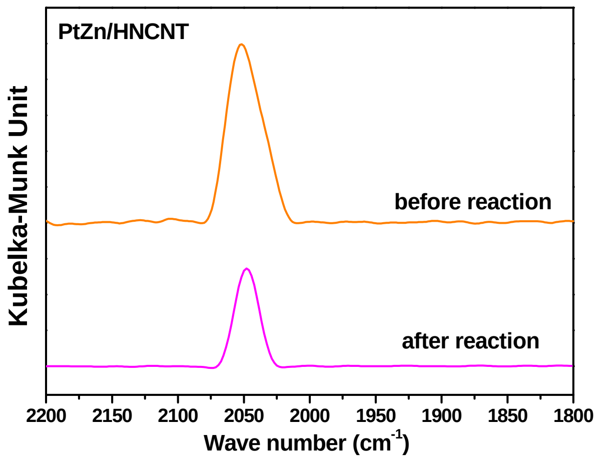

Supplementary Figure 31. *In situ* FTIR spectra of CO chemisorption of

PtZn/HNCNT before and after the reaction.

Reviewer #3:

The authors have made minor modifications to the paper, including only a few ex situ characterizations (which do not provide further insights) and a substrate scope analysis. All major comments raised by Reviewers #2 and #3 during the first round of Peer Review on the surface segregation of metal species, core-shell synthesis, and microkinetic analysis have remained unaddressed.

Response:

We appreciate your comments very much. We are sorry that some points are not well addressed in the 1st-round response. We give more responses regarding the three aspects your respected reviewer concerned.

(1) In respect to the surface segregation of metal species, we give more theoretical proof and experimental evidences to show that segregation is not easy to occur, which will be discussed in detail as follows.

① Segregation is indeed a common phenomenon for alloys (Phys. Rev. B, 209, 79, 094112; Nat. Mater., 2013, 12, 765; J. Phys. Chem. C, 2008, 112, 10242). Pt segregation does occur on Pt-based alloys easily (Nat. Mater., 2013, 12, 765; Nat. Mater., 2016, 15, 1188). However, intermetallic compounds, possessing distinct crystal structures with highly ordered atomic arrangements, are more stable in nature and show different properties to the traditional alloys. Therefore they have received more research attention in the past decades (ACS Catal., 2017, 7, 75). Segregation is not easy to take place for intermetallic compounds. The PtZn nanoparticles synthesized in our work are PtZn intermetallic compounds with fixed molar ratio (Pt:Zn is 1:1) and distinct crystal structure, which are stable in nature. Firstly, we want to express why PtZn intermetallic compound is stable and segregation is not preferred based on theory.

The surface energies of Pt and Zn were previous reported (Surf. Sci., 1998, 411, 186), and listed below. We can see that Pt has higher surface energy than Zn.

Table R2-1. Surface energies of Pt and Zn.

	surface	Calculated Surface energy (J/m^2)	Experimental surface energy (J/m^2)
Pt	(111)	2.299	2.489,2.475
	(100)	2.734	
	(110)	2.819	
Zn	(0001)	0.989	0.993,0.990

From a theoretical view, neither Pt nor Zn segregation on the PtZn intermetallic compound is preferred, which was discussed in a previous report comprehensively (Phys. Rev. B, 2003, 68, 075417). Through calculation, they found that "c*alculated surface energies of PdZn and PtZn alloys show that the surfaces exposing stoichiometric layers are more stable than surfaces comprising alternating Pd (Pt) and Zn layers. The surface energy values of alloys lie between the surface energies of the individual components, but they differ from their composition weighted averages.*

Compared with the pure metals, the valence d-band widths and the Pd or Pt partial densities of states at the Fermi level are dramatically reduced in PdZn and PtZn alloys.”

What’s more, we also conducted theoretical calculations to prove that segregation is not easy to occur on the PtZn intermetallic compound. In the 1st-round of reply, we calculated the relative energy for Pt-segregation on PtZn alloy based on the comment 2 of your respected reviewer #2, and found that the Pt segregation is not preferred (please see Figure R2-1). In this 2nd-round response, oxygen induced surface segregation is additionally calculated based on your concern about stability under reaction condition. Since previous studies have determined that surface segregation can be substantially modified by the presence of adsorbates (Phys Chem Chem Phys, 2012, 14, 7448-7455; Surf Sci 1991, 247, L215-L220; Surf Sci 1992, 261, 342-348; Phys Chem Chem Phys, 2009, 11, 3263-3270; Chem Phys Lett 2012, 521, 98-103; Chem Phys Lett 2011, 503, 97; Phys Rev B 2005, 72, 205409), the pristine PtZn($02\bar{2}$) together with the Zn-segregated PtZn($02\bar{2}$) and Pt-segregated PtZn($02\bar{2}$) slab models are considered here in the presence of oxygen. The most stable oxygen adsorption configuration is the pristine PtZn ($02\bar{2}$), as shown in Figure R2-2.

Based on the above theory, we deduce that PtZn intermetallic compounds are stable, neither Pt segregation nor Zn segregation occurs, even in the presence of oxygen.

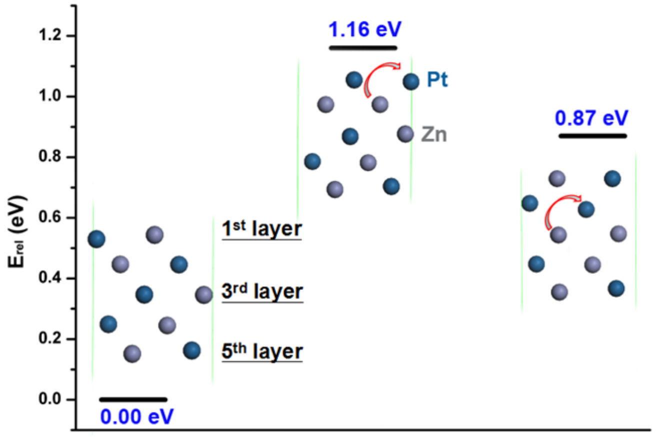

Figure R2-1. Structures and relative energies of PtZn($02\bar{2}$) surface and the considered segregation configurations with Pt monolayer.

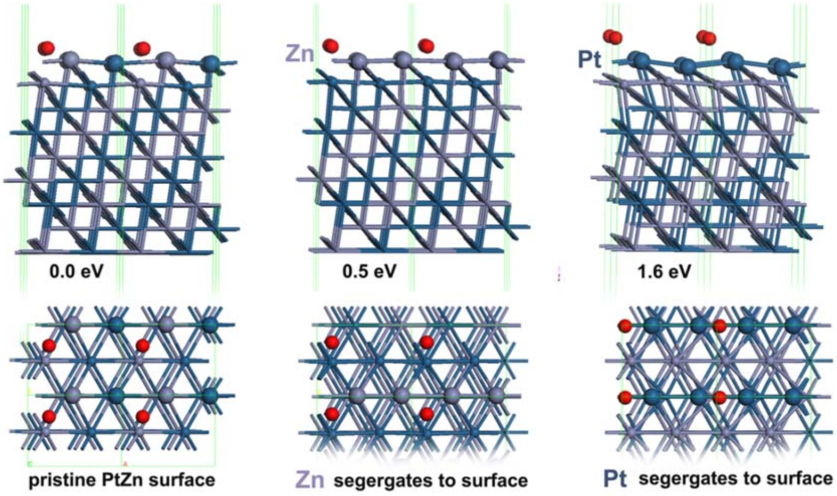

Figure R2-2. Surface segregation of Zn and Pt in the presence of oxygen.

② Secondly, we find experimental evidences that segregation is not preferred for the PtZn/HNCNT sample. Zn atoms stay on the surface within the crystal structure of PtZn intermetallic compounds instead of forming metallic zinc layer. It is stable under catalytic reactions and its transformation to ZnO is not easy to occur as the Pt-Zn bond energy is high. We did the in situ CO FTIR for PtZn/HNCNT. It showed typical linearly CO bonds on Pt atoms at 2053 cm^{-1}, indicating that zinc segregation doesn't occur (please see Supplementary Figure 19 shown below). And the red shift of the CO linear bonds for PtZn/HNCNT compared to Pt/CN (with Pt nanoparticles) also indicates that Pt segregation doesn't happen. Besides, the CO linear bonds positions of PtZn/HNCNT before and after reactions are nearly the same, indicating that no ZnO or Pt segregation occurs during the catalytic process (please see Supplementary Figure 31 shown below). These experimental evidences testify that PtZn intermetallic compound is formed without Pt or Zn segregation, and it is stable under reaction condition.

Taken the theory and experimental evidences together, we prove that PtZn intermetallic nanoparticles synthesized in this paper is stable and no segregation occur.

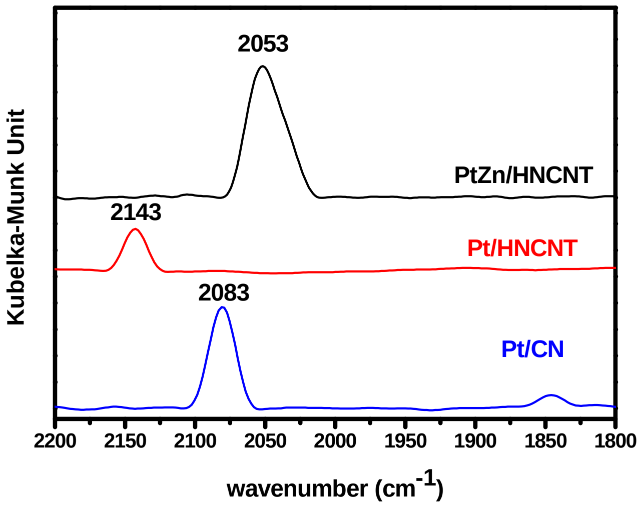

Supplementary Figure 19. *In situ* FTIR spectra of CO chemisorption for PtZn/HNCNT, Pt/HNCNT, and Pt/CN.

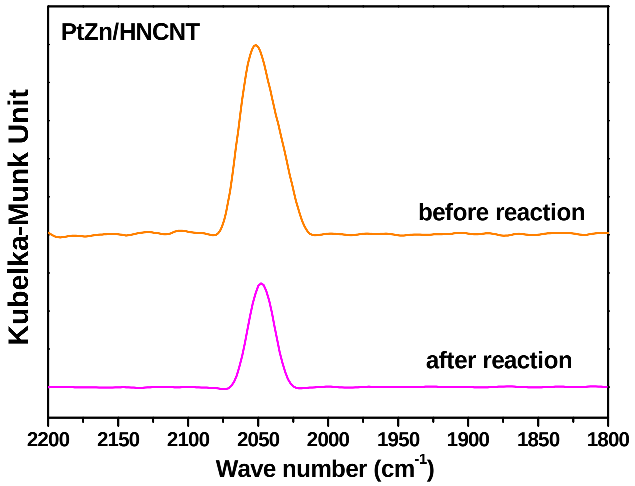

Supplementary Figure 31. *In situ* FTIR spectra of CO chemisorption of PtZn/HNCNT before and after the reaction.

(2) In respect to the core-shell synthesis, we are sorry that we failed to prepare the core-shell structures as suggested by your respected reviewer. In fact, we had groped extensive routes without stop to prepare the core-shell structures during the 1st-round reply, but failed until the deadline of the 1st-round response because the preparation of such kind of structures is not available and we have to explore various ways to synthesize them. It is really not an easy task. Since the deadline for the 1st-round response approached, we had to use substitutions of the core-shell structured samples reluctantly to provide indirect evidences. Considering that the shell plays major roles in catalytic process as reactions take place on the surface of catalysts, we

tested the activity of ZnO to substitute Pt core-Zn shell structures as zinc nanoparticles are easily oxidized, and we tested the activity of Pt nanoparticles and Pt_3Zn with enriched Pt on the surface to substitute Zn core-Pt shell structures. The poor selectivity to 4-aminophenylacetylene of these catalysts indicates that isolating Pt atoms and forming Pt-Zn intermetallic compounds are important for the high selectivity to 4-aminophenylacetylene. During the period for 2nd-round response, we have taken extensive times continuously to grope the possible routes to synthesize the core-shell structures. Since nanosized Zn are easily oxidized into ZnO, rendering it difficult to synthesize. We only successfully obtained Pt core-ZnO shell and ZnO core-Pt shell structures (TEM images are shown as Figure R3-1) until the deadline of this round response. Since there are no literatures on the synthesis of Pt core-Zn shell or Zn core-Pt shell structures, it is not an easy task to fabricate these kinds of structures for us through keeping trying. The catalytic activities for those Pt core-ZnO shell and ZnO core-Pt shell have been tested, and both of them exhibit poor selectivity to 4-aminophenylacetylene, with the 4-nitrostyrene and 4-aminoethylbenzene as the main product for Pt core-ZnO shell structure and ZnO core-Pt shell structure respectively.

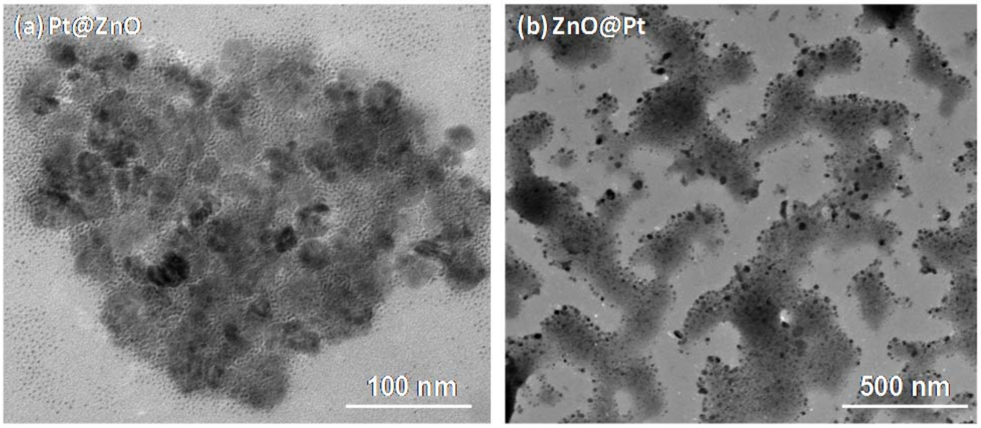

Figure R3-1. TEM images of Pt core-ZnO shell (a) and ZnO core-Pt shell (b).

(3) In respect to the microkinetic analysis, we have given responses regarding the comments raised by your respected reviewer in the Response to Comment 9 Reviewer 3 in the 1st- round response. We investigated the effect of temperature and pressure, as well as the kinetic profile of PtZn/HNCNT. The responses are given bellow again for your convenience. We hope that the responses are satisfied to your respected reviewer.

"(2) The kinetic curve is added in Figure S18. A discussion is added in page 9.

"The kinetic profile as followed and shown in Supplementary Figure 18. The conversion increased fast in the first 2 h while the selectivity kept 99 % to **2a**."

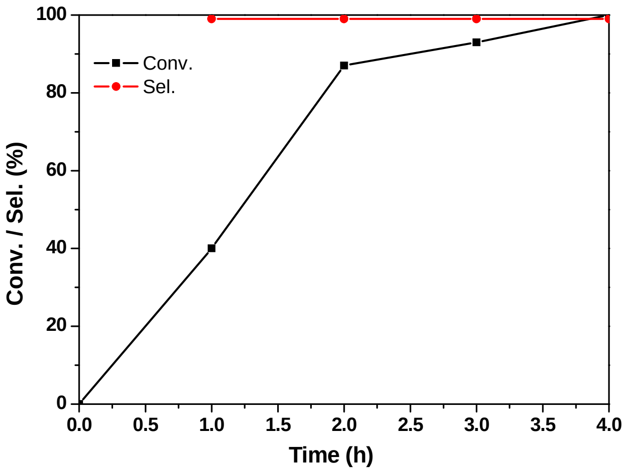

Supplementary Figure 18. Kinetic profile of 4-nitrophenylacetylene hydrogenation over PtZn/HNCNT.

(3)The effect of temperature and pressure is added in Table S1. A discussion is added in page 9. "Different reaction conditions was also studied and shown in Supplementary Table 1. The highest selectivity was obtained at 40 ^{o}C. Since the reaction was carried out in the pressure tube, a larger volume of tube was used to reduce the pressure during the reaction. However, a decreased selectivity was observed."

Supplementary Table 1. Effect of temperature and pressure in the hydrogenation of 4-nitro phenylacetylene over the PtZn/HNCNT catalyst.

Entry	Temp (^{o}C)	Time (h)	Tube Vol (ml)	Conv.(%)	Sel.(%)
1	40	4	15	100	98.0
2	60	2	15	99.0	76.5
3	70	1	15	99.5	70.0
4	40	4	35	100	90.0

Condition: 4-nitrophenylacetylene 0.5 mmol, ammonia borane 3 mmol, catalyst 2.0 mg, ethanol 4.9 ml, deionized water 0.1 ml, reaction was carried out in a pressure tube."

Comment 1: *In the replies, the authors write: "we have tried our best to consider the full reaction path for both Pt and PtZn systems. The thermochemistry-only potential energy pathways were constructed (J. Phys. Chem. C, 10.1021/acs.jpcc.7b11629 (2018); J. Phys. Chem. B, 122, 432–443 (2018))". These two papers have nothing to do with the reduction of nitroaromatics to anilines, as they deal with the mechanism of nitric oxide reduction to nitrogen. The full reaction scheme (which is as well unreadable) is hence questionable.*

Response:

We appreciate your valuable comment very much. We are sorry that we didn't write clearly enough, causing misunderstanding of our paper. The two papers cited are examples of constructing the thermochemistry-only potential energy pathways to

analyze the selective catalytic reduction of NO_x. In our case, several potential hydrogenation targets times several hydrogen adsorption sites lead a considerably complex mesh. In this regard, the thermochemistry-only energy surface approach in those two papers gives us inspiration of constructing thermochemistry-only potential energy pathways in the case of complicated full reaction path. To avoid the misleading, we deleted the citation of these two papers in 1st paragraph line 1 page 14.

"The thermochemistry-only potential energy pathways were constructed (Supplementary Figs. 37-46)."

Comment 2: *In the Supporting Info, the authors have included a scheme of the possible reaction path, but have not conducted a full energy analysis of the different steps by DFT (as requested by Reviewers #2 and #3), indicating for instance which elementary steps are critical to the reaction. Thus, there is still no fundamental understanding of the process in the work.*

Response:

We thank the reviewer for the constructive comment, which help us to improve the quality of our paper a lot.

As you suggested, in order to provide fundamental understanding of the reaction process, we conducted a full energy analysis of the different steps proposed in Supplementary Figure 49a and add the results about relative energies as Supplementary Figure 49b in this revised manuscript. CH3-CH2-C6H4-NHO to CH3-CH2-C6H4-N is the only endothermic step. The description in 1st paragraph page 14 of the main text is revised to clearly state the fundamental understanding of the process.

"Based on the direct hydrogenation path nitro group, the barriers of first hydrogenation step on alkynyl group for CH-C-C_6H_4-NOOH, CH-C-C_6H_4-NO, CH-C-C_6H_4-NHO, CH-C-C_6H_4-N, CH-C-C_6H_4-NH and CH-C-C_6H_4-NH_2 on alloy surface were also calculated (Supplementary Figs. 49-58). We can find that every reaction needs to climb over a barrier higher than 200 kJ/mol, which is difficult to trigger the subsequent hydrogenation reactions on the alkynyl group. We considered that such high reaction barrier on alkynyl part is the essential reason for the high selectivity on PtZn surface."

To make the reaction pathway more readable, we add some blue boxes to mark the intermediates with lower relative energies, and add figures about the corresponding intermediates in Supplementary Figures 51-58. Besides, the figures regarding the intermediates for the reaction path on Pt (111) are also added as Supplementary Figures 39-46.

Those figures added in this revised version are shown as follows.

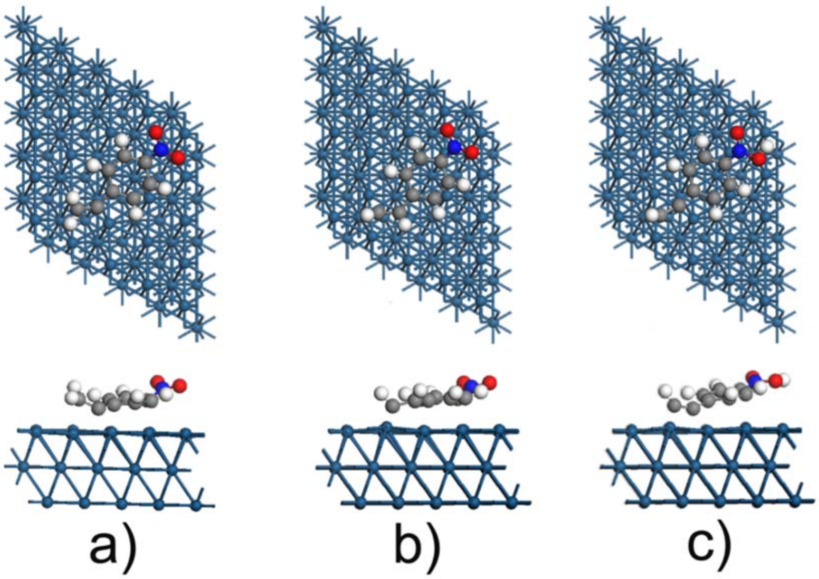

Supplementary Figure 39. Optimized structures of intermediates for 4-NPA 1st hydrogenation reaction step over Pt(111) surface.

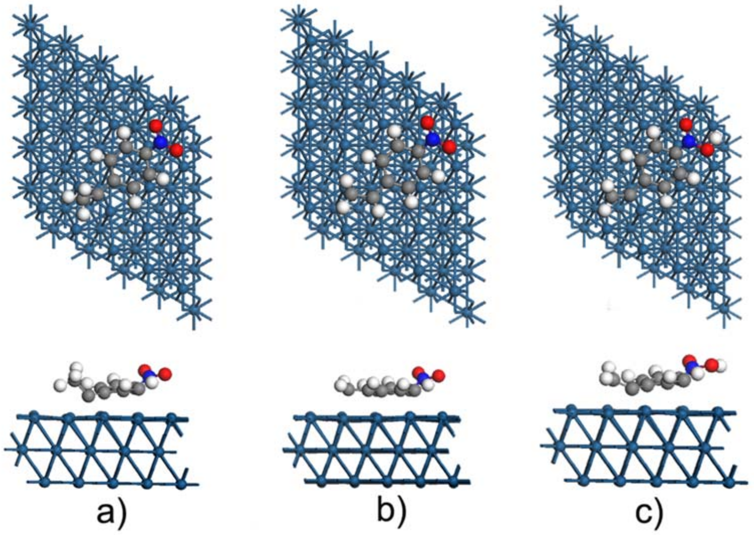

Supplementary Figure 40. Optimized structures of intermediates for 4-NPA 2nd hydrogenation reaction step over Pt(111) surface.

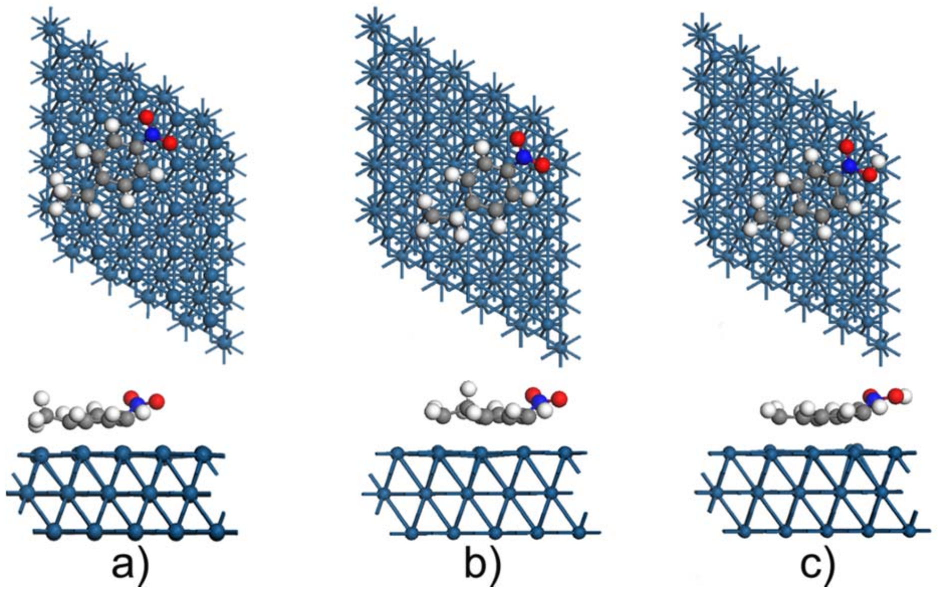

Supplementary Figure 41. Optimized structures of intermediates for 4-NPA 3rd hydrogenation reaction step over Pt(111) surface.

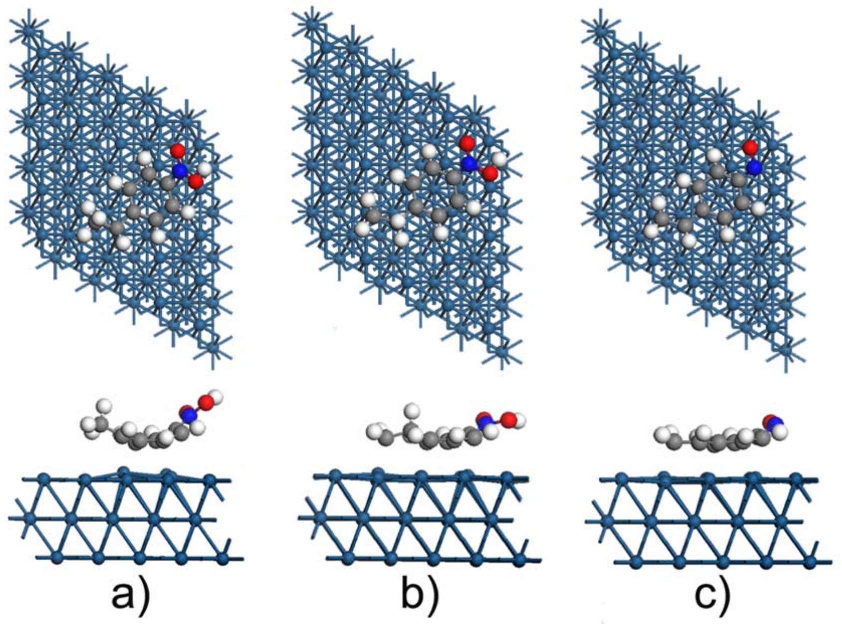

Supplementary Figure 42. Optimized structures of intermediates for 4-NPA 4th hydrogenation reaction step over Pt(111) surface.

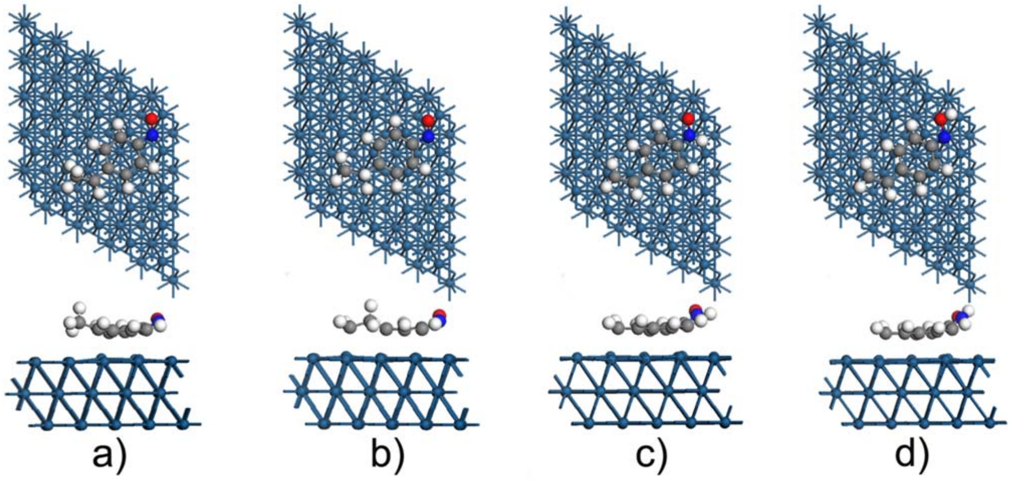

Supplementary Figure 43. Optimized structures of intermediates for 4-NPA 5th hydrogenation reaction step over Pt(111) surface.

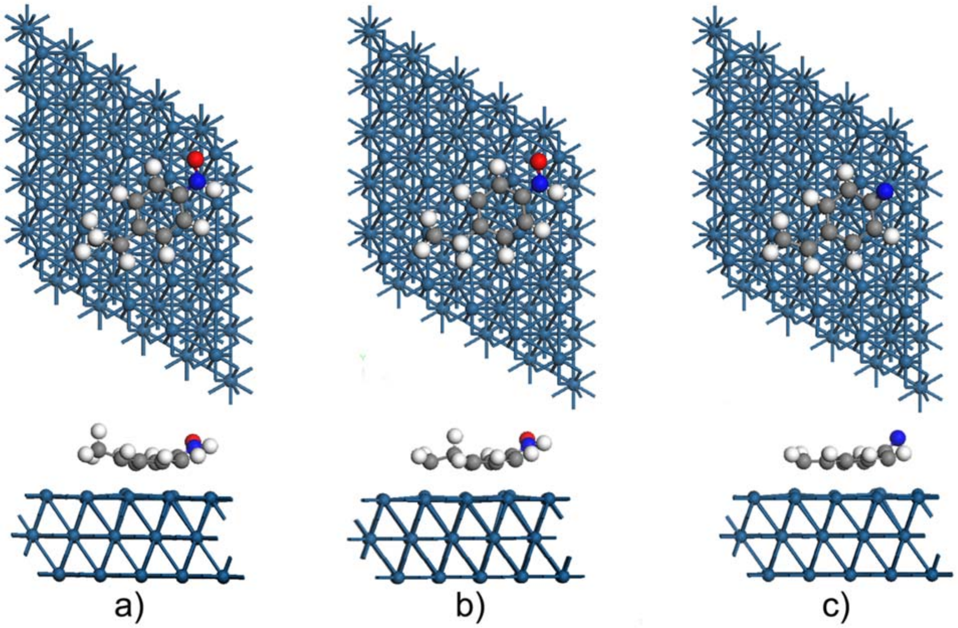

Supplementary Figure 44. Optimized structures of intermediates for 4-NPA 6th hydrogenation reaction step over Pt(111) surface.

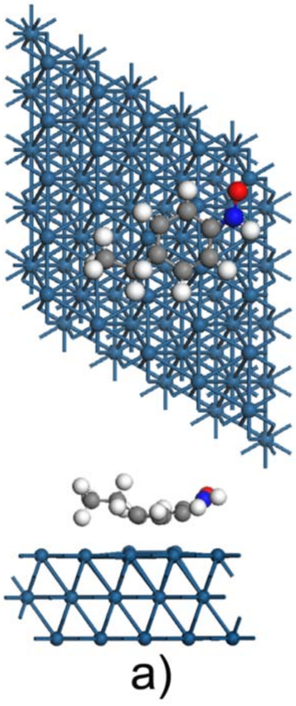

Supplementary Figure 45. Optimized structures of intermediates for 4-NPA 7th hydrogenation reaction step over Pt(111) surface.

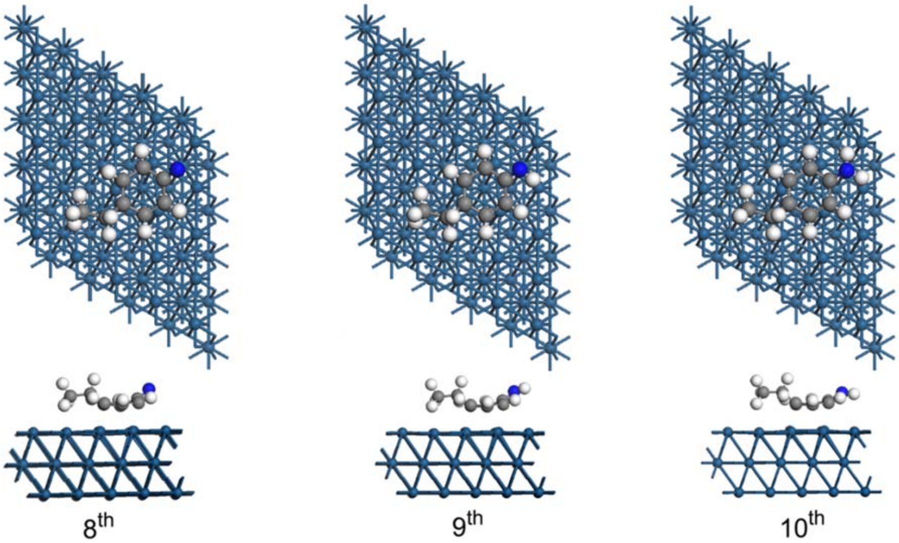

Supplementary Figure 46. Optimized structures of intermediates for 4-NPA 8th, 9th and 10th hydrogenation reaction step over Pt(111) surface.

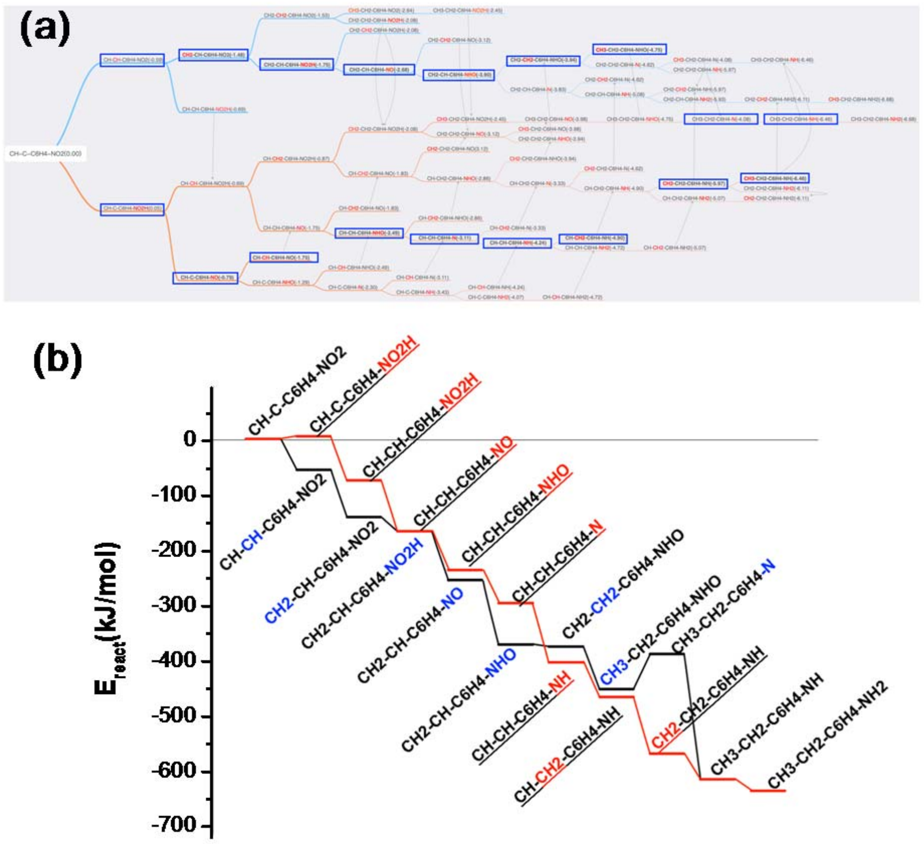

Supplementary Figure 49. (a) The simplified hydrogenation reaction pathway and the corresponding (b) reaction energies of hydrogenation reaction pathway of CH-C-C_6H_4-NO_2 on the PtZn(02$\bar{2}$) surface. The hydrogenation sites are marked in red. Blue boxes mark the intermediates with lower relative energies (unit : kJ/mol).

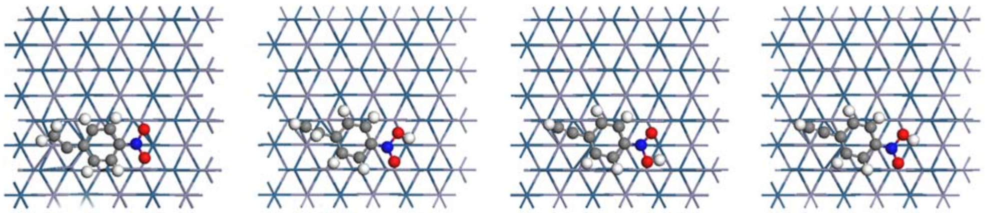

1st-a 1st-b 1st-c 1st-d

Supplementary Figure 51. Optimized structures of intermediates for 4-NPA 1st hydrogenation reaction step over PtZn(02$\bar{2}$) surface.

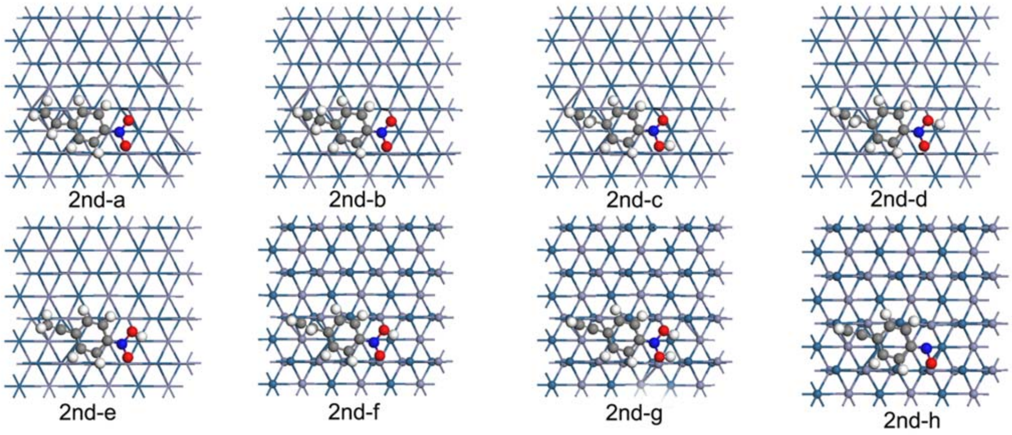

Supplementary Figure52. Optimized structures of intermediates for 4-NPA 2nd hydrogenation reaction step over PtZn($02\bar{2}$) surface.

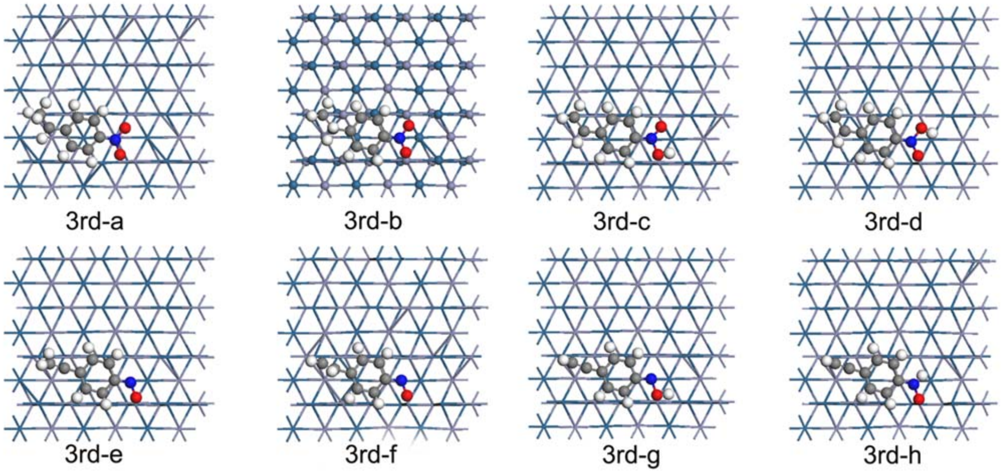

Supplementary Figure 53. Optimized structures of intermediates for 4-NPA 3rd hydrogenation reaction step over PtZn($02\bar{2}$) surface.

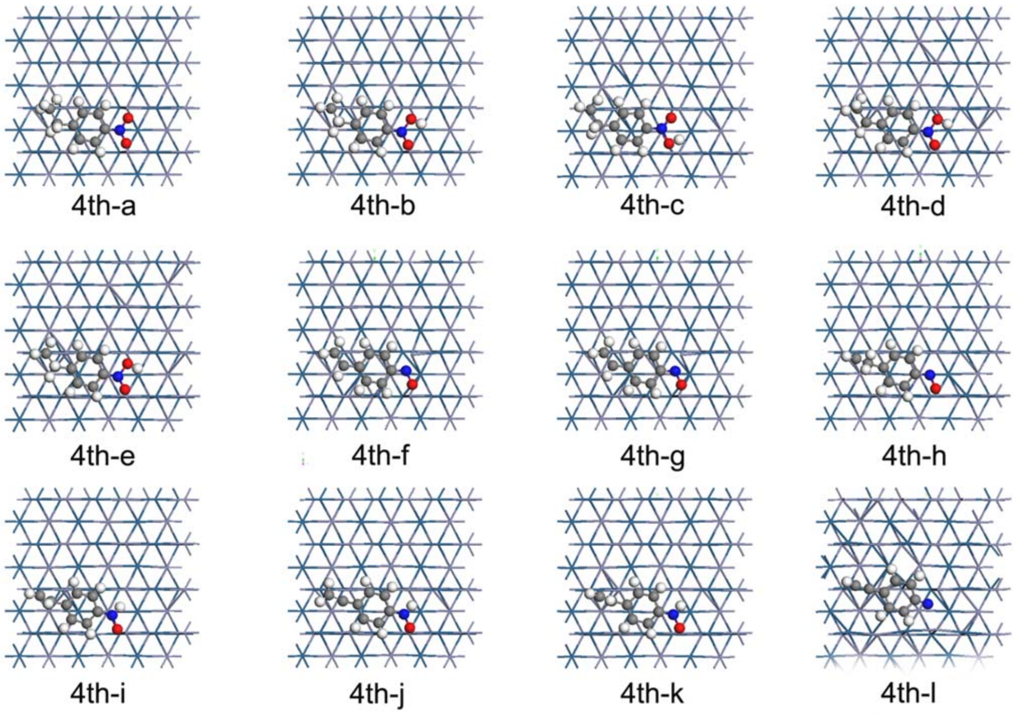

Supplementary Figure 54. Optimized structures of intermediates for 4-NPA 4th hydrogenation reaction step over PtZn($02\bar{2}$) surface.

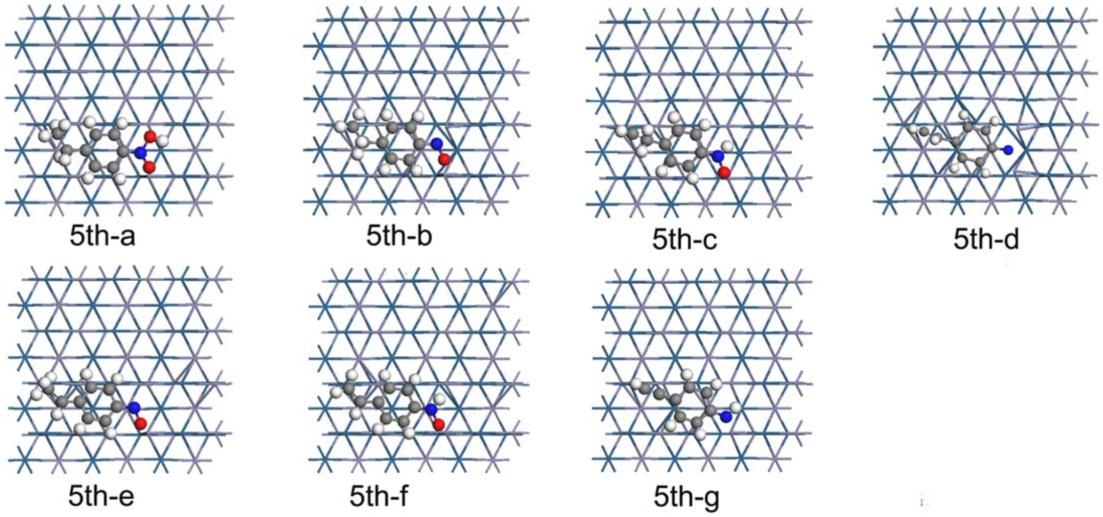

Supplementary Figure 55. Optimized structures of intermediates for 4-NPA 5th hydrogenation reaction step over PtZn($02\bar{2}$) surface.

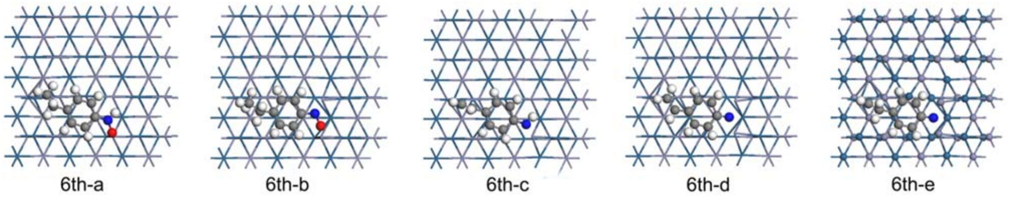

Supplementary Figure 56. Optimized structures of intermediates for 4-NPA 6th hydrogenation reaction step over PtZn($02\bar{2}$) surface.

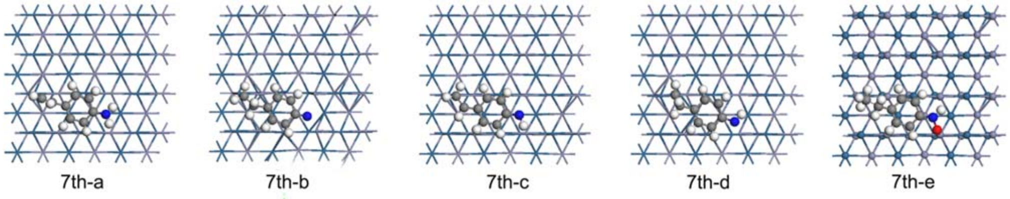

Supplementary Figure 57. Optimized structures of intermediates for 4-NPA 7th hydrogenation reaction step over PtZn($02\bar{2}$) surface.

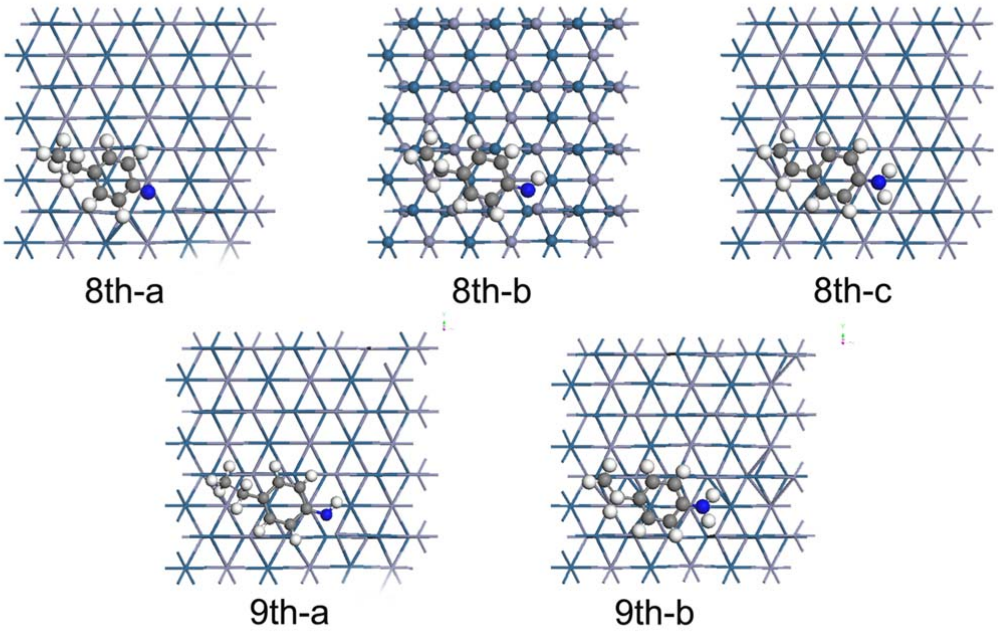

Supplementary Figure 58. Optimized structures of intermediates for 4-NPA 8th abd 9th hydrogenation reaction steps over PtZn($02\bar{2}$) surface.

Comment 3: *Many sentences in the DFT part seems to be taken from already published papers (see: Journal of Advanced Physics, 2016, 5, pp. 354-358).*

Response:

We appreciate your valuable comment very much. The computational details have many fixed expression, and the similarity are pure coincidence. We are really grateful for your pointing of the similar description in the DFT part of our paper to the published paper (Journal of Advanced Physics, 2016, 5, pp. 354-358). The description of the computational details is revised carefully in page 4-5 of the revised Supplementary Information to avoid duplication to published papers.

"The calculations were performed ultilizing the plane-wave pseodopotential method in the framework of density functional theory (DFT). The ion core and valence electron interaction was described by Vanderbilt-type ultrasoft pseudopotential. The electron exchange-correlation effects were described by the generalized gradient approximation (GGA) in the form of Perdew-Burke-Ernzerh (PBE) functional[2]. All spin-polarized density functional theory (DFT) calculations were performed on the Cambridge serial total energy package (CASTEP) program[3]. The role of the van der Waals (vdw) force should not be ignored in the considered systems. Hence, the dispersion correction included DFT-D method was used[4]. The plane-wave cutoff enrgy was set to 350 eV. The convergence thresholds between optimization cycles for energy change and maximum force were set as 10^{-5} eV/atom and 0.03 eV/Å, respectively. "

Comment 4: *The authors mention: "the hydrogenation on alkynyl group is along the perpendicular direction", which is questionable considering that catalysis happens at the surface (and the surface here is planar). Most of the theoretical figures are in kJ/mol but, in the discussion, the authors jump from eV to kJ/mol, rendering the DFT analysis unclear and suspicious.*

Response:

We thanks very much for your constructive comments, which help us to improve the quality of our manuscript. We are sorry that we didn't write clearly in our former manuscript.

To make the DFT part more clearly and readable, we revise these sentences by labeling the corresponding directions using lattice axis (<011> and <100> directions) in paragraph 1 page 14 and Supplementary Figure 47. The units in eV are converted to kJ/mol and the units are unified in the DFT analysis in paragraph 1 page 13 and paragraph 1 of page 14, as well as Supplementary Figs 34-36 & 47-48.

"….however, this configuration is 94 kJ/mol less stable. For PtZn($02\bar{2}$) surface,…."

"For 4-NPA@PtZn($02\bar{2}$), hydrogenation on nitro group is along the hydrogen atom diffusion path from Pt-Zn(bridge) to Zn-Pt(bridge) (along <011> direction, Supplementary Fig. 48). The diffusion barrier is 29.0 kJ/mol. Meanwhile, the hydrogenation on alkynyl group is along <100> direction. Hydrogen diffusion along this path is unfavorable (diffusion barrier is 143 kJ/mol)."

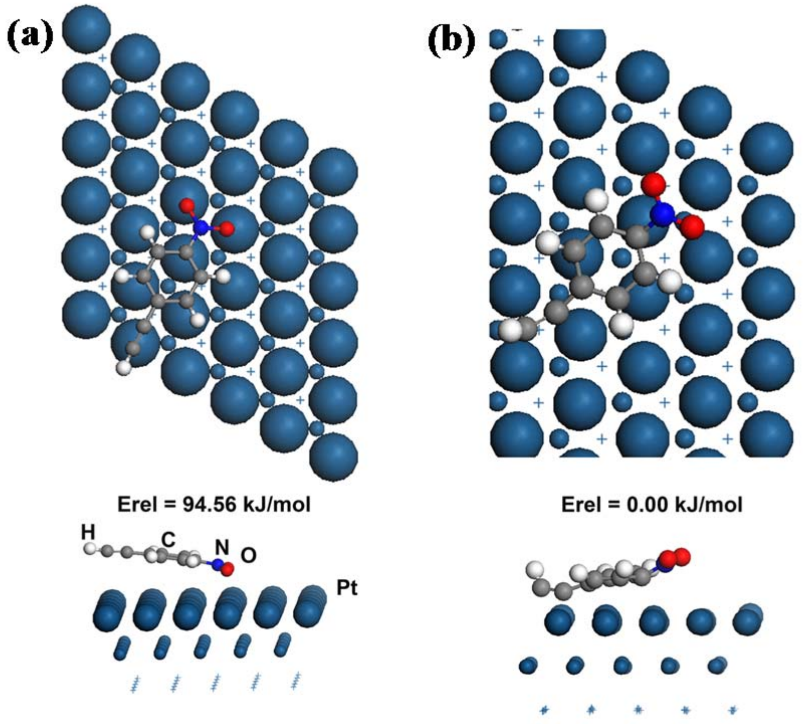

Supplementary Figure 33. Top and side view of adsorption configurations for 4-NPA@Pt(111) surface. The right configuration (b) is more stable.

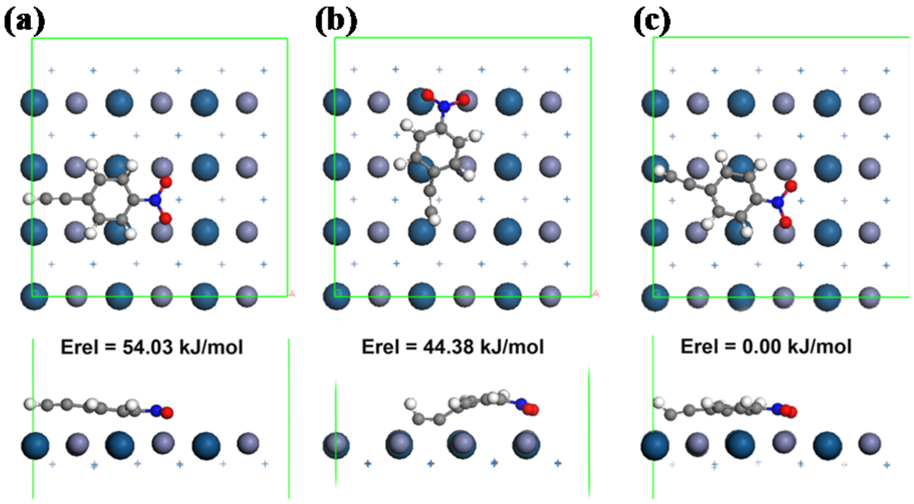

Supplementary Figure 34. Top and side view of adsorption configurations for 4-NPA@PtZn($02\bar{2}$) surface. (Zn: grey). The right configuration (c) is more stable.

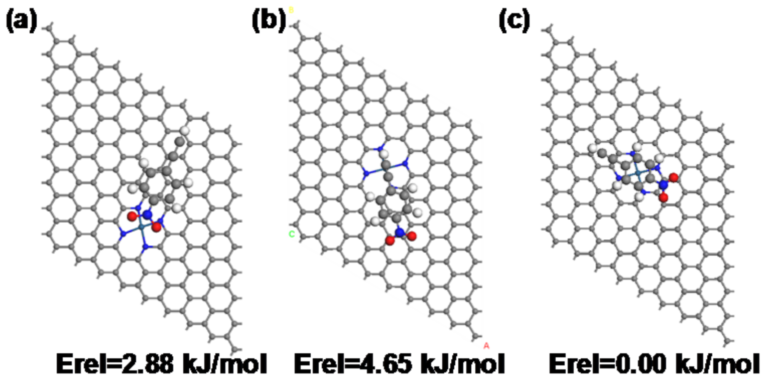

Supplementary Figure 35. Top view of adsorption configurations for 4-NPA@Pt-N-C surface. The right configuration (c) is more stable.

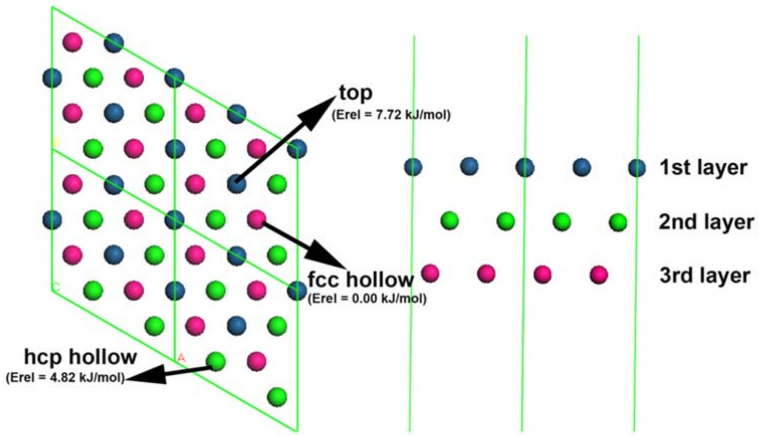

Supplementary Figure 36. Schematic illustration of the high-symmetry sites on Pt(111) surface. The values in parentheses represent the relative total energy for hydrogen atom on the site.

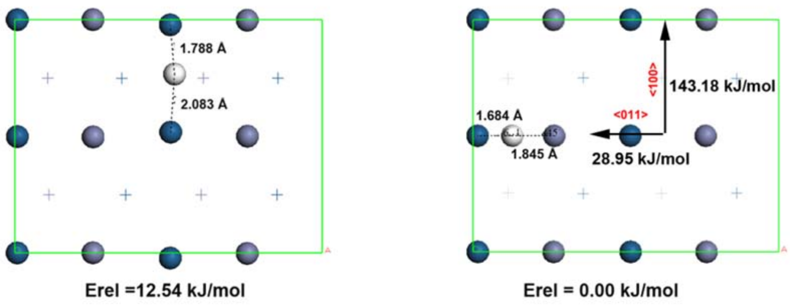

Supplementary Figure 47. Top view of adsorption configurations for H@PtZn($02\bar{2}$) surface. The right configuration is more stable. Two hydrogen atom diffusion paths together with their diffusion barriers are labeled.

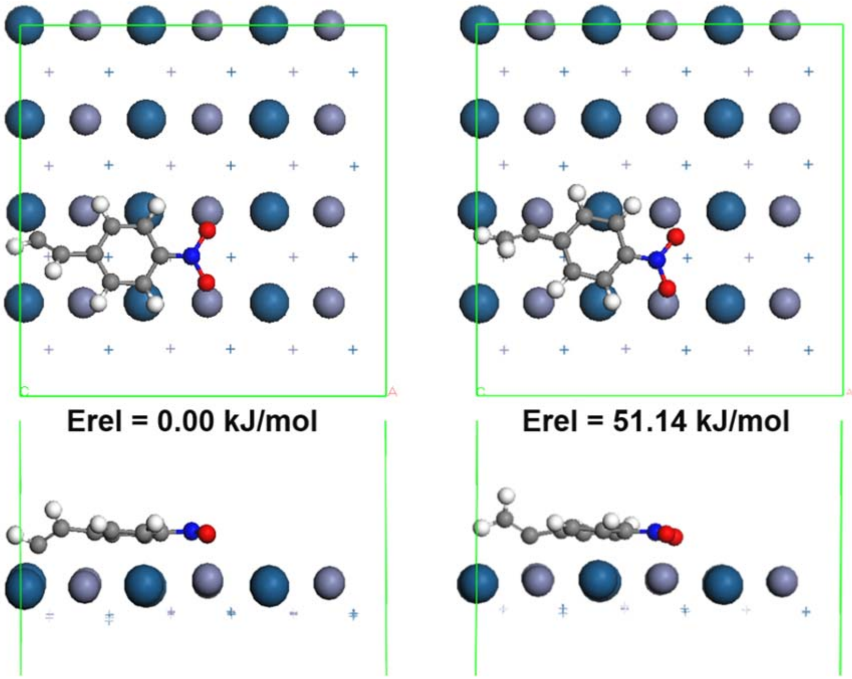

Supplementary Figure 48. Top and side view of different product configurations of the first hydrogenation step of alkynyl group for 4-NPA@PtZn($02\bar{2}$) surface. The left configuration is more stable.

Comment 5: *There have been many reports on nitro hydrogenation on PtZn systems (ACS Catal, 2016, 6, 742–746; J. Mol. Catal. 1992, 71, 317-333; ACS Catal., 2014, 4, pp 1441–1450). I do not see what the added value of this publication truly is, or what the novelty of this paper is. It will not attract enough citations.*

Response:

We appreciate the reviewer's comments. We are sorry that we did not write clearly enough in our former manuscript. In order to more clearly demonstrate the novelty of our work, we have carefully revised our manuscript (*please see: paragraph 2 page 4, and paragraph 1 page 7highlighted in* ***RED***).

Here, we want to express the novelty and highlight of our work. And, we sincerely hope that you could kindly reconsider our manuscript.

(1) The synthetic strategy to fabricate PtZn intermetallic nanoparticles in this paper is a novel and interesting strategy. Actually, this strategy is a general strategy to construct ultra-small Zn based intermetallic nanoparticles. To prove this generality, we further extend this strategy to PdZn intermetallic nanoparticles using Na_2PdCl_4 as the Pd source. PdZn/HNCNT is successfully obtained (The characterizations are given as Supplementary Figure 14 in page 13 of the Supplementary Information). This work would attract the attention of readers in the field of material science. A description is added in paragraph 1 page 7 of the revised manuscript to emphasize the generality of this synthetic strategy.

"Using the similar method, PdZn/HNCNT with PdZn intermetallic nanoparticles supported on HNCNT was successfully obtained (its characterizations are shown in Supplementary Fig. 14), proving the generality of this strategy to synthesize intermetallic compounds."

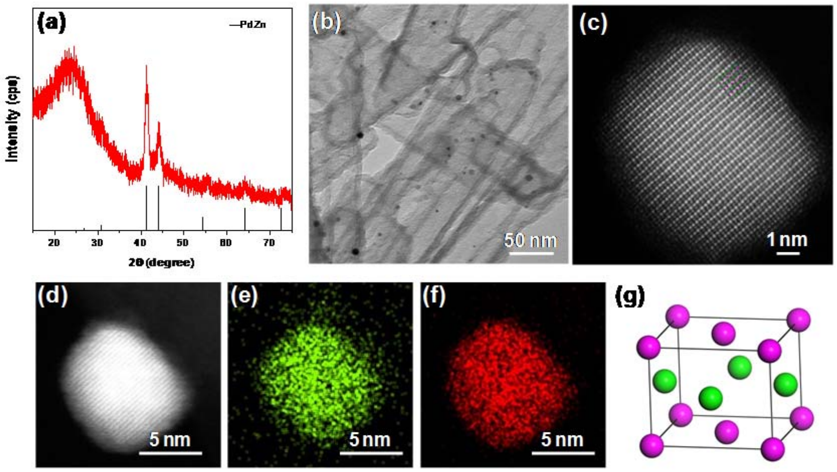

Supplementary Figure 14. Characterizations of the PdZn/HNCNT. XRD (a), TEM (b), AC HAADF STEM image (c), and elemental mappings (d-f) of PdZn/HNCNT. Crystal structure of PdZn intermetallic compound (g).

(2) In this paper, we found that by forming PtZn intermetallic compound, the catalyst show excellent selectivity in hydrogenation of 4-nitrophenylacetylene to 4-aminophenylacetylene, realizing the selective hydrogenation of nitro group in the presence of highly reducible ethynyl group. Since ethynyl group is easily to be hydrogenated in the presence of Pt catalysts, it is a challenging task realize the selective hydrogenation of 4-nitrophenylacetylene.

(3) Recently, single atom catalysis has attracted extensive research attention and rapidly rises up as a research hotspot in catalysis. In this work, we find that the Pt isolated-single-atomic-site catalyst (Pt/HNCNT) has poor selectivity. Instead, PtZn/HNCNT, where contiguous Pt atoms are isolated by Zn atoms and forming Pt-Zn bridge sites in the PtZn intermetallic particles gave a high selectivity to 4-aminophenylacetylene. This work finds that single atom catalysis doesn't work in every condition. In the selective hydrogenation of 4-nitrophenylacetylene, isolating Pt into single-atomic-sites is not enough to bear high selectivity. The neighboring Zn atoms, which form Pt-Zn bridge sites with Pt atoms in the PtZn intermetallic compound, also play important roles in the catalytic process. This work sheds light on the way to obtain efficient noble-metal catalysts through isolating the contiguous noble metal atoms and forming noble metal-non noble metal bridge sites in distinct intermetallic compounds.

The manuscript is revised to highlight this advantage of PtZn intermetallic compound to the single-atomic-sites catalysts in the main text, 1st paragraph line 5-13 of page 7 and 1st paragraph line 11-14 as well as 2nd paragraph line 1-7 of page 8 as

follows.

"AC HAADF STEM image (Supplementary Fig. 12) shows only bright dots on the support, indicating that the Pt component exists in the form of isolated-single-atomic-sites. Pt/CN, prepared by a similar method using polydopamine nanospheres as the support and treated in H_2/He atmosphere at 800 ^oC, contains obvious Pt nanoparticles, as deduced from XRD, TEM and AC HAADF STEM (Supplementary Fig. 13). These results prove that Pt atoms tend to aggregates upon high temperature, and the presence of ZnO helps to isolate contiguous Pt atoms by forming Pt-Zn bridge sites in the PtZn intermetallic nanoparticles."

"Pt-Pt bonds at around 2.4 Å were obviously observed in Pt/CN, while only a peak at 1.6 Å in the first shell was observed in Pt/HNCNT (Fig. 2c), furthering confirming that the Pt dispersed as nanoparticles and isolated-single-atomic-sites in Pt/CN and Pt/HNCNT respectively."

"CO adsorption was carried out to study the dispersion and electronic structures of Pt phase (Supplementary Fig. 19). There is a red shift of the CO stretching frequency for PtZn/HNCNT compared to Pt/CN (from 2083 cm^{-1} to 2053 cm^{-1}), indicating that the electron density is increased for PtZn/HNCNT due to the presence of zinc[37], in consistent with the observation of Pt XPS. Pt/HNCNT demonstrates a peak at 2143 cm^{-1}, further confirming that the Pt component in this sample is dispersed as isolated-single-atomic-sites[38]."

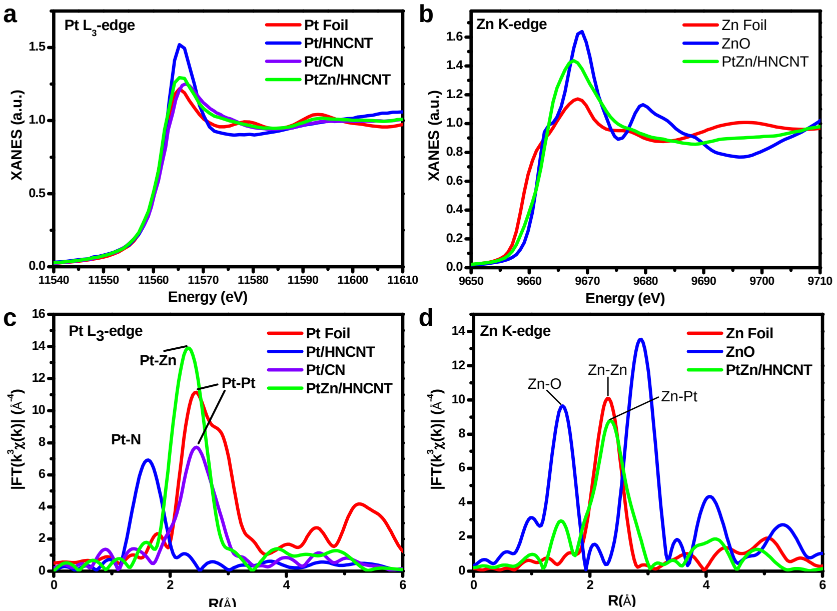

Fig. 2 | X-ray absorption spectroscopy characterization of the catalysts. a,c, The normalized XANES spectra (a) and FT-EXAFS (c) at the Pt L_3-edge of the PtZn/HNCNT, Pt/HNCNT, Pt/CN and Pt foil. b,d, The normalized XANES spectra (b) and FT-EXAFS (d) at the Zn K-edge of the PtZn/HNCNT and Zn foil.

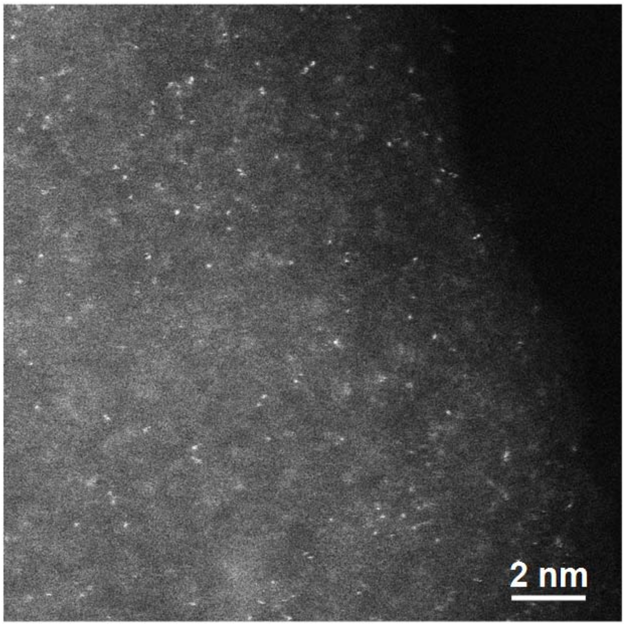

Supplementary Figure 12. AC HAADF STEM image Pt/HNCNT.

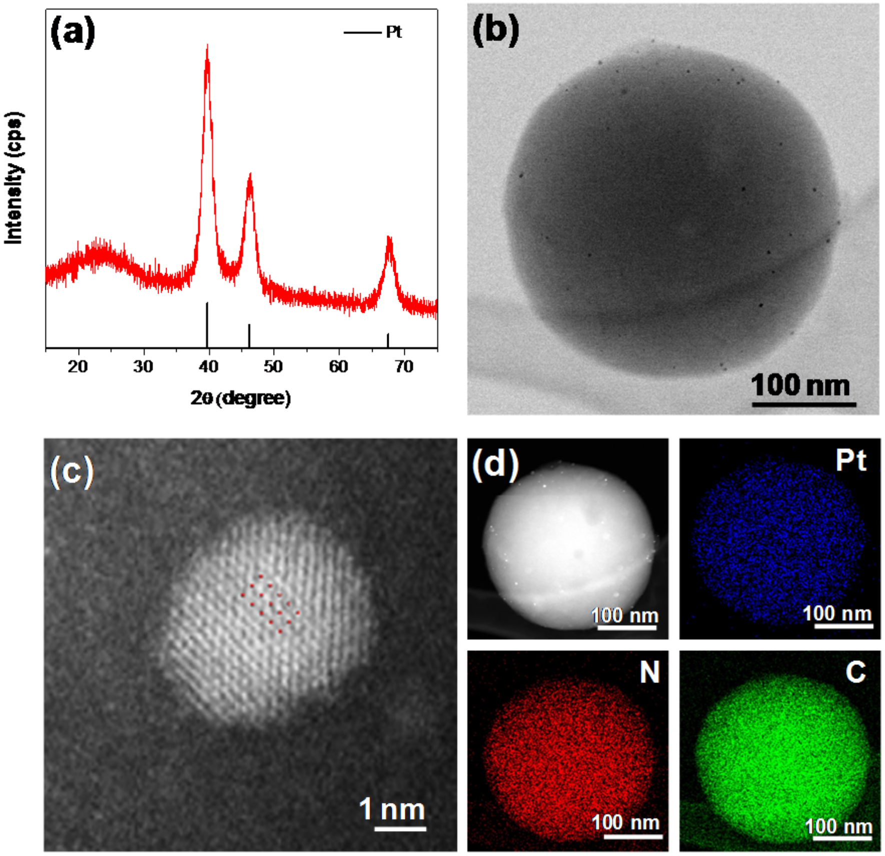

Supplementary Figure 13. Characterizations of the Pt/CN catalyst. XRD (a), TEM (b), AC HAADF STEM image (c), and elemental mappings (d) of Pt/CN.

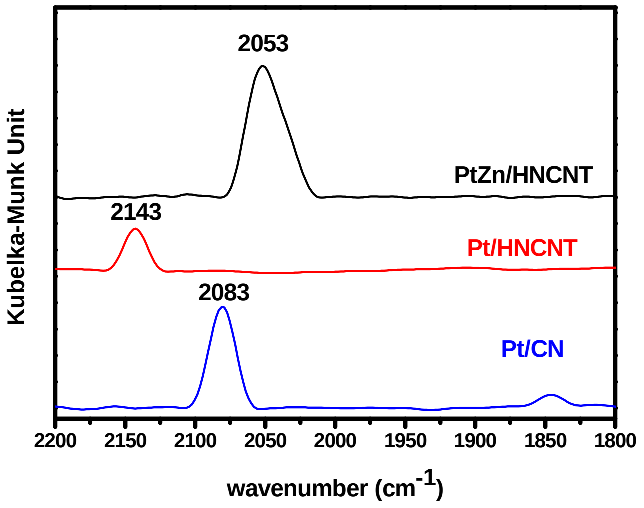

Supplementary Figure 19. *In situ* FTIR spectra of CO chemisorption for PtZn/HNCNT, Pt/HNCNT, and Pt/CN.

Reviewers' comments:

Reviewer #1 (Remarks to the Author):

The authors have addressed some points I raised previously, but, as Reviewer #3 pointed out, Pt metal segregation and structure have remained still unclear. Especially, I think the reactivity of catalysts was not properly compared because Pt structure in all catalysts seems different. I have the following major concerns on this work, rendering it not suitable for publication in Nature Communications. Please see below:
1) As shown in Figure 2 (C), the authors assigned the contribution around 2.1 A of PtZn/HNCNT to a Pt-Zn bond, while the contribution around 2.2A of Pt/CN was assigned to a Pt-Pt bond. I think the bond length difference is too small, so careful EXAFS fitting seems necessary to differentiate them. If you look at the EXAFS oscillation carefully (S. Figure 17), PtZn/HNCNT, Pt/CN, and Pt foil overlap very well different from Pt/HNCNT, potentially suggesting PtZn/HNCNT, Pt/CN, and Pt foil have similar Pt structure (nanoparticles) different from Pt/HNCNT (atomically dispersed).
2) In situ FTIR spectra in S. Figure 19 seem consistent with my 1st comment. As the authors claimed, I agree that the peak at 2143 cm-1 of Pt/HNCNT can be atomically dispersed Pt, but peaks at 2053 and 2083 cm-1 over PtZN/HNCNT and Pt/CN, respectively, are likely to be associated with Pt nanoparticles based on well-established previous studies (Boccuzzi et al. Surf. Sci. 368 (1996) 264–269, Brandt et al. Surf. Sci. 286 (1993) 15–25, Yoshida et al. Phys. Chem. Chem., Yoshida et al., Catal. Lett. 145 (2015) 606–611, Greenler et al. Surf. Sci. 271 (1992) 605–615). I do know that a change in electronic structure influence the peak position, but peaks in that wavenumber regime (2040-2080 cm-1) are associated with Pt nanoparticles, not isolated Pt species. It is possible that ZnO helps to isolate Pt species to nanoparticles (NOT to isolated sites as authors claimed) because of the lowered wavenumber over PtZn/HNCNT compared to Pt/CN (lower wavenumber peaks have lower Pt coordination number). However, if this is true, a physical Pt structure in all catalysts, influencing reactivity (unless the reaction is structure insensitive), is different. Then, I am not sure how the conclusion of the role of Zn on reactivity (facilitating adsorption of nitro group) can be validated.

Reviewer #2 (Remarks to the Author):

The comments are addressed partially, but the issue is clarified. I think it is now acceptable for publication.

Reviewer #3 (Remarks to the Author):

The authors have improved considerably the quality of the work and, compared to the first round of revisions, this amended piece covers most of the Referees' concerns. Additional requests such as an operando IR analysis can be a focus of a follow-up paper.

There is a certain tendency of the authors to self-citation. Before final acceptance and publication, it is important to cite additional literature. For example, in the first paragraph, when discussing metal catalysis and selectivity, please include more references such as:

- B. Roldan Cuenya, F. Behafarid, Surf. Sci. Rep. 2015, 70, 135-187
- M. Moliner, J. E. Gabay, C. E. Kliewer, R. T. Carr, J. Guzman, G. L. Casty, P. Serna, A. Corma, J. Am. Chem. Soc. 2016, 138, 15743-15750
- G. Vilé, S. Richard-Bildstein, A. Lhuillery, G. Rueedi, ChemCatChem 2018, 10, 3786-3794

In the second paragraph, when discussing Pt-Zn intermetallic nanocatalysts, please refer to the

following papers:

- M. Armbrüster, K. Kovnir, M. Behrens, D. Teschner, Y. Grin, R. Schlögl, J. Am. Chem. Soc. 2010, 132, 14745-14747
- L. Shao, W. Zhang, M. Armbrüster, D. Teschner, F. Girgsdies, B. Zhang, O. Timpe, M. Friedrich, R. Schlögl, D. S. Su, Angew. Chem. Int. Ed. 2011, 50, 10231-10235
- M. Friedrich, D. Teschner, A. Knop-Gericke, M. Armbrüster, J. Phys. Chem. C 2012, 116, 14930-14935
- Y. Kang, J. B. Pyo, X. Ye, T. R. Gordon, C. B. Murray, ACS Nano 2012, 6, 5642-5647
- S. Jana, J. Woong Chang, R. M. Rioux Nano Lett. 2013, 13, 3618-3625
- S. Furukawa, Y. Yoshida, T. Komatsu, ACS Catal. 2014, 4, 1441-1450

Dear all reviewers,

Thank you very much for reviewing our manuscript entitled "Isolating contiguous Pt atoms and forming Pt-Zn intermetallic nanoparticles to regulate selectivity in catalytic hydrogenation of 4-nitrophenylacetylene" (reference number: NCOMMS-18-27012B). We have carefully read your valuable comments and appreciate for your efforts which help us to improve the quality of the whole manuscript. Based on the reviewers' comments and the revision request, we have tried our best to revise the manuscript and supplementary information accordingly. The reviewers' comments are laid out below in *Italic font* and specific concerns have been numbered. Our responses are given in normal font and changes/additions to the manuscript and supplementary information are highlighted by using red colored text.

Following is our point to point answer to all the comments:

Reviewer #1 (Remarks to the Author):

The authors have addressed some points I raised previously, but, as Reviewer #3 pointed out, Pt metal segregation and structure have remained still unclear. Especially, I think the reactivity of catalysts was not properly compared because Pt structure in all catalysts seems different. I have the following major concerns on this work, rendering it not suitable for publication in Nature Communications. Please see below:

1) As shown in Figure 2 (C), the authors assigned the contribution around 2.1 Å of PtZn/HNCNT to a Pt-Zn bond, while the contribution around 2.2 Å of Pt/CN was assigned to a Pt-Pt bond. I think the bond length difference is too small, so careful EXAFS fitting seems necessary to differentiate them. If you look at the EXAFS oscillation carefully (S. Figure 17), PtZn/HNCNT, Pt/CN, and Pt foil overlap very well different from Pt/HNCNT, potentially suggesting PtZn/HNCNT, Pt/CN, and Pt foil have similar Pt structure (nanoparticles) different from Pt/HNCNT (atomically dispersed).

Response:

We sincerely appreciate the reviewer for the insightful comment and constructive suggestion. We are sorry that we didn't write clearly enough in the previous version. We would like to address this comment from the following aspects.

(1) Firstly, we want to introduce our three samples in this work more vividly through the below schemes in Figure R1. The Pt atoms distribute very differently, i.e. metallic Pt nanoparticles, Pt-N_4 single-atomic-sites, and PtZn intermetallic nanoparticles. In the case of metallic Pt nanoparticles (i.e. Pt/CN, Pt nanoparticles supported on nitrogen-doped carbon support), the Pt atoms are continuous, with high coordination number by other Pt atoms. In the case of Pt-N_4 single-atomic-site catalyst (i.e. Pt/HNCNT, Pt-N_4 supported on nitrogen-doped carbon support), the Pt atoms are coordinated by four nitrogen atoms, and stay far away from other Pt atoms. Meanwhile, in the case of PtZn intermetallic nanoparticles (i.e. PtZn/HNCNT, PtZn intermetallic nanoparticles are supported on nitrogen-doped carbon support), Pt atoms are neither as far as that in Pt

single-atomic-site catalyst nor as close as that in Pt nanoparticles. Instead, the Pt atom is isolated by one Zn atom in an ordered form in this PtZn intermetallic structure, which can be seen as a special kind of isolated Pt single-atoms that spaced by Zn atoms.

Previously, we found that isolated single-atom Pd sites in PdIn intermetallic nanostructures, where the main role of In atoms is isolating Pd into single-atom sites, demonstrated much higher selectivity for acetylene semihydrogenation compared to Pd_3In intermetallic nanostructures with Pd trimer sites (J. Am. Chem. Soc. 2017, 139, 7294-7301). This isolated single-atom Pd sites in PdIn intermetallic nanostructures exhibit similar high selectivity to ethylene as Pd single atom catalysts, such as Pd_1/C_3N_4 (Nano Res. 2017, 10, 1302-1312) and Pd_1/ND@G (J. Am. Chem. Soc. 2018, 140, 13142). From the previous work, we learned that the non-noble metal can have an isolation role to noble metal atoms. With a more in-depth thinking, we envision that in a specific intermetallic compound, the non-noble metal atoms can not only work as isolating atoms, but also have some specific influence to the noble metal atoms (such as alternating the adsorption/desorption ability of reaction species and reaction pathway), hence possibly enable different selectivity of this intermetallic compound from the noble metal nanoparticles and single-atomic site catalyst. With this target in mind, we started this work, and finally found the PtZn intermetallic nanoparticles where contiguous Pt atoms are isolated by Zn atoms and forming Pt-Zn bridge exhibited different selectivity towards 4-nitrophenylacetylene hydrogenation compared to Pt nanoparticles and Pt single-atomic-site catalyst with Pt-N_4 structure, which well testified our perspective.

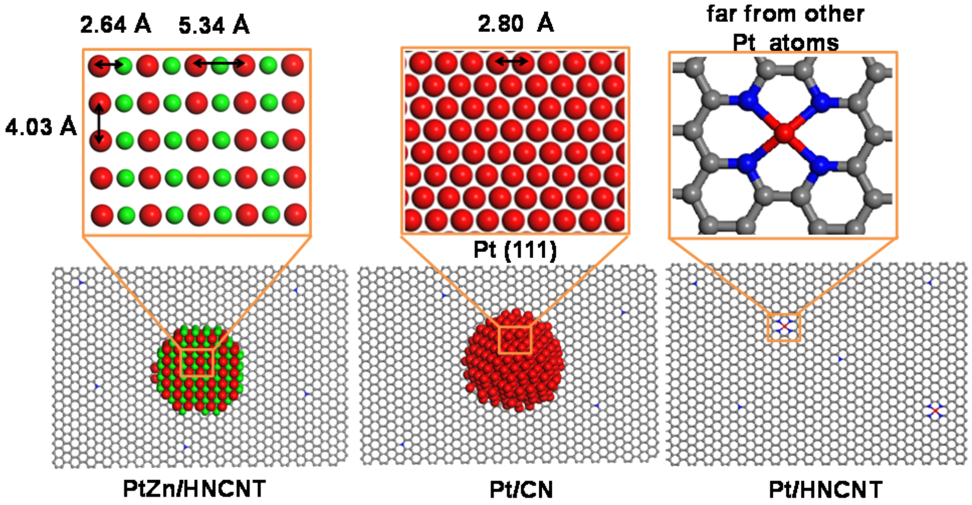

Figure R1. Schematic schemes of PtZn/HNCNT, Pt/CN and Pt/HNCNT.

(2) Thanks very much for your constructive suggestion. The bond length difference of PtZn/HNCNT and Pt/CN is indeed small, because the length difference of Pt-Zn bond in PtZn crystal structure (2.64 Å) and Pt-Pt bond in Pt crystal structure (2.80 Å) are not big. We sincerely appreciate your knowledgeable comment about

EXAFS fitting to differentiate them, which helps us to characterize the sample more clearly and improve the quality of our manuscript greatly. The fitting results are added as Supplementary Figure 19. The EXAFS of PtZn/HNCNT, Pt/CN and Pt/HNCNT are fitted well with PtZn intermetallic compound, metallic Pt, and Pt-N_4 respectively. It further confirms that Pt distribution is different in the three samples. Since the sample in PtZn/HNCNT and Pt/CN are nanoparticles, it is possible that their EXAFS oscillation have overlap.

In order to clearly state the difference of the sample, a text is added in page 8 paragraph 1 to describe the fitting results as follows.

"EXAFS fitting were further carried out to obtain quantitative structural configuration of Pt in the three samples (Supplementary Fig 19 and Table S1). The average coordination number of Pt/HNCNT is 3.8, indicating a Pt-N4 structure. The experimental curves of Pt/CN and PtZn/HNCNT could fit well with that of Pt metals and PtZn intermetallic compound respectively, furthering confirming that the Pt dispersed as nanoparticles and isolated-single-atomic-sites in Pt/CN and Pt/HNCNT respectively."

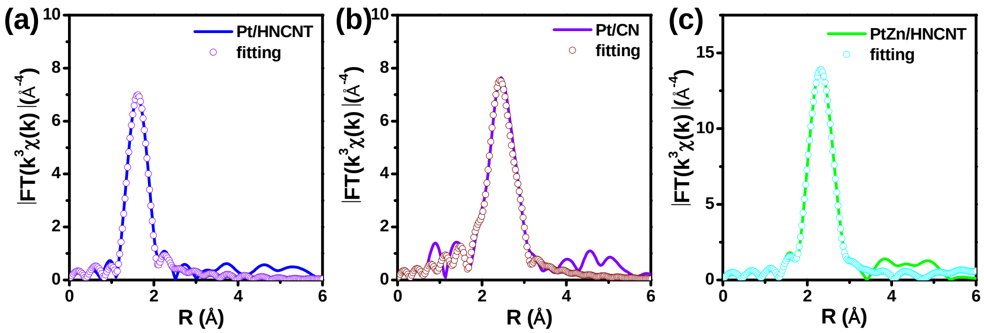

Supplementary Figure 19. The fitting results of the EXAFS spectra of Pt/HNZNT (a), Pt/CN (b), and PtZn/HNCNT (c).

Supplementary Table 1. Summary of the EXAFS fitting results of PtZn/HNCNT, Pt/CN, and Pt/HNCNT.

sample	Scattering pair	C.N.	R(Å)	$\sigma^2(10^{-3}Å^2)$	R factor
PtZn/HNCNT	Pt-Zn	5.8 ± 1.1	2.66 ± 0.03	1.0 ± 0.3	0.005
Pt/CN	Pt-Pt	10.6 ± 1.6	2.78 ± 0.01	2.8 ± 0.2	0.002
Pt/HNCNT	Pt-N	3.8 ± 0.4	1.72 ± 0.04	6.0 ± 0.3	0.003

C.N. is the coordination number; R is interatomic distance (the bond length between central atoms and surrounding coordination atoms); σ^2 is Debye-Waller factor (a measure of thermal and static disorder in absorber-scatterer distances); R factor is used to value the goodness of the fitting.

2) In situ FTIR spectra in S. Figure 19 seem consistent with my 1st comment. As the authors claimed, I agree that the peak at 2143 cm^{-1} of Pt/HNCNT can be atomically dispersed Pt, but peaks at 2053 and 2083 cm^{-1} over PtZN/HNCNT and Pt/CN, respectively, are likely to be associated with Pt nanoparticles based on well-established previous studies (Boccuzzi et al. Surf. Sci. 368 (1996) 264–269,

Brandt et al. Surf. Sci. 286 (1993) 15–25, Yoshida et al. Phys. Chem. Chem., Yoshida et al., Catal. Lett. 145 (2015) 606–611, Greenler et al. Surf. Sci. 271 (1992) 605–615). I do know that a change in electronic structure influence the peak position, but peaks in that wavenumber regime (2040-2080 cm^{-1}) are associated with Pt nanoparticles, not isolated Pt species. It is possible that ZnO helps to isolate Pt species to nanoparticles (NOT to isolated sites as authors claimed) because of the lowered wavenumber over PtZn/HNCNT compared to Pt/CN (lower wavenumber peaks have lower Pt coordination number). However, if this is true, a physical Pt structure in all catalysts, influencing reactivity (unless the reaction is structure insensitive), is different. Then, I am not sure how the conclusion of the role of Zn on reactivity (facilitating adsorption of nitro group) can be validated.

Response:

We sincerely appreciate the reviewer for the comments. We will answer your comment from two aspects.

(1) The sample PtZn/HNCNT is consisted of PtZn intermetallic nanoparticles, where Pt is isolated by Zn atoms but is not as far as that in Pt/HNCNT (Pt isolated-single-atomic-sites supported on nitrogen doped carbon). On one hand, many references have reported that the PtZn intermetallic nanoparticles or their similarities such as PtSn and PdZn, have in situ FTIR peaks during 2040-2080 cm^{-1}, which are listed below in Table R1. On the other hand, the peak would shift to lower wavenumber due to the electronic effect of Zn to Pt. Therefore, we think that the in situ FTIR spectra of PtZn/HNCNT at 2053 cm^{-1} is reasonable.

Table R1. Summary of in situ FTIR peaks on intermetallic compounds in literatures.

No.	Sample	Wavelength Number (cm^{-1})	Ref
1	PtZn/ZnO	2053	J. Catal. 2004, 221, 32-42.
2	Pt@mSiO_2<br>PtSn@mSiO_2	2072<br>2060	J. Catal. 2017, 356, 307-314.
3	Pd/ZnO<br>ZnPd/ZnO	2084<br>2068	J. Phys. Chem. C 2018, 122, 12395-12403.
4	Pd/Al_2O_3<br>PdZn	2076<br>2054	ACS Catal. 2016, 6, 1054-1061.

To avoid the misunderstanding, we revise the manuscript as follows.

- "noble metal atoms might be isolated into **single atomic site** on intermetallic structures" is revised as "noble metal atoms might be isolated into single atom on intermetallic structures" in paragraph 2 of page 3.

(2) The role of Zn on reactivity is validated by DFT calculations. DFT calculations were carried out to distinguish the adsorption energies of 4-NPA on PtZn IMCs and Pt metals. We calculated the adsorption energy of different configurations on PtZn($02\bar{2}$) surface and Pt(111) surface and found that the Zn could facilitate the

nitro group adsorption. The detailed explanation is written in paragraph 1 page 12 and paragraph 1 page 13 in the manuscript. For your convenience, we show it bellow.

"The optimized geometry of 4-NPA@Pt(111) is shown in Supplementary Fig. 34. The distances between nitro group, aromatic ring, alkynyl group and metal surface decrease gradually. The configuration which nitro group is most close to Pt surface was also considered (Supplementary Fig. 34), however, this configuration is 94 kJ/mol less stable. For PtZn($02\bar{2}$) surface, the shortest distance between Pt atom in the same layer is 4.026 Å, which is significantly longer than it in Pt(111) surface (2.804 Å). Meanwhile the shortest distance between Pt and Zn in the same layer is 2.639 Å. In this sense, the Pt atom on the surface was isolated partly by two nearest-neighboring Zn atoms. The difference in electronegativity between Pt and Zn implies that the Pt atoms and Zn atoms in PtZn are negatively charged and positively charged, respectively, which is confirmed by the calculated charge population. As shown in Supplementary Fig. 34, maintaining more carbon atoms in both aromatic ring and alkynyl group close to Pt atoms and oxygen atoms in nitro close to Zn could make the configuration more stable. The adsorption configurations of 4-NPA at the Pt isolated-single-atomic-site (Pt-N-C) was also calculated and found that the one with the aromatic ring adsorbed on the Pt-N4 are more stable, but the difference between different mode was not big (Supplementary Fig. 36)."

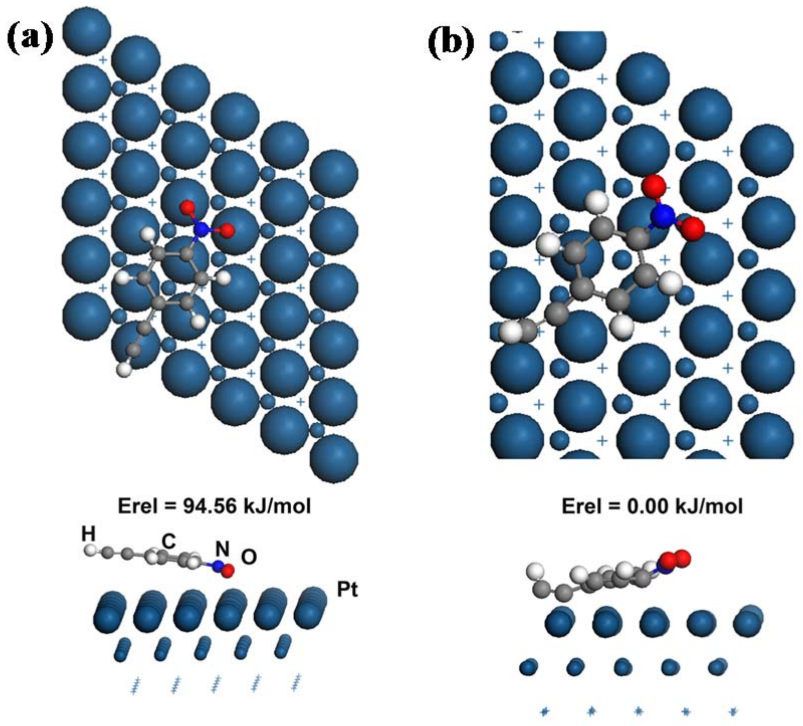

Supplementary Figure 34. Top and side view of adsorption configurations for 4-NPA@Pt(111) surface. The right configuration (b) is more stable.

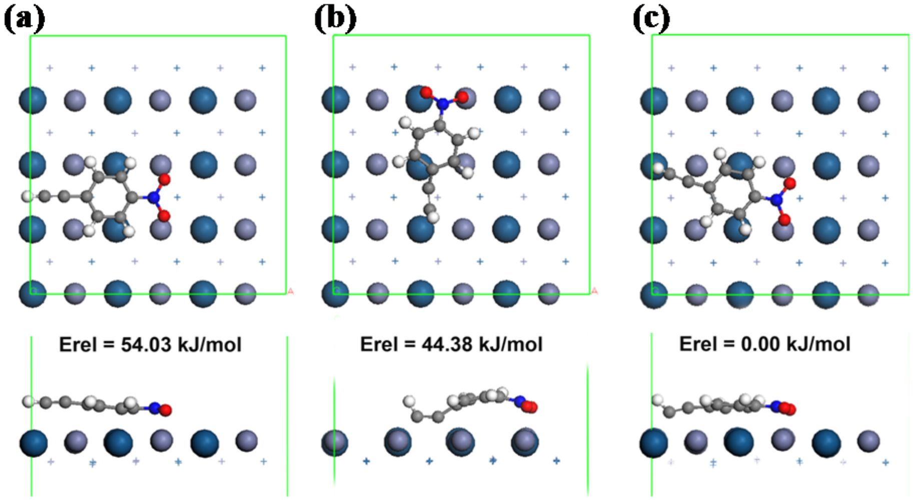

Supplementary Figure 35. Top and side view of adsorption configurations for 4-NPA@PtZn($02\bar{2}$) surface. (Zn: grey). The right configuration (c) is more stable.

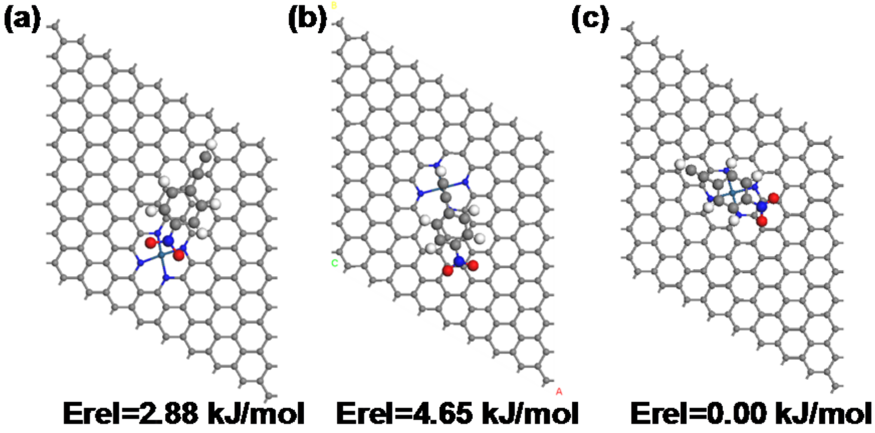

Supplementary Figure 36. Different adsorption configurations for 4-NPA@Pt-N-C surface. The right configuration (c) is more stable.

Reviewer #2:

The comments are addressed partially, but the issue is clarified. I think it is now acceptable for publication.

Response:

We sincerely appreciate the reviewer for the comments raised in the three rounds, which help us to improve the quality of our manuscript. We are glad that the responses are satisfactory to you. Thanks very much for your insightful comments again.

Reviewer #3:

The authors have improved considerably the quality of the work and, compared to the first round of revisions, this amended piece covers most of the Referees' concerns. Additional requests such as an operando IR analysis can be a focus of a follow-up paper.

There is a certain tendency of the authors to self-citation. Before final acceptance and publication, it is important to cite additional literature. For example, in the first paragraph, when discussing metal catalysis and selectivity, please include more references such as:

- B. Roldan Cuenya, F. Behafarid, Surf. Sci. Rep. 2015, 70, 135-187
- M. Moliner, J. E. Gabay, C. E. Kliewer, R. T. Carr, J. Guzman, G. L. Casty, P. Serna, A. Corma, J. Am. Chem. Soc. 2016, 138, 15743-15750
- G. Vilé, S. Richard□Bildstein, A. Lhuillery, G. Rueedi, ChemCatChem 2018, 10, 3786-3794

In the second paragraph, when discussing Pt-Zn intermetallic nanocatalysts, please refer to the following papers:

- M. Armbrüster, K. Kovnir, M. Behrens, D. Teschner, Y. Grin, R. Schlögl, J. Am. Chem. Soc. 2010, 132, 14745-14747
- L. Shao, W. Zhang, M. Armbrüster, D. Teschner, F. Girgsdies, B. Zhang, O. Timpe, M. Friedrich, R. Schlögl, D. S. Su, Angew. Chem. Int. Ed. 2011, 50, 10231-10235
- M. Friedrich, D. Teschner, A. Knop-Gericke, M. Armbrüster, J. Phys. Chem. C 2012, 116, 14930-14935
- Y. Kang, J. B. Pyo, X. Ye, T. R. Gordon, C. B. Murray, ACS Nano 2012, 6, 5642-5647
- S. Jana, J. Woong Chang, R. M. Rioux Nano Lett. 2013, 13, 3618-3625
- S. Furukawa, Y. Yoshida, T. Komatsu, ACS Catal. 2014, 4, 1441-1450

Response:

We thank the reviewer and appreciate the suggestions and comments very much.

(1) We will put more efforts to the operando IR analysis in the follow-up work and focus on this point in a follow-up paper. We sincerely appreciate your insightful suggestion, which helps us to get more deep and comprehensive understanding about this PtZn system.

(2) We appreciate your pointing these references to us. According to your suggestion, the important references you mentioned has been cited in the right place in our revised manuscript.

"13 Cuenya, B. R. & Behafarid, F. Nanocatalysis: size- and shape-dependent chemisorption and catalytic reactivity. *Surf. Sci. Rep.* **70,** 135-187 (2015).

14 Moliner, M. Gabay, *et al.* Reversible Transformation of Pt Nanoparticles into Single Atoms inside High-Silica Chabazite Zeolite. *J. Am. Chem. Soc.* **138**, 15743-15750 (2016).

15 Vilé, G., Richard-Bildstein, S., Lhuillery, A. & Rueedi, G. Electrophile, Substrate Functionality, and Catalyst Effects in the Synthesis of α-Mono and Di-Substituted Benzylamines via Visible□Light Photoredox Catalysis in Flow. *ChemCatChem* **10**, 3786-3794 (2018).

39 Armbrüster, M., Kovnir, K., Behrens, M., Teschner, D., Grin, Y. & Schlögl, R. Pd-Ga Intermetallic Compounds as Highly Selective Semihydrogenation Catalysts. *J. Am. Chem. Soc.* **132**, 14745-14747 (2010).

40 Shao, L. *et al.* Nanosizing Intermetallic Compounds Onto Carbon Nanotubes: Active and Selective Hydrogenation Catalysts. *Angew. Chem. Int. Ed.* **50**, 10231-10235 (2011).

41 Friedrich, M., Teschner, D., Knop-Gericke, A. & Armbrüster, M. Surface and Subsurface Dynamics of the Intermetallic Compound ZnNi in Methanol Steam Reforming. *J. Phys. Chem. C* **116**, 14930-14935 (2012).

42 Kang, Y., Pyo, J. B., Ye, X., Gordon, T. R. & Murray, C. B. Synthesis, Shape Control, and Methanol Electro-oxidation Properties of Pt-Zn Alloy and Pt_3Zn Intermetallic Nanocrystals. *ACS Nano* **6**, 5642-5647 (2012).

43 Jana, S., Chang, J. W. & Rioux R. M. Synthesis and Modeling of Hollow Intermetallic Ni−Zn Nanoparticles Formed by the Kirkendall Effect. *Nano Lett.* **13**, 3618-3625 (2013).

44 Furukawa, S., Yoshida, Y. & Komatsu, T. Chemoselective Hydrogenation of Nitrostyrene to Aminostyrene over Pd- and Rh-Based Intermetallic Compounds. *ACS Catal.* **4**, 1441-1450 (2014)."

REVIEWERS' COMMENTS:

Reviewer #1 (Remarks to the Author):

The points I have raised in the previous round of review have been satisfactorily addressed by authors and now I recommend it for publication.
